# Bioactive Compounds from *Euphorbia usambarica* Pax. with HIV-1 Latency Reversal Activity

**DOI:** 10.3390/ph14070653

**Published:** 2021-07-07

**Authors:** Yu-Chi Tsai, Racheal A. Nell, Jonathan E. Buckendorf, Norbert Kúsz, Peter Waweru Mwangi, Róbert Berkecz, Dóra Rédei, Andrea Vasas, Adam M. Spivak, Judit Hohmann

**Affiliations:** 1 Interdisciplinary Excellence Centre, Department of Pharmacognosy, University of Szeged, H-6720 Szeged, Hungary; yuchi0713@gmail.com (Y.-C.T.); kusznorbert@gmail.com (N.K.); redei@pharmacognosy.hu (D.R.); vasasa@pharmacognosy.hu (A.V.); 2 Department of Medicine, University of Utah School of Medicine, Salt Lake City, UT 84132, USA; racheal.nell@outlook.com (R.A.N.); jonny.buckendorf@path.utah.edu (J.E.B.); 3 Department of Medical Physiology, School of Medicine, University of Nairobi, Nairobi P.O. Box 30197-00100, Kenya; waweruk2001@gmail.com; 4 Department of Medical Chemistry, University of Szeged, H-6720 Szeged, Hungary; berkecz.robert@szte.hu; 5 Interdisciplinary Centre of Natural Products, University of Szeged, H-6720 Szeged, Hungary

**Keywords:** *Euphorbia usambarica*, diterpenoid, usambariphane, HIV, latency reactivation, latency reversal agent, PKC

## Abstract

*Euphorbia usambarica* is a traditional medicine used for gynecologic, endocrine, and urogenital illnesses in East Africa; however, its constituents and bioactivities have not been investigated. A variety of compounds isolated from *Euphorbia* species have been shown to have activity against latent HIV-1, the major source of HIV-1 persistence despite antiretroviral therapy. We performed bioactivity-guided isolation to identify 15 new diterpenoids (**1**–**9**, **14**–**17**, **19**, and **20**) along with 16 known compounds from *E. usambarica* with HIV-1 latency reversal activity. Euphordraculoate C (**1**) exhibits a rare 6/6/3-fused ring system with a 2-methyl-2-cyclopentenone moiety. Usambariphanes A (**2**) and B (**3**) display an unusual lactone ring constructed between C-17 and C-2 in the jatrophane structure. 4*β*-Crotignoid K (**14**) revealed a 250-fold improvement in latency reversal activity compared to crotignoid K (**13**), identifying that configuration at the C-4 of tigliane diterpenoids is critical to HIV-1 latency reversal activity. The primary mechanism of the active diterpenoids **12**–**14** and **21** for the HIV-1 latency reversal activity was activation of PKC, while lignans **26** and **27** that did not increase CD69 expression, suggesting a non-PKC mechanism. Accordingly, natural constituents from *E. usambarica* have the potential to contribute to the development of HIV-1 eradication strategies.

## 1. Introduction

Antiretroviral therapy (ART) durably blocks HIV-1 transcription by targeting viral enzymes; however, these drugs do not result in viral eradication due to the presence of replication-competent proviruses that are stably integrated into the genomes of a small population of long-lived memory T cells, known as the latent reservoir [1]. A promising strategy to address HIV-1 persistence is to use small molecules to reactivate latent proviruses in order to expose these cells to immune clearance and/or viral cytopathic effect. Natural products offer much promise regarding the discovery of new latency reversal agents (LRAs) for HIV-1 eradication [2,3,4].

The *Euphorbia* is one of the largest genera in Euphorbiaceae [5,6]. There are many bioactive secondary metabolites in the genus *Euphorbia*, including more than 20 different types of diterpenoids (abietane, atisane, casbane, daphnane, ingenane, jatrophane, karane, lathyrane, tigliane, and others) [7]. Moreover, sesquiterpenoids, triterpenoids, flavonoids, alkaloids, polyphenols, tannins, volatile compounds, and phytosterols have also been discovered in *Euphorbia* species, many of which are in active use as traditional medicines [8,9,10]. The pharmacological effects of *Euphorbia* species are related to anti-inflammatory [11], multidrug-resistance-reversing [12,13], antiviral [14,15], cytotoxic [16,17], anti-arrhythmic [18], antifungal [19], anti-thrombotic [20], antiallergic [21], and muscle relaxant [22] properties.

In past, several *Euphorbia* plants have previously been evaluated to determine their efficacy as LRAs [23,24,25,26,27,28,29,30]. For instance, Liu et al. reported the effects on HIV-1 transcription of ingenane esters 3-angeloylingenol and 3-(2-naphthoyl)ingenol from *E. kansui*, which can reactivate latent HIV with EC_50_ values at 4.2 and 2.4 nM, respectively [28]. Yan et al. published atisane diterpenoids euphorneroid D and *ent*-3-oxoatisan-16*α*,17-acetonide from *E. neriifolia* which showed anti-HIV-1 activities with EC_50_ values at 34 and 24 μM, respectively [29]. Valadão et al. established deoxyphorbol esters from *E. umbellata* which increased HIV-1 latency reactivation through NF-κB activation, nuclear translocation, and HIV-1 LTR promoter [30].

*Euphorbia usambarica* Pax. distributes mainly in East Africa [31] and is a large branching shrub as well as used as a traditional medicine for gynecologic, endocrine, and urogenital illnesses [32,33]. In our preliminary study, we found that the whole plant extract of *E. usambarica* showed a significant HIV-1 latency reversal activity. However, there was no study related to the chemical constituents and bioactivities of *E. usambarica* in the reported literature. In addition, the prevention and treatment of HIV infection and acquired immune deficiency syndrome (AIDS) are still the central issues around the world. Therefore, we would investigate the active constituents and the pharmacological effect of *E. usambarica*. Further, we sought to test its aqueous, and organic fractions for HIV-1 latency reversal activity and cytotoxicity. Dichloromethane and *n*-hexane fractions showed increased activity compared to the whole plant extract (EU) in dose-response analysis. Further sub-fractionation of the active fractions was followed by compounds purification and identification using multistep chromatography, NMR, and mass spectroscopy to yield 31 purified compounds. Six of those compounds demonstrated HIV-1 anti-latency activity. Extended dose-response curves were then generated for these compounds. Several of these compounds have no previously described anti-HIV-1 or anti-latency activity. These results support further exploration of medicinal plants, and *Euphorbia* species in particular, as sources of new means to address HIV-1 persistence.

## 2. Results

### 2.1. Structure Elucidation of New Compounds

**Figure 1:**
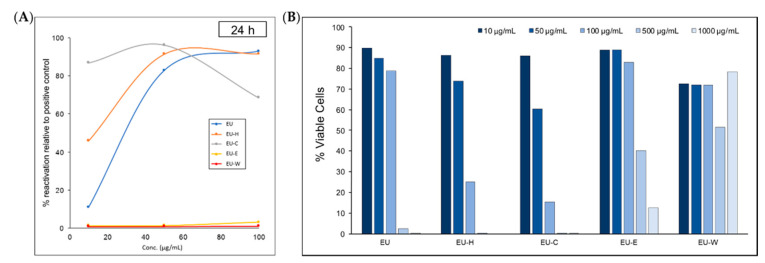
HIV-1 latency reversal activity of methanolic crude extract (EU), partitioned *n*-hexane (EU-H), dichloromethane (EU-C), ethyl acetate (EU-E), and water-soluble residue (EU-W) phases. (**A**) dose-response experiments conducted with Jurkat T cells that were latently infected with full-length HIV-1 reporter construct (J-Lat 10.6 cells), HIV-1 reactivation quantified as % of positive control (PMA); (**B**) cell viability of each sample at 10, 50, 100, 500, and 1000 μg/mL.

**Figure 2:**
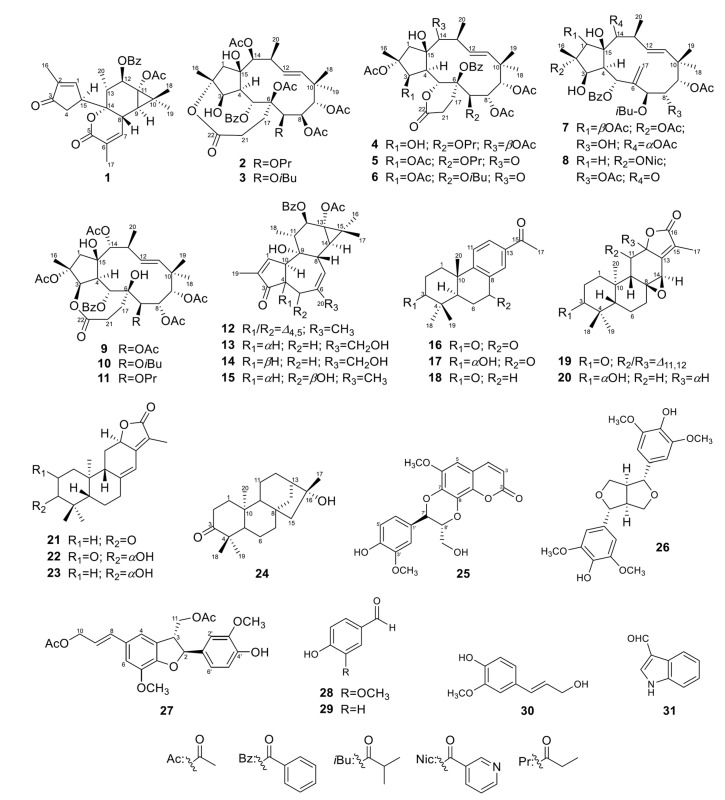
Structures of compounds **1**–**31** isolated from *E. usambarica*.

The partitioned *n*-hexane (EU-H) and dichloromethane (EU-C) phases significantly improved upon reactivation efficacy compared to the EU. The EU-H reactivated latent HIV-1 to 91% at concentrations of 50 and 100 μg/mL. The EU-C phase reactivated latent HIV-1 up to 86% at 10 μg/mL concentration and 98% at 50 μg/mL. The partitioned ethyl acetate (EU-E) and water-soluble residue (EU-W) phases did not appear to have any activity (Figure 1A). Cell viability declined steeply above concentrations of 100 μg/mL. Significant toxicity at concentrations above 100 μg/mL limits conclusions about reactivation. The lower concentrations of the EU-H and EU-C fractions did not affect toxicity but markedly improved viral reactivation (Figure 1B). Due to the high reactivation ratio (86%) at the lowest tested concentration (10 μg/mL), the EU-C phase was selected for bioactivity-guided isolation. This led to identification of 15 new diterpenoids (**1**–**9**, **14**–**17**, **19**, and **20**) along with 16 known compounds (**10**–**13**, **18**, and **21**–**31**) (Figure 2).

#### 2.1.1. Euphordraculoate C (**1**)

**Figure 3:**
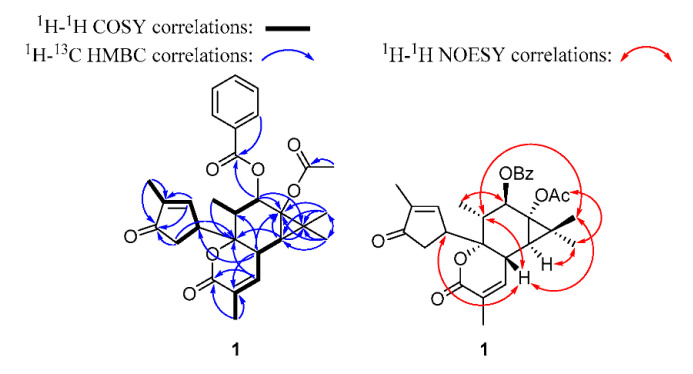
The ^1^H-^1^H COSY, key HMBC, and NOESY correlations of compound **1**.

Compound **1** was purified as a colorless gum with ${\left[\alpha \right]}_{D}^{28}$ −63 (*c* 0.05, CHCl_3_). The molecular formula was identified as C_29_H_32_O_7_ by HR-ESIMS *m/z* 493.2237 [M + H]^+^ (calcd. for C_29_H_33_O_7_ 493.2221), indicating 14 unsaturated degrees. The ^1^H NMR spectrum of **1** revealed six methyls, an oxygenated methine, two unsaturated methines, and a monosubstituted aromatic group (Table 1). The ^13^C-JMOD spectrum of **1** evidenced 29 carbon signals, including six methyls, one methylene, seven olefinic methines, one oxygenated methine, four saturated methines, one quaternary, three olefinic quaternary, two oxygenated quaternary, and four carbonyl carbons (Table 2). According to the combination of the 1D and 2D NMR spectra, one benzoyloxy group (OBz) [*δ*_H_ 8.02 (2H), 7.58, 7.46 (2H); *δ*_C_ 166.2, 133.2, 130.4, 129.9 (2C), 128.6 (2C)] and one acetoxy (OAc) (*δ*_H_ 1.97; *δ*_C_ 170.4, 21.1) could be identified in **1**. Based on the COSY and HSQC spectra of **1**, a series of COSY correlations between an olefinic methine (*δ*_H_ 7.52, CH-1)/a methine (*δ*_H_ 3.80, CH-15)/methylene (*δ*_H_ 2.52, 2.24, CH_2_-4), together with the allylic four-bond coupling between H-1 and a methyl group [*δ*_H_ 1.74 (3H), CH_3_-16]. Key HMBC correlations from H-1 and H-16 to an olefinic quaternary carbon C-2 (*δ*_C_ 141.3) and a ketone carbon C-3 (*δ*_C_ 206.8), and H-4 to C-3, indicated the presence of an *α*-methyl-*α*,*β*-unsaturated cyclopentanone moiety. Moreover, a series of COSY correlations between a methine (*δ*_H_ 2.37, CH-8), an olefinic methine (*δ*_H_ 6.68, CH-7), and an allylic coupled methyl group [*δ*_H_ 2.02 (3H), CH_3_-17], together with the key HMBC correlations from H-7 to a carbonyl carbon C-5 (*δ*_C_ 163.4) and an oxygenated quaternary carbon C-14 (*δ*_C_ 85.8), H-8 to an olefinic quaternary carbon C-6 (*δ*_C_ 127.8), and H_3_-17 to C-5, C-6, and C-7, indicated the presence of an *α*-methyl-*α*,*β*-unsaturated-*δ*-lactone moiety. A *gem*-dimethylcyclopropane moiety could be identified by the key HMBC correlations from two methyl groups [*δ*_H_ 1.43 (3H), *δ*_C_ 16.5, CH_3_-18; *δ*_H_ 1.16 (3H), *δ*_C_ 24.8, CH_3_-19] to a methine C-9 (*δ*_H_ 1.02, *δ*_C_ 34.0), a quaternary carbon C-10 (*δ*_C_ 24.9), an oxygenated quaternary carbon C-11 (*δ*_C_ 63.4) and each other, and H-9 to C-10 and C-11. In addition, the ^1^H–^1^H COSY cross peak between H-8/H-9, a methyl group [*δ*_H_ 0.91 (3H), CH_3_-20]/a methine (*δ*_H_ 2.10, CH-13)/an oxygenated methine (*δ*_H_ 5.86, CH-12), as well as the HMBC correlations from H-7 to C-14, H-8 to C-11 and C-13, H-9 to C-11 and C-14, H-12 to C-10 and C-11, and C-13 to C-14, demonstrated the presence of a six-membered ring fusion with the *gem*-dimethyl-cyclopropane moiety at C-9 and C-11, and the *α*-methyl-*α*,*β*-unsaturated-*δ*-lactone moiety at C-8 and C-14. The HMBC correlations from H-4 to C-14 and H-8 to C-15 indicated that the *α*-methyl-*α*,*β*-unsaturated cyclopentanone moiety was linked to C-14. The OAc and OBz groups should be connected to C-11 and C-12, respectively, based on HMBC correlations (Figure 3). Additionally, comparing the NMR data of **1** with those of euphordraculoate A [34] suggested the same rare diterpenoid skeleton of both compounds. According to the NOESY cross-peaks between H-8/H-13, H-8/H_3_-18, H-8/H-15, H-9/H_3_-19, 11-OAc/H_3_-19, H-12/H_3_-20, and H-13/H_3_-18, as well as comparing with euphordraculoate A [34] and euphodendriane A [35], the relative configuration of **1** was established as shown on structural formula (Figure 3), and the compound was named as euphordraculoate C.

#### 2.1.2. Usambariphane A (**2**)

Compound **2** was obtained as a white amorphous powder. Its molecular formula was calculated as C_40_H_52_O_16_ by the analysis of HR-ESIMS *m/z* 789.3327 [M + H]^+^ (calcd. for C_40_H_53_O_16_ 789.3328). The NMR spectra of **2** revealed clearly four OAc (*δ*_H_ 2.56, *δ*_C_ 174.3, 21.4; *δ*_H_ 2.17, *δ*_C_ 170.2, 22.7; *δ*_H_ 2.14, *δ*_C_ 169.8, 21.5; *δ*_H_ 2.08, *δ*_C_ 170.0, 21.6), one OBz (*δ*_H_ 7.93 (2H), 7.52, 7.39 (2H), *δ*_C_ 164.5, 133.5, 129.7, 129.6, 128.8), one propionate group [*δ*_H_ 2.41 (2H), 1.16 (3H), *δ*_C_ 174.2, 27.7, 8.9; OPr], four methyls [*δ*_H_ 1.73 (3H), *δ*_C_ 19.1, CH_3_-16; *δ*_H_ 0.93 (3H), *δ*_C_ 26.0, CH_3_-18; *δ*_H_ 1.13 (3H), *δ*_C_ 21.7, CH_3_-19; *δ*_H_ 1.14 (3H), *δ*_C_ 23.2, CH_3_-20], a *trans*-disubstituted C=C (*δ*_H_ 5.49, *δ*_C_ 134.6, CH-11; *δ*_H_ 5.83, *δ*_C_ 133.0, CH-12), and a lactone carbonyl carbon (*δ*_C_ 175.1, C-22). Further, comparing the 1D NMR data of **2** (Table 1 and Table 2) with those of isoterracinolide A (**10**) [36], the skeleton of **2** was established as a dihomojatrophane type diterpenoid [7] with a double bond at *∆*_11,12_ and a lactone moiety. An OH group was located at C-3 based on a ^1^H–^1^H COSY cross peak between H-3 and 3-OH, as well as the HBMC correlations from 3-OH to C-3 and C-4. Another OH group was connected to C-15 by the confirmation of the HMBC correlations from 15-OH to C-4, C-14, and C-15. Moreover, the HMBC correlations from H-5 to *δ*_C_ 164.5, H-7 to *δ*_C_ 174.2, H-8 to *δ*_C_ 170.0, H-9 to *δ*_C_ 170.2, H-14 to *δ*_C_ 174.3, indicated that OBz and OPr were located at C-5 and C-7 respectively, and three OAc were linked to C-8, C-9, and C-14 each. The last OAc was located apparently to C-6 based on NOESY correlations between the acetyl proton signal *δ*_H_ 2.14 (6-OAc) with H-5 and H-17a. The remaining lactone ring was proposed to be constructed between C-17 and C-2 in structure **2**. According to the ^13^C signal value of C-6 (*δ*_C_ 92.5) of **2** was close to the signal in sororianolide A (*δ*_C_ 93.0, C-6-*β*OAc) and different from sororianolide B (*δ*_C_ 80.9, C-6-*α*OAc), suggesting the OAc at C-6 in **2** can be assigned as *β*-oriented [37]. Moreover, the NOESY correlations of H-3/H_2_-17, H-3/H-4, H-4/H-7, H-4/H-8, H-8/H_3_-19, H_3_-19/H-13, H_3_-19/H-14, H_3_-16/3-OH, H_3_-16/H-1b, H-1b/15-OH, 15-OH/H-9, and H-9/H_3_-18 indicted the configurations of 3*β*-OH, H*α*-4, H*β*-5, 6*β*-OAc, 7*β*-OPr, 8*β*-OAc, 9*α*-OAc, 14*β*-OAc, 15-*β*OH, *β*CH_3_-16, and *β*CH_3_-20. Thus, the structure of **2** was established and named as usambariphane A.

#### 2.1.3. Usambariphane B (**3**)

Compound **3** was obtained as a white amorphous powder. The molecular formula was determined as C_41_H_54_O_16_ based on HR-ESIMS m/z 803.3488 [M + H]^+^ (calcd. for C_41_H_55_O_16_ 803.3485). The 1D NMR data (Table 1 and Table 2) of 3 were highly similar to those of 2, except for an isobutyryl group (OiBu) [δ_H_ 2.61, 1.21 (3H), 1.20 (3H), δ_C_ 176.4, 34.2, 18.8, 18.5] instead of propanoyl. The isobutyryl group was connected to C-7 in 3 based on the HMBC correlation from H-7 (δ_H_ 5.38) to the OiBu carbonyl carbon (δ_C_ 176.4). The NOESY correlations of 3 revealed the same relative configuration as that of 2. The structure of 3 was established and named as usambariphane B.

#### 2.1.4. Usambariphane C (**4**)

Compound **4** was purified as a colorless crystal. The molecular formula was identified as C_40_H_52_O_16_ by HR-ESIMS *m/z* 789.3346 [M + H]^+^ (calcd. for C_40_H_53_O_16_ 789.3328). Based on the comparison of the ^1^H and ^13^C NMR data (Table 1 and Table 2) for **4** with those of usambariphane B (**2**), the skeleton of **4** was suggested to be a C_22_ dihomojatrophane with a double bond at ∆_11,12_ (*δ*_H_ 5.41, *δ*_C_ 135.3, CH-11; *δ*_H_ 5.72, *δ*_C_ 134.1, CH-12) and a lactone moiety (*δ*_H_ 3.09, 2.40, *δ*_C_ 23.4, CH_2_-17; *δ*_H_ 2.65, 2.16, *δ*_C_ 26.0, CH_2_-21; *δ*_C_ 168.1, C-22). A *δ*-lactone ring was constructed at C-5 and C-6 supporting by the ^1^H–^1^H COSY cross peak between H_2_-17 and H_2_-21, as well as the HMBC correlations from H-5 to C-17 and C-22, H_2_-17 to C-5, C-6, and C-22, and H_2_-21 to C-6 and C-22. Moreover, the 1D NMR data of **4** were highly close to those of euphosorophane D [38] except for an OPr group [*δ*_H_ 2.57, 2.50, 1.22 (3H); *δ*_C_ 174.6, 27.5, 8.9] at C-7 according to an HMBC correlation from H-7 (*δ*_H_ 5.40) to *δ*_C_ 174.6. The NOESY cross-peaks of **4** demonstrated the same relative orientations to those of euphosorophane D [38]. Therefore, the structure of **4** was established and named as usambariphane C.

#### 2.1.5. Usambariphane D (**5**)

Compound **5** was purified as a colorless crystal. The molecular formula was identified as C_40_H_50_O_16_ by HR-ESIMS *m/z* 787.3193 [M + H]^+^ (calcd. for C_40_H_51_O_16_ 787.3172). The inspection of 1D (Table 1 and Table 2) and 2D NMR data suggested that compound **5** was a bishomojatrophane type diterpenoid with a double bond at *∆*_11,12_ (*δ*_H_ 6.16, *δ*_C_ 137.4, CH-11; *δ*_H_ 5.43, *δ*_C_ 128.9, CH-12), a lactone moiety (*δ*_H_ 2.72, 2.01, *δ*_C_ 26.5, CH_2_-17; *δ*_H_ 3.43, 2.50, *δ*_C_ 29.1, CH_2_-21; *δ*_C_ 172.7, C-22), and a ketone unit *δ*_C_ 211.4 (C-14). A *δ*-lactone ring was constructed at C-5 and C-6 supporting by the ^1^H–^1^H COSY cross peak between H_2_-17 and H_2_-21, and the HMBC correlations from H-5 to C-17 and C-22, H_2_-17 to C-5, C-6, and C-22, and H_2_-21 to C-6 and C-22. The ketone unit in **5** was located at C-14 based on the HMBC correlations from H-1, H-12, H-13, and H_3_-20 to C-14, respectively. An OH group was connected to C-15 by the confirmation of HMBC correlations from 15-OH to C-4, C-14, and C-15. Moreover, four OAc [*δ*_H_ 2.26 (3H), *δ*_C_ 169.6, 22.4; *δ*_H_ 2.05 (3H), *δ*_C_ 169.1, 20.6; *δ*_H_ 2.03 (3H), *δ*_C_ 169.9, 20.9; *δ*_H_ 2.00 (3H), *δ*_C_ 170.0, 21.2], one OBz [*δ*_H_ 7.88 (2H), 7.65, 7.51 (2H), *δ*_C_ 165.8, 133.8, 130.6, 129.7 (2C), 128.5 (2C)], and one OPr [*δ*_H_ 2.49, 2.31, 1.23 (3H), *δ*_C_ 173.4, 27.6, 8.6] moieties were identified clearly by the examination of the NMR spectra. The HMBC correlations from H-3 to *δ*_C_ 169.1, H-7 to *δ*_C_ 173.4, H-8 to *δ*_C_ 170.0, and H-9 to *δ*_C_ 169.9, indicated the OPr was located at C-7, and three OAc were linked to C-3, C-8, and C-9, respectively. The location of the OBz at C-6 was confirmed by the NOESY correlations between the benzoyl proton signal *δ*_H_ 7.88 with H-5, H-8, and H-12. The last OAc was connected to C-2 based on the NOE cross-peak between the acetyl proton signal *δ*_H_ 2.26 with H_3_-16. The relative configuration of **5** was evaluated by the NOESY spectrum and comparison with a similar structure terracinolide J [39] to assign 2*α*-OAc, 3*β*-OAc, H*α*-4, H*β*-5, 6*β*-OBz, 7*β*-OPr, 8*α*-OAc, 9*α*-OAc, *β*CH_3_-20, and 15-*β*OH. Above all, the structure of **5** was established and named as usambariphane D.

#### 2.1.6. Usambariphane E (**6**)

Compound **6** was obtained as a colorless crystal. The molecular formula was identified as C_41_H_52_O_16_ by HR-ESIMS *m/z* 801.3356 [M + H]^+^ (calcd. for C_41_H_53_O_16_ 801.3328). The 1D (Table 1 and Table 2) and 2D NMR data of **6** were almost identical with those of **5**, except for the ester group at C-7. In **6**, an O*i*Bu [*δ*_H_ 2.63, 1.26 (3H), 1.22 (3H), *δ*_C_ 175.2, 34.5, 19.0, 18.1] was presented at C-7 as confirmed by the HMBC correlation from H-7 (*δ*_H_ 6.39) to *δ*_C_ 175.2. The NOESY correlations of **6** revealed the same relative configuration as that of **5**. The structure of **6** was established and named as usambariphane E.

#### 2.1.7. Usambariphane F (**7**)

Compound **7** was obtained as a colorless crystal. The molecular formula was identified as C_39_H_52_O_15_ by HR-ESIMS *m/z* 761.3383 [M + H]^+^ (calcd. for C_39_H_53_O_15_ 761.3379). The 1D (Table 3) and 2D NMR spectra of **7** revealed four OAc [*δ*_H_ 2.24 (3H), *δ*_C_ 170.1, 21.0; *δ*_H_ 2.12 (3H), *δ*_C_ 170.9, 22.5; *δ*_H_ 2.06 (3H), *δ*_C_ 172.1, 20.9; *δ*_H_ 1.70 (3H), *δ*_C_ 172.2, 20.4], one O*i*Bu [*δ*_H_ 2.55, 1.19 (3H), 1.14 (3H), *δ*_C_ 175.1, 34.0 19.6, 18.4], one OBz [*δ*_H_ 8.00 (2H), 7.56, 7.42 (2H), *δ*_C_ 165.4, 133.4, 130.1, 129.7 (2C), 128.8 (2C)], four methyls [*δ*_H_ 1.55 (3H), *δ*_C_ 17.1, CH_3_-16; *δ*_H_ 1.03 (3H), *δ*_C_ 27.6, CH_3_-18; *δ*_H_ 1.40 (3H), δ_C_ 23.4, CH_3_-19; *δ*_H_ 1.06 (3H), δ_C_ 23.9, CH_3_-20], a *trans*-disubstituted C=C (*δ*_H_ 5.93, *δ*_C_ 134.0, CH-11; *δ*_H_ 5.76, *δ*_C_ 130.9, CH-12), and an exocyclic methylene (*δ*_H_ 5.26, 5.10, *δ*_C_ 110.4, CH_2_-17). Further, the skeleton of **7** was established as a jatrophane type diterpenoid with two double bonds at *∆*_6,17_ and *∆*_11,12_ based on the series ^1^H–^1^H COSY correlations of H-3/H-4/H-5 and H-11/H-12/H-13/H-14 and H_3_-20, as well as the HMBC correlations from H-1 to C-2 and C-16, H-3 to C-1, C-2, C-4 and C-15, and H_3_-16 to C-1, C-2 and C-3, H-5 to C-3, C-4, C-6, C-15, C-17, H-7 to C-6 and C-9, H-8 to C-6 and C-10, H-9 to C-8 and C-11, H-11 to C-10 and C-13, H-12 to C-10, H-14 to C-1, C-4, C-12, C-13, and C-15, H_2_-17 to C-5, C-6, and C-7, H_3_-18 and H_3_-19 to C-9, C-10 and C-11, H_3_-20 to C-12, C-13, and C-14. The presence of the 3-OH group was deduced by the ^1^H–^1^H COSY cross-peak between H-3 and 3-OH, and the HMBC correlations from 3-OH to C-2, C-3, and C-4. Another OH group was located at C-8 by the COSY cross-peak between H-8 and 8-OH, and the HMBC correlations from 8-OH to C-7 and C-8. The third OH group was connected to C-15 by the confirmation of the HMBC correlations from 15-OH to C-1, C-4, and C-15. The HMBC correlations of H-1/*δ*_C_ 170.1 (OAc), H-5/*δ*_C_ 165.4 (OBz), H-7/*δ*_C_ 175.1 (O*i*Bu), H-9/*δ*_C_ 172.1 (OAc), and H-14/*δ*_C_ 172.2 (OAc), demonstrated the locations of the acyl groups, and of necessity, the last OAc was located at C-2. The relative configuration of **7** was deduced by the NOESY spectrum. The H-4 and 15-OH in **7** can be assigned as *α*- and *β*-oriented, respectively, according to the comparison of the NMR data with those of known jatrophane-type diterpenoids [38,40]. The NOESY cross-peaks of H-1/H-4, H-3/H-4, and H-4/H-7 indicated the *α*-orientation of H-1, H-3, and H-7; meanwhile, the NOESY cross-peaks of H-5/15-OH, H-5/H-8, H-8/H_3_-19, H-9/H_3_-19, H-14/15-OH and H-14/H_3_-20 indicated the *β*-orientation of H-5, H-8, H-9, H-14, H_3_-19, and H_3_-20. Above all, the structure of **7** was established and named as usambariphane F.

#### 2.1.8. Usambariphane G (**8**)

Compound **8** was obtained as a colorless crystal. The molecular formula was identified as C_41_H_49_O_13_N by HR-ESIMS *m/z* 764.3230 [M + H]^+^ (calcd. for C_41_H_50_O_13_N 764.3277), indicating 18 degrees of molecular unsaturation. The 1D (Table 3) and 2D NMR spectra of **8** revealed two OAc [*δ*_H_ 2.07 (3H), *δ*_C_ 169.7, 20.7; *δ*_H_ 2.00 (3H), *δ*_C_ 169.9, 20.8], an O*i*Bu [*δ*_H_ 2.60, 1.23 (3H), 1.11 (3H), *δ*_C_ 175.8, 34.0 19.7, 18.4], an OBz [*δ*_H_ 8.06 (2H), 7.56, 7.44 (2H), *δ*_C_ 164.7, 133.4, 131.1, 130.0 (2C), 128.7 (2C)], a nicotinate group [*δ*_H_ 9.41, 8.79, 8.52, 7.39, *δ*_C_ 164.9, 153.4, 151.5, 137.6, 127.5, 123.2; ONic], four methyls [(*δ*_H_ 1.89 (3H), *δ*_C_ 20.9, CH_3_-16; *δ*_H_ 0.91 (3H), *δ*_C_ 26.5, CH_3_-18; *δ*_H_ 1.36 (3H), *δ*_C_ 23.2, CH_3_-19; *δ*_H_ 1.24 (3H), *δ*_C_ 19.6, CH_3_-20)], a *trans*-disubstituted C=C (*δ*_H_ 5.87, *δ*_C_ 137.9, CH-11; *δ*_H_ 5.57, *δ*_C_ 129.6, CH-12), ketone unit (*δ*_C_ 211.2, C-14), and an exocyclic methylene (*δ*_H_ 5.41, 5.16, *δ*_C_ 111.6, CH_2_-17). The skeleton of **8** was established as a jatrophane-type diterpenoid with two double bonds at *∆*_6,17_ and *∆*_11,12_, and the ketone unit at C-14 based on the series ^1^H–^1^H COSY correlations of H-3 (*δ*_H_ 4.76)/H-4 (*δ*_H_ 3.32)/H-5 (*δ*_H_ 5.67) and H-11/H-12/H-13 (*δ*_H_ 3.75)/H_3_-20; and the HMBC correlations from H_2_-1 (*δ*_H_ 2.95 and 2.27) to C-4 (*δ*_C_ 47.9), C-14, and C-15 (*δ*_C_ 89.0), H-3 to C-1(*δ*_C_ 51.4), C-2 (*δ*_C_ 92.0) and C-15, H-4 to C-15, H_3_-16 to C-1, C-2 and C-3 (*δ*_C_ 79.1), H-5 to C-3, C-6 (*δ*_C_ 144.9), and C-7 (*δ*_C_ 68.5), H-8 (*δ*_H_ 5.18) to C-6, C-7, C-9 (*δ*_C_ 80.6), and C-10 (*δ*_C_ 41.1), H-9 (*δ*_H_ 4.96) to C-10 and C-11, H-11 to C-9, C-10, C-13 (*δ*_C_ 44.4), C-18, and C-19, H-12 to C-10, H-13 to C-11, C-12, and C-14, H_2_-17 to C-5, C-6, and C-7, H_3_-18 and H_3_-19 to C-9 and C-10, H_3_-20 to C-12, C-13, and C-14. An OH group was located at C-3 based on a ^1^H–^1^H COSY cross-peak between H-3 and 3-OH (*δ*_H_ 3.57), and the HMBC correlations from 3-OH to C-3 and C-4. Another OH group was connected to C-15 by the confirmation of the HMBC correlations from 15-OH (*δ*_H_ 4.34) to C-1, C-4, and C-15. Furthermore, the HMBC correlations of H-5/*δ*_C_ 164.7 (OBz), H-7/*δ*_C_ 175.8 (O*i*Bu), H-8/*δ*_C_ 169.9 (OAc), H-9/*δ*_C_ 169.7 (OAc), demonstrated the locations of these acyl groups, and thereby the ONic was located at C-2. According to the NOESY cross-peaks of H-1a/H-4, H-1a/H-13, H-3/H-4, H-4/H-7, H-4/H-13, H-5/H-8, H-5/15-OH, H-8/H_3_-19, H-9/H_3_-19, H_3_-19/H_3_-20, 3-OH/15-OH, 3-OH/H_3_-16, and comparison of the NMR data with those of (2*R*,3*R*,4*R*,5*R*,7*S*,8*S*,9*S*,11*E*, 13*S*,15*R*)-2,3,5,7,8,9,15-heptahydroxyjatropha-6(17),11-diene-14-one-2,3,8,9-tetraacetate-5-benzoate-7-(2-methylpropionate) [41] indicated the relative configuration of **8** as depicted on Figure 2. The structure of **8** was established and named as usambariphane G.

#### 2.1.9. Isoterracinolides C (**9**)

Compound **9** was obtained as a white amorphous powder with a molecular formula of C_39_H_50_O_16_ determined based on HR-ESIMS *m/z* 775.3172 [M + H]^+^ (calcd. for C_39_H_51_O_16_ 775.3172). The 1D (Table 3) and 2D NMR spectra of **9** revealed five OAc [*δ*_H_ 2.36 (3H), *δ*_C_ 172.0, 20.7; *δ*_H_ 2.19 (3H), *δ*_C_ 169.6, 22.9; *δ*_H_ 2.16 (3H), *δ*_C_ 170.4, 21.4; *δ*_H_ 2.15 (3H), *δ*_C_ 171.2, 21.7; *δ*_H_ 2.14 (3H), *δ*_C_ 171.0, 20.9], one OBz [*δ*_H_ 8.07 (2H), 7.57, 7.46 (2H), *δ*_C_ 168.3, 133.9, 130.1 (2C), 128.8 (2C), 128.6], four methyls [*δ*_H_ 1.75 (3H), *δ*_C_ 19.8, CH_3_-16; *δ*_H_ 0.98 (3H), *δ*_C_ 26.4, CH_3_-18; *δ*_H_ 1.04 (3H), *δ*_C_ 20.8, CH_3_-19; *δ*_H_ 1.11 (3H), *δ*_C_ 22.4, CH_3_-20], and a *trans*-disubstituted C=C (*δ*_H_ 5.50, *δ*_C_ 134.7, CH-11; *δ*_H_ 5.79, *δ*_C_ 134.1, CH-12), and a lactone carbonyl carbon (*δ*_C_ 173.5, C-22). Further, comparing the NMR data of **9** with those of isoterracinolide A (**10**) [36] indicated that the structure of **9** is very similarly to **10**, except for the O*i*Bu which was replaced in **10** by an OAc. The HMBC correlation from H-7 to *δ*_C_ 171.0 suggested that the OAc was located at C-7 in **9**. Compound **9** was thus established and named as isoterracinolides C.

#### 2.1.10. 4β-Crotignoid K (**14**)

Compound **14** was obtained as a white amorphous powder. The molecular formula was determined as C_29_H_34_O_7_ by HR-ESIMS *m/z* 495.2385 [M + H]^+^ (calcd. for C_29_H_35_O_7_ 495.2377). The 1D (Table 4 and Table 5) and 2D NMR of **14** revealed one OAc [*δ*_H_ 2.14 (3H), *δ*_C_ 173.9, 21.3], one OBz [*δ*_H_ 8.02 (2H), 7.59, 7.47 (2H), *δ*_C_ 166.4, 133.4, 130.1, 129.9 (2C), 128.7 (2C)], four methyls [*δ*_H_ 1.21 (3H), *δ*_C_ 23.9, CH_3_-16; *δ*_H_ 1.33 (3H), *δ*_C_ 17.1, CH_3_-17; *δ*_H_ 0.98 (3H), *δ*_C_ 15.3, CH_3_-18; *δ*_H_ 1.73 (3H), *δ*_C_ 10.3, CH_3_-19], an oxygenated methylene [*δ*_H_ 4.05 (2H), *δ*_C_ 67.6, CH_2_-20], an oxygenated methine (*δ*_H_ 5.68, *δ*_C_ 77.8, CH-12), two unsaturated methines (*δ*_H_ 7.57, *δ*_C_ 159.7, CH-1; *δ*_H_ 5.56, *δ*_C_ 126.6, CH-7), and a ketone unit (*δ*_C_ 208.7, C-3). The interpretation of HMBC correlations suggested the skeleton of **14** was a tigliane-type diterpenoid [7] with an *α*-methyl-*α*,*β*-unsaturated cyclopentanone ring fused between C-4 and C-10, an OH (*δ*_H_ 5.62) connected to C-9, the OBz connected to C-12, the OAc connected to C-13 and a hydroxymethyl linked to C-6. Moreover, according to the NOESY correlations of H-4/H-8/H-11/H_3_-17 and H-12/H-14/9-OH/H_3_-18, as well as comparing the 1D NMR data of **14** with those of crotignoid K (**13**) [42] and 4-deoxyphorbol 12, 13-*bis*(isobutyrate) [43]. The structure of **14** was established as a 4*β* proton against the 4*α* proton of crotignoid K, thus named as 4*β*-crotignoid K.

#### 2.1.11. Euphodendriane B (**15**)

Compound **15** was obtained as a white amorphous powder. The molecular formula was determined as C_29_H_34_O_7_ by HR-ESIMS *m/z* 495.2396 [M + H]^+^ (calcd. for C_29_H_35_O_7_ 495.2377). The 1D (Table 4 and Table 5) and 2D NMR of **15** revealed one OAc [*δ*_H_ 2.11 (3H), *δ*_C_ 174.1, 21.2], one OBz [*δ*_H_ 8.06 (2H), 7.61, 7.49 (2H), *δ*_C_ 166.4, 133.4, 130.1, 129.9 (2C), 128.7 (2C)], five methyls [*δ*_H_ 1.20 (3H), *δ*_C_ 24.3, CH_3_-16; *δ*_H_ 1.33 (3H), *δ*_C_ 16.7, CH_3_-17; *δ*_H_ 1.16 (3H), *δ*_C_ 11.9, CH_3_-18; *δ*_H_ 1.83 (3H), *δ*_C_ 10.6, CH_3_-19; *δ*_H_ 1.90 (3H), *δ*_C_ 27.2, CH_3_-20], two oxygenated methines (*δ*_H_ 4.46, *δ*_C_ 71.1, CH-5; *δ*_H_ 5.73, *δ*_C_ 75.7, CH-12), two unsaturated methines (*δ*_H_ 7.06, *δ*_C_ 154.6, CH-1; *δ*_H_ 4.88 *δ*_C_ 125.5, CH-7), and a ketone unit (*δ*_C_ 207.5, C-3). The interpretation of HMBC correlations demonstrated that **15** was a tigliane-type diterpenoid with an *α*-methyl-*α*,*β*-unsaturated cyclopentanone ring fused between C-4 and C-10, with two OH (*δ*_H_ 5.92 and 5.95) connected to C-5 and C-9 respectively, one OBz connected to C-12, and one OAc connected to C-13 and 20-methyl group. The NMR data of **15** was highly close to those of euphodendriane A [35], except for the substitution at C-13 where an O*i*Bu in euphodendriane A was replaced in **15** by the OAc. The relative configuration of **15** was deduced by inspection of the NOESY spectrum, showing the same orientations to euphodendriane A [35]. Thus, the structure of **15** was established and named as euphodendriane B.

#### 2.1.12. 16-Nor-abieta-8,11,13-trien-3,7,15-trione (**16**)

Compound **16** was obtained as a colorless crystal with a molecular formula of C_19_H_22_O_3_ identified by HR-ESIMS *m/z* 299.1648 [M + H]^+^ (calcd. for C_19_H_22_O_3_ 299.1642). The 1D (Table 4 and Table 5) and 2D NMR data of **16** revealed an acetyl moiety (*δ*_C_ 197.3, C-15; *δ*_H_ 2.64 (3H), *δ*_C_ 26.9, CH_3_-17), three methyls [*δ*_H_ 1.17 (3H), *δ*_C_ 25.2, CH_3_-18; *δ*_H_ 1.23 (3H), *δ*_C_ 21.7, CH_3_-19; *δ*_H_ 1.48 (3H), *δ*_C_ 22.8, CH_3_-20], three methylenes [*δ*_H_ 2.68, 2.05, *δ*_C_ 36.8, CH_2_-1; *δ*_H_ 2.91, 2.59, *δ*_C_ 34.6, CH_2_-2; *δ*_H_ 2.83, 2.75, *δ*_C_ 36.5, CH_2_-6], a methine (*δ*_H_ 2.36, *δ*_C_ 49.2, CH-5), a set of trisubstituted aromatic ring (*δ*_C_ 130.8, C-8; *δ*_C_ 158.2, C-9; *δ*_H_ 7.49 (d, *J* = 8.5), *δ*_C_ 125.2, CH-11; *δ*_H_ 8.17 (dd, *J* = 8.5, 2.5), *δ*_C_ 133.4, CH-12; *δ*_C_ 135.9, C-13; *δ*_H_ 8.57 (d, *J* = 2.5), *δ*_C_ 128.3, CH-14], two ketone units (*δ*_C_ 214.0, C-3; *δ*_C_ 197.4, C-7), and two quaternary carbons (*δ*_C_ 47.6, C-4; *δ*_C_ 38.3, C-10). The HMBC correlations of **16** from H_2_-1 to C-3, C-9, and C-20, H_2_-2 to C-3 and C-4, H-5 to C-1, C-4, C-9, and C-10, H_2_-6 to C-7, C-8, and C-10, H-12 to C-15, H-14 to C-7 and C-15, H_3_-17 to C-13, H_3_-18 and H_3_-19 to C-3, C-4, and C-5, and H_3_-20 to C-5 and C-10, suggested that **16** was an abietane-type diterpenoid [7] and was structurally similar to a known compound abieta-8,11,13-triene-3,7-dione [44,45], except for the substitution of the acetyl moiety at C-15–C-17. The relative configuration of **16** was the same as the typical abieta-8,11,13-triene diterpenoids [45] based on the NOESY correlations of H-5/H_3_-18 and H_3_-19/H_3_-20 as well as the comparison of the NMR data of **16** with those of literature [44,45]. The structure of **16** was identified as 16-nor-abieta-8,11,13-trien-3,7,15-trione.

#### 2.1.13. 16-Nor-3β-hydroxy-abieta-8,11,13-trien-7,15-dione (**17**)

Compound **17** was obtained as a colorless crystal with a molecular formula of C_19_H_24_O_3_ identified by HR-ESIMS *m/z* 301.1803 [M + H]^+^ (calcd. for C_19_H_25_O_3_ 301.1798). The inspection of 1D (Table 4 and Table 5) and 2D NMR data revealed that **17** was a 16-nor-abieta-8,11,13-triene diterpenoid [45]. The ^1^H and ^13^C NMR data of **17** was close to those of **16**, except for a hydroxy group that was situated at C-3 (*δ*_C_ 78.0) by the confirmation of the ^1^H–^1^H COSY cross-peaks between H_2_-1/H_2_-2/H-3 as well as the HMBC correlations from H_2_-1 to C-3 and H_3_-18 to C-3. The H-3 [*δ*_H_ 3.37 (dd, *J* = 11.5, 4.0)] was identified as *α*-oriented by the NOESY correlations of H-3/H-5/H_3_-18 and the comparison of the proton signals for **17** with those of the similar compound 3*β*-hydroxy-abieta-8,11,13-trien-7-one [46]. The structure of **17** was identified as 16-nor-3*β*-hydroxy-abieta-8,11,13-trien-7,15-dione.

#### 2.1.14. ent-8β,14β-Epoxyabieta-3-one-11,13(15)-dien-16,12-olide (**19**)

Compound **19** was purified as a colorless gum. The molecular formula was calculated as C_20_H_24_O_4_ by HR-ESIMS *m/z* 329.1753 [M + H]^+^ (calcd. for C_20_H_25_O_4_ 329.1747). The 1D (Table 4 and Table 5) and 2D NMR data of **19** revealed four methyls [*δ*_H_ 2.09 (3H), *δ*_C_ 9.0, CH_3_-17; *δ*_H_ 1.17 (3H), *δ*_C_ 25.9, CH_3_-18; *δ*_H_ 1.09 (3H), *δ*_C_ 22.4, CH_3_-19; *δ*_H_ 0.95 (3H), *δ*_C_ 15.0, CH_3_-20], four methylenes [*δ*_H_ 2.05, 1.75, *δ*_C_ 38.1, CH_2_-1; *δ*_H_ 2.65, 2.37, *δ*_C_ 34.2, CH_2_-2; *δ*_H_ 1.79, 1.70, *δ*_C_ 21.7, CH_2_-6; *δ*_H_ 2.17, 1.68, *δ*_C_ 33.9, CH_2_-7], four methine (*δ*_H_ 1.67, *δ*_C_ 54.1, CH-5; *δ*_H_ 2.70, *δ*_C_ 50.1, CH-9; *δ*_H_ 5.44, *δ*_C_ 102.9, CH-11; *δ*_H_ 3.76, *δ*_C_ 54.5, CH-14), six quaternary carbons (*δ*_C_ 48.1, C-4; *δ*_C_ 61.0, C-8; *δ*_C_ 40.9, C-10; *δ*_C_ 148.1, C-12; *δ*_C_ 144.7, C-13; *δ*_C_ 126.2, C-15), and two carbonyl carbons (*δ*_C_ 215.0, C-3; *δ*_C_ 170.5, C-16). The structure of **19** was suggested an abietane-type diterpenoid with a ketone carbon at C-3, an epoxy ring fused at C-8 and C-14, a double bond at *∆*_11,12_, and an *α*-methyl-*α*,*β*-unsaturated *δ*-lactone ring formed as D ring according to the analysis of the COSY cross-peaks of H_2_-1/H_2_-2, H_2_-5/H_2_-6/H_2_-7, and H-9/H-11, as well as the HMBC correlations from H_2_-1 to C-3, C-5, and C-10, H_2_-2 to C-3 and C-10, H-5 to C-4, C-18, C-19, and C-20, H_2_-7 to C-5 and C-8, H-9 to C-1, C-5, C-8, C-12, C-14, and C-20, H-11 to C-8, C-9, C-12, and C-13, H-14 to C-7, C-8, C-12, and C-13, H_3_-17 to C-13, C-15, and C-16, H_3_-18 and H_3_-19 to C-3 and C-4, together with H_3_-20 to C-1, C-9, and C-10. Comparison of the ^13^C NMR data with those of the related compounds jolkinolide A [47] and gelomulide C [48] further evidenced the presence of an *ent*-abietane skeleton with 8*β*,14*β*-epoxide in **19**. Thus, **19** was established as ent-8β,14β-epoxyabieta-3-one-11,13(15)-dien-16,12-olide.

#### 2.1.15. ent-8β,14β-Epoxyabieta-3α-hydroxy-13(15)-en-16,12-olide (**20**)

Compound **20** was purified as a colorless gum. The molecular formula was calculated as C_20_H_28_O_4_ by HR-ESIMS *m/z* 333.2067 [M + H]^+^ (calcd. for C_20_H_29_O_4_ 333.2060). The ^1^H and ^13^C NMR data (Table 4 and Table 5) of **20** were close to those of **19** suggesting that the skeleton of **20** was an *ent*-abietane with 8*β*,14*β*-epoxide. Instead of the ketone carbon at C-3 in **19**, a hydroxyl group was connected to C-3 (*δ*_C_ 78.6) based on the inspection of the ^1^H–^1^H COSY cross-peaks between H_2_-1/H_2_-2/H-3 together with the HMBC correlations from H-3 to C-1, C-4, C-18, and C-19. Saturated methylene (*δ*_H_ 2.27, 1.41, *δ*_C_ 24.0) and an oxygenated methine (*δ*_H_ 4.99, *δ*_C_ 75.6) were assigned to be at C-11 and C-12, respectively, according to the analysis of the ^1^H–^1^H COSY cross-peaks between H-9/H_2_-11/H-12 together with the HMBC correlations from H_2_-11 to C-8, C-10, and C-13, as well as H-12 to C-13 and C-15. The structure was found to be highly similar to the NMR features of gelomulide A [48], except for instead of the hydroxyl group at C-3 in **20**. The configuration of 3-OH was deduced to be *α*-oriented as the proton signal of H-3 at 3.30 (dd, *J* = 12.0, 4.0 Hz) [49,50]. It was also supported by the NOESY correlations of H-3/H-5/H-9/H_3_-18. The structure of **20** was established as ent-8β,14β-epoxyabieta-3α-hydroxy-13(15)-en-16,12-olide.

The known compounds were identified as isoterracinolide A (**10**) [36], isoterracinolide B (**11**) [36], 12-*O*-benzoyl-13-acetoxy-4,20-dideoxyphorbol-4-ene (**12**) [51,52], crotignoid K (**13**) [42], 16-*nor*-abieta-8,11,13-trien-3,15-dione (**18**) [53], helioscopinolide E (**21**) [54], helioscopinolide C (**22**) [54], helioscopinolide A (**23**) [54], *ent*-kauran-16*β*-ol-3-one (**24**) [55], cleomiscosin A (**25**) [56], (+)-syringaresinol (**26**) [57], dimeric coniferyl acetate (**27**) [58], vanillin (**28**) [59], 4-hydroxybenzaldehyde (**29**) [60], coniferol alcohol (**30**) [61], and indole-3-carboxaldehyde (**31**) [62] by comparison of the NMR data with those of the literature.

### 2.2. HIV-1 Latency Reversal Activity of Isolated Compounds in Vitro

**Figure 4:**
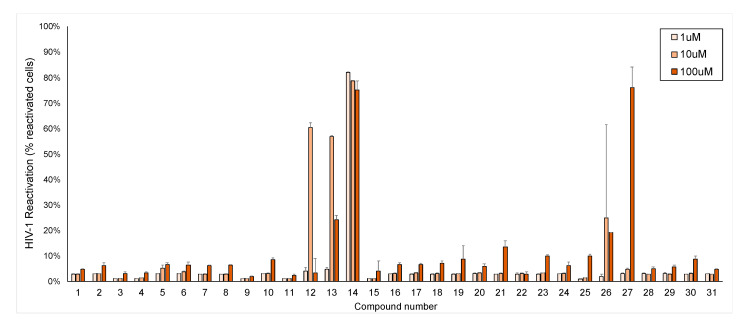
HIV-1 latency reversal activity of compounds **1**–**31** on J-Lat 10.6 cells in vitro.

**Figure 5:**
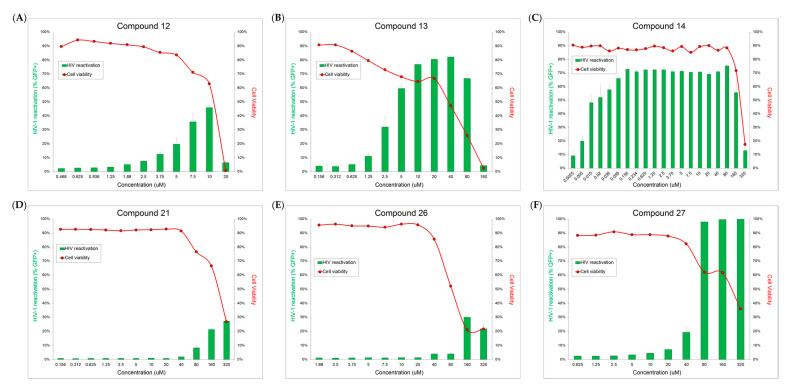
HIV-1 latency reversal activity dose-response (green bar) and cytotoxicity (red curve) of active compounds **12**–**14**, **21**, **26**, and **27** at additional concentrations. (**A**) dose-response experiments of **12** in a series concentration from 0.468 to 20 μM; (**B**) dose-response experiments of **13** in a series concentration from 0.158 to 160 μM; (**C**) dose-response experiments of **14** in a series concentration from 0.0025 to 320 μM; (**D**) dose-response experiments of **21** in a series concentration from 0.156 to 320 μM; (**E**) dose-response experiments of **26** in a series concentration from 1.88 to 320 μM; (**F**) dose-response experiments of **27** in a series concentration from 0.625 to 320 μM.

Jurkat cells with a full-length integrated HIV-1 provirus that have been modified to contain a GFP coding region in place of the env gene (J-lat 10.6 cells) were used for HIV-1 anti-latency activity, cytotoxicity, and cellular activation testing. All 31 compounds were tested at 1, 10, and 100 μM. Through the GFP expression of J-lat 10.6 cells, it was determined that compounds **12**, **13**, **14**, **21**, **26**, and **27** showed HIV-1 latency reversal activity (Figure 4). These compounds were further tested at additional concentrations to determine dose response and toxicity curves (Figure 5A–F). Cell viability for all isolated compounds is presented in Figure S106 in the supplementary material.

**Figure 6:**
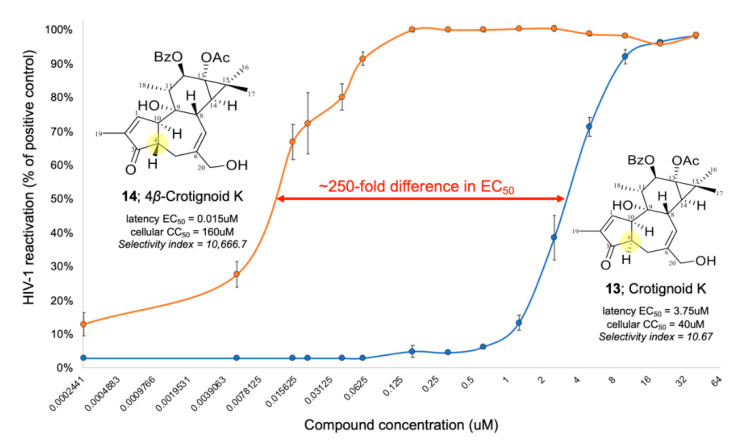
HIV-1 reactivation dose-response for stereoisomers crotignoid K (**13**) and 4*β*-crotignoid K (**14**).

4*β*-Crotignoid K (**14**) showed high reactivation levels into nM concentrations, ~250-fold less than crotignoid K (**13**), which is a stereoisomer of **14**, differing only in the configuration on C-4 (Figure 6). The striking difference between these compounds isolated from *E. usambarica* demonstrated a structure-activity relationship (SAR) of an important cellular trigger to induce HIV-1 proviral transcription. A similar SAR has recently been described between protein kinase C (PKC) agonists, 4-deoxyphorbol (4*β*-dPEA), phorbol myristate acetate (PMA), and prostratin [63].

**Figure 7:**
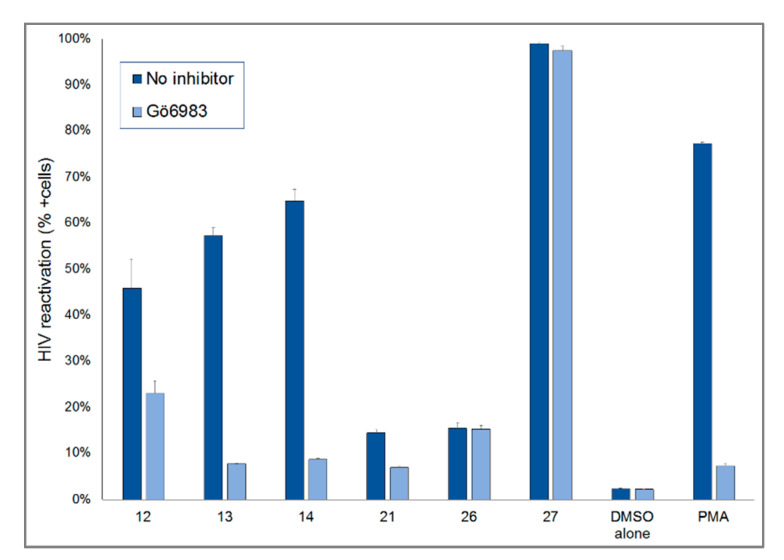
Effect of PKC inhibition on HIV-1 reactivation of compounds **13**–**14**, **21**, **26**, and **27**.

*Euphorbia* species have been shown to be enriched for compounds capable of protein kinase C (PKC) activation in human cells [5,9,11,14,15]. In order to determine whether our active compounds were acting through PKC, we evaluated the latency reversal activity of each of these compounds in the presence and absence of a pan-PKC inhibitor, Gö6983 (Figure 7). Compounds **12**, **13**, **14**, and **21** all showed reduced efficacy, indicating likely activation of PKC as their primary mechanism of action. In contrast, compounds **26** and **27** did not show a significant reduction in their activity when PKC was inhibited. In addition, these compounds did not increase CD69 expression (a hallmark of PKC activation), further suggesting an alternative (non-PKC) mechanism of latency reversal.

## 3. Discussion

In this study, 15 new diterpenoids, together with 16 known compounds, were isolated from the dichloromethane phase of methanolic extract of the medicinal plant *E. usambarica*. Compound **1** exhibited a 6/6/3-fused ring system with an *α*-methyl-*α*,*β*-unsaturated cyclopentanone moiety to construct a rare diterpenoid lactone, this skeleton was the second time discovered from nature [34]. The other compounds could be summarized in 4 types of diterpenoids, including jatrophanes (**2**–**11**), tiglianes (**12**–**15**), abietanes (**16**–**23**), and kaurane (**24**), alone with coumarinolignoid (**25**), lignan (**26**), coniferyl acetate (**27**), and benzenoids (**28**–**31**). Especially, usambariphanes A (**2**) and B (**3**) displayed an unusual lactone ring constructed between C-17 and C-2 in the jatrophane structure, which is different from such lactone ring commonly constructed between C-17 and C-3 or between C-17 and C-5.

Furthermore, compounds **12**–**14**, **21**, **26**, and **27** showed significant HIV-1 latency reversal activity demonstrated by the GFP expression of J-lat 10.6 cells. 4*β*-Crotignoid K (**14**) showed the reactivation of HIV-1 latency at a very low concentration of EC_50_ about 0.015 μM and a higher CC_50_ concentration than 160 μM. The stereoisomer, crotignoid K (**13**), showed the EC_50_ and CC_50_ concentrations about 3.75 and 40 μM, respectively, indicating that 4*β*-crotignoid K (**14**) was provided with higher safety and efficacy. There is a 250-fold difference in EC_50_ and ~1000-fold difference in selectivity index (CC_50_/EC_50_) between these compounds. The structural difference between **13** and **14** is only in the relative configuration on C-4. However, they demonstrated dramatically different biological activity, indicating that the configuration on C-4 of tigliane-type diterpenoids is critical to HIV-1 latency reversal activity and likely reflects improved PKC activation. The primary mechanism of the active compounds **12**–**14** and **21** for the HIV-1 latency reversal activity was activation of PKC.

Currently, LRAs are still under investigation and have not been approved by the US Food and Drug Administration (US-FDA). Therefore, the intensive study of LRAs is an important topic, especially to discover new candidates from natural sources. For example, a known LRA, ingenol, is isolated originally from *Euphorbia peplus* [64] and is a US-FDA-approved topical treatment for actinic keratosis (AK) [65], showed a significant effect in the reactivation of HIV-1 latency through the PKC pathway [65]. Both compound **14** and ingenol mebutate are *Euphorbia* diterpenoids and the potent PKC agonists. In addition, compound **14** presented the lower cytotoxicity, indicating **14** is a promising candidate for the development of an LRA.

In contrast, (+)-syringaresinol (**26**) and dimeric coniferyl acetate (**27**) did not increase CD69 expression, further suggesting a non-PKC mechanism of latency reversal that merits further exploration.

## 4. Materials and Methods

### 4.1. General Experimental Procedures

Optical rotation was performed on a Perkin-Elmer 341 polarimeter. 1D and 2D NMR spectra were recorded on a Bruker Avance DRX 500 spectrometer at 500 MHz (^1^H) and 125 MHz (^13^C). Chemical shifts were reported in parts per million (*δ*), and the coupling constants (*J*) were expressed in Hertz. The residual peaks of the deuterated solvents were taken as reference points. The NMR data were acquired and processed with MestReNova v12.0.0−20080 software. High-resolution MS spectra were acquired on an FTHRMS-Orbitrap (Thermo-Finnigan) mass spectrometer equipped with an ESI ion source in positive ionization mode. HPLC analyses were performed with a Shimadzu LC-10AS pump interface equipped with a Shimadzu SPD-10A UV–VIS detector (Shimadzu Inc., Kyoto, Japan) using Kinetex C18 column (5 μm, 100 Å, 250 × 4.6 mm), Kinetex Biphenyl column (5 μm, 100 Å, 250 × 4.6 mm), Kinetex XB-C18 column (5 μm, 100 Å, 250 × 10 mm), and/or Luna^®^ Phenyl-Hexyl column (5 μm, 250 × 10 mm) (Phenomenex Inc., Torrance, CA, USA) using a mixture of acetonitrile–H_2_O or mixture of methanol–H_2_O as mobile phase. Rotational planar chromatography (RPC) was performed on self-coated silica plates (Kieselgel 60 GF_254_, 15 µm, Merck, Germany) using a Chromatotron apparatus (Harrison Research, Palo Alto, CA, USA). Silica gel (Kieselgel 60, 63–200 μm, Merck, Darmstadt, Germany), polyamide (MP Polyamide, 50−160 μm, MP Biomedicals, Irvine, CA, USA), and Sephadex LH-20 gel (Pharmacia Fine Chemicals AB, Uppsala, Sweden) were used for column chromatography (CC). Thin-layer chromatography (TLC) was carried out using silica gel (Kieselgel 60 F_254_, Merck) and RP-C18 (F_254s_, Merck) pre-coated plates, and the preparative TLC (pre-TLC) was performed on glass sheet silica gel pre-coated plates (20 × 20 cm, Kieselgel 60 F_254_, Merck). The compounds were detected with a developer (20% H_2_SO_4_ (*v*/*v*) with 5% vanillin (*w*/*v*) in ethanol) followed by heating (120 °C).

### 4.2. Plant Material

*Euphorbia usambarica* Pax. (Euphorbiaceae) was collected in Taita Taveta county, Kenya in 2019. Identification was performed by Peter Waweru Mwangi (Department of Medical Physiology, School of Medicine, University of Nairobi, Nairobi, Kenya). A voucher specimen (No. EU-001) has been deposited in the Herbarium of the Department of Pharmacognosy, University of Szeged, Szeged, Hungary.

### 4.3. Extraction and Isolation

The dried stem and root part (2.7 kg) were chopped and extracted with methanol (MeOH, 15 L) at room temperature. After removing the solvent, the crude methanolic extract (EU, 220.0 g) was dissolved in 50% MeOH_aq_ and subjected to liquid–liquid partition to afford *n*-hexane (EU-H), dichloromethane (CH_2_Cl_2_, EU-C), ethyl acetate (EtOAc, EU-E), and water-soluble residue (EU-W) phases. The EU-C (25.7 g) was subjected to polyamide CC with MeOH–H_2_O mixture solvent system (40%, 60%, 80%, and 100% MeOH_aq_; EU-C-P1–P4). The EU-C-P1 (8.7 g) was further subjected to normal phase CC (silica gel, 63–200 μm) with a gradient solvent system of *n*-hexane–EtOAc–MeOH mixtures (from 40:5:1 to 0:8:1) to obtain ten subfractions (EU-C-P1-1–10) based on the TLC monitoring. EU-C-P1-2 (29.7 mg) was subjected to Sephadex LH-20 CC with the eluent of CH_2_Cl_2_–EtOAc–MeOH (1:1:6) to yield 6 subfractions (EU-C-P1-2/1–6), and EU-C-P1-2/2 was further separated by RP-HPLC on Kinetex XB-C18 column with an isocratic solvent system of MeCN–H_2_O (60:40, 2.0 mL/min) to yield compound **18** (1.1 mg). EU-C-P1-3 (895.5 mg) was separated by Sephadex LH-20 CC using CH_2_Cl_2_–EtOAc–MeOH (1:1:6) as eluent to obtain 5 subfractions (EU-C-P1-3/1–5). EU-C-P1-3/2 (245.5 mg) was further subjected to RPC (thickness 2 mm) using a gradient system of CH_2_Cl_2_–MeOH (from 100:0 to 15:1) to obtain 5 subfractions (EU-C-P1-3/2/1–5). Compound **21** (63.5 mg) was purified by recrystallization (MeOH) from EU-C-P1-3/2/1 (133.8 mg), and the residue of this fraction was further purified by RP-HPLC on Kinetex XB-C18 column with an isocratic system of MeCN–H_2_O (53:47, 2.0 mL/min) to yield compounds **16** (4.0 mg) and **19** (3.5 mg). EU-C-P1-3/2/2 (38.6 mg) was subjected to prep-TLC using CH_2_Cl_2_–MeOH (60:1) as the eluent to obtain 5 subfractions (EU-C-P1-3/2/2/1–5), then the second and third subfractions were purified by RP-HPLC on Kinetex XB-C18 column with an isocratic system of MeCN–H_2_O (65:35, 2.0 mL/min) to yield compounds **10** (4.7 mg) and **15** (1.0 mg), respectively. EU-C-P1-3/3 (178.5 mg) was subjected to RPC (thickness 2 mm) using a gradient system of *n*-hexane–CH_2_Cl_2_–MeOH (from 5:1:0 to 20:1) to obtain 9 subfractions (EU-C-P1-3/3/1–9), then the second subfraction (23.0 mg) was purified by RP-HPLC on Kinetex Biphenyl column with an isocratic system of MeOH–H_2_O (75:25, 1.0 mL/min) to yield compound **24** (2.7 mg). EU-C-P1-3/4 (39.3 mg) was separated by prep-TLC using CH_2_Cl_2_–MeOH (60:1) as eluent to yield compound **28** (11.0 mg). EU-C-P1-3/5 (10.2 mg) was purified by prep-TLC using CH_2_Cl_2_–MeOH (35:1) as eluent to yield compound **29** (1.8 mg). EU-C-P1-4 (1060.4 mg) was subjected to Sephadex LH-20 gel chromatography eluting with CH_2_Cl_2_–EtOAc–MeOH (1:1:6) to obtain 9 subfractions (EU-C-P1-4/1–9). EU-C-P1-4/2 (442.8 mg) was further separated by RPC (thickness 2 mm) using a gradient system of *n*-hexane–CH_2_Cl_2_–MeOH (from 1:1:0 to 10:1) to obtain 8 subfractions (EU-C-P1-4/2/1–9). EU-C-P1-4/2/2 (14.1 mg) was further purified by RP-HPLC on Kinetex XB-C18 column with an isocratic solvent system of MeCN–H_2_O (50:50, 2.0 mL/min) to yield compound **27** (4.6 mg). EU-C-P1-4/2/4 (68.6 mg) was further purified by RP-HPLC on Kinetex XB-C18 column with isocratic solvent system of MeCN–H_2_O (55:45, 2.0 mL/min) to yield compounds **1** (1.1 mg), **2** (4.0 mg), **3** (1.5 mg), **4** (2.0 mg), **5** (5.2 mg), **6** (2.5 mg), **7** (3.2 mg), **8** (1.4 mg), **9** (1.7 mg), **11** (3.7 mg), and **22** (2.2 mg). EU-C-P1-4/2/6 (60.4 mg) was purified by RP-HPLC on Kinetex XB-C18 column with an isocratic solvent system of MeCN–H_2_O (50:50, 2.0 mL/min) to yield compounds **12** (2.3 mg) and **13** (4.2 mg). EU-C-P1-4/2/7 (40.9 mg) was purified by RP-HPLC on Luna^®^ Phenyl-Hexyl column with an isocratic system of MeCN–H_2_O (51:49, 2.0 mL/min) to yield compound **14** (1.4 mg). EU-C-P1-4/4 (218.3 mg) was further separated by RPC (thickness 2 mm) using a gradient system of CH_2_Cl_2_–MeOH (from 100:0 to 20:1) to obtain 6 subfractions (EU-C-P1-4/4/1–6). EU-C-P1-4/4/2 (52.4 mg) was further chromatographed by Sephadex LH-20 CC eluting with CH_2_Cl_2_–EtOAc–MeOH (1:1:6) to obtain 3 subfractions, then the second subfraction was purified by RP-HPLC on Kinetex XB-C18 column with an isocratic solvent system of MeCN–H_2_O (53:47, 2.0 mL/min) to yield compounds **17** (2.8 mg), **20** (2.7 mg), and **23** (14.2 mg). EU-C-P1-4/8 (11.6 mg) was purified by RP-HPLC on Kinetex XB-C18 column with an isocratic solvent system of MeCN–H_2_O (35:65, 2.0 mL/min) to yield compounds **30** (1.3 mg) and **31** (1.6 mg). EU-C-P1-7 (480.1 mg) was subjected to Sephadex LH-20 CC eluting with CH_2_Cl_2_–EtOAc–MeOH (1:1:6) to obtain 8 subfractions (EU-C-P1-7/1–8). EU-C-P1-7/6 was further purified by RP-HPLC on Kinetex XB-C18 column with an isocratic system of MeOH–H_2_O (48:52, 2.0 mL/min) to yield compound **26** (2.6 mg). Compound **25** (8.3 mg) was yielded by re-crystallization (MeOH) from EU-C-P1-7/7.

### 4.4. Physical Characteristic of New Compounds

*Euphordraculoate C (**1**)*: Colorless gum; ${\left[\alpha \right]}_{D}^{28}$ −63 (*c* 0.05, CHCl_3_); the ^1^H and ^13^C NMR spectroscopic data, see Table 1 and Table 2; HR-ESIMS *m/z* 493.2237 [M + H]^+^ (calcd. for C_29_H_33_O_7_ 493.2221), *m/z* 515.2046 [M + Na]^+^ (calcd. for C_29_H_32_O_7_Na 515.2040).

*Usambaricinophane A (**2**)*: White amorphous powder; ${\left[\alpha \right]}_{D}^{28}$ −17 (*c* 0.20, CHCl_3_); the ^1^H and ^13^C NMR spectroscopic data, see Table 1 and Table 2; HR-ESIMS *m/z* 789.3327 [M + H]^+^ (calcd. for C_40_H_53_O_16_ 789.3328), *m/z* 811.3169 [M + Na]^+^ (calcd. for C_40_H_52_O_16_Na 811.3148).

*Usambaricinophane B (**3**)*: White amorphous powder; ${\left[\alpha \right]}_{D}^{28}$ −7 (*c* 0.07, CHCl_3_); the ^1^H and ^13^C NMR spectroscopic data, see Table 1 and Table 2; HR-ESIMS *m/z* 803.3488 [M + H]^+^ (calcd. for C_41_H_55_O_16_ 803.3485), *m/z* 825.3333 [M + Na]^+^ (calcd. for C_41_H_54_O_16_Na 825.3304).

*Usambaricinophane C (**4**)*: colorless crystal; ${\left[\alpha \right]}_{D}^{28}$ +34 (*c* 0.10, CHCl_3_); the ^1^H and ^13^C NMR spectroscopic data, see Table 1 and Table 2; HR-ESIMS *m/z* 789.3346 [M + H]^+^ (calcd. for C_40_H_53_O_16_ 789.3328) *m/z* 811.3171 [M + Na]^+^ (calcd. for C_40_H_52_O_16_Na 811.3148).

*Usambaricinophane D (**5**)*: Colorless crystal; ${\left[\alpha \right]}_{D}^{28}$ +54 (*c* 0.30, CHCl_3_); the ^1^H and ^13^C NMR spectroscopic data, see Table 1 and Table 2; HR-ESIMS *m/z* 787.3193 [M + H]^+^ (calcd. for C_40_H_51_O_16_ 787.3172), *m/z* 809.3011 [M + Na]^+^ (calcd. for C_40_H_50_O_16_Na 809.2991).

*Usambaricinophane E (**6**)*: Colorless crystal; ${\left[\alpha \right]}_{D}^{28}$ +54 (*c* 0.15, CHCl_3_); the ^1^H and ^13^C NMR spectroscopic data, see Table 1 and Table 2; HR-ESIMS *m/z* 801.3356 [M + H]^+^ (calcd. for C_41_H_53_O_16_ 801.3328) *m/z* 823.3170 [M + Na]^+^ (calcd. for C_41_H_52_O_16_Na 823.3148).

*Usambaricinophane F (**7**)*: Colorless crystal; ${\left[\alpha \right]}_{D}^{28}$ +15 (*c* 0.20, CHCl_3_); the ^1^H and ^13^C NMR spectroscopic data, see Table 3; HR-ESIMS *m/z* 761.3383 [M + H]^+^ (calcd. for C_39_H_53_O_15_ 761.3379), *m/z* 783.3215 [M + Na]^+^ (calcd. for C_39_H_52_O_15_Na 783.3198).

*Usambaricinophane G (**8**)*: Colorless crystal; ${\left[\alpha \right]}_{D}^{28}$ +15 (*c* 0.08, CHCl_3_); the ^1^H and ^13^C NMR spectroscopic data, see Table 3; HR-ESIMS *m/z* 764.3230 [M + H]^+^ (calcd. for C_41_H_50_O_13_N 764.3277), *m/z* 786.3098 [M + Na]^+^ (calcd. for C_41_H_49_O_13_NNa 786.3096).

*Isoterracinolide C (**9**)*: White amorphous powder; ${\left[\alpha \right]}_{D}^{28}$ ‒2 (*c* 0.10, CHCl_3_); the ^1^H and ^13^C NMR spectroscopic data, see Table 3; HR-ESIMS *m/z* 775.31721 [M + H]^+^ (calcd. for C_39_H_51_O_16_ 775.3172), *m/z* 797.3011 [M + Na]^+^ (calcd. for C_39_H_50_O_16_Na 797.2991).

*4β-Crotignoid K (**14**)*: White amorphous powder; ${\left[\alpha \right]}_{D}^{28}$ +48 (*c* 0.05, CHCl_3_); the ^1^H and ^13^C NMR spectroscopic data, see Table 4 and Table 5; HR-ESIMS *m/z* 495.2385 [M + H]^+^ (calcd. for C_29_H_35_O_7_ 495.2377), *m/z* 517.2202 [M + Na]^+^ (calcd. for C_29_H_34_O_7_Na 517.2197).

*Euphodendriane B (**15**)*: White amorphous powder; ${\left[\alpha \right]}_{D}^{28}$ +12 (*c* 0.033, CHCl_3_); the ^1^H and ^13^C NMR spectroscopic data, see Table 4 and Table 5; HR-ESIMS *m/z* 495.2396 [M + H]^+^ (calcd. for C_29_H_35_O_7_ 495.2377), *m/z* 517.2209 [M + Na]^+^ (calcd. for C_29_H_34_O_7_Na 517.2197).

*16-Nor-abieta-8,11,13-trien-3,7,15-trione (**16**)*: Colorless crystal; ${\left[\alpha \right]}_{D}^{28}$ +15 (*c* 0.20, CHCl_3_); the ^1^H and ^13^C NMR spectroscopic data, see Table 4 and Table 5; HR-ESIMS *m/z* 299.1648 [M + H]^+^ (calcd. for C_19_H_22_O_3_ 299.1642), *m/z* 321.1466 [M + Na]^+^ (calcd. for C_19_H_22_O_3_Na 321.1461).

*16-Nor-3β-hydroxy-abieta-8,11,13-trien-7,15-dione (**17**)*: Colorless crystal; ${\left[\alpha \right]}_{D}^{28}$ −9 (*c* 0.20, CHCl_3_); the ^1^H and ^13^C NMR spectroscopic data, see Table 4 and Table 5; HR-ESIMS *m/z* 301.1803 [M + H]^+^ (calcd. for C_19_H_25_O_3_ 301.1798), *m/z* 323.1623 [M + Na]^+^ (calcd. for C_19_H_24_O_3_Na 323.1618).

*ent-8β,14β-Epoxyabieta-3-one-11,13(15)-dien-16,12-olide (**19**)*: Colorless gum; ${\left[\alpha \right]}_{D}^{28}$ +90 (*c* 0.20, CHCl_3_); the ^1^H and ^13^C NMR spectroscopic data, see Table 4 and Table 5; HR-ESIMS *m/z* 329.1753 [M + H]^+^ (calcd. for C_20_H_25_O_4_ 329.1747), *m/z* 351.1572 [M + Na]^+^ (calcd. for C_20_H_24_O_4_Na 351.1567).

*ent-8β,14β-Epoxyabieta-3α-hydroxy-13(15)-en-16,12-olide (**20**)*: Colorless gum; ${\left[\alpha \right]}_{D}^{28}$ +57 (*c* 0.20, CHCl_3_); the ^1^H and ^13^C NMR spectroscopic data, see Table 4 and Table 5; HR-ESIMS *m/z* 333.2067 [M + H]^+^ (calcd. for C_20_H_29_O_4_ 333.2060), *m/z* 355.1886 [M + Na]^+^ (calcd. for C_20_H_28_O_4_Na 355.1880).

### 4.5. Cell Isolation and Culture

The HIV-1-infected Jurkat T cell line (J-Lat 10.6) was obtained in January 2019 from the NIH/ATCC HIV-1 Reagent Program (www.hivreagentprogram.org) and cultured in RPMI-based media supplemented with 10% fetal calf serum, 1% penicillin, and 1% streptomycin.

### 4.6. Flow Cytometry

After in vitro culture, J-lat cells were washed with phosphate-buffered saline (1× PBS) prior to staining with 0.1 µL fixable viability dye Live/Dead Aqua (Cat L34957, www.thermofisher.com) per 10^5^ cells for 30 min at 4 °C. Simultaneously, J-lat cells were also stained with antibodies against CD69 conjugated to an APC fluorophore (APC anti-human CD69 antibody, biolegend.com). Cells were then washed and re-suspended in 1× PBS prior to flow cytometry acquisition evaluating for cellular viability, green fluorescent protein (GFP) expression, and CD69 expression. Flow cytometry was performed with a BD FACSCelesta or FACSCanoto flow cytometer with FACSDiva acquisition software (Becton Dickinson, Mountain View, CA) prior to analysis with FlowJo (TreeStar Inc., Ashland, OR, USA).

### 4.7. Compound Screening

Isolated compounds were resuspended in dimethyl sulfoxide (DMSO; Sigma-Aldrich, Burlington, USA), at a concentration of 10 mM, and diluted with PBS, and tested with J-lat 10.6 cells at concentrations of 100, 10, and 1 μM. J-lat 10.6 cells were tested with compounds at a concentration of 2.5 × 10^5^ cells/mL. We performed a minimum of three replicates of each condition for all experiments. Negative controls contained 1% DMSO to account for any effect of DMSO in the highest dilution of compounds. Additional dilutions were tested for those compounds that showed reactivation through increased GFP production in J-lat 10.6 cells.

### 4.8. Statistical Analysis

Statistical significance was analyzed using software from GraphPad Prism Version 7.0c (GraphPad Software, San Diego, CA, USA). The mean values and standard deviations for all replicate J-lat results were calculated and used to create Figure 4, Figure 5, Figure 6 and Figure 7. Where applicable, Students t-test was used to determine statistical significance of experimental mean results relative to negative controls.

## 5. Conclusions

In this study, 4*β*-crotignoid K (**14**) revealed a very higher effect improvement compared to crotignoid K (**13**), indicating that configuration at the C-4 of tigliane diterpenoids is critical to HIV-1 latency reversal activity. (+)-Syringaresinol (**26**) and dimeric coniferyl acetate (**27**) showed no exhibition of CD69 expression, suggesting a non-PKC mechanism of latency reversal. Our results provide insights into the stereochemistry importance of bioactive diterpenoids and suggest that isolated compounds from *E. usambarica* can further research and development into therapeutic strategies for HIV-1 management, particularly as reactivators of latent HIV-1.

## Figures and Tables

**Table 1:** ^1^H NMR data of compounds **1**–**6** in CDCl_3_ at 500 MHz (*δ*_H_ in ppm, mult. *J* in Hz).

Position	1	2	3	4	5	6
1	7.52, br s	a: 2.87, d (16.5)	a: 2.89, d (17.0)	a: 2.87, d (16.5)	a: 2.76, d (16.0)	a: 2.75, d (16.0)
		b: 2.09, d (16.5)	b: 2.08, d (17.0)	b: 2.18, d (16.5)	b: 2.04, d (16.0)	b: 2.06, d (16.0)
3		4.26, dd (9.5, 3.5)	4.25, dd (9.5, 3.5)	4.52, dd (12.5, 4.0)	5.80, dd (4.5, 1.0)	5.82, dd (4.0, 1.0)
4	a: 2.52, dd (18.5, 6.5)	2.53, m	2.54, m	2.63, m	3.92, m	3.92, m
	b: 2.24, dd (18.5, 3.5)					
5		6.53, d (2.0)	6.55, d (2.0)	6.09, m	5.70, d (10.0)	5.70, d (10.0)
7	6.68, dd (6.5, 1.5)	5.39, s	5.38, s	5.40, s	6.39, s	6.39, s
8	2.37, br d (6.5)	5.73, d (4.5)	5.75, d (4.5)	5.77, d (5.0)	5.72, s	5.75, s
9	1.02, d (2.0)	4.88, d (4.5)	4.91, d (4.5)	4.83, d (5.0)	4.99, s	5.01, s
11		5.49, d (16.0)	5.48, d (16.0)	5.41, d (16.0)	6.16, d (16.0)	6.16, d (16.0)
12	5.86, d (10.5)	5.83, dd (16.0, 10.0)	5.85, dd (16.0, 9.5)	5.72, dd (16.0, 10.0)	5.43, dd (16.0, 10.0)	5.41, dd (16.0, 10.0)
13	2.10, dq (10.5, 6.5)	2.46, m	2.46, m	2.55, m	3.97, m	3.98, m
14		4.89, s	4.89, s	5.18, s		
15	3.80, m					
16	1.74, dd (2.5, 1.5)	1.73, s	1.73, s	1.72, s	1.56, s	1.57, s
17	2.02, br s	a: 3.31, m	a: 3.33, m	a: 3.09, ddd (15.5, 7.5, 2.5)	a: 2.72, m	a: 2.69, m
		b: 2.12, m	b: 2.06, m	b: 2.40, m	b: 2.01, m	b: 1.98, m
18	1.43, s	0.93, s	0.93, s	0.93, s	0.98, s	0.97, s
19	1.16, s	1.13, s	1.12, s	0.96, s	1.44, s	1.43, s
20	0.91, d (6.5)	1.14, d (6.5)	1.14, d (7.0)	1.15, d (7.0)	1.23, d (7.0)	1.23, d (6.5)
21		a: 2.36, m	a: 2.35, dd (11.5, 7.5)	a: 2.65, m	a: 3.43, m	a: 3.53, m
		b: 2.27, m	b: 2.28, dd (11.5, 5.0)	b: 2.16, m	b: 2.50, m	b: 2.52, m
2-OAc				2.13, s	2.26, s	2.27, s
3-OAc					2.05, s	2.05, s
3-OH		3.45, d (9.5)	3.44, d (9.5)	3.03, d (12.5)		
5-OBz		7.93, m 7.52, m 7.39, m	7.93, m 7.53, m 7.39, m			
6-OAc		2.14, s	2.15, s			
6-OBz				7.92, m 7.58, m 7.41, m	7.88, m 7.65, m 7.51, m	7.90, m 7.67, m 7.51, m
7-O*i*Bu			2.61, h (7.0) 1.21, d (7.0) 1.20, d (7.0)			2.63, h (7.0) 1.26, d (7.0) 1.22, d (7.0)
7-OPr		2.41, q (7.5) 1.16, t (7.5)		2.57, m 2.50, m 1.22, t (7.5)	2.49, m 2.31, m 1.23, m	
8-OAc		2.08, s	2.09, s	1.35, s	2.00, s	2.02, s
9-OAc		2.17, s	2.17, s	2.07, s	2.03, s	2.07, s
11-OAc	1.97, s					
12-OBz	8.02, dd (8.5, 1.5) 7.58, m 7.46, dd (8.5, 7.5)					
14-OAc		2.56, s	2.56, s	2.45, s		
15-OH		3.07, s	3.05, s	4.61, s	4.04, s	4.08, s

**Table 2:** ^13^C NMR data of compounds **1**–**6** in CDCl_3_ at 125 MHz (*δ*_C_ in ppm).

Position	1	2	3	4	5	6
1	160.2	49.1	49	45.7	52.5	52.5
2	141.3	89.5	89.5	90.1	87.7	87.7
3	206.8	80.5	80.4	80.7	80	79.8
4	37.9	43.3	43.1	44.9	45.2	45.1
5	163.4	72.8	73.1	85	73.2	72.9
6	127.8	92.5	92.4	84.4	81.1	81.6
7	141.4	70.8	70.5	69.1 ^e^	68.2	68.1
8	35.2	68.9	69	69.2 ^e^	68.1	68.1
9	34	79.4	78.9	78.8	81.7	81.7
10	24.9	41.2	41.2	40.6	40.3	40.4
11	63.4	134.6	134.6	135.3	137.4	137.3
12	76.2	133	132.9	134.1	128.9	128.9
13	38.9	37.4	37.4	39	43.9	43.6
14	85.8	81.6	81.5	82.2	211.4	211.6
15	45.8	86.3	86.3	87.1	84.6	84.6
16	10.2	19.1	19	20	18.5	18.5
17	17.4	24.5	24	23.4	26.5	26.6
18	16.5	26	26	26.8	25.8	26
19	24.8	21.7 ^a^	21.2 ^c^	21.9	23.2	23.1
20	12.5	23.2	23.1	22.4	21.7	21.6
21		26	28.3	26	29.1	29.2
22		175.1	174.9	168.1	172.7	172.7
2-OAc				169.7 ^f^, 22.7	169.6 ^g^, 22.4	169.6 ^i^, 22.4
3-OAc					169.1 ^g^, 20.6 ^h^	169.2 ^i^, 21.0 ^j^
5-OBz		164.5, 133.5, 129.7, 129.6, 128.8	164.3, 133.3, 129.6, 129.5, 128.7			
6-OAc		169.8, 21.5 ^a^	169.6 ^d^, 21.6			
6-OBz				163.9, 133.8 130.1, 130.0 128.7	165.8, 133.81 30.6, 129.7 128.5	166.0, 133.8 130.7, 129.7 128.5
7-O*i*Bu			176.4, 34.2 18.8, 18.5			175.2, 34.5 19.0, 18.1
7-OPr		174.2 ^b^, 27.7, 8.9		174.6, 27.5, 8.9	173.4, 27.6, 8.6	
8-OAc		170, 21.6 ^a^	169.9 ^d^, 21.4 ^c^	169.9 ^f^, 21.0	170.0 ^g^, 21.2 ^h^	170.0 ^i^, 21.1 ^j^
9-OAc		170.2, 22.7	169.8 ^d^, 22.6	169.8 ^f^, 21.5	169.9 ^g^, 20.9 ^h^	170.1 ^i^, 20.9 ^j^
11-OAc	170.4, 21.1					
12-OBz	166.2, 133.2 130.4, 129.9 128.6					
14-OAc		174.3 ^b^, 21.4 ^a^	174.3, 21.3 ^c^	170.8, 20.8		

^a–j^: Exchangeable.

**Table 3:** ^1^H (500 MHz) and ^13^C (125 MHz) NMR data of compounds **7**–**9** in CDCl_3_ (*δ* in ppm).

Position	7		8		9	
	*δ*_H_, mult. (*J* in Hz)	*δ*_C_	*δ*_H_, mult. (*J* in Hz)	*δ*_C_	*δ*_H_, mult. (*J* in Hz)	*δ*_C_
1	5.46, s	79.8	a: 2.95, d (15.5) b: 2.27, d (15.5)	51.4	a: 2.83, d (16.5) b: 2.24, d (16.5)	52.0
2		90.6		92.0		88.8
3	4.36, dd (10.5, 5.5)	78.0	4.67, dd (10.0, 4.5)	79.1	5.48, d (4.0)	84.7
4	2.76, m	41.4	3.32, m	47.9	2.97, dd (4.0, 3.5)	44.2
5	6.00, br s	71.0	5.67, br s	69.2	6.58, d (3.5)	77.9
6		144.4		144.9		81.8
7	5.21, s	68.8	5.41, br s	68.5	5.27, s	68.4
8	4.30, d (11.0)	70.2	5.18, s	70.7	5.71, d (6.5)	70.0
9	4.79, s	86.6	4.96, s	80.6	4.96, d (6.5)	78.4
10		40.1		41.1		40.7
11	5.93, d (16.5)	134.0	5.87, d (15.5)	137.6	5.50, d (16.0)	134.7
12	5.76, d (16.5)	130.9	5.57, dd (15.5, 9.5)	129.6	5.79, d (16.0)	134.1
13	2.76, m	36.9	3.75	44.4	2.69, m	36.9
14	4.78, s	76.9		211.2	5.04, s	80.4
15		84.8		89.0		85.4
16	1.55, s	17.1	1.89, s	20.9	1.75, s	19.8
17	a: 5.26, s; b: 5.10, s	110.4	a: 5.41, s; b: 5.16, s	111.6	a: 1.85, m; b: 1.73, m	32.0
18	1.03, s	27.6	0.91, s	26.5	0.98, s	26.4
19	1.40, s	23.4	1.36, s	23.2	1.04, s	20.8 ^e^
20	1.06, d (7.0)	23.9	1.24, d (6.5)	19.6	1.11, d (7.0)	22.4
21					a: 3.21, m; b: 2.33, m	28.1
22						173.5
1-OAc	2.24, s	170.1, 21.0 ^a^				
2-OAc	2.12, s	170.9, 22.5			2.19, s	169.6, 22.9
2-ONic			9.41, dd (2.0, 1.0) 8.79, dd (5.0, 2.0) 8.52, m; 7.39, m	164.9, 153.4 151.5, 137.6 127.5, 123.2		
3-OH	3.36, d (10.5)		3.57, d (10.0)			
5-OBz	8.00, m 7.56, m 7.42, m	165.4, 133.4 130.1, 129.7 128.8	8.06, m 7.56, m 7.44, m	164.7, 133.4 131.1, 130.0 128.7	8.07, m 7.57, m 7.46, m	168.3, 133.9 130.1, 128.8 128.6
6-OH					3.57, s	
7-OAc					2.14, s	171.0, 20.9 ^e^
7-O*i*Bu	2.55, h (7.0) 1.19, d (7.0) 1.14, d (7.0)	175.1, 34.0 19.6, 18.4	2.60, h (7.0) 1.23, d (7.0) 1.11, d (7.0)	175.8, 34.0 19.7, 18.4		
8-OAc			2.00, s	169.9 ^c^, 20.8 ^d^	2.15, s	171.2, 21.7 ^f^
8-OH	3.15, d (11.0)					
9-OAc	2.06, s	172.1 ^b^, 20.9 ^a^	2.07, s	169.7 ^c^, 20.7 ^d^	2.16, s	170.4, 21.4 ^f^
14-OAc	1.70, s	172.2 ^b^, 20.4			2.36, s	172.0, 20.7 ^e^
15-OH	2.75, s		4.34, s		2.40, s	

^a–f^: Exchangeable.

**Table 4:** ^1^H NMR data of compounds **14**–**17**, **19**, and **20** in CDCl3 at 500 MHz (*δ*_H_ in ppm, mult. *J* in Hz).

Position	14	15	16	17	19	20
1	7.57, s	7.06, br s	a: 2.68, m	a: 2.42, m	a: 2.05, m	a: 1.98, m
			b: 2.05, m	b: 1.73, m	b: 1.75, m	b: 1.24, m
2			a: 2.91, m	a: 1.92, m	a: 2.65, ddd (15.5, 14.0, 6.0)	a: 1.73, m
			b: 2.59, ddd (15.5, 5.5, 3.0)	b: 1.87, m	b: 2.37, ddd (15.5, 4.8, 3.2)	b: 1.62, m
3				3.37, dd (11.5, 4.0)		3.30, dd (12.0, 4.0)
4	2.52, m	3.13, dd (6.5, 4.5)				
5	a: 2.87, dd (18.5,9.5)	4.46, dd (11.5, 4.5)	2.36, dd (14.0, 3.5)	1.88, m	1.67, m	1.05, m
	b: 2.19, dd (18.5,4.0)					
6			a: 2.83, dd (17.5, 14.0)	a: 2.79, dd (18.0, 13.5)	a: 1.79, m	a: 1.79, m
			b: 2.75, dd (17.5, 3.5)	b: 2.77, dd (18.0, 4.5)	b: 1.70, m	b: 1.52, m
7	5.56, m	4.88, br s			a: 2.17, m	a: 1.98, m
					b: 1.68, m	b: 1.66, m
8	2.46, t (5.5)	2.06, m				
9					2.70, d (5.0)	1.95, m
10	3.28, m	3.65, m				
11	1.75, m	1.86, dd (10.5, 6.5)	7.49, d (8.5)	7.47, d (8.0)	5.44, d (5.0)	a: 2.27, dd (13.5, 5.5)
						b: 1.41, m
12	5.68, d (10.0)	5.73, d (10.5)	8.17, dd (8.5, 2.5)	8.14, dd (8.0, 2.0)		4.99, ddd (13.0, 5.5, 2.0)
13						
14	1.14, d (5.5)	0.89, d (6.5)	8.57, d (2.5)	8.55, d (2.0)	3.76, br s	3.77, s
15						
16	1.21, s	1.20, s				
17	1.33, s	1.33, s	2.64, s	2.63, s	2.09, s	1.97, d (2.0)
18	0.98. d (6.5)	1.16, d (6.5)	1.17, s	0.99, s	1.17, s	1.06, s
19	1.73, dd (2.5, 1.0)	1.83, br s	1.23, s	1.08, s	1.09, s	0.88, s
20	4.05, m	1.90, s	1.48, s	1.27, s	0.95, s	1.08, s
5-OH		5.92, d (11.5)				
9-OH	5.62, s	5.95, s				
12-OBz	8.02, m 7.59, m 7.47, m	8.06, m 7.61, m 7.49, m				
13-OAc	2.14, s	2.11, s				

**Table 5:** ^13^C NMR data of compounds **14**–**17**, **19**, and **20** in CDCl_3_ at 125 MHz (*δ*_C_ in ppm).

Position	14	15	16	17	19	20
1	159.7	154.6	36.8	35.9	38.1	38.7
2	136.7	144.3	34.6	27.5	34.2	27.3
3	208.7	207.5	214.0	78.0	215.0	78.6
4	44.4	56.3	47.6	39.1	48.1	39.0
5	29.8	71.1	49.2	48.3	54.1	53.6
6	142.3	138.0	36.5	36.1	21.7	20.8
7	126.6	125.5	197.4 ^a^	198.5	33.9	34.8
8	42.3	40.2	130.8	130.8	61.0	61.0
9	78.0	78.7	158.2	159.9	50.1	49.2
10	54.3	48.0	38.3	38.5	40.9	39.2
11	42.8	43.5	125.2	124.8	102.9	24.0
12	77.8	75.7	133.4	133.2	148.1	75.6
13	65.5	65.4	135.9	135.5	144.7	155.6
14	36.0	38.5	128.3	128.1	54.5	56.2
15	26.0	25.1	197.3 ^a^	197.4	126.2	128.9
16	23.9	24.3			170.5	174.1
17	17.1	16.7	26.9	26.8	9.0	8.9
18	15.3	11.9	25.2	27.6	25.9	29.1
19	10.3	10.6	21.7	15.2	22.4	16.1
20	67.6	27.2	22.8	23.3	15.0	19.3
12-OBz	166.4 133.4 130.1 129.9 128.7	166.4 133.4 130.1 129.9 128.7				
13-OAc	173.9 21.3	174.1 21.2				

^a^: Exchangeable.

## Data Availability

Data sharing not applicable.

## Supplementary Materials

The following are available online at https://www.mdpi.com/article/10.3390/ph14070653/s1, Figures S1–S105: 1D-NMR, 2D-NMR, and HR-ESIMS spectra of compounds **1**–**9**, **14**–**16**, **19**, and **20**. Figure S106: 24 h cell viability after exposure to the compounds **1**–**31**.
