# Supplementary material for: Bioactive Compounds from Euphorbia usambarica Pax. with HIV-1 Latency Reversal Activity
Source: Pharmaceuticals (Basel). 2021 Jul 7;14(7):653. doi: 10.3390/ph14070653 (PMC8308672; doi:10.3390/ph14070653)
Supplement: Supplementary file 1 [file pharmaceuticals-14-00653-s001.zip › pharmaceuticals-1267461-supplementary.pdf]

Supplementary Materials

Bioactive Compounds from *Euphorbia usambarica* Pax. with HIV-1 Latency Reversal Activity

Yu-Chi Tsai [1], Racheal A. Nell [2], Jonathan E. Buckendorf [2], Norbert Kúsz [1], Peter Waweru Mwangi [3], Róbert Berkecz [4], Dóra Rédei [1], Andrea Vasas [1], Adam M. Spivak [2,*] and Judit Hohmann [1,5,*]

1 Department of Pharmacognosy, Interdisciplinary Excellence Centre, University of Szeged, H-6720 Szeged, Hungary
2 Department of Medicine, University of Utah School of Medicine, Salt Lake City, Utah 84132, USA
3 Department of Medical Physiology, School of Medicine, University of Nairobi, P.O Box 30197-00100, Nairobi, Kenya
4 Department of Medical Chemistry, University of Szeged, H-6720 Szeged, Hungary
5 Interdisciplinary Centre of Natural Products, University of Szeged, H-6720 Szeged, Hungary
* Correspondence: adam.spivak@hsc.utah.edu (A.M.S.); hohmann.judit@szte.hu (J.H.)

TABLE OF CONTENTS

Figure S1. The ^{1}H-NMR spectrum of euphordraculoate C (**1**) (500 MHz, $CDCl_3$). 7

Figure S2. The ^{13}C-JMOD spectrum of euphordraculoate C (**1**) (125 MHz, $CDCl_3$). 7

Figure S3. The HSQC spectrum of euphordraculoate C (**1**). 8

Figure S5. The HMBC spectrum of euphordraculoate C (**1**). 9

Figure S6. The NOESY spectrum of euphordraculoate C (**1**). 9

Figure S7. The HR-ESIMS spectra of euphordraculoate C (**1**). 10

Figure S8. The ^{1}H-NMR spectrum of usambariphane A (**2**) (500 MHz, $CDCl_3$). 11

Figure S9. The ^{13}C-JMOD spectrum of usambariphane A (**2**) (125 MHz, $CDCl_3$). 11

Figure S10. The HSQC spectrum of usambariphane A (**2**). 12

Figure S11. The ^{1}H-^{1}H COSY spectrum of usambariphane A (**2**). 12

Figure S12. The HMBC spectrum of usambariphane A (**2**). 13

Figure S13. The NOESY spectrum of usambariphane A (**2**). 13

Figure S14. The HR-ESIMS spectra of usambariphane A (**2**). 14

Figure S15. The ^{1}H-NMR spectrum of usambariphane B (**3**) (500 MHz, $CDCl_3$). 15

Figure S16. The ^{13}C-JMOD spectrum of usambariphane B (**3**) (125 MHz, $CDCl_3$). 15

Figure S17. The HSQC spectrum of usambariphane B (**3**). 16

Figure S18. The ^{1}H-^{1}H COSY spectrum of usambariphane B (**3**). 16

Figure S19. The HMBC spectrum of usambariphane B (**3**). 17

Figure S20. The NOESY spectrum of usambariphane B (**3**). 17

Figure S21. The HR-ESIMS spectra of usambariphane B (**3**). 18

Figure S22. The ^{1}H-NMR spectrum of usambariphane C (**4**) (500 MHz, $CDCl_3$). 19

Figure S23. The ^{13}C-JMOD spectrum of usambariphane C (**4**) (125 MHz, $CDCl_3$). 19

Figure S24. The HSQC spectrum of usambariphane C (**4**). 20

Figure S25. The ^{1}H-^{1}H COSY spectrum of usambariphane C (**4**). 20

Figure S26. The HMBC spectrum of usambariphane C (**4**). 21

Figure S27. The NOESY spectrum of usambariphane C (**4**). 21

Figure S28. The HR-ESIMS spectra of usambariphane C (**4**). 22

Figure S29. The ^{1}H-NMR spectrum of usambariphane D (**5**) (500 MHz, $CDCl_3$). 23

Figure S30. The ^{13}C-JMOD spectrum of usambariphane D (**5**) (125 MHz, $CDCl_3$).................... 23
Figure S31. The HSQC spectrum of usambariphane D (**5**).. 24
Figure S32. The ^{1}H-^{1}H COSY spectrum of usambariphane D (**5**). 24
Figure S33. The HMBC spectrum of usambariphane D (**5**)... 25
Figure S34. The NOESY spectrum of usambariphane D (**5**).. 25
Figure S36. The ^{1}H-NMR spectrum of usambariphane E (**6**) (500 MHz, $CDCl_3$). 27
Figure S37. The ^{13}C-JMOD spectrum of usambariphane E (**6**) (125 MHz, $CDCl_3$). 27
Figure S38. The HSQC spectrum of usambariphane E (**6**).. 28
Figure S39. The ^{1}H-^{1}H COSY spectrum of usambariphane E (**6**).. 28
Figure S40. The HMBC spectrum of usambariphane E (**6**). 29
Figure S41. The NOESY spectrum of usambariphane E (**6**). 29
Figure S42. The HR-ESI-MS spectra of usambariphane E (**6**). 30
Figure S43. The ^{1}H-NMR spectrum of usambariphane F (**7**) (500 MHz, $CDCl_3$)....................... 31
Figure S44. The ^{13}C-JMOD spectrum of usambariphane F (**7**) (125 MHz, $CDCl_3$). 31
Figure S45. The HSQC spectrum of usambariphane F (**7**).. 32
Figure S46. The ^{1}H-^{1}H COSY spectrum of usambariphane F (**7**).. 32
Figure S47. The HMBC spectrum of usambariphane F (**7**)... 33
Figure S48. The NOESY spectrum of usambariphane F (**7**). 33
Figure S49. The HR-ESI-MS spectra of usambariphane F (**7**).. 34
Figure S50. The ^{1}H-NMR spectrum of usambariphane G (**8**) (500 MHz, $CDCl_3$)....................... 35
Figure S51. The ^{13}C-JMOD spectrum of usambariphane G (**8**) (125 MHz, $CDCl_3$).................... 35
Figure S52. The HSQC spectrum of usambariphane G (**8**)... 36
Figure S53. The ^{1}H-^{1}H COSY spectrum of usambariphane G (**8**). 36
Figure S54. The HMBC spectrum of usambariphane G (**8**)... 37
Figure S55. The NOESY spectrum of usambariphane G (**8**).. 37
Figure S56. The HR-ESI-MS spectra of usambariphane G (**8**)... 38
Figure S57. The ^{1}H-NMR spectrum of isoterracinolide C (**9**) (500 MHz, $CDCl_3$). 39
Figure S58. The ^{13}C-JMOD spectrum of isoterracinolide C (**9**) (500 MHz, $CDCl_3$).................... 39
Figure S59. The HSQC spectrum of isoterracinolide C (**9**). 40

Figure S60. The ^{1}H-^{1}H COSY spectrum of isoterracinolide C (**9**). 40
Figure S61. The HMBC spectrum of isoterracinolide C (**9**). 41
Figure S62. The NOESY spectrum of isoterracinolide C (**9**). 41
Figure S63. The HR-ESI-MS spectra of isoterracinolide C (**9**). 42
Figure S64. The ^{1}H-NMR spectrum of 4*β*-crotignoid K (**14**) (500 MHz, $CDCl_3$). 43
Figure S65. The ^{13}C-JMOD spectrum of 4*β*-crotignoid K (**14**) (125 MHz, $CDCl_3$). 43
Figure S66. The HSQC spectrum of 4*β*-crotignoid K (**14**). 44
Figure S67. The ^{1}H-^{1}H COSY spectrum of 4*β*-crotignoid K (**14**). 44
Figure S69. The NOESY spectrum of 4*β*-crotignoid K (**14**). 45
Figure S70. The HR-ESI-MS spectra of 4*β*-crotignoid K (**14**). 46
Figure S71. The ^{1}H-NMR spectrum of euphodendriane B (**15**) (500 MHz, $CDCl_3$). 47
Figure S72. The ^{13}C-JMOD spectrum of euphodendriane B (**15**) (125 MHz, $CDCl_3$). 47
Figure S73. The HSQC spectrum of euphodendriane B (**15**). 48
Figure S74. The ^{1}H-^{1}H COSY spectrum of euphodendriane B (**15**). 48
Figure S75. The HMBC spectrum of euphodendriane B (**15**). 49
Figure S76. The NOESY spectrum of euphodendriane B (**15**). 49
Figure S77. The HR-ESI-MS spectra of euphodendriane B (**15**). 50
Figure S78. The ^{1}H-NMR spectrum of 16-nor-abieta-8,11,13-trien-3,7,15-trione (**16**) (500 MHz, $CDCl_3$). 51
Figure S79. The ^{13}C-JMOD spectrum of 16-nor-abieta-8,11,13-trien-3,7,15-trione (**16**) (125 MHz, $CDCl_3$). 51
Figure S80. The HSQC spectrum of 16-nor-abieta-8,11,13-trien-3,7,15-trione (**16**). 52
Figure S81. The ^{1}H-^{1}H COSY spectrum of 16-nor-abieta-8,11,13-trien-3,7,15-trione (**16**). 52
Figure S82. The HMBC spectrum of 16-nor-abieta-8,11,13-trien-3,7,15-trione (**16**). 53
Figure S83. The NOESY spectrum of 16-nor-abieta-8,11,13-trien-3,7,15-trione (**16**). 53
Figure S84. The HR-ESI-MS spectra of 16-nor-abieta-8,11,13-trien-3,7,15-trione (**16**). 54
Figure S85. The ^{1}H-NMR spectrum of 16-nor-3*β*-hydroxy-abieta-8,11,13-trien-7,15-dione (**17**) (500 MHz, $CDCl_3$). 55

Figure S86. The ^{13}C-JMOD spectrum of 16-nor-3*β*-hydroxy-abieta-8,11,13-trien-7,15-dione (**17**) (125 MHz, $CDCl_3$). 55
Figure S87. The HSQC spectrum of 16-nor-3*β*-hydroxy-abieta-8,11,13-trien-7,15-dione (**17**).. 56
Figure S88. The ^{1}H-^{1}H COSY spectrum of 16-nor-3*β*-hydroxy-abieta-8,11,13-trien-7,15-dione (**17**). 56
Figure S89. The HMBC spectrum of 16-nor-3*β*-hydroxy-abieta-8,11,13-trien-7,15-dione (**17**). 57
Figure S90. The NOESY spectrum of 16-nor-3*β*-hydroxy-abieta-8,11,13-trien-7,15-dione (**17**).57
Figure S91. The HR-ESI-MS spectra of 16-nor-3*β*-hydroxy-abieta-8,11,13-trien-7,15-dione (**17**). 58
Figure S92. The ^{1}H-NMR spectrum of *ent*-8*β*,14*β*-epoxyabieta-3-one-11,13(15)-dien-16,12-olide (**19**) (500 MHz, $CDCl_3$). 59
Figure S93. The ^{13}C-JMOD spectrum of *ent*-8*β*,14*β*-epoxyabieta-3-one-11,13(15)-dien-16,12-olide (**19**) (125 MHz, $CDCl_3$). 59
Figure S94. The HSQC spectrum of *ent*-8*β*,14*β*-epoxyabieta-3-one-11,13(15)-dien-16,12-olide (**19**). 60
Figure S95. The ^{1}H-^{1}H COSY spectrum of *ent*-8*β*,14*β*-epoxyabieta-3-one-11,13(15)-dien-16,12-olide (**19**). 60
Figure S96. The HMBC spectrum of *ent*-8*β*,14*β*-epoxyabieta-3-one-11,13(15)-dien-16,12-olide (**19**). 61
Figure S97. The NOESY spectrum of *ent*-8*β*,14*β*-epoxyabieta-3-one-11,13(15)-dien-16,12-olide (**19**). 61
Figure S98. The HR-ESI-MS spectra of *ent*-8*β*,14*β*-epoxyabieta-3-one-11,13(15)-dien-16,12-olide (**19**). 62
Figure S99. The ^{1}H-NMR spectrum of *ent*-8*β*,14*β*-epoxyabieta-3*α*-hydroxy-13(15)-en-16,12-olide (**20**) (500 MHz, $CDCl_3$). 63
Figure S100. The ^{13}C-JMOD spectrum of *ent*-8*β*,14*β*-epoxyabieta-3*α*-hydroxy-13(15)-en-16,12-olide (**20**) (125 MHz, $CDCl_3$). 63
Figure S101. The HSQC spectrum of *ent*-8*β*,14*β*-epoxyabieta-3*α*-hydroxy-13(15)-en-16,12-olide (**20**). 64

Figure S102. The ^1H-^1H COSY spectrum of *ent*-8*β*,14*β*-epoxyabieta-3*α*-hydroxy-13(15)-en-16,12-olide (**20**). 64
Figure S103. The HMBC spectrum of *ent*-8*β*,14*β*-epoxyabieta-3*α*-hydroxy-13(15)-en-16,12-olide (**20**). 65
Figure S104. The NOESY spectrum of *ent*-8*β*,14*β*-epoxyabieta-3*α*-hydroxy-13(15)-en-16,12-olide (**20**). 65
Figure S105. The HR-ESI-MS spectra of *ent*-8*β*,14*β*-epoxyabieta-3*α*-hydroxy-13(15)-en-16,12-olide (**20**). 66
Figure S106. 24 hours cell viability after exposure to the compounds **1** – **31**. 67

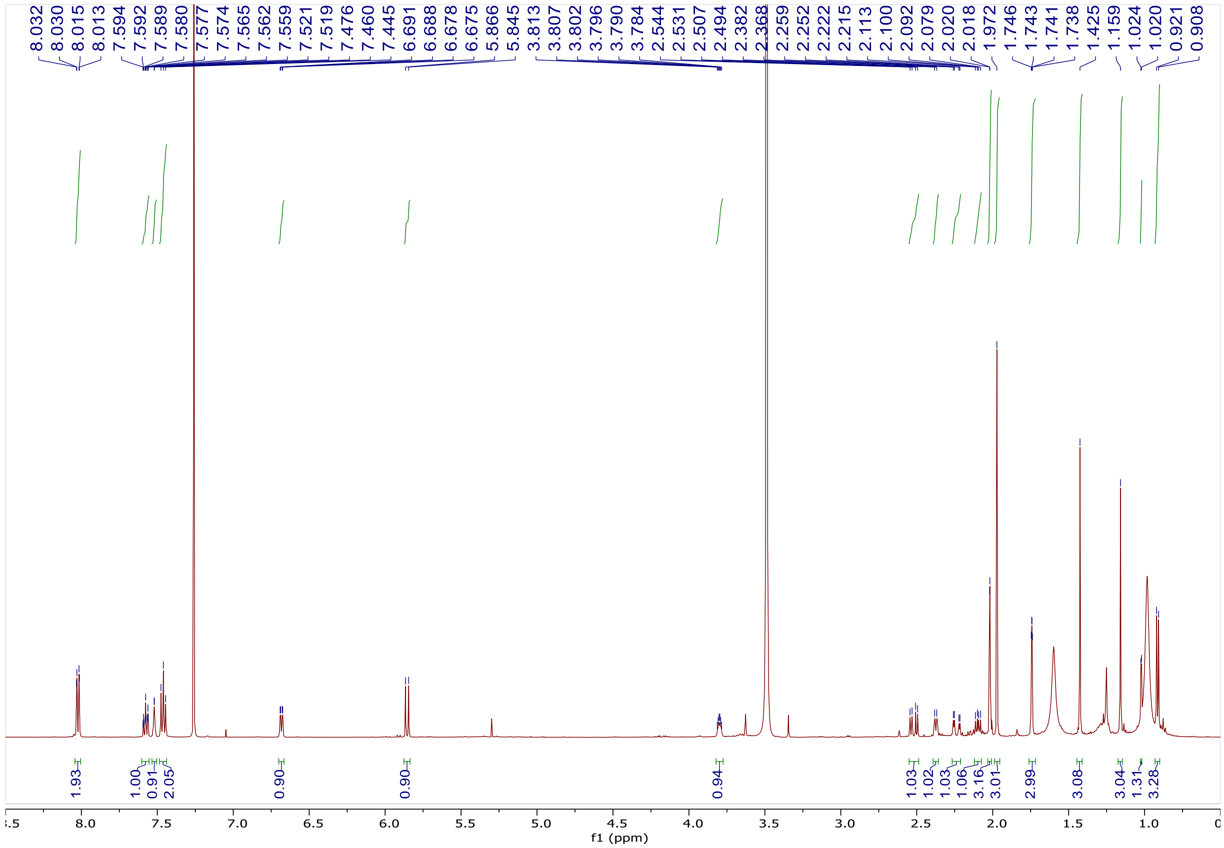

Figure S1. The ¹H-NMR spectrum of euphordraculoate C (**1**) (500 MHz, $CDCl_3$).

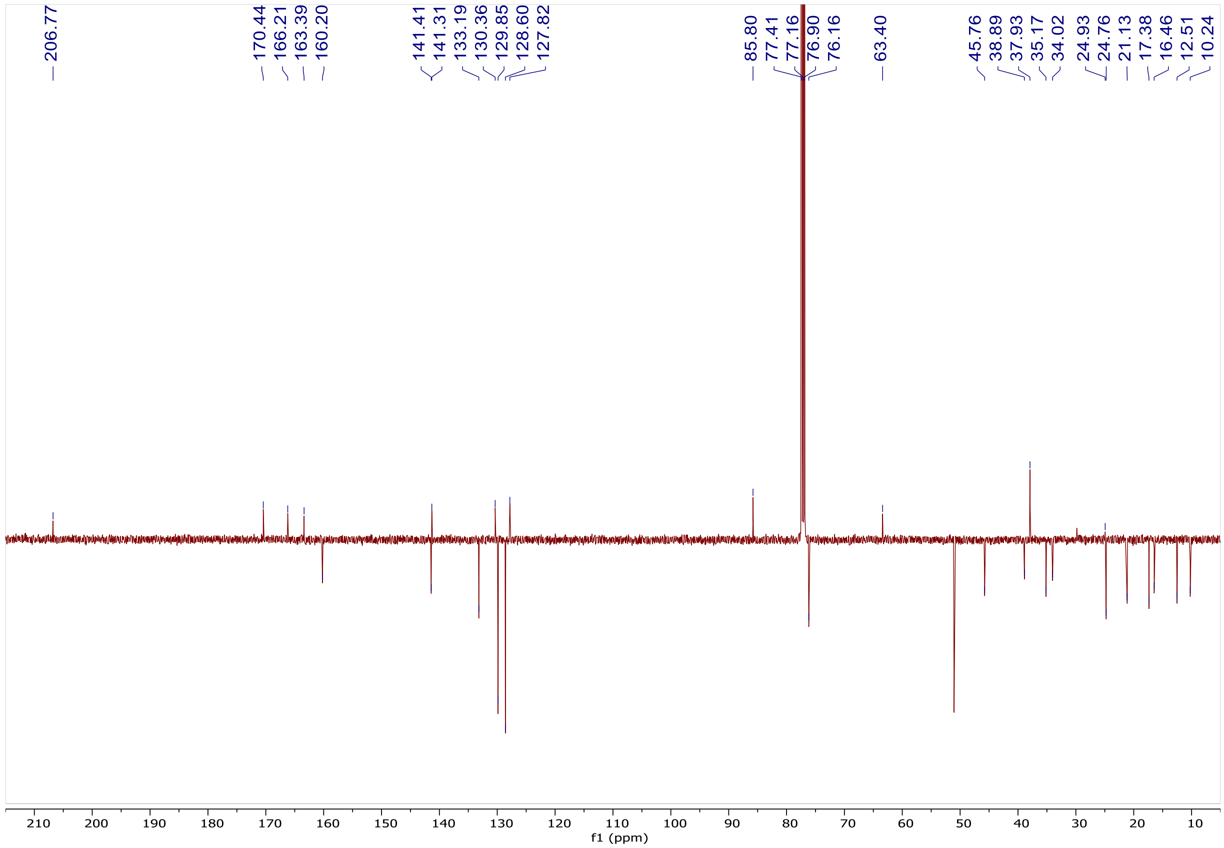

Figure S2. The ¹³C-JMOD spectrum of euphordraculoate C (**1**) (125 MHz, $CDCl_3$).

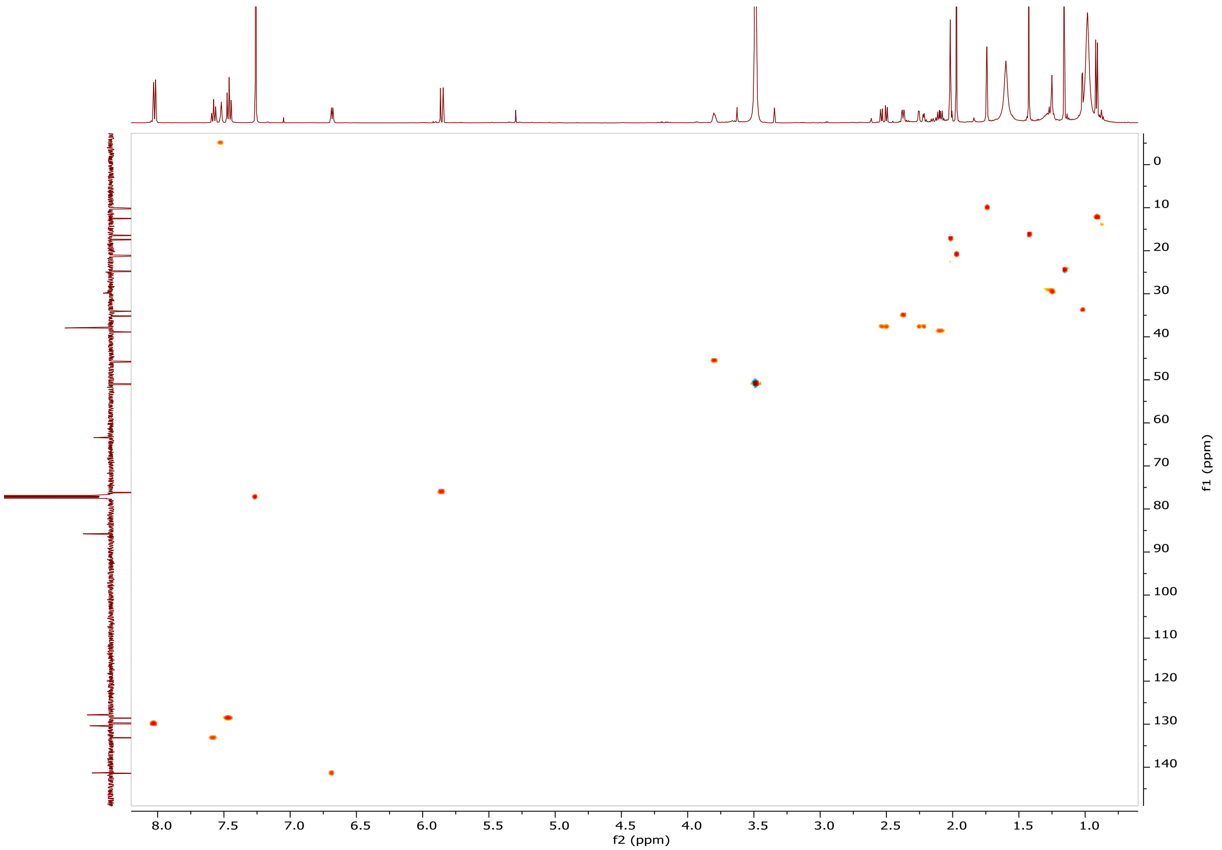

Figure S3. The HSQC spectrum of euphordraculoate C (**1**).

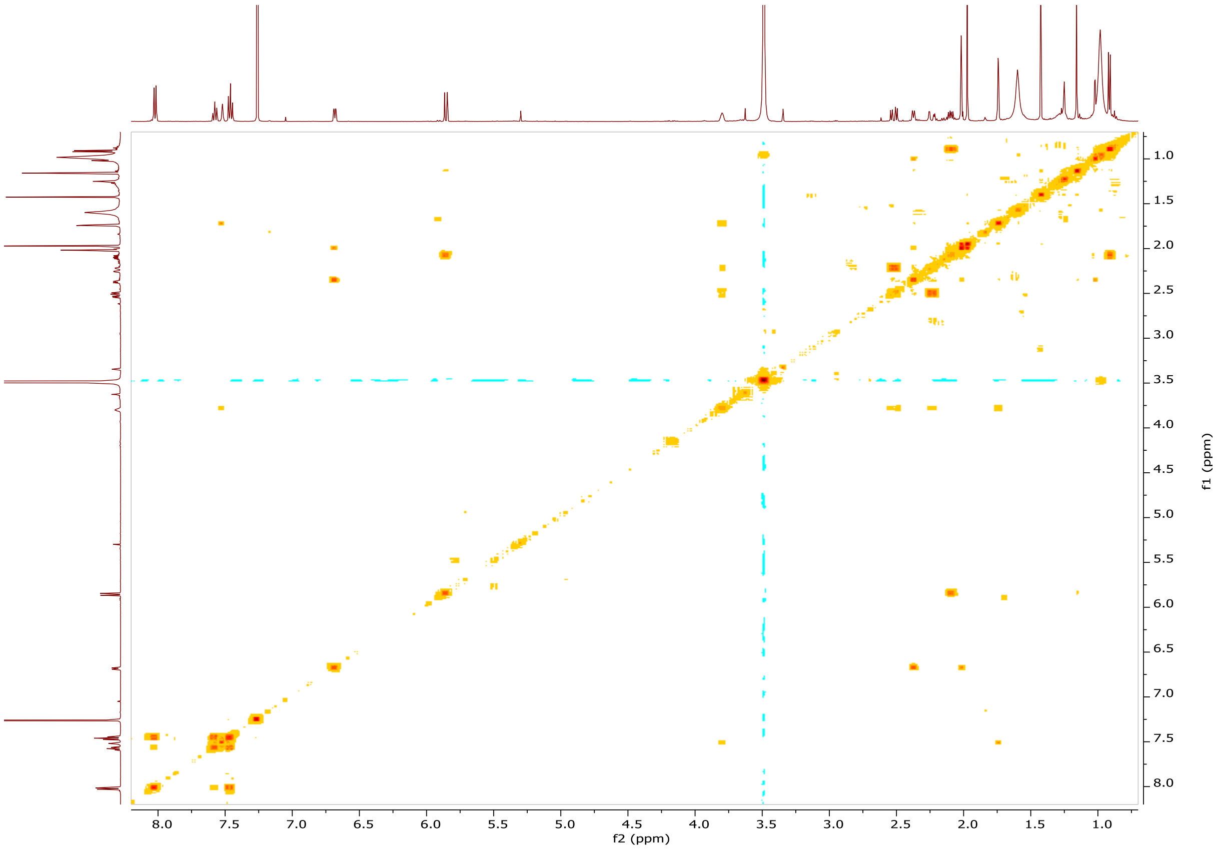

Figure S4. The ¹H-¹H COSY spectrum of euphordraculoate C (**1**).

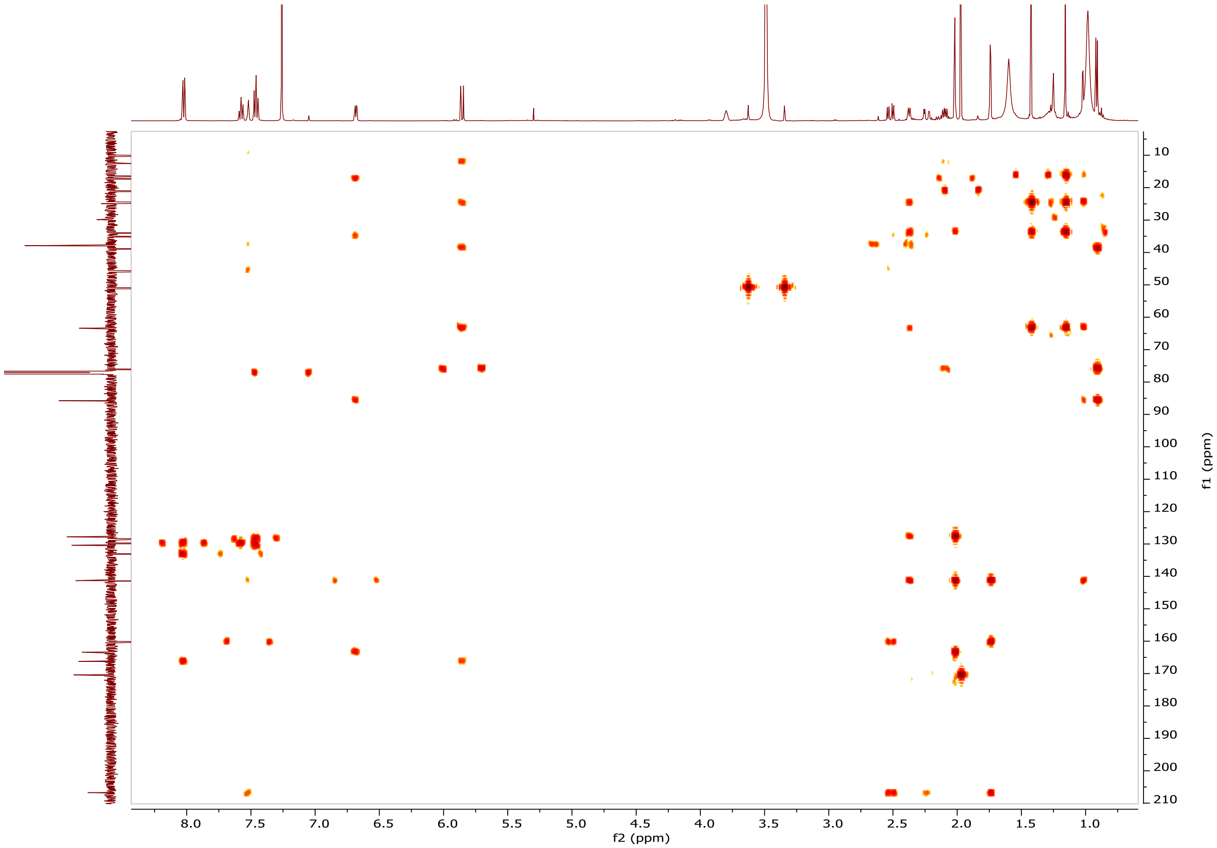

Figure S5. The HMBC spectrum of euphordraculoate C (**1**).

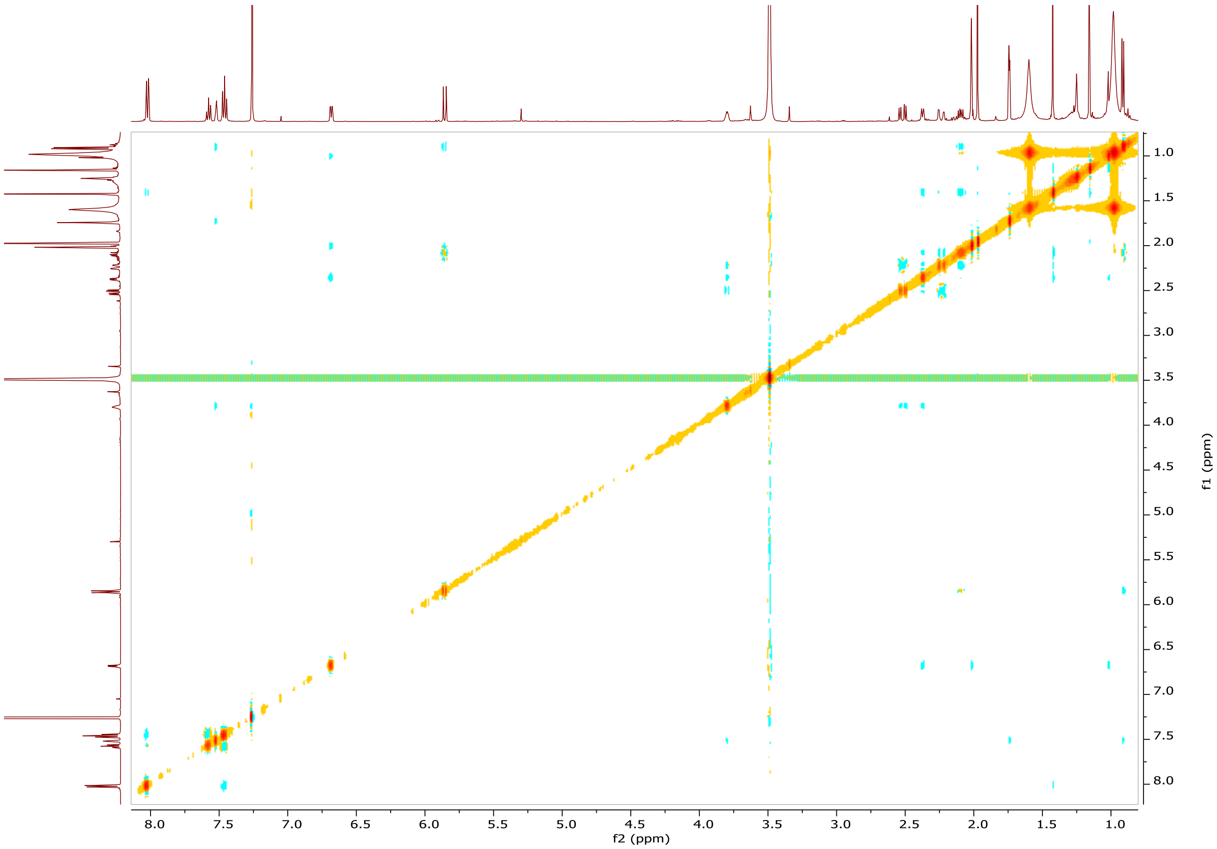

Figure S6. The NOESY spectrum of euphordraculoate C (**1**).

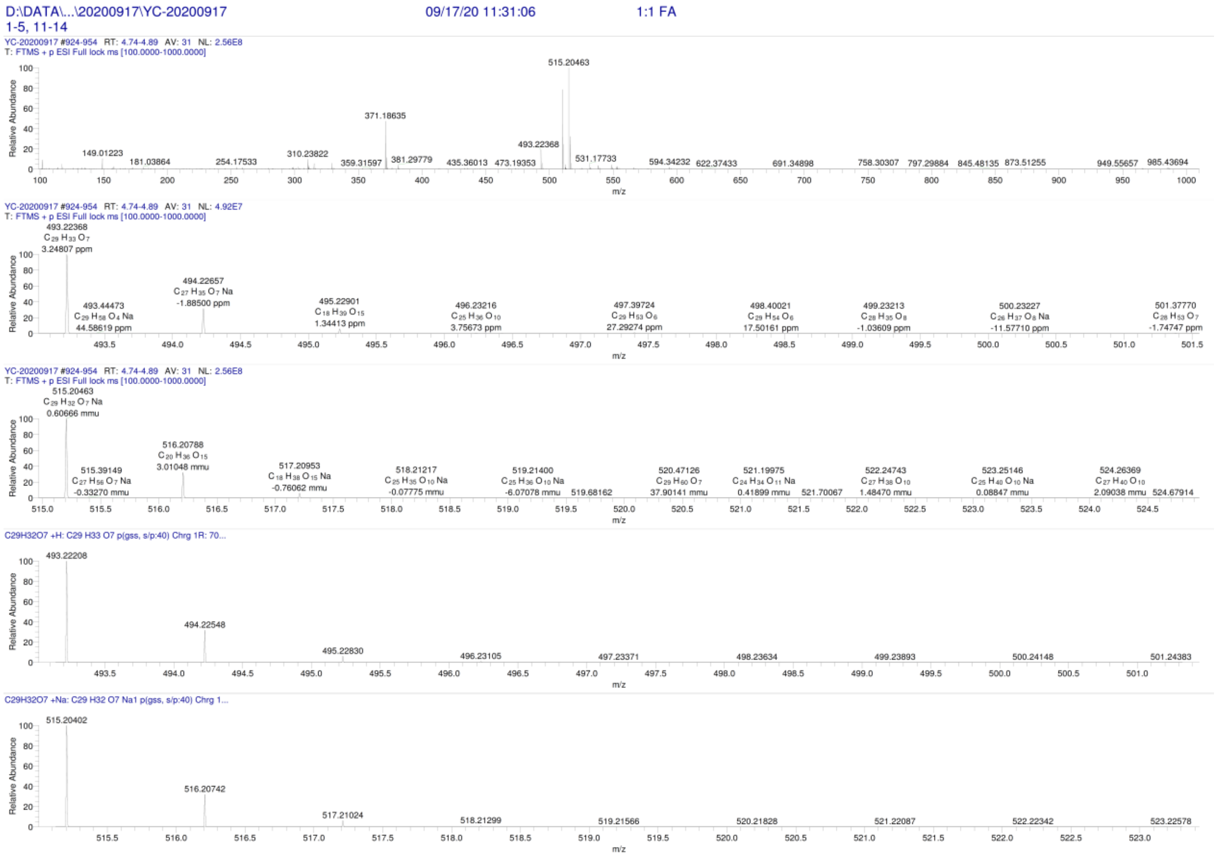

Figure S7. The HR-ESIMS spectra of euphordraculoate C (**1**).

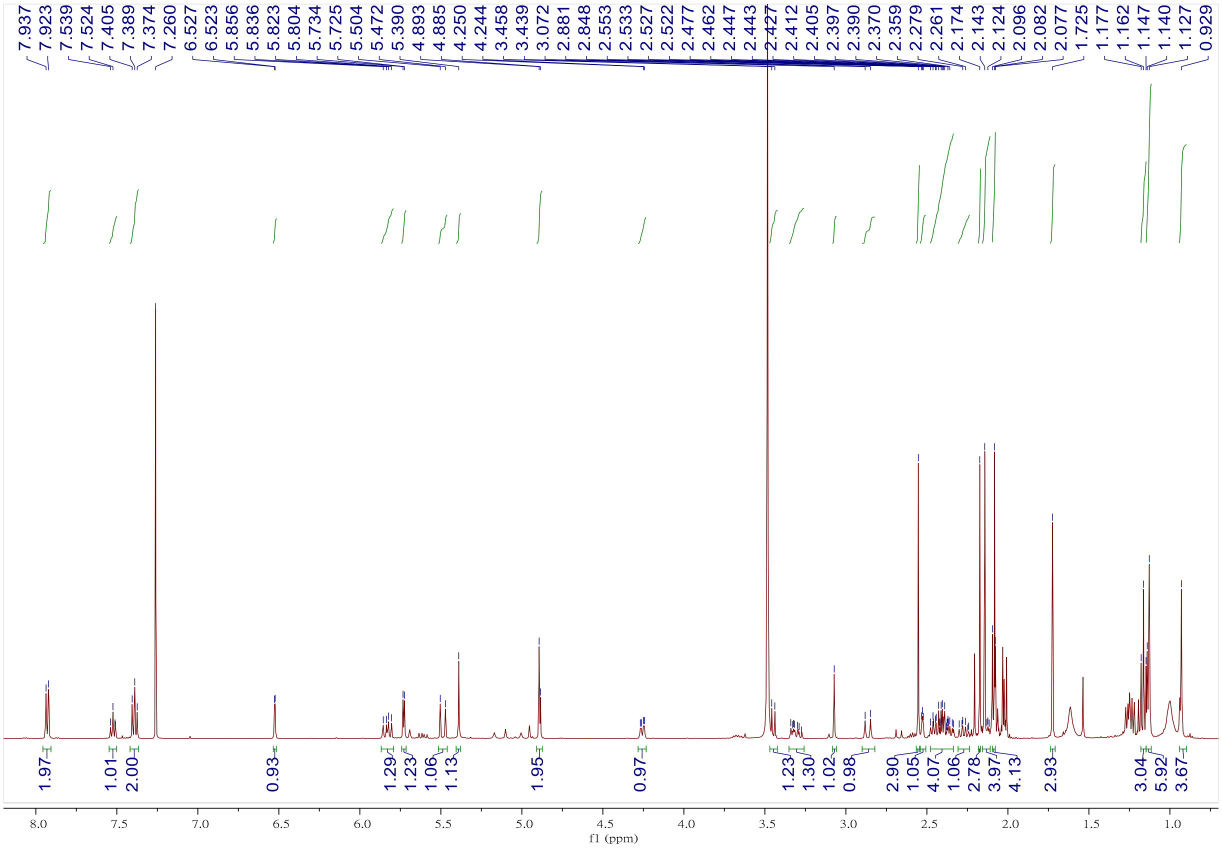

Figure S8. The ^{1}H-NMR spectrum of usambariphane A (**2**) (500 MHz, $CDCl_3$).

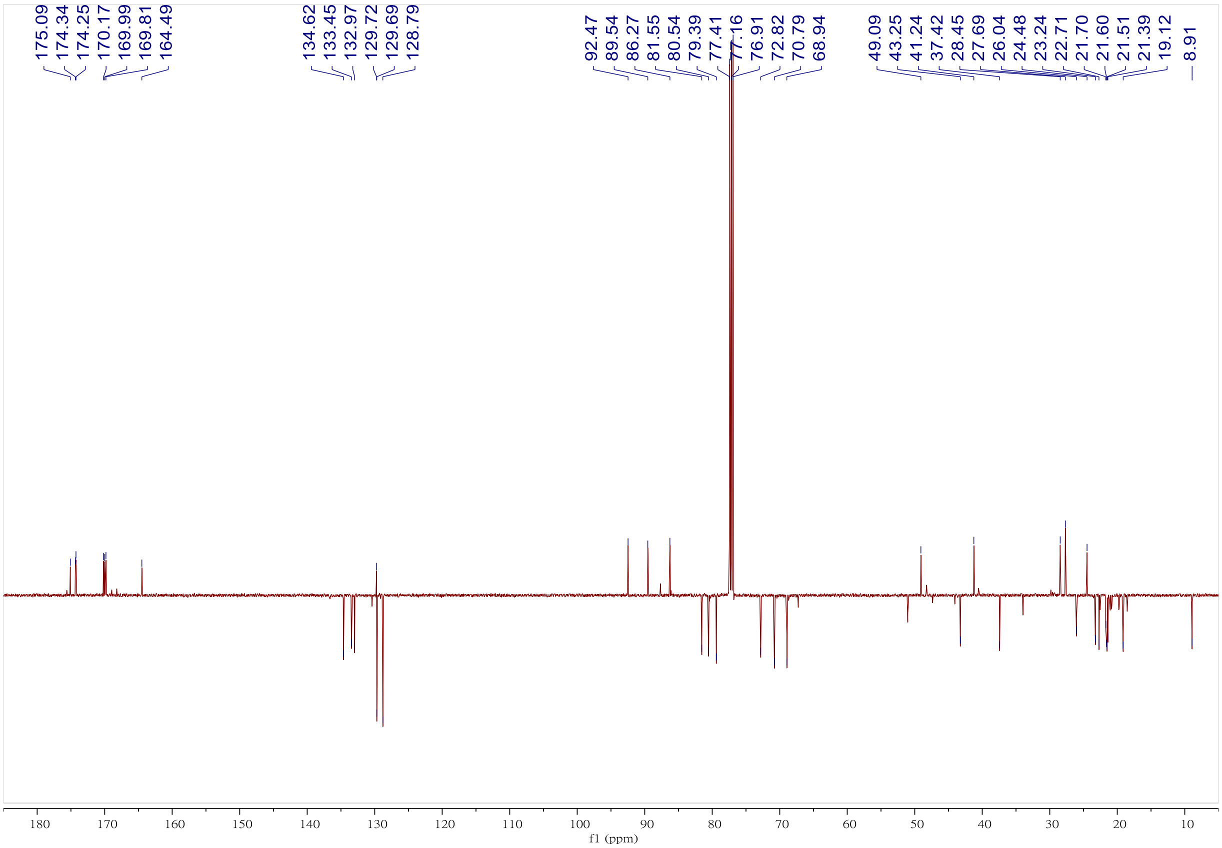

Figure S9. The ^{13}C-JMOD spectrum of usambariphane A (**2**) (125 MHz, $CDCl_3$).

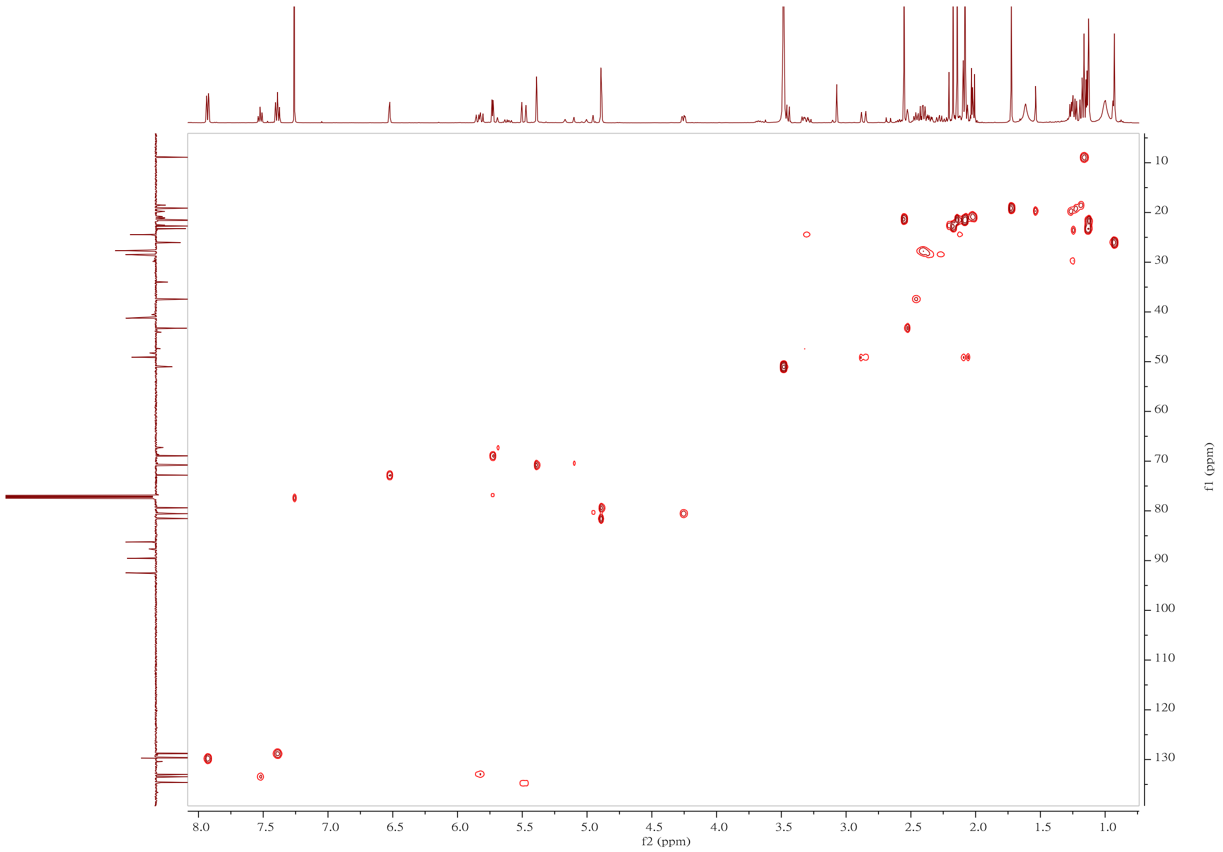

Figure S10. The HSQC spectrum of usambariphane A (**2**).

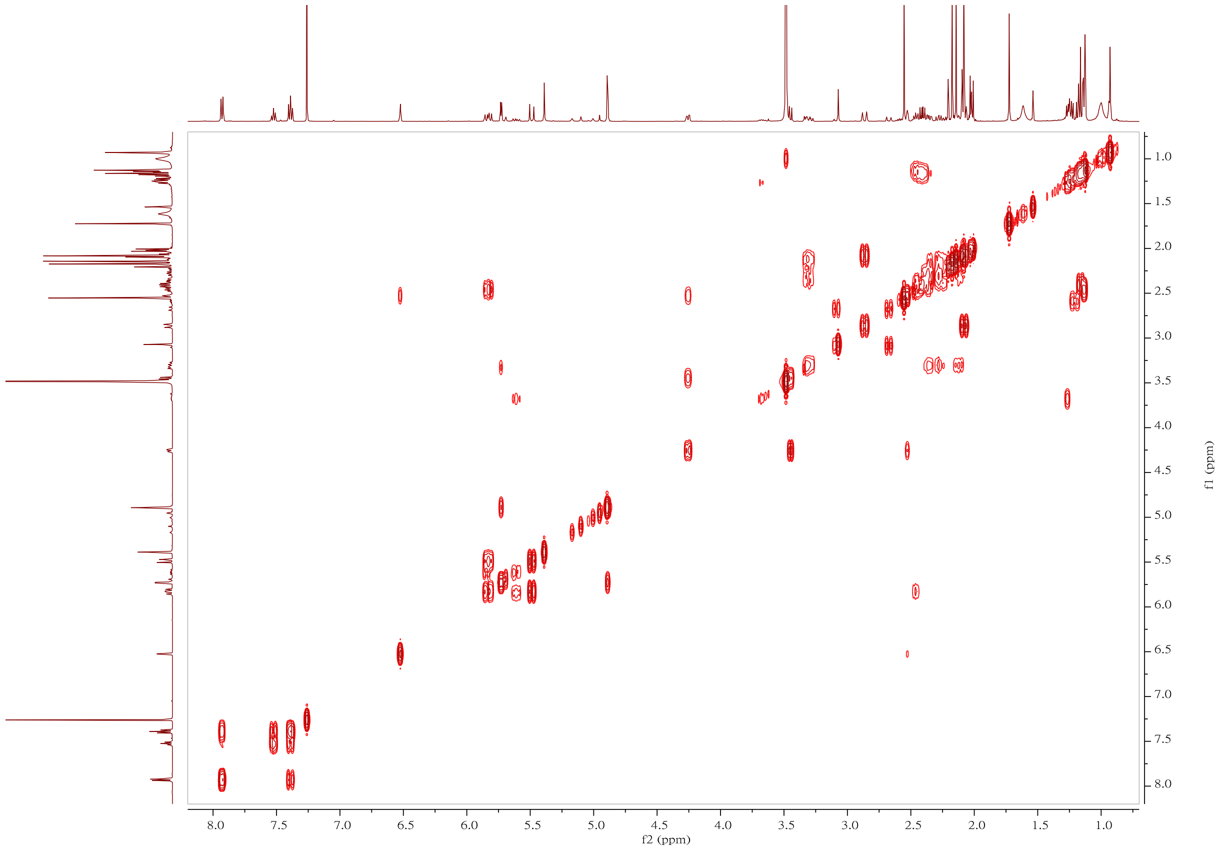

Figure S11. The ^{1}H-^{1}H COSY spectrum of usambariphane A (**2**).

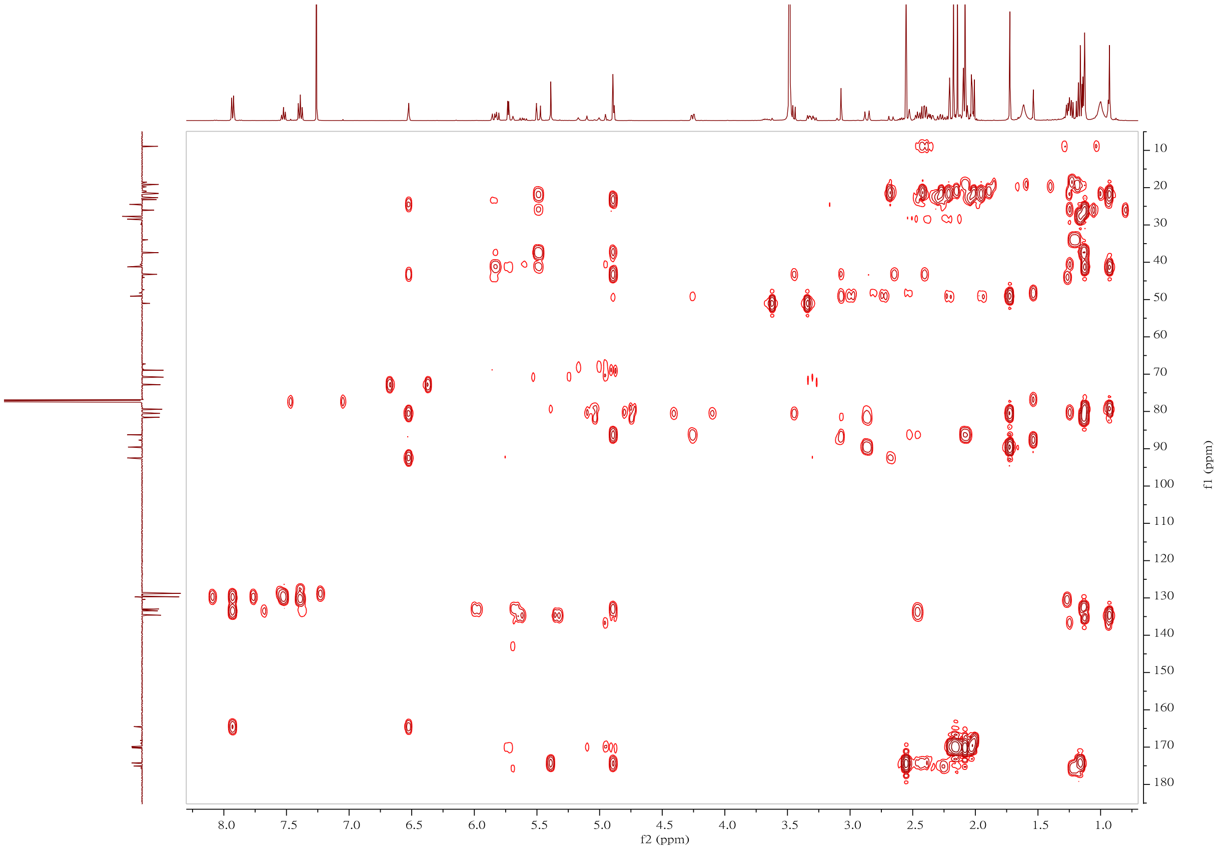

Figure S12. The HMBC spectrum of usambariphane A (**2**).

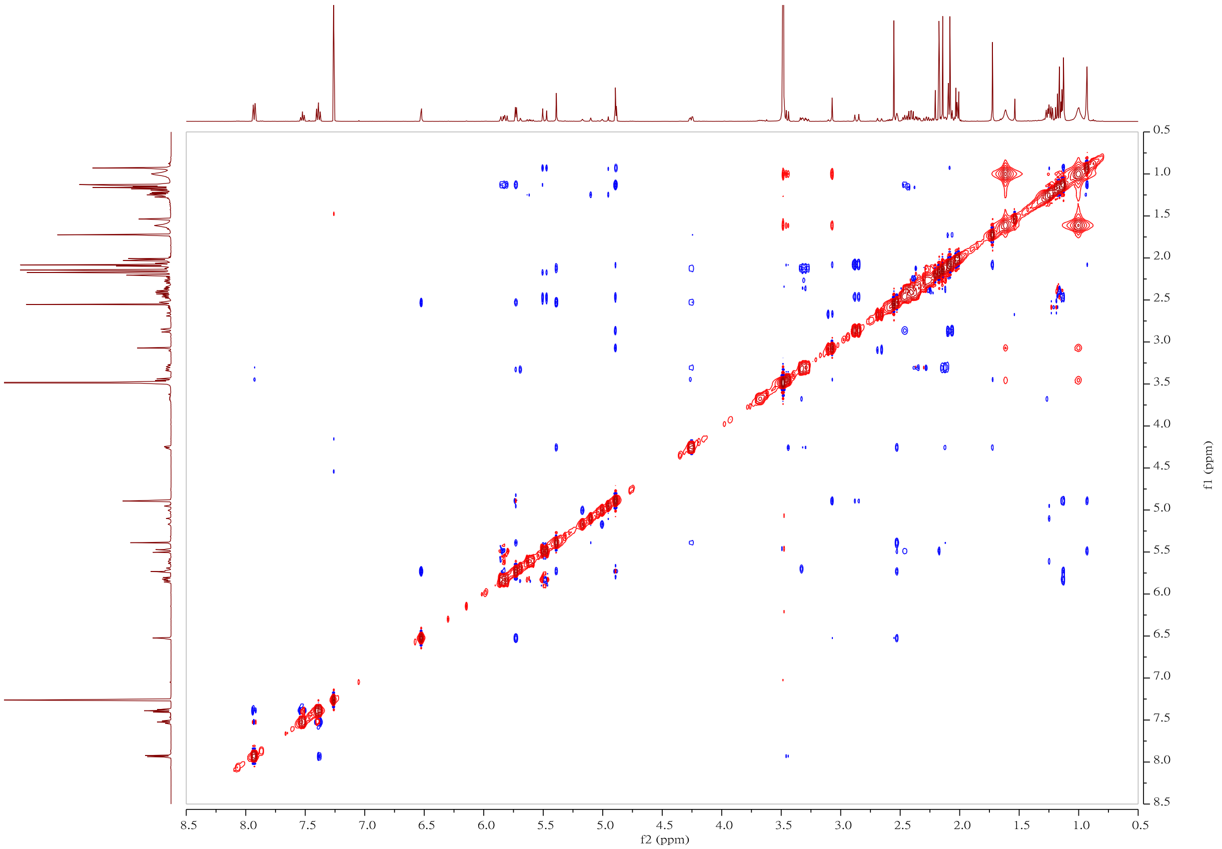

Figure S13. The NOESY spectrum of usambariphane A (**2**).

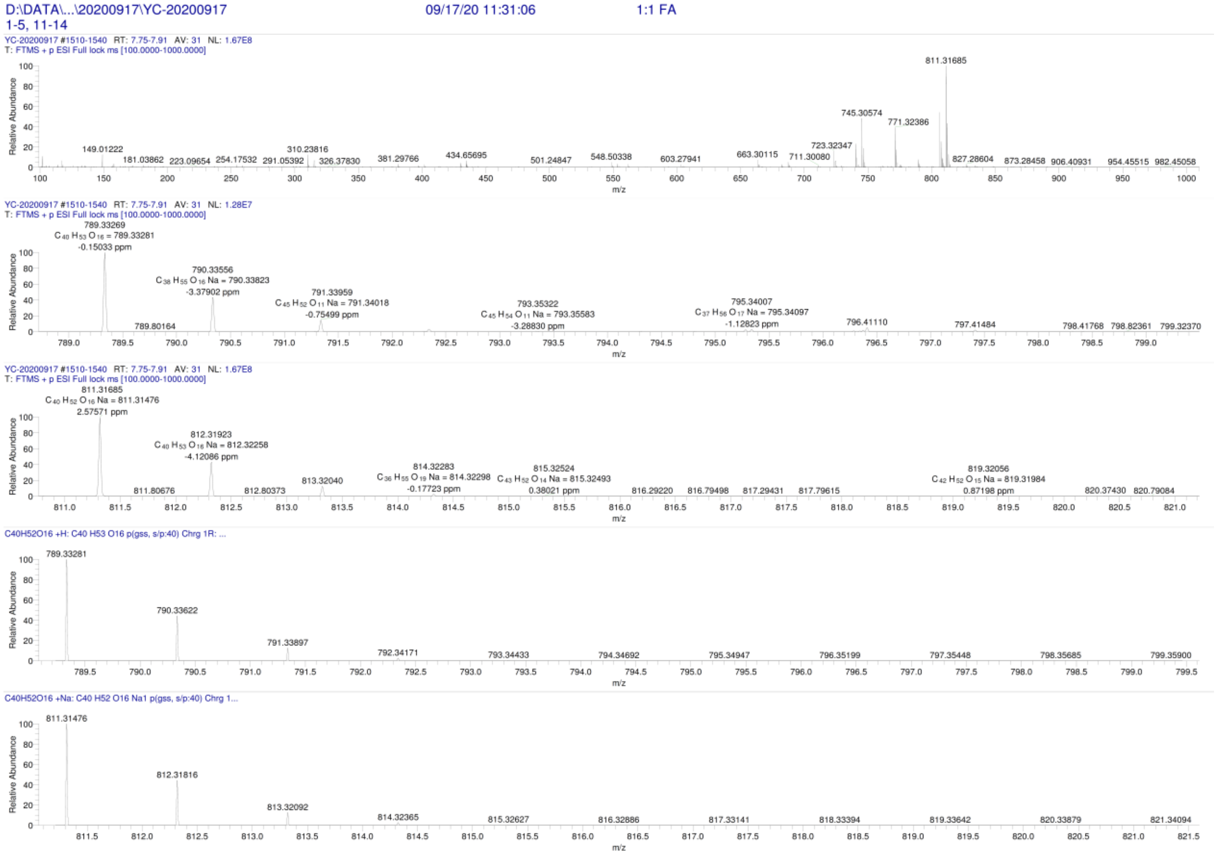

Figure S14. The HR-ESIMS spectra of usambariphane A (**2**).

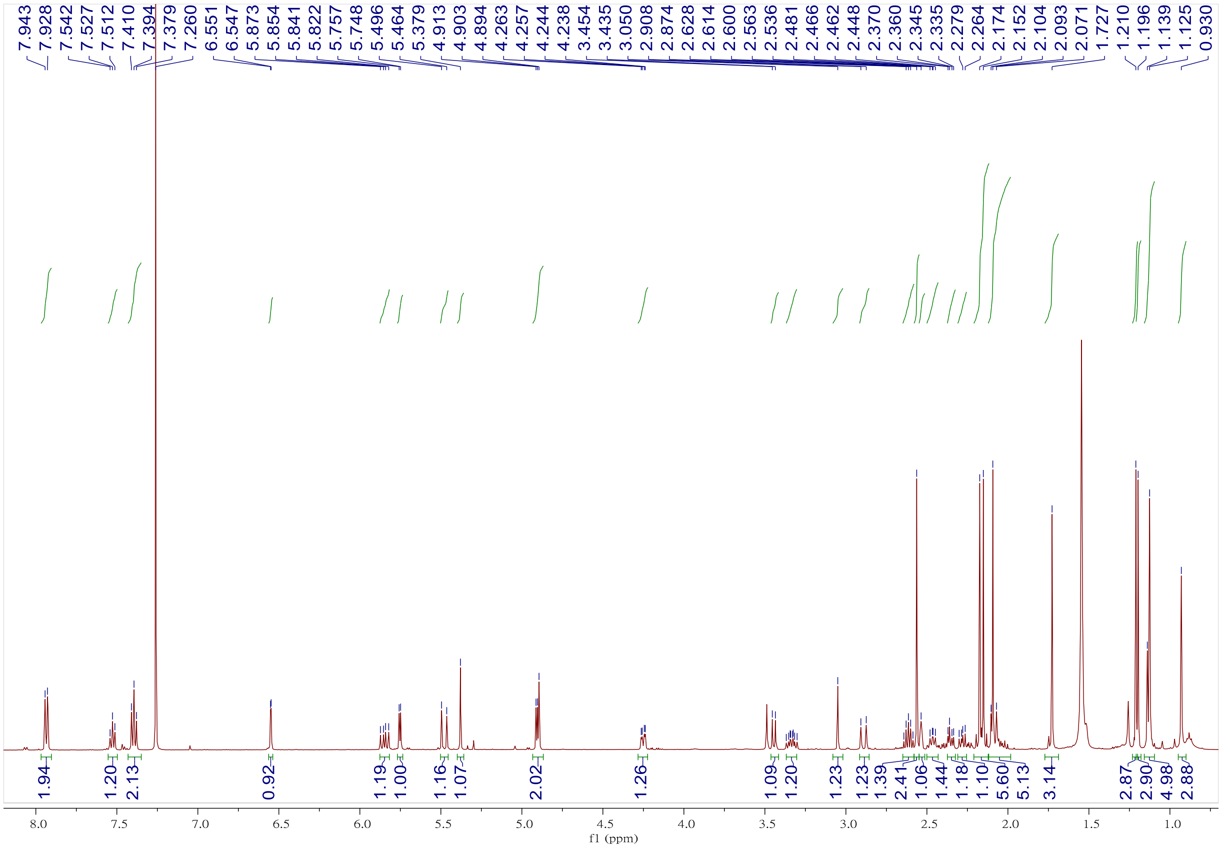

Figure S15. The ^{1}H-NMR spectrum of usambariphane B (**3**) (500 MHz, $CDCl_3$).

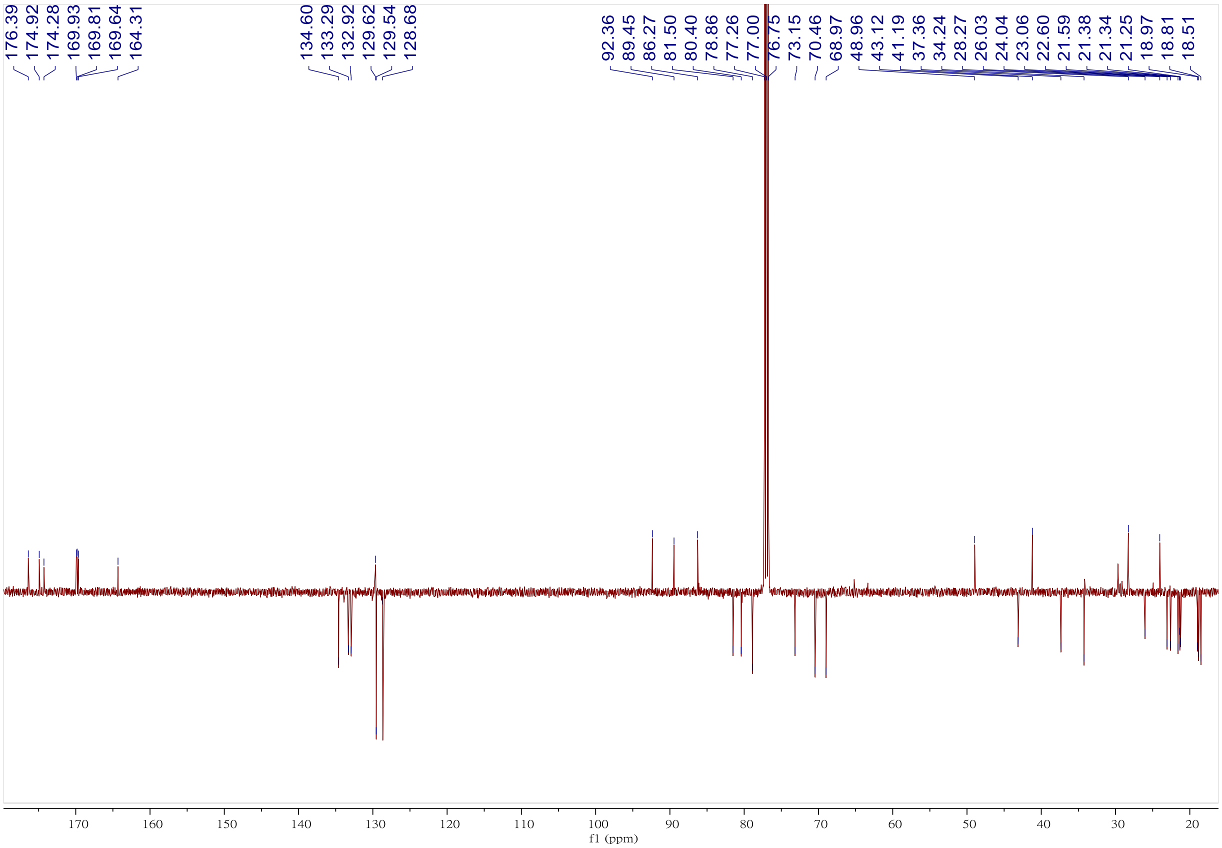

Figure S16. The ^{13}C-JMOD spectrum of usambariphane B (**3**) (125 MHz, $CDCl_3$).

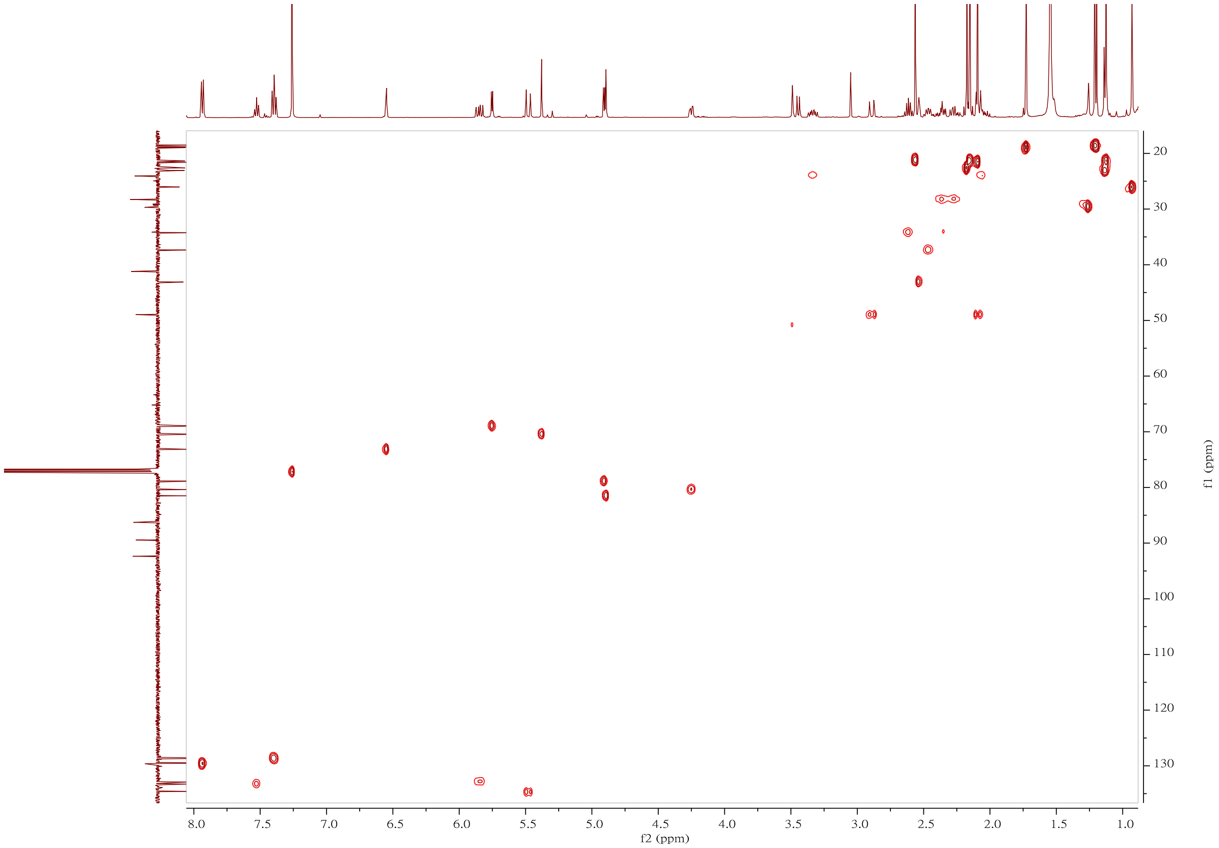

Figure S17. The HSQC spectrum of usambariphane B (**3**).

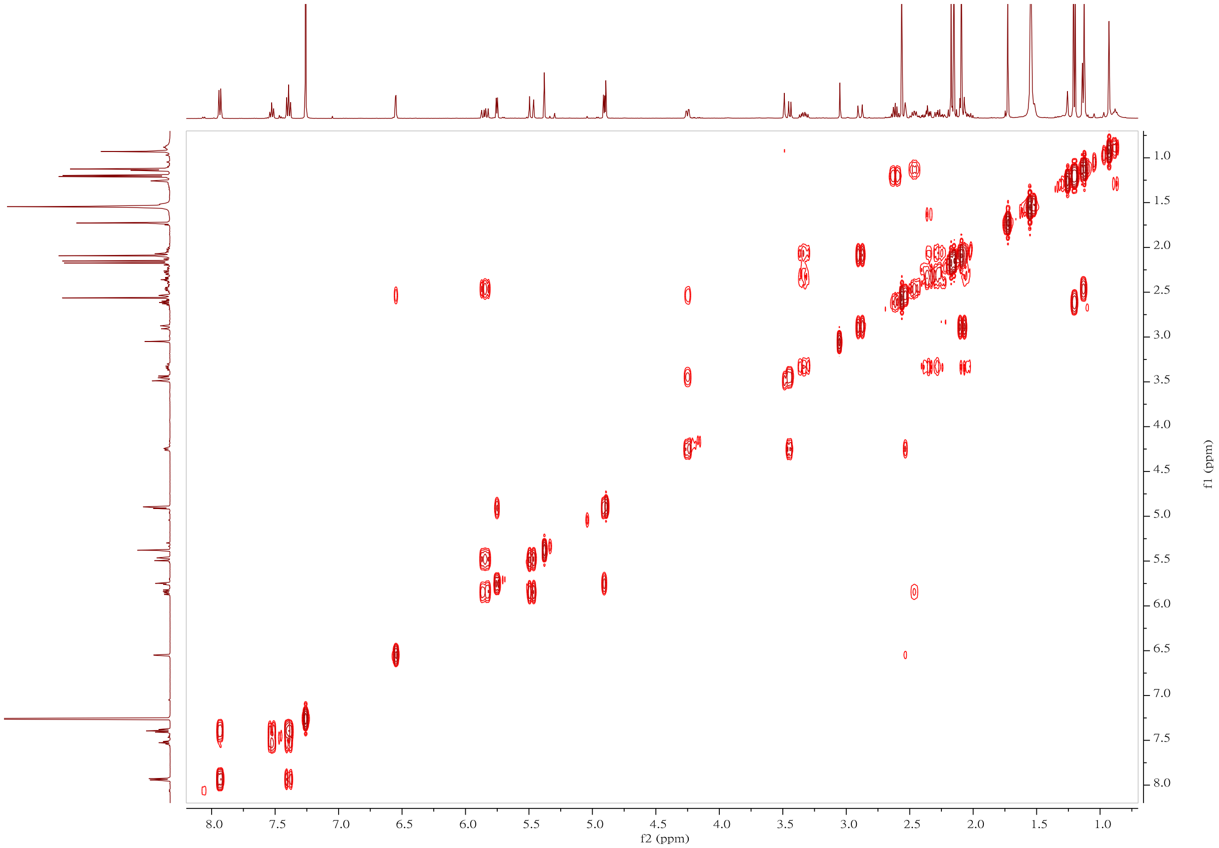

Figure S18. The ^{1}H-^{1}H COSY spectrum of usambariphane B (**3**).

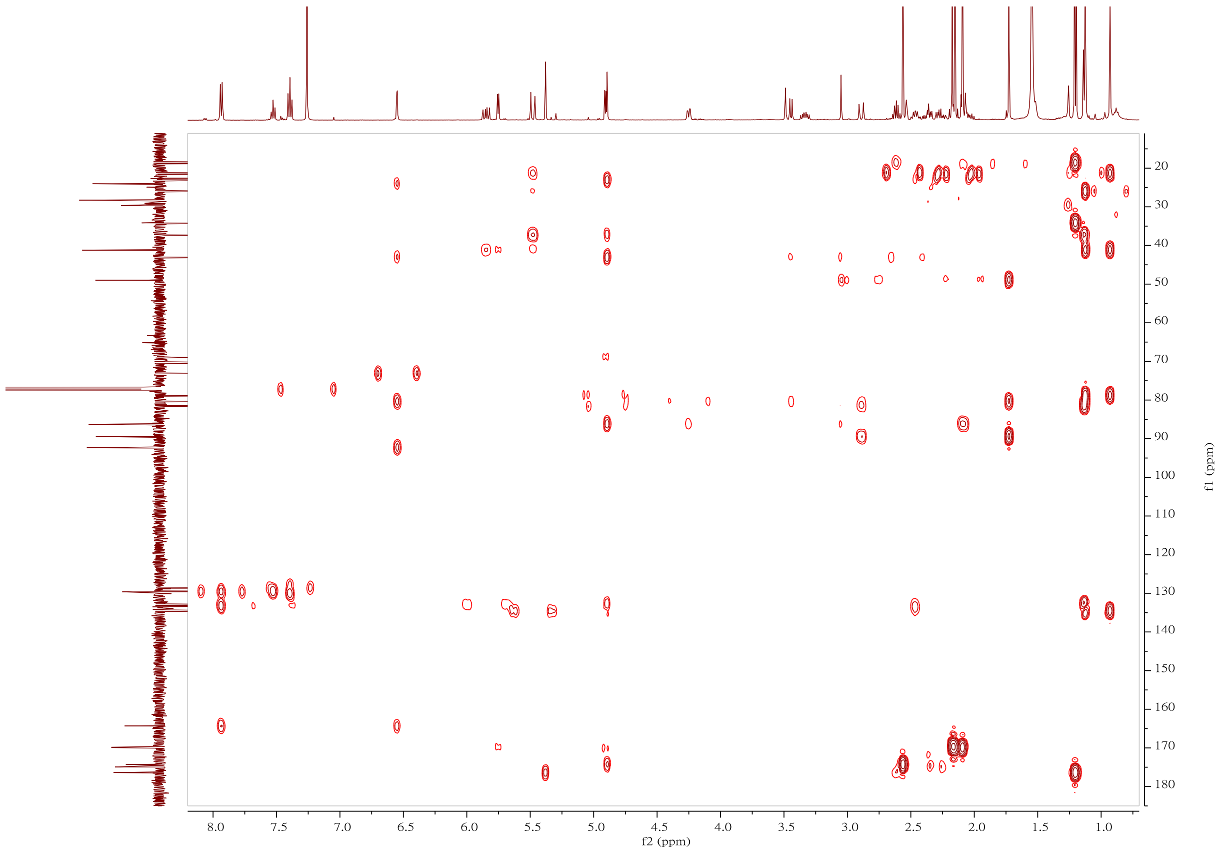

Figure S19. The HMBC spectrum of usambariphane B (**3**).

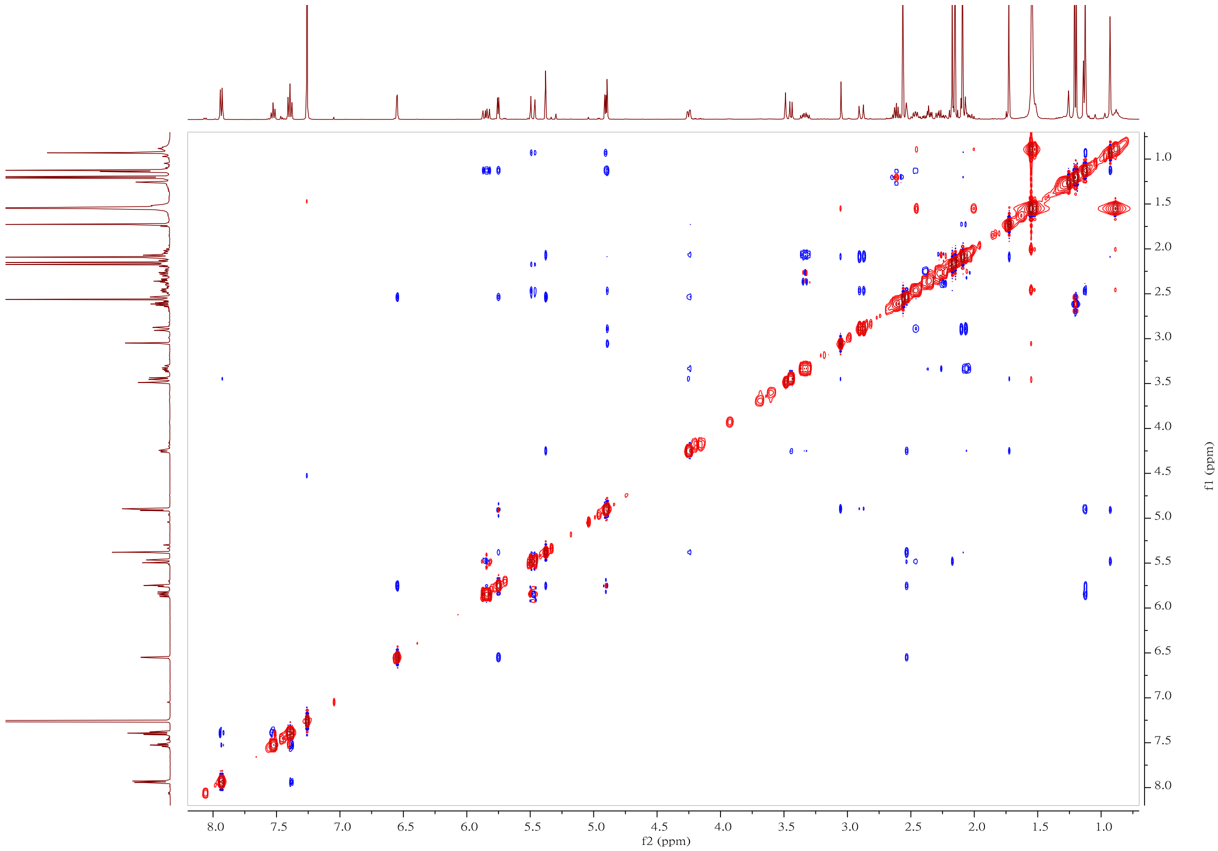

Figure S20. The NOESY spectrum of usambariphane B (**3**).

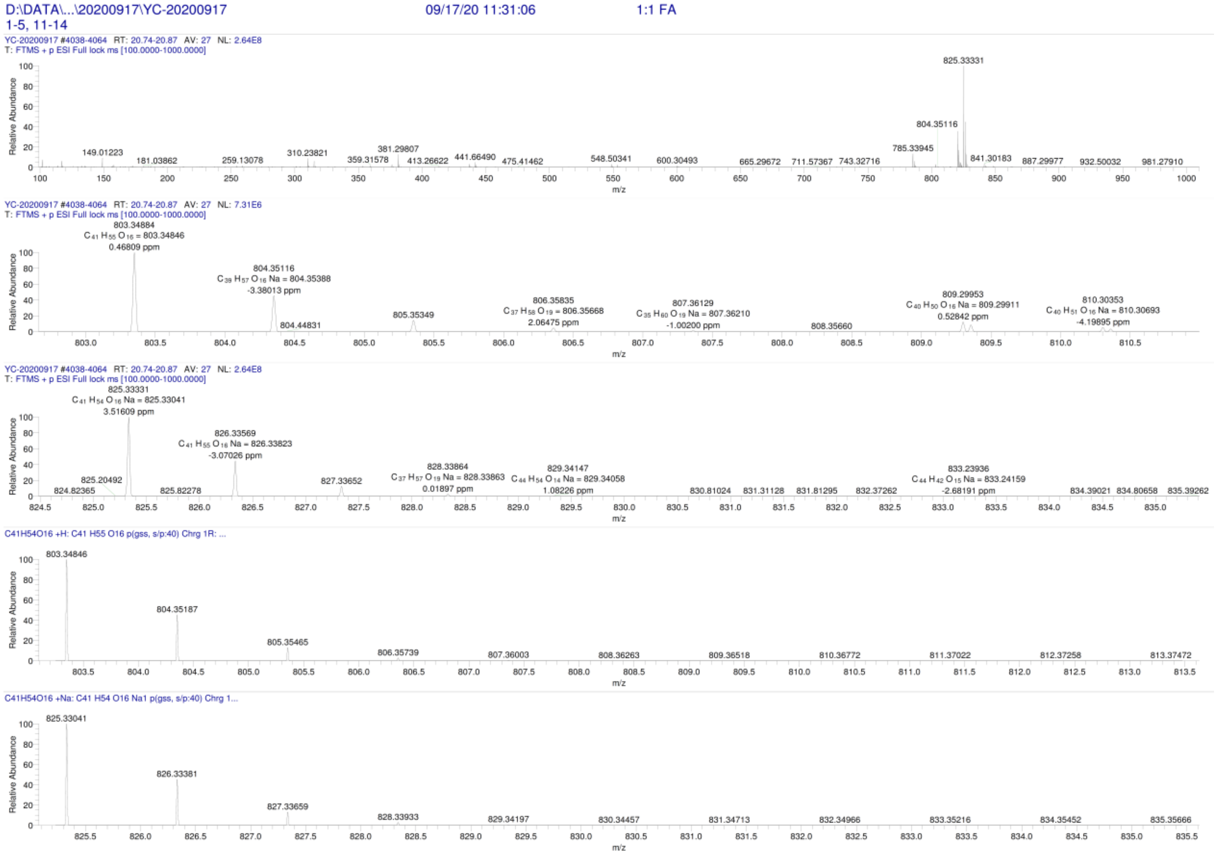

Figure S21. The HR-ESIMS spectra of usambariphane B (**3**).

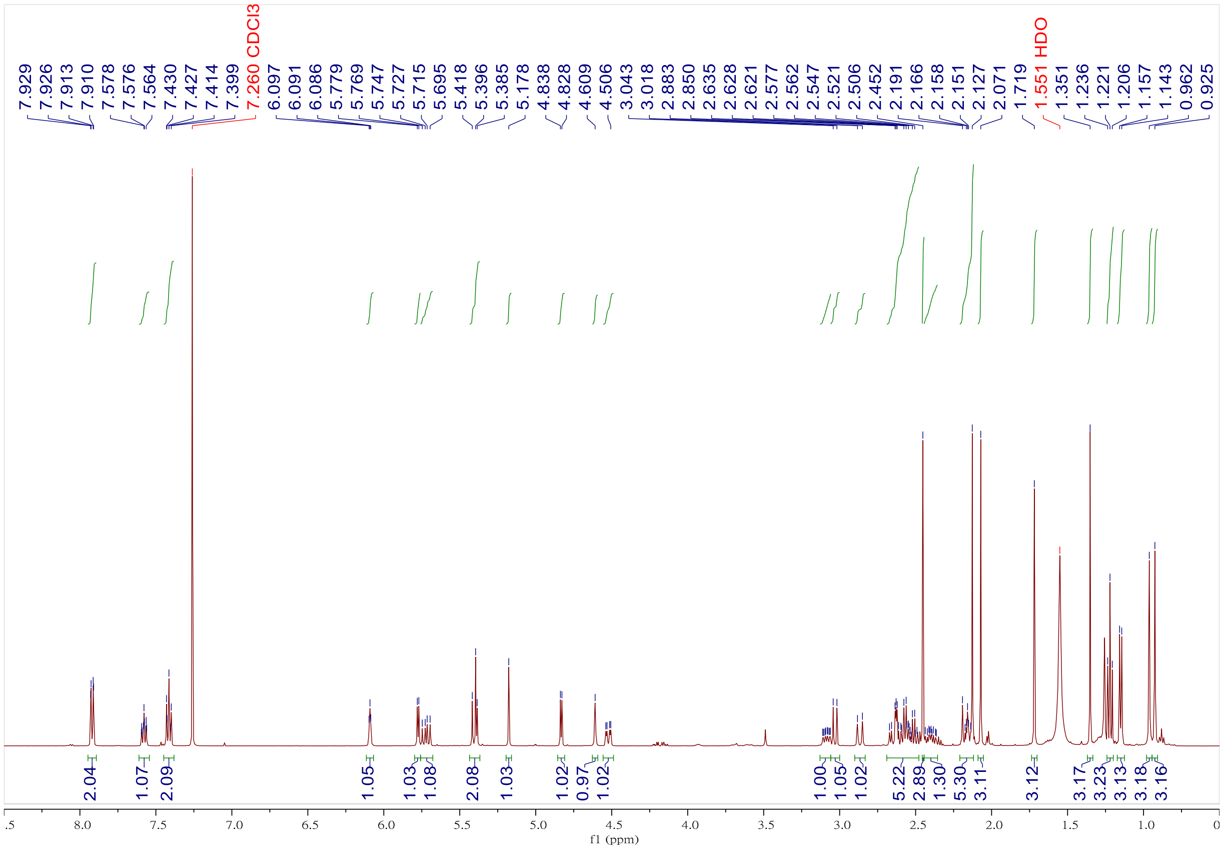

Figure S22. The ^{1}H-NMR spectrum of usambariphane C (**4**) (500 MHz, $CDCl_3$).

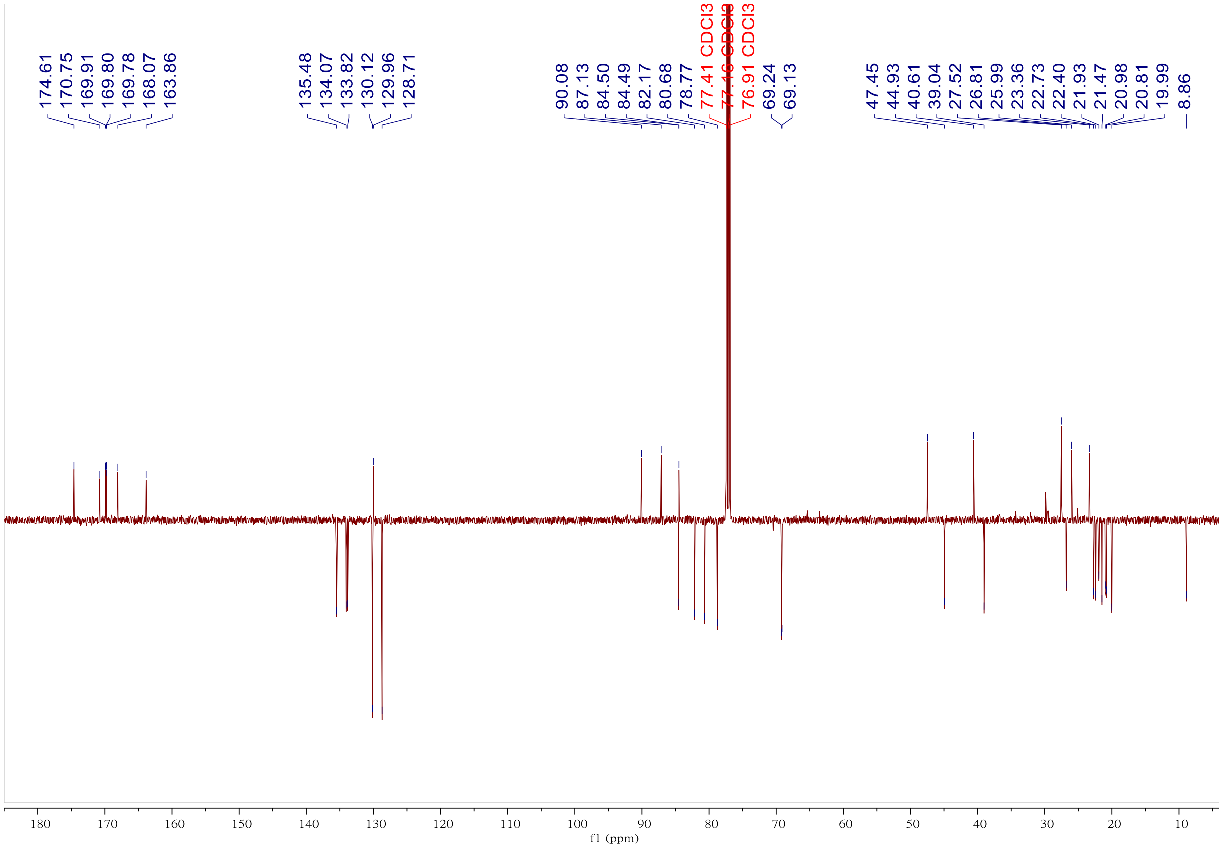

Figure S23. The ^{13}C-JMOD spectrum of usambariphane C (**4**) (125 MHz, $CDCl_3$).

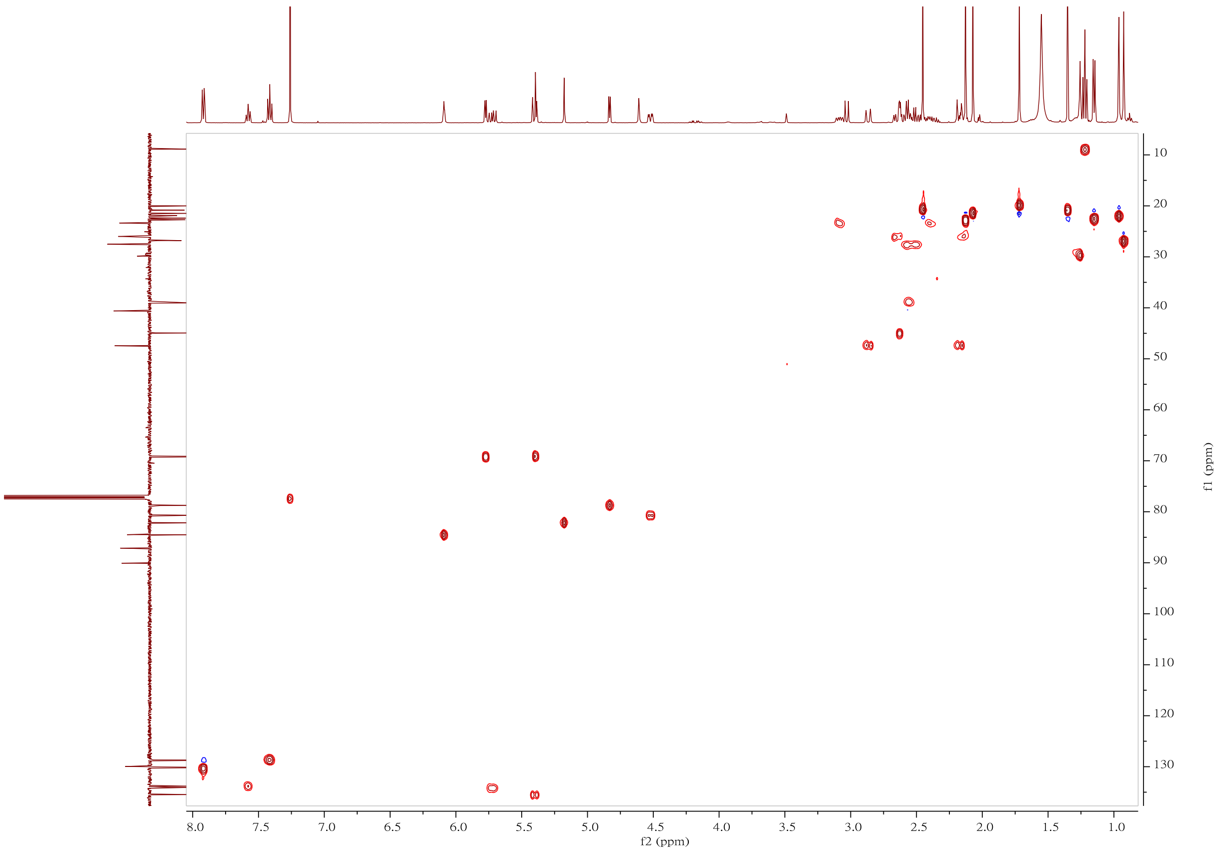

Figure S24. The HSQC spectrum of usambariphane C (**4**).

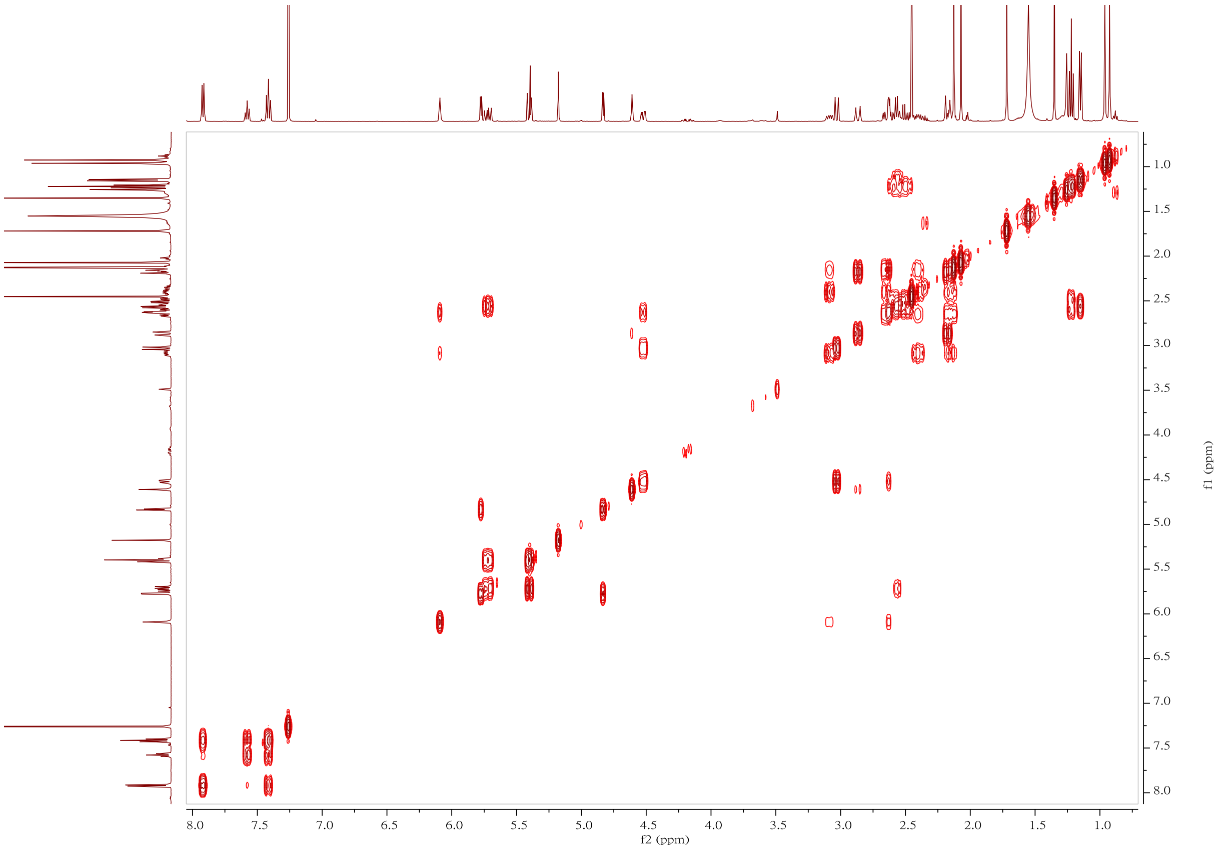

Figure S25. The ${}^{1}H$-${}^{1}H$ COSY spectrum of usambariphane C (**4**).

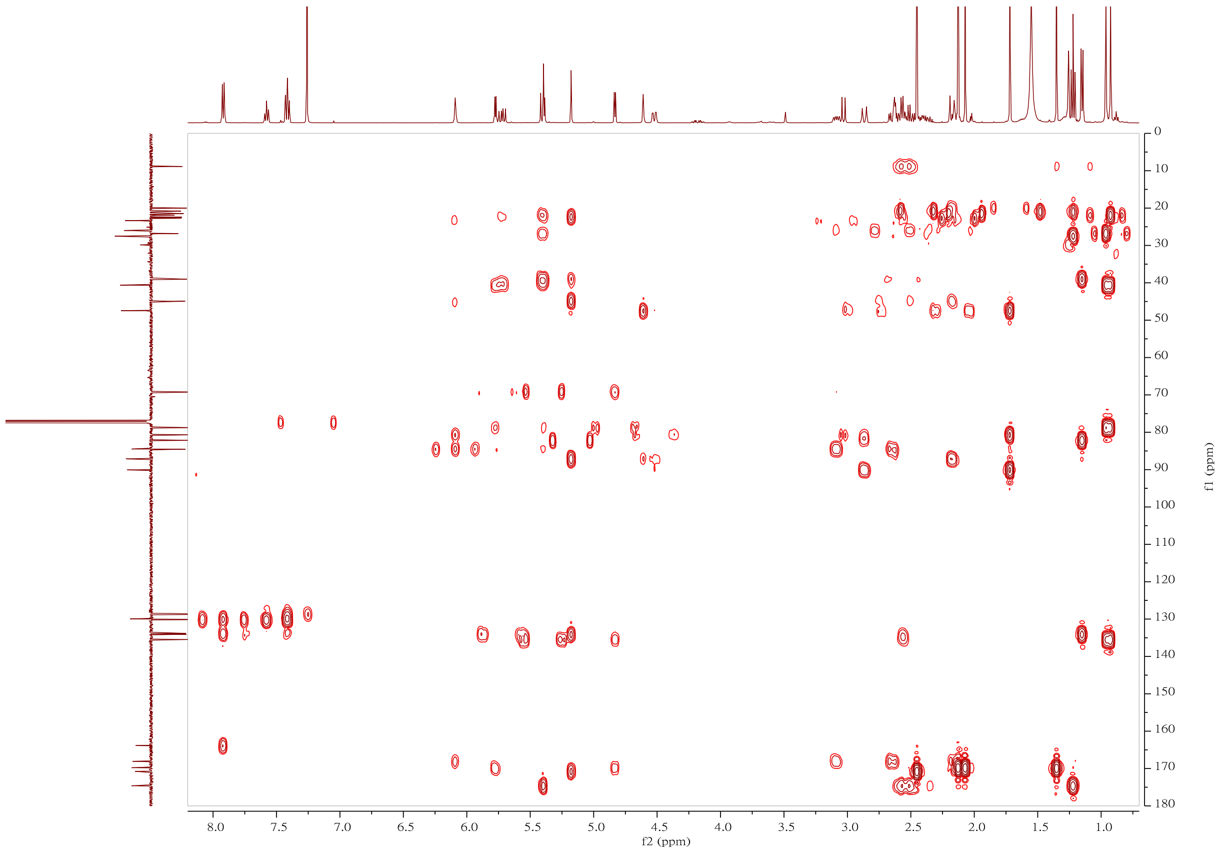

Figure S26. The HMBC spectrum of usambariphane C (**4**).

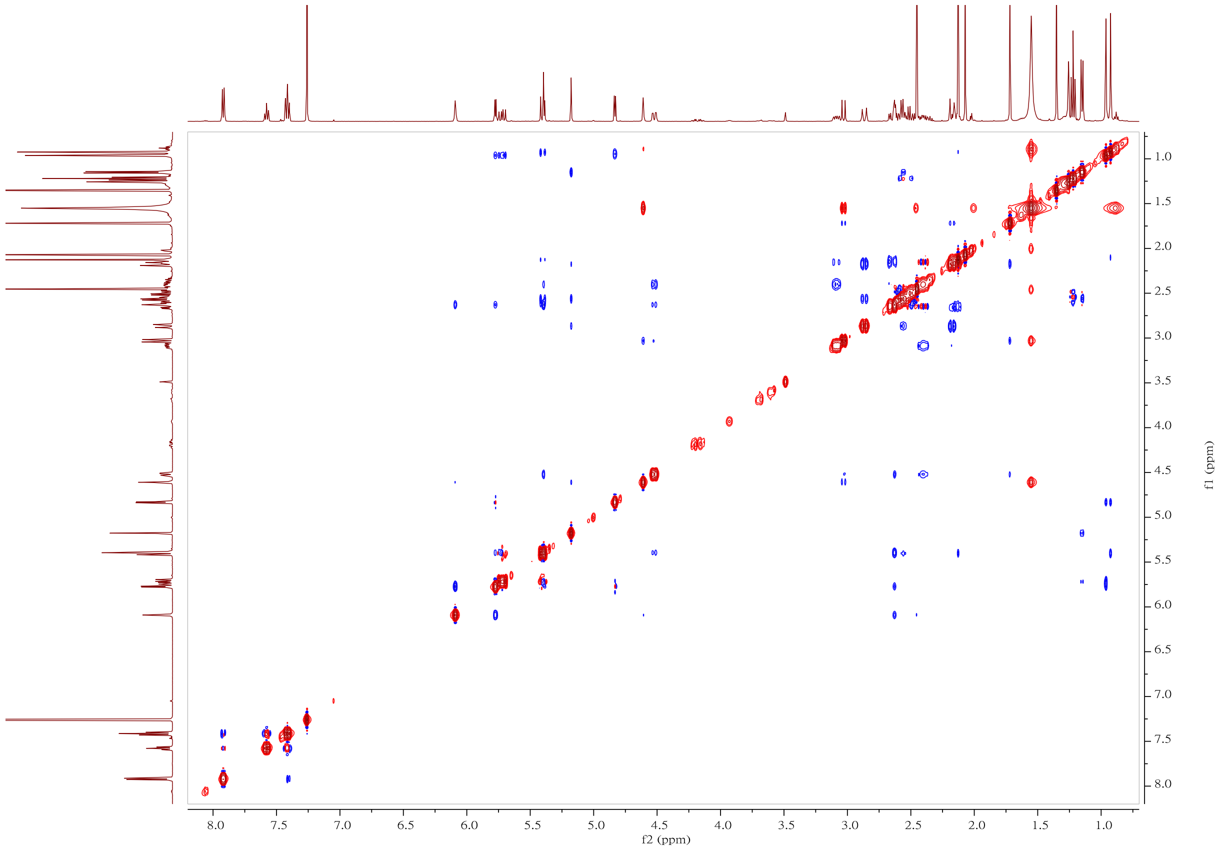

Figure S27. The NOESY spectrum of usambariphane C (**4**).

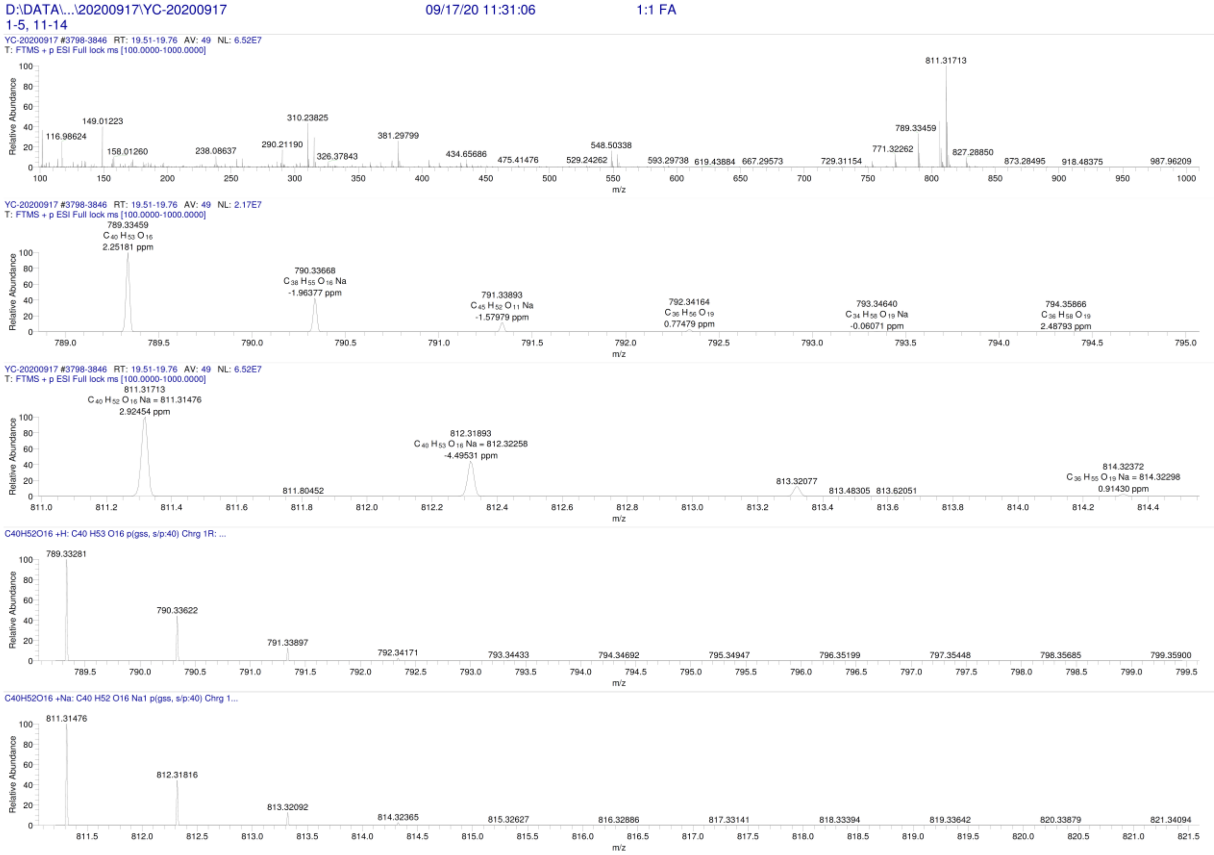

Figure S28. The HR-ESIMS spectra of usambariphane C (**4**).

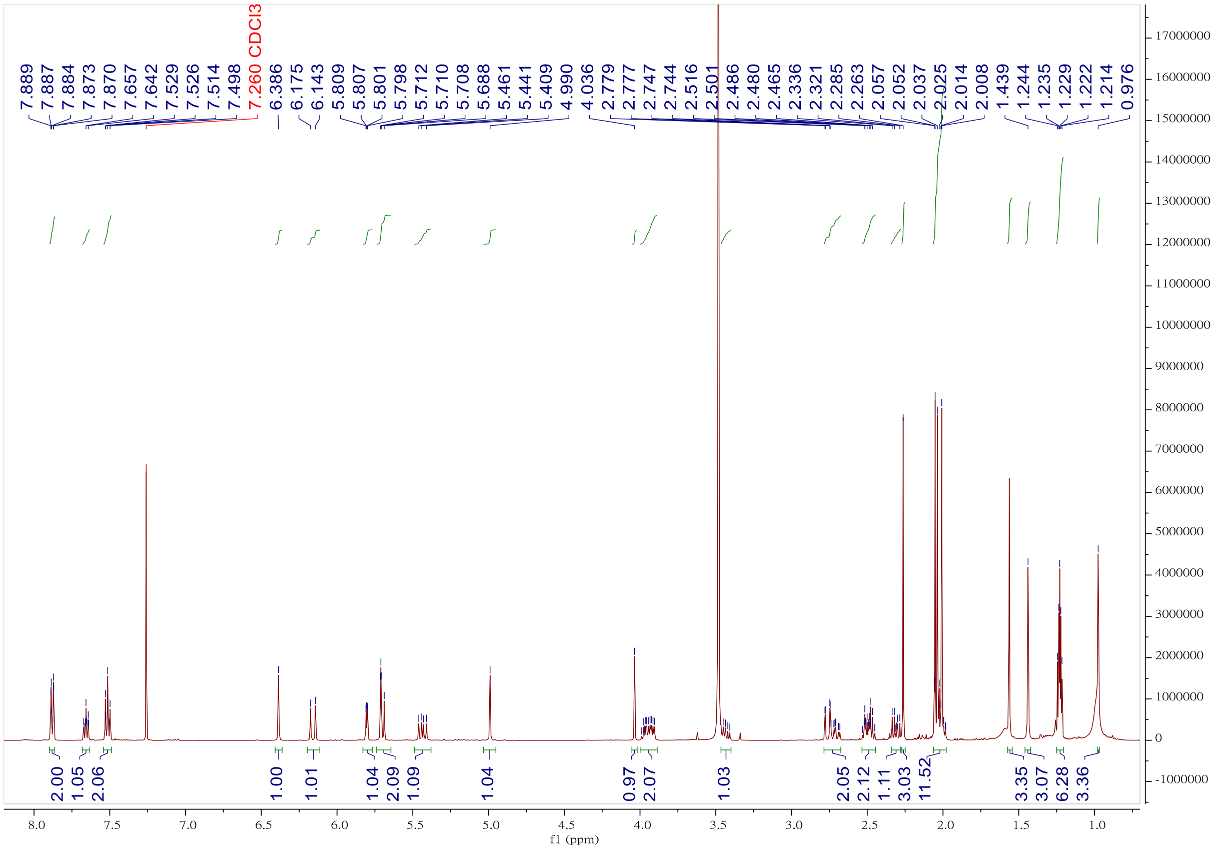

Figure S29. The ^{1}H-NMR spectrum of usambariphane D (**5**) (500 MHz, $CDCl_3$).

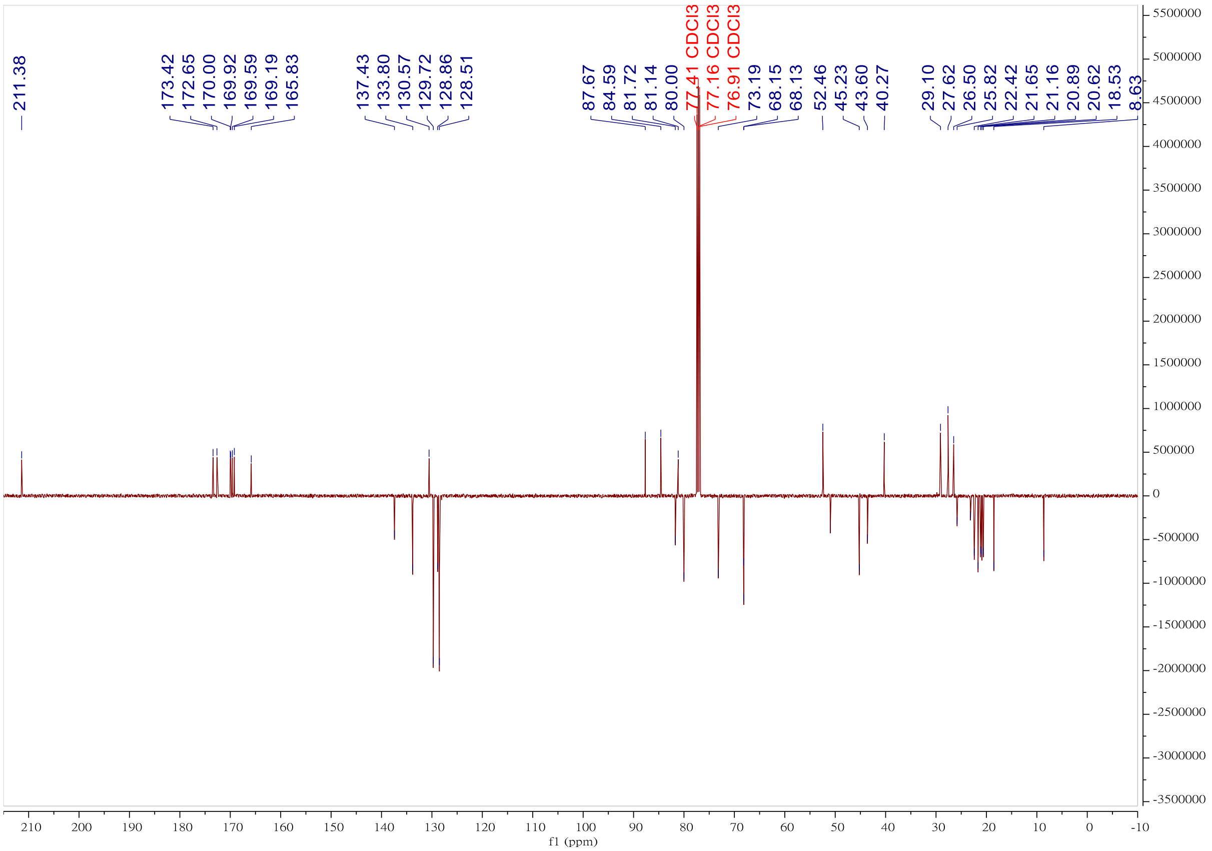

Figure S30. The ^{13}C-JMOD spectrum of usambariphane D (**5**) (125 MHz, $CDCl_3$).

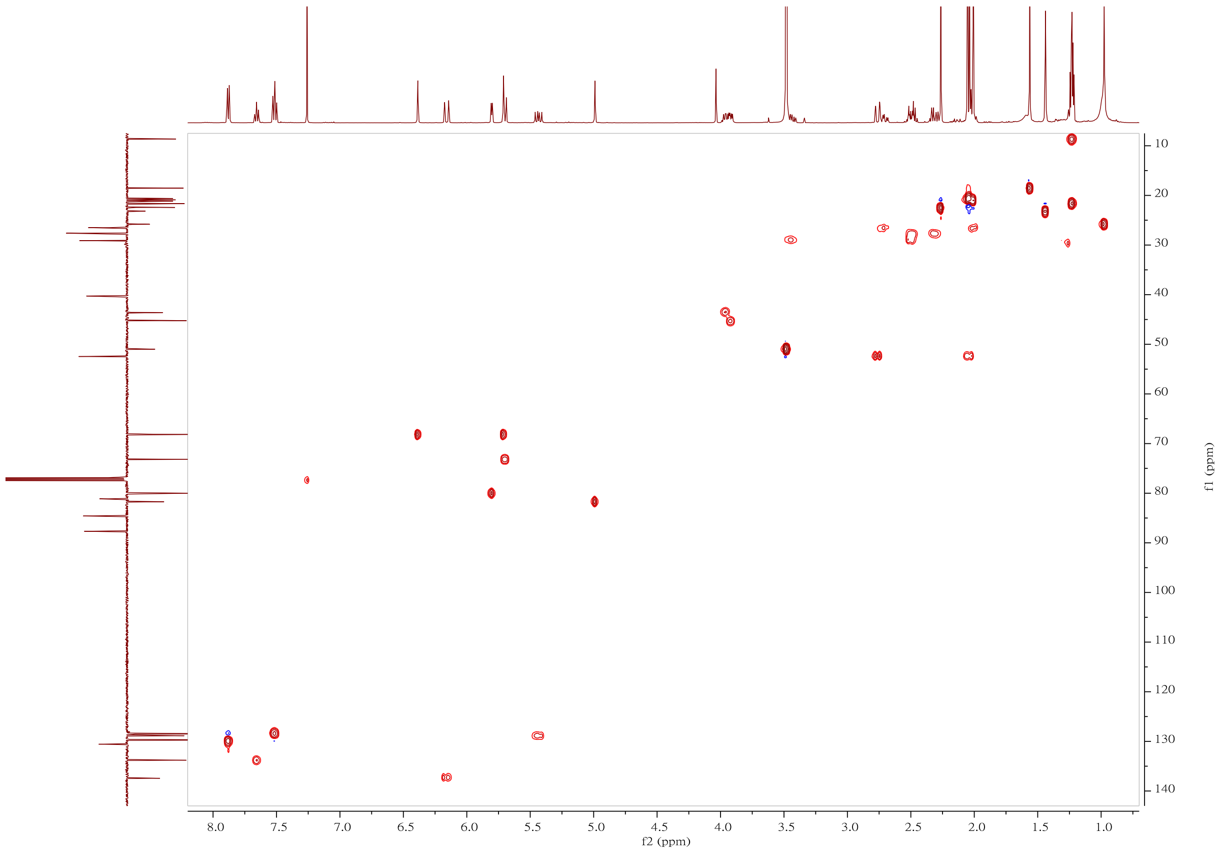

Figure S31. The HSQC spectrum of usambariphane D (**5**).

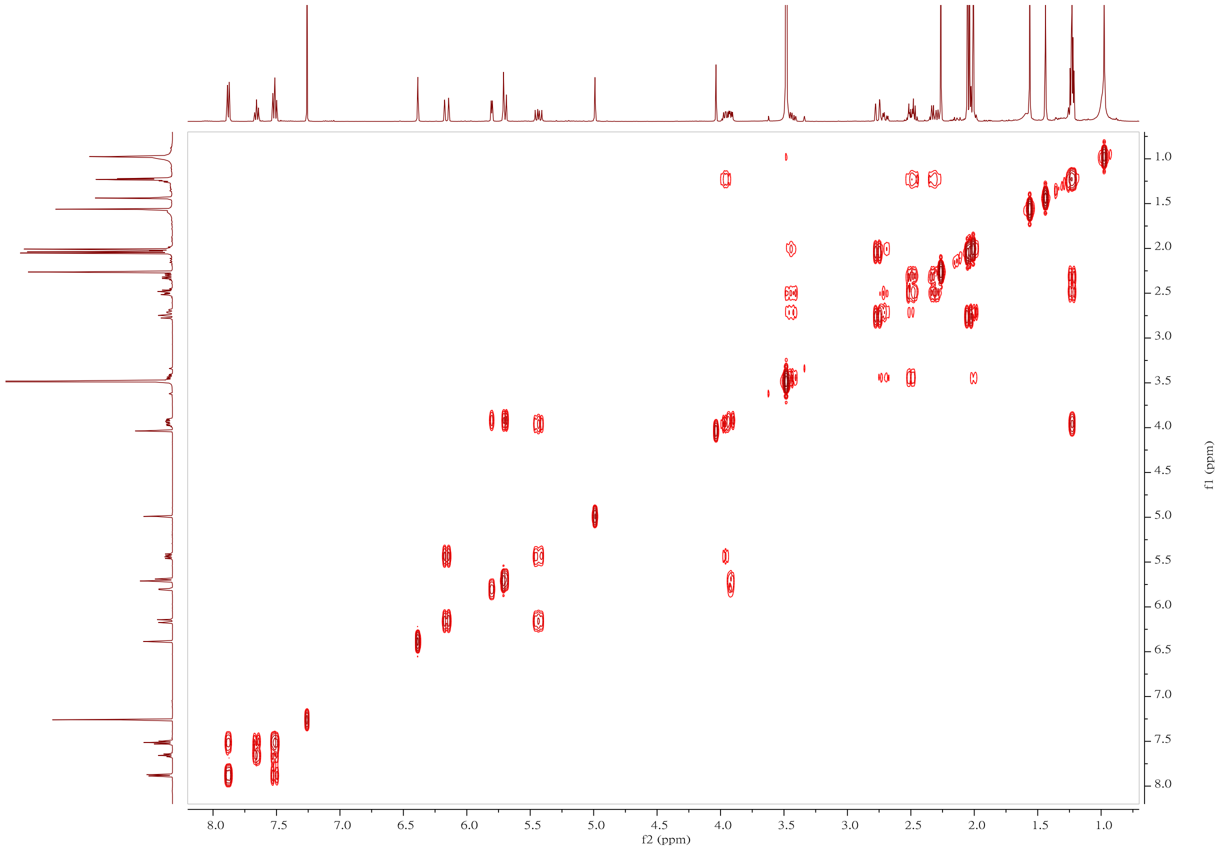

Figure S32. The ^{1}H-^{1}H COSY spectrum of usambariphane D (**5**).

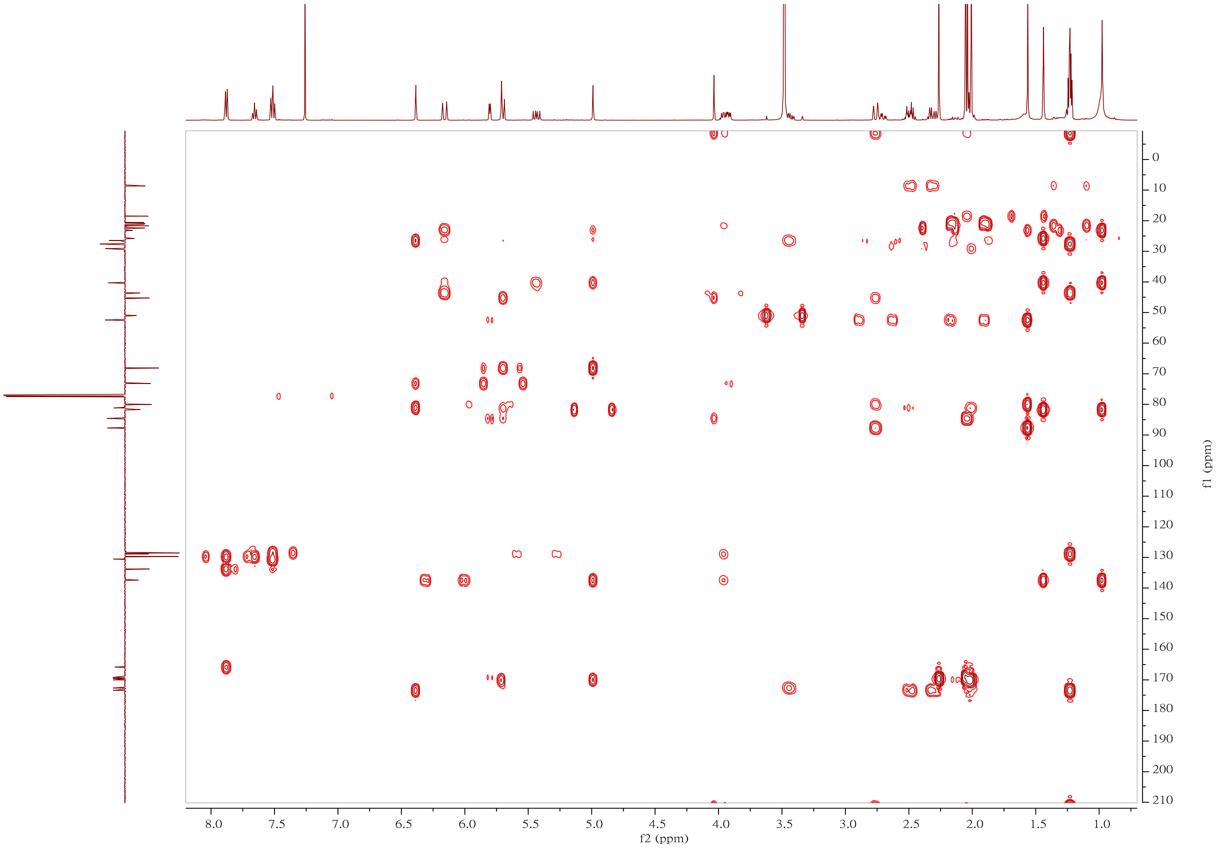

Figure S33. The HMBC spectrum of usambariphane D (**5**).

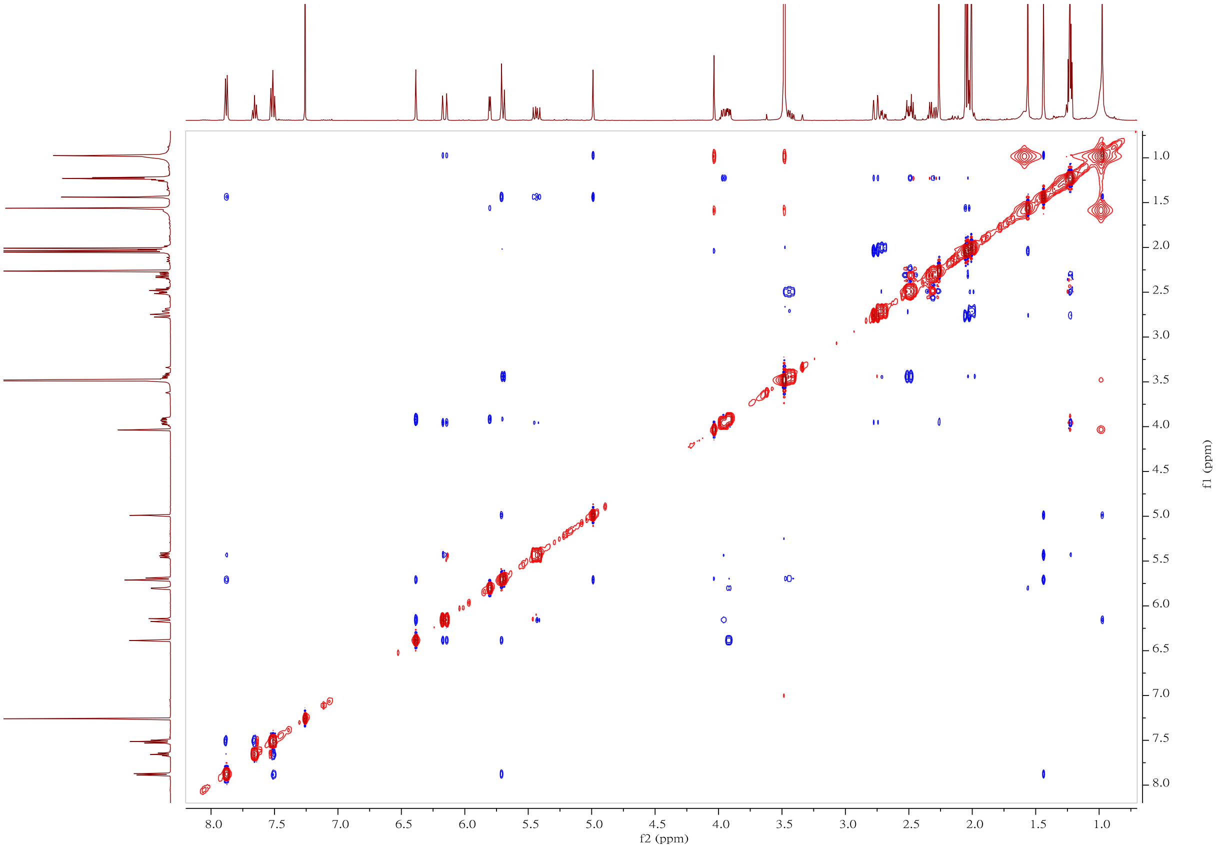

Figure S34. The NOESY spectrum of usambariphane D (**5**).

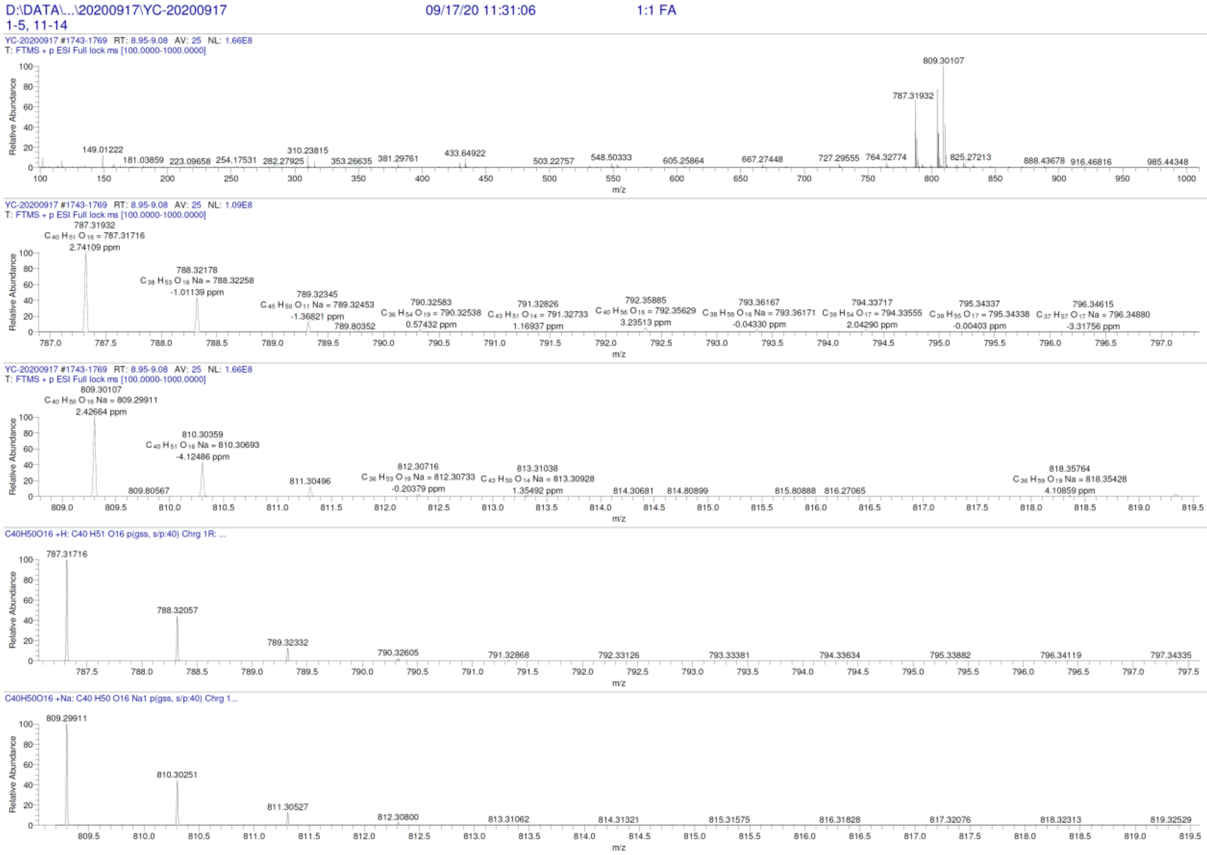

Figure S35. The HR-ESI-MS spectra of usambariphane D (**5**).

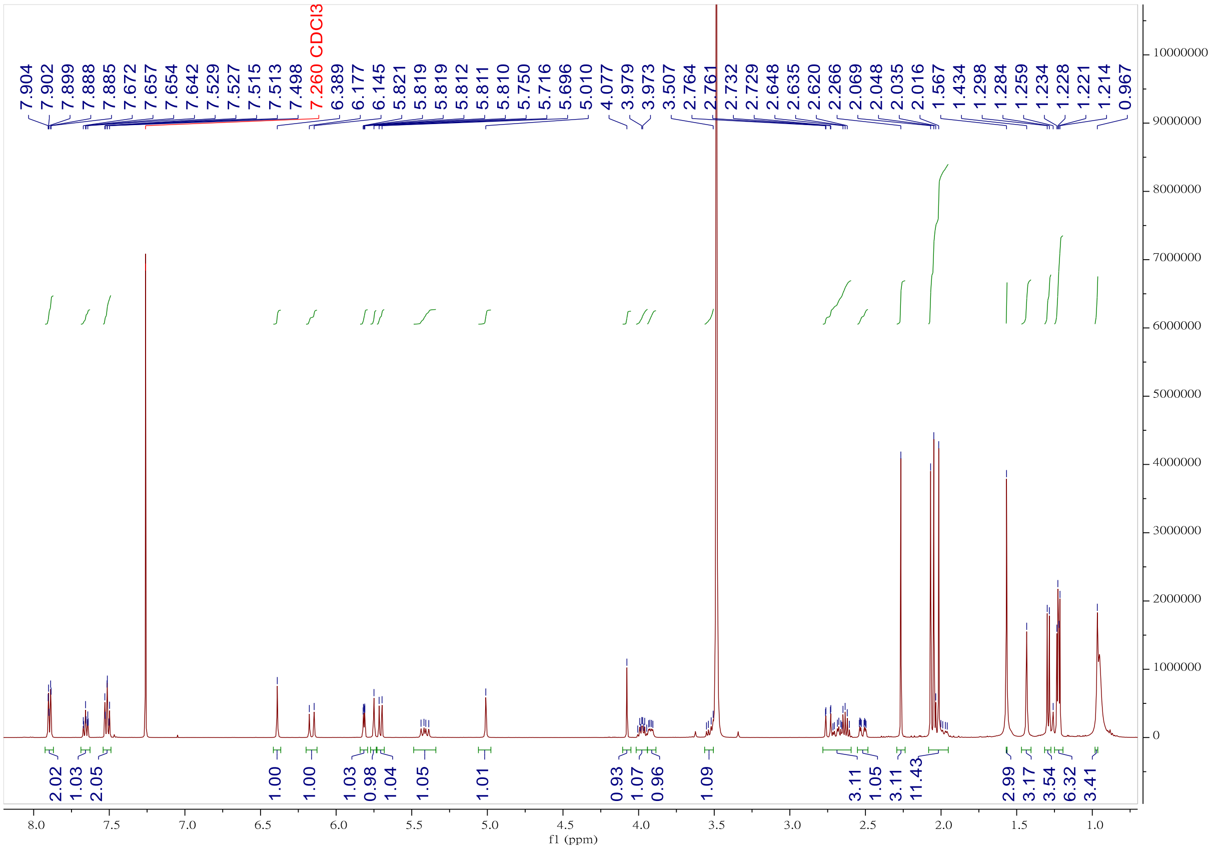

Figure S36. The ^{1}H-NMR spectrum of usambariphane E (**6**) (500 MHz, $CDCl_3$).

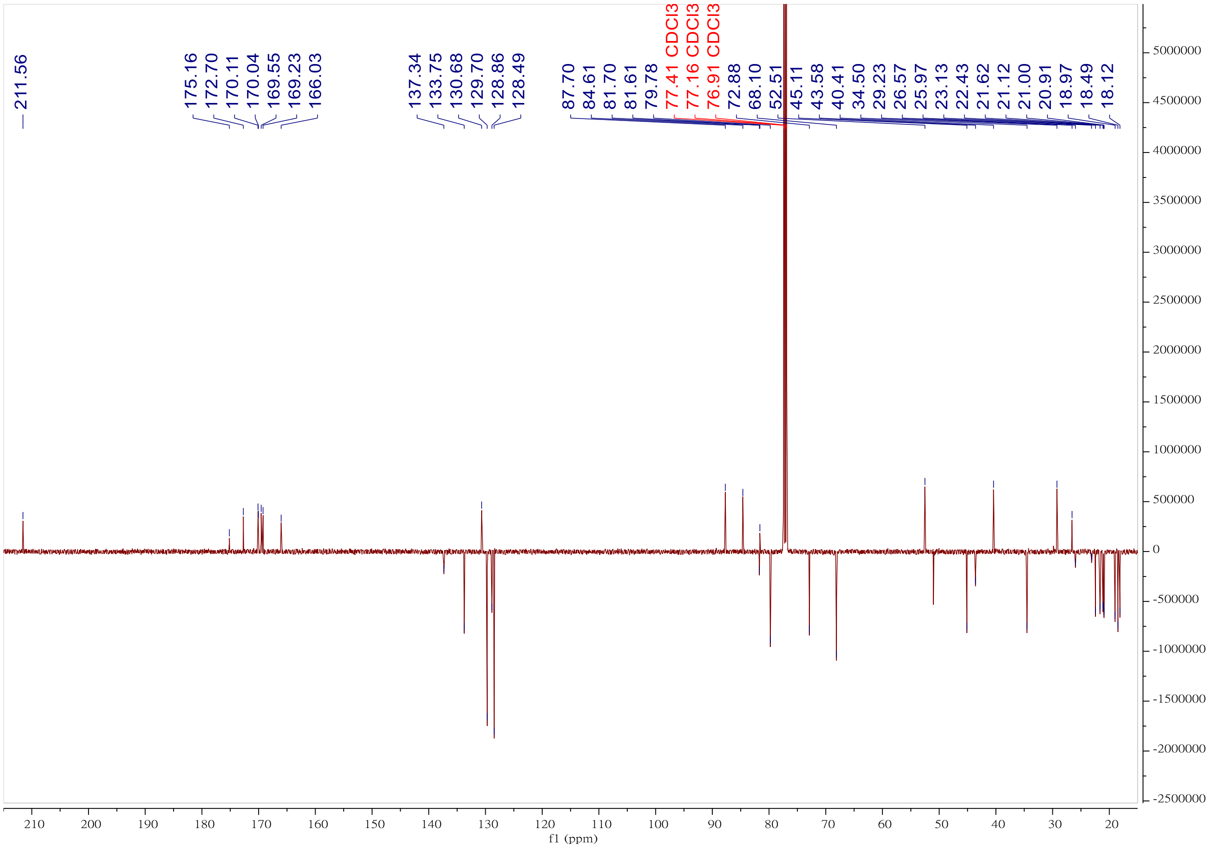

Figure S37. The ^{13}C-JMOD spectrum of usambariphane E (**6**) (125 MHz, $CDCl_3$).

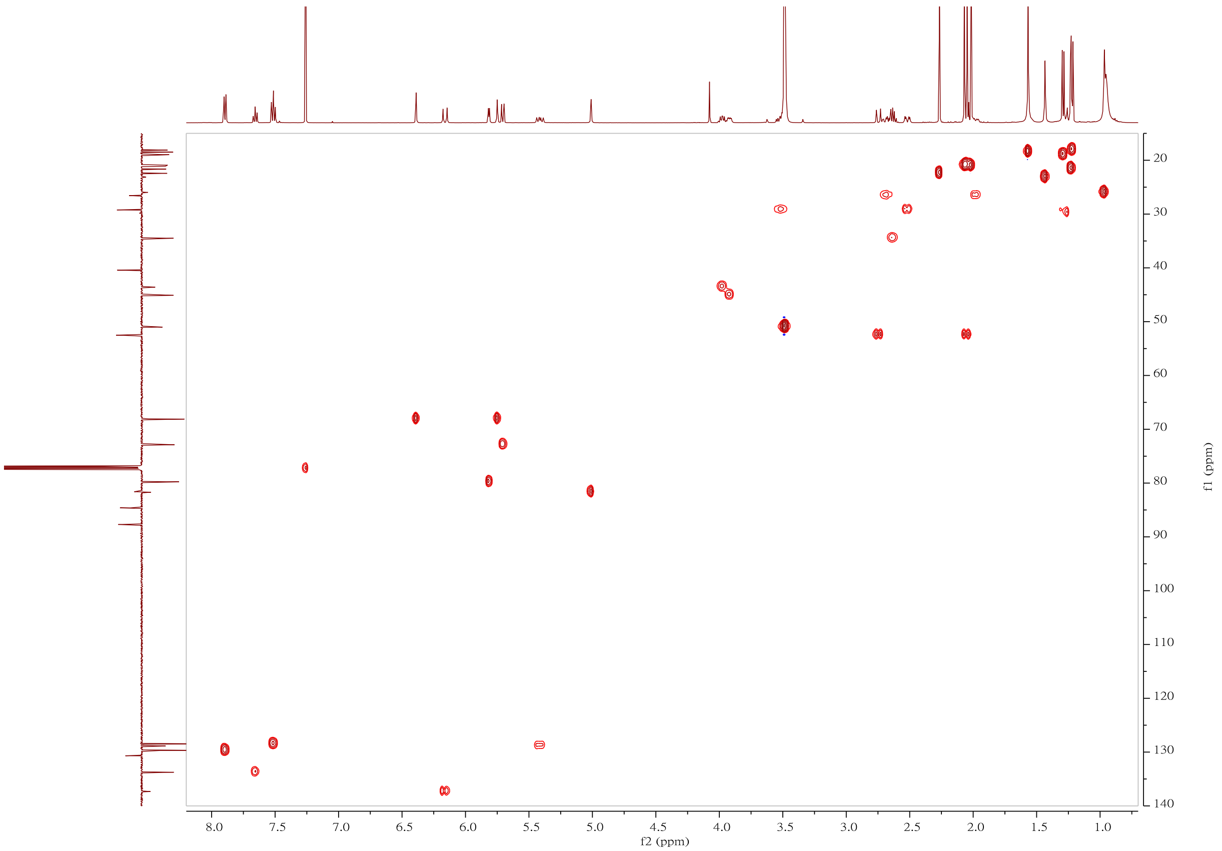

Figure S38. The HSQC spectrum of usambariphane E (**6**).

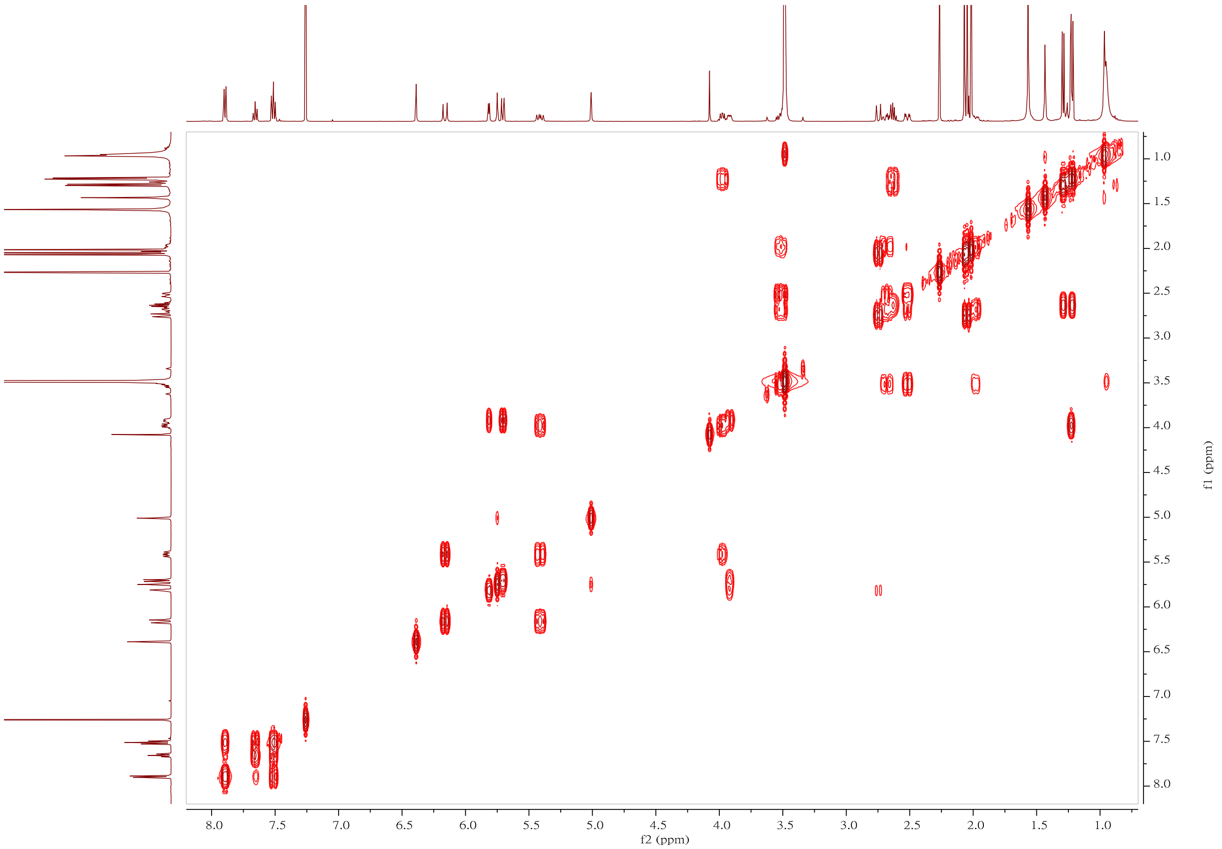

Figure S39. The ¹H-¹H COSY spectrum of usambariphane E (**6**).

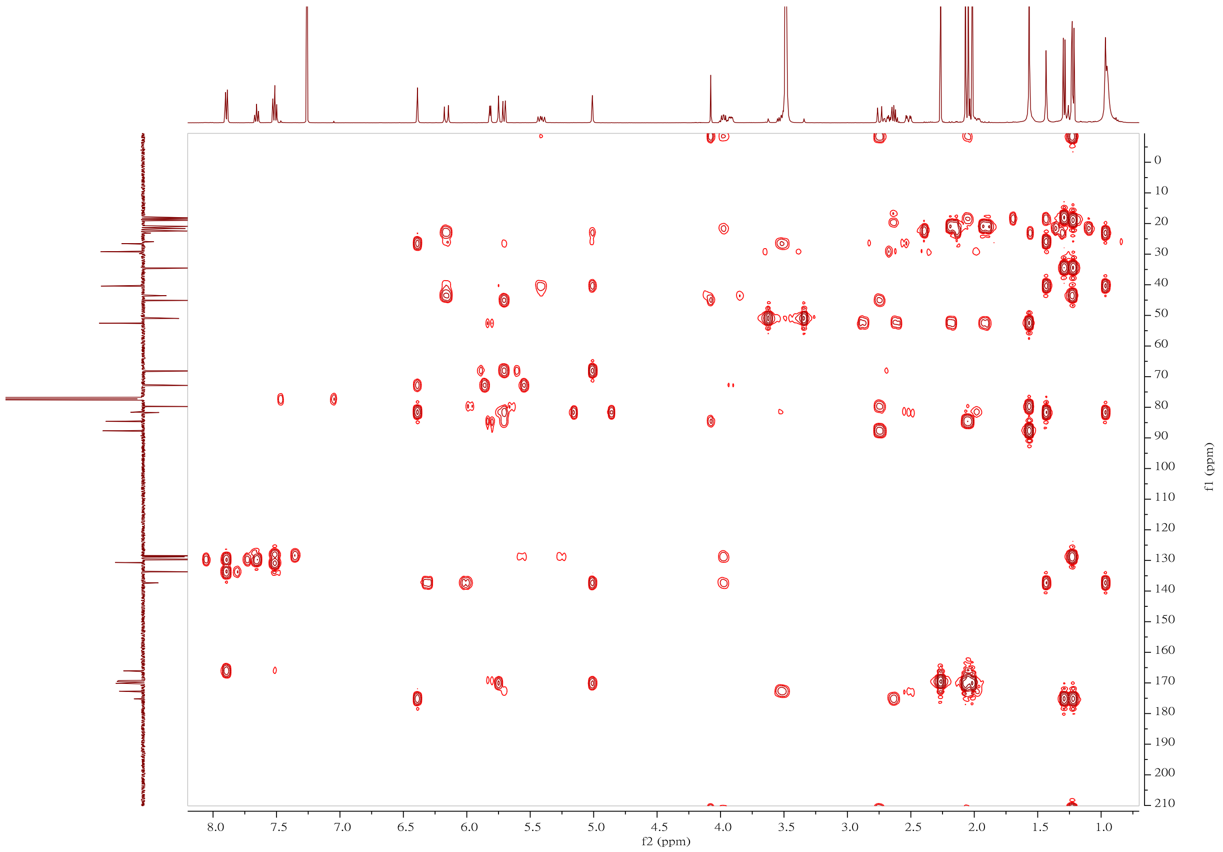

Figure S40. The HMBC spectrum of usambariphane E (**6**).

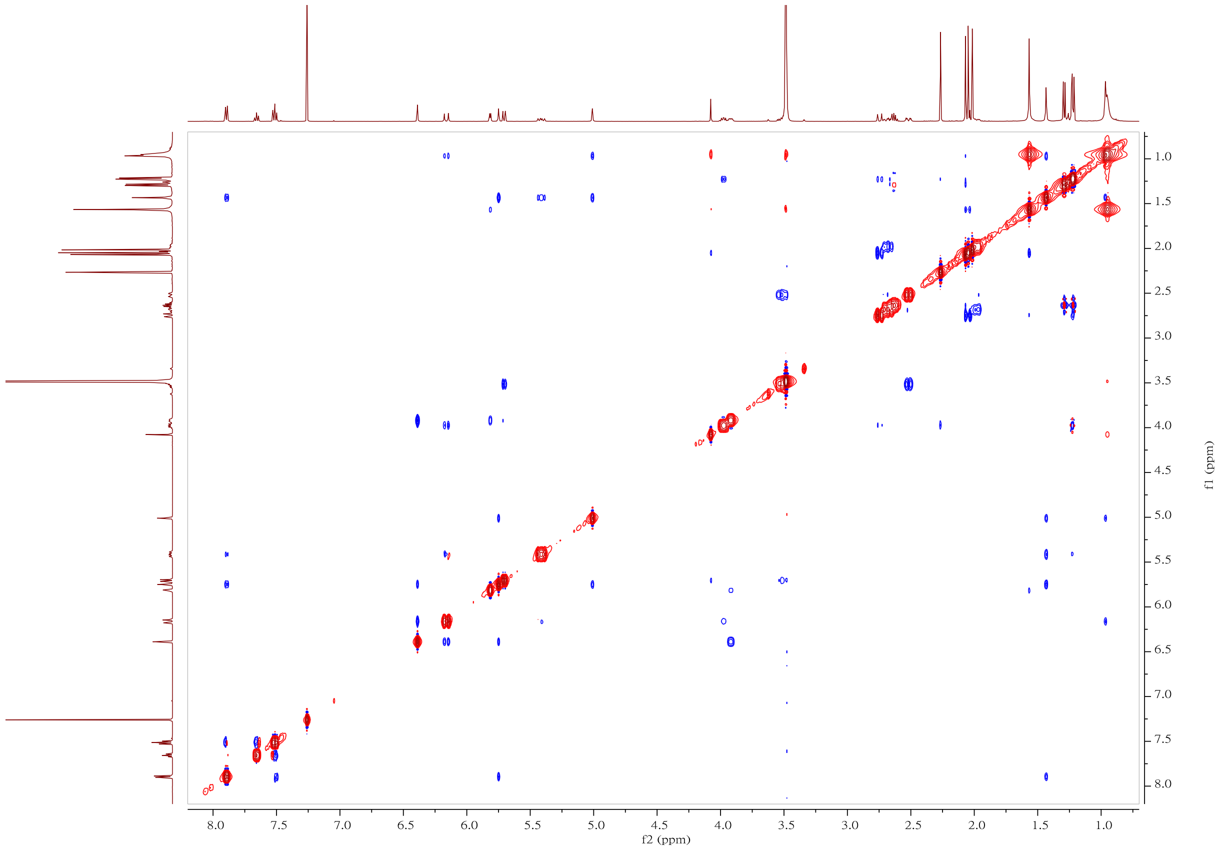

Figure S41. The NOESY spectrum of usambariphane E (**6**).

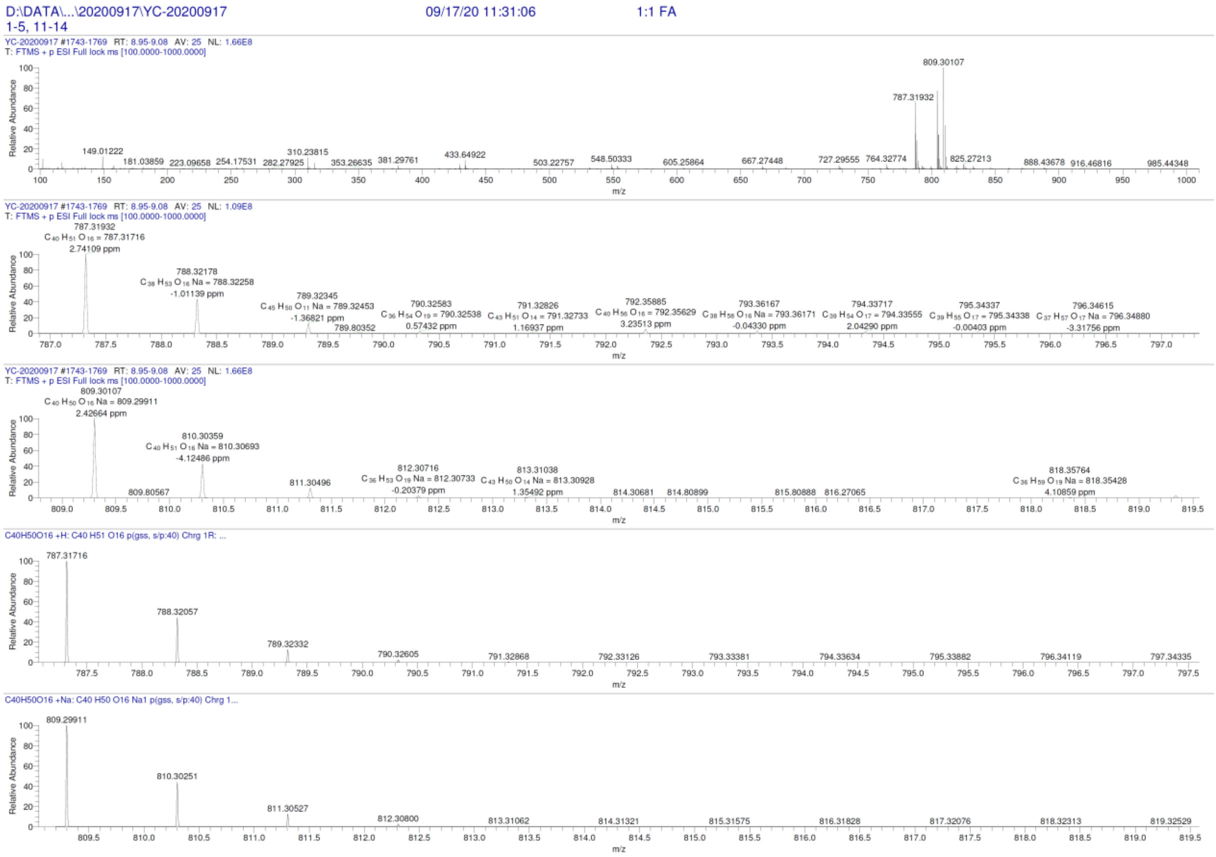

Figure S42. The HR-ESI-MS spectra of usambariphane E (**6**).

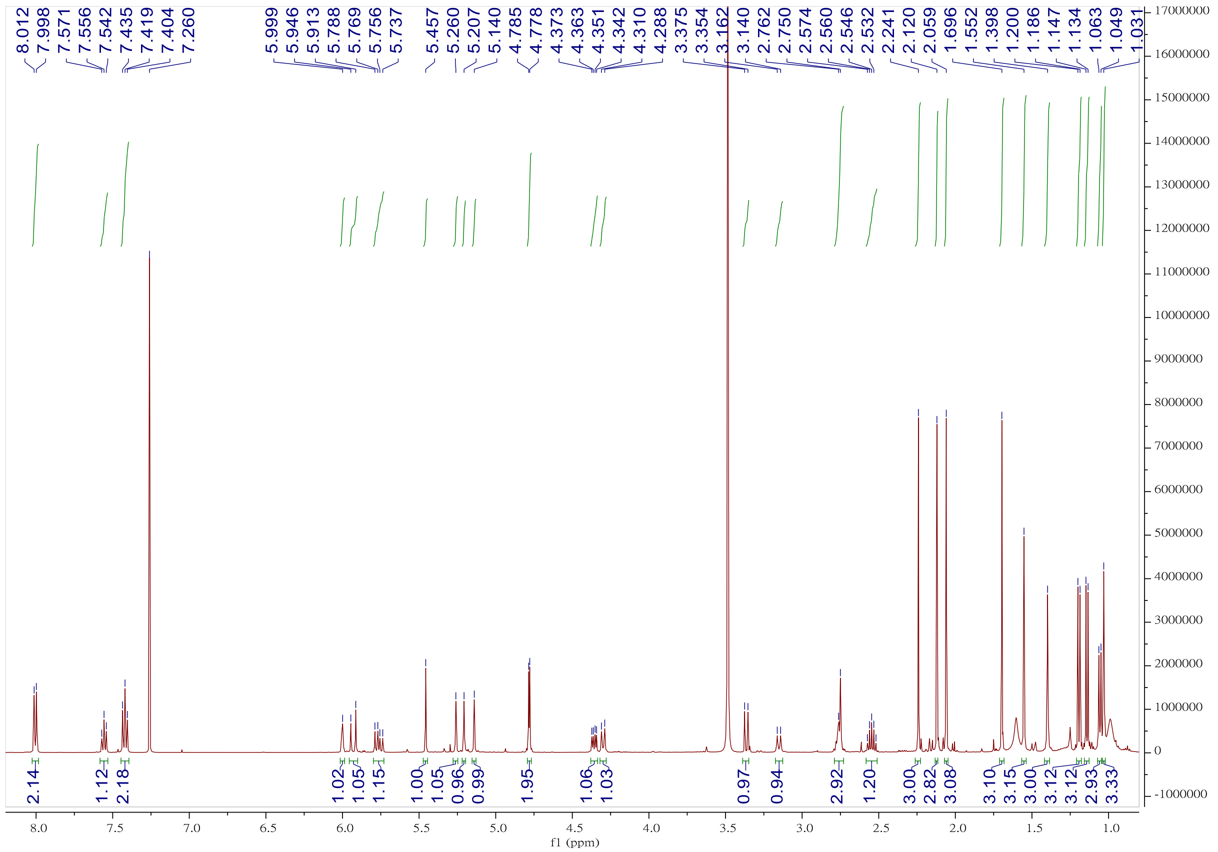

Figure S43. The ^{1}H-NMR spectrum of usambariphane F (**7**) (500 MHz, $CDCl_3$).

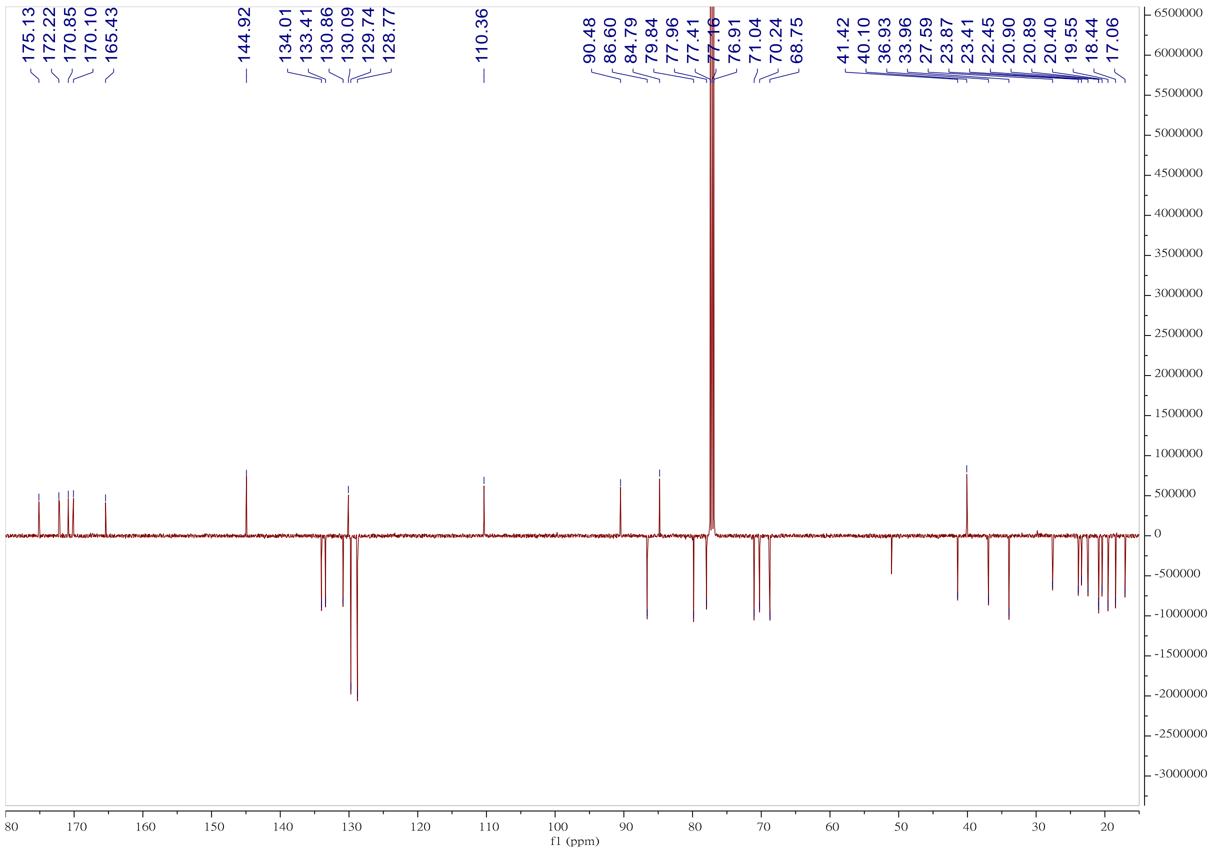

Figure S44. The ^{13}C-JMOD spectrum of usambariphane F (**7**) (125 MHz, $CDCl_3$).

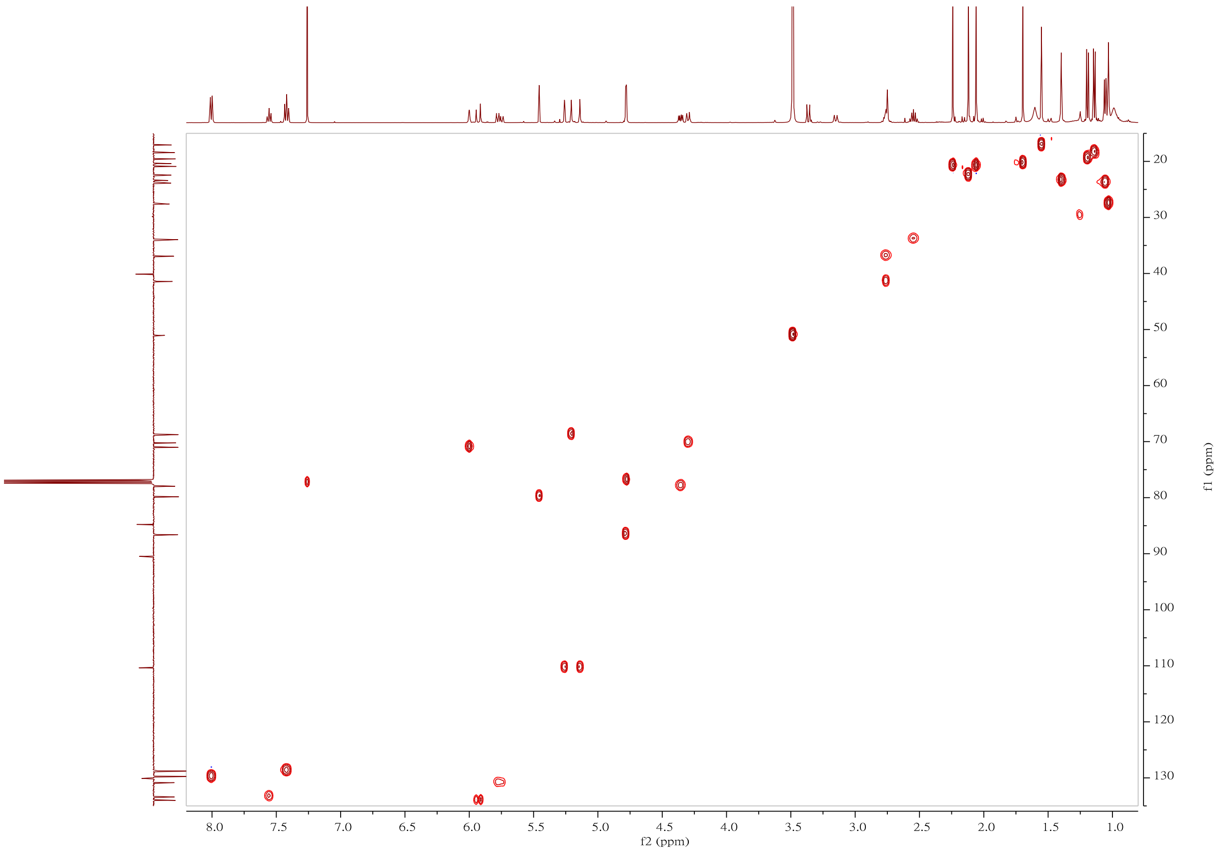

Figure S45. The HSQC spectrum of usambariphane F (**7**).

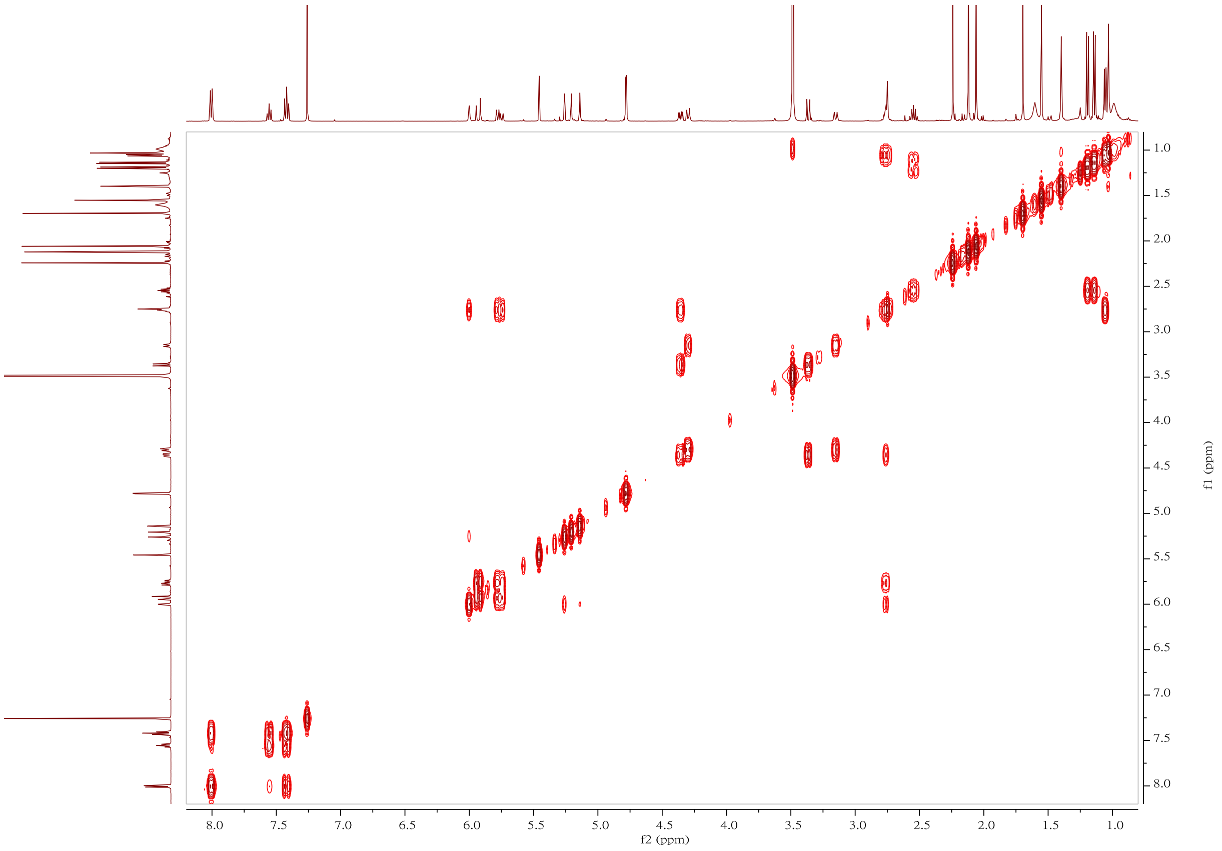

Figure S46. The ^{1}H-^{1}H COSY spectrum of usambariphane F (**7**).

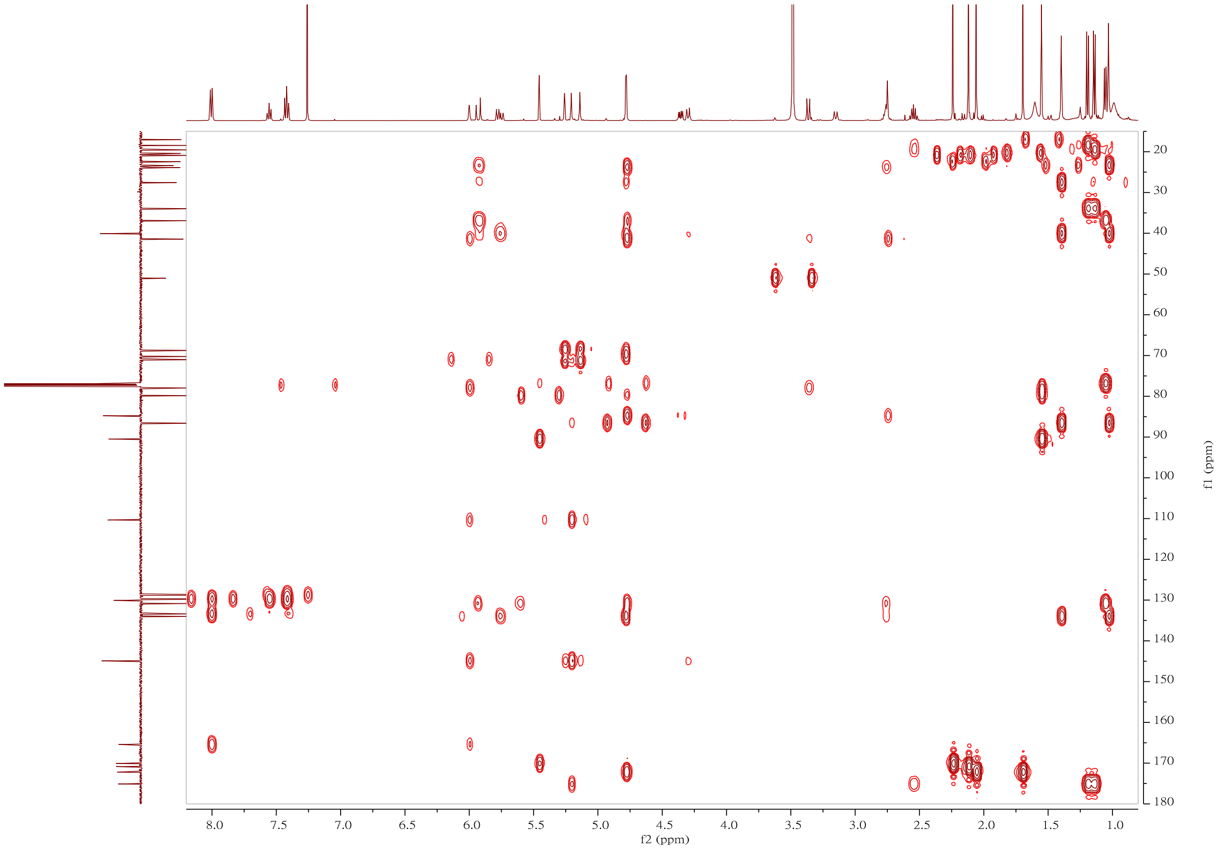

Figure S47. The HMBC spectrum of usambariphane F (**7**).

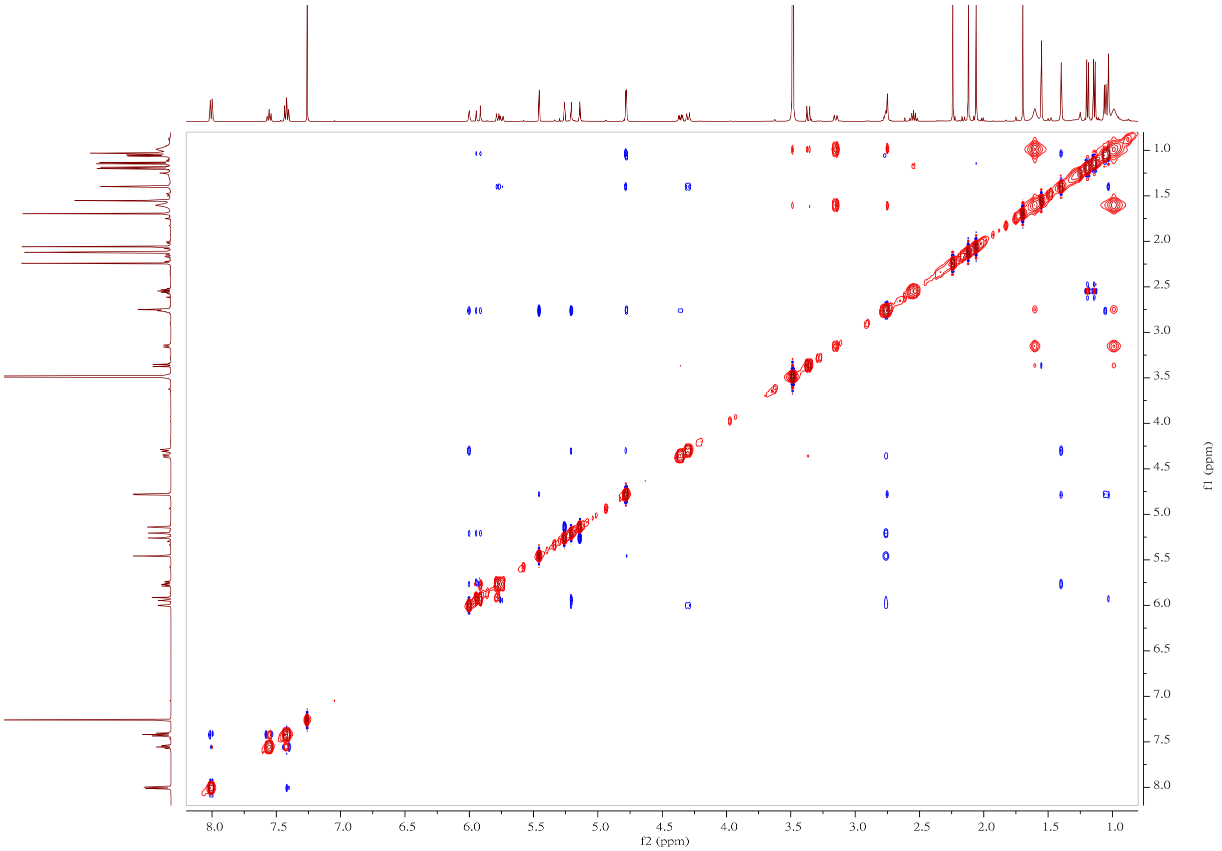

Figure S48. The NOESY spectrum of usambariphane F (**7**).

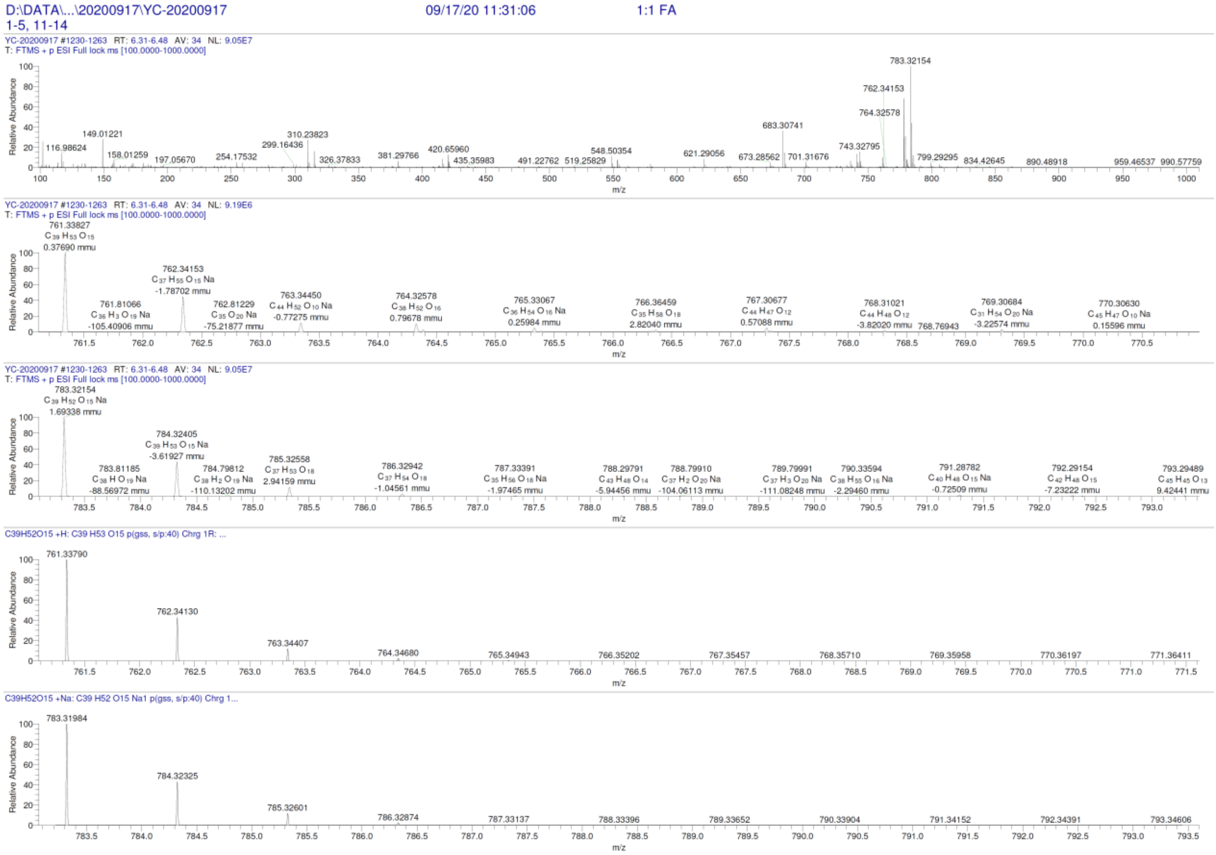

Figure S49. The HR-ESI-MS spectra of usambariphane F (**7**).

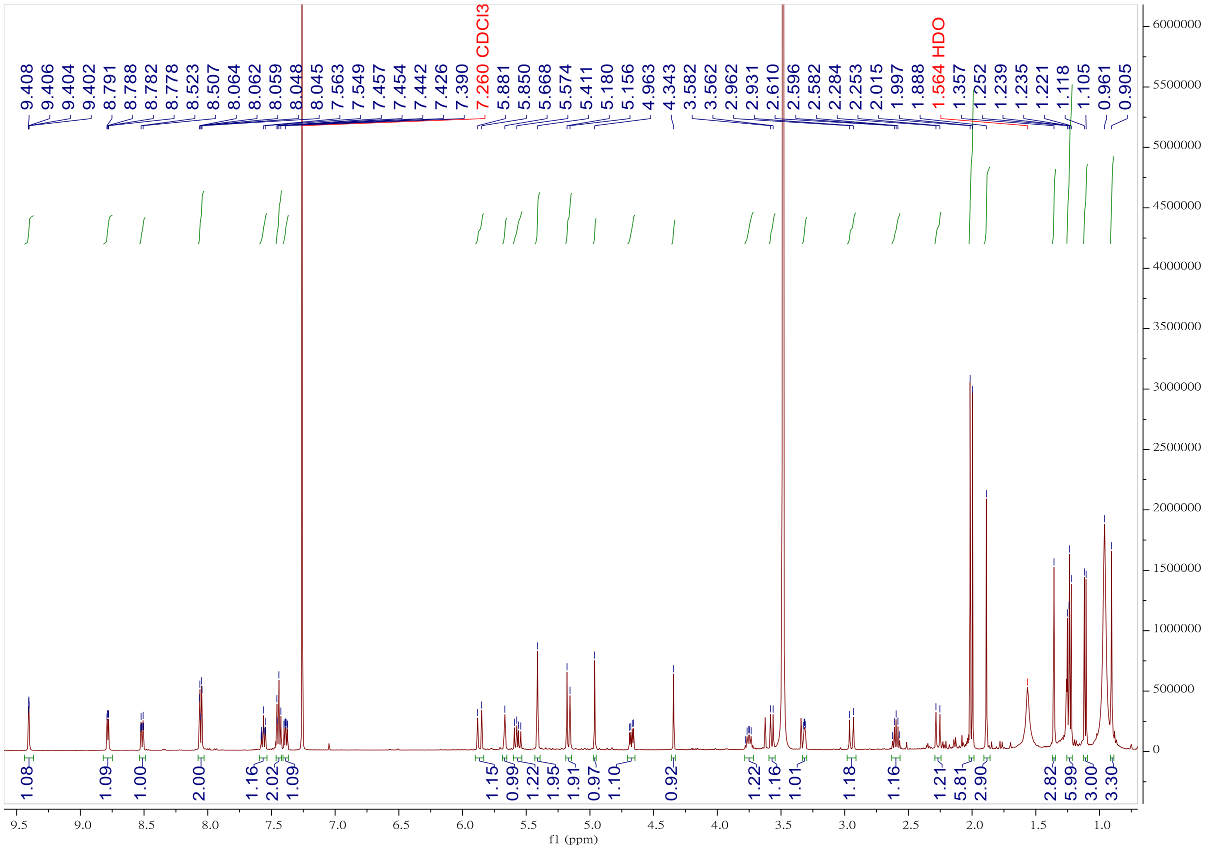

Figure S50. The ^{1}H-NMR spectrum of usambariphane G (**8**) (500 MHz, $CDCl_3$).

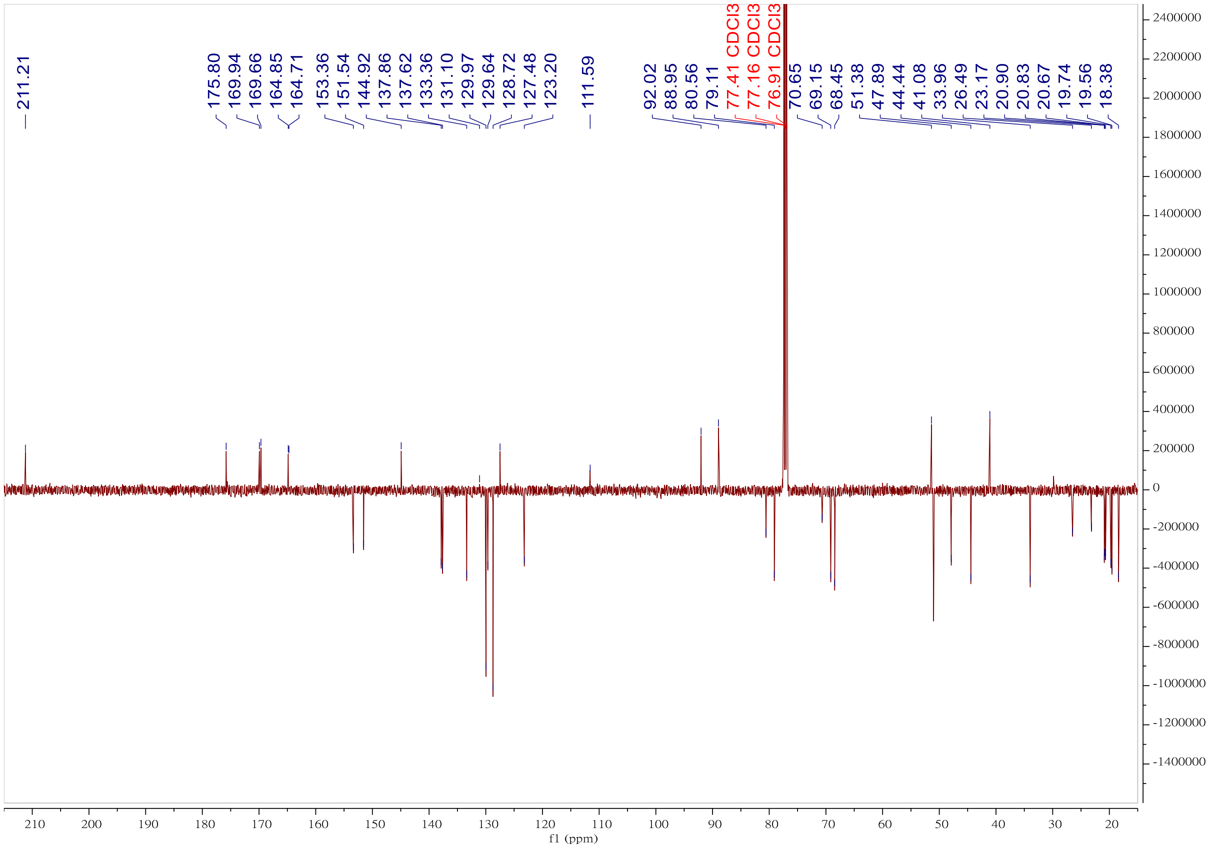

Figure S51. The ^{13}C-JMOD spectrum of usambariphane G (**8**) (125 MHz, $CDCl_3$).

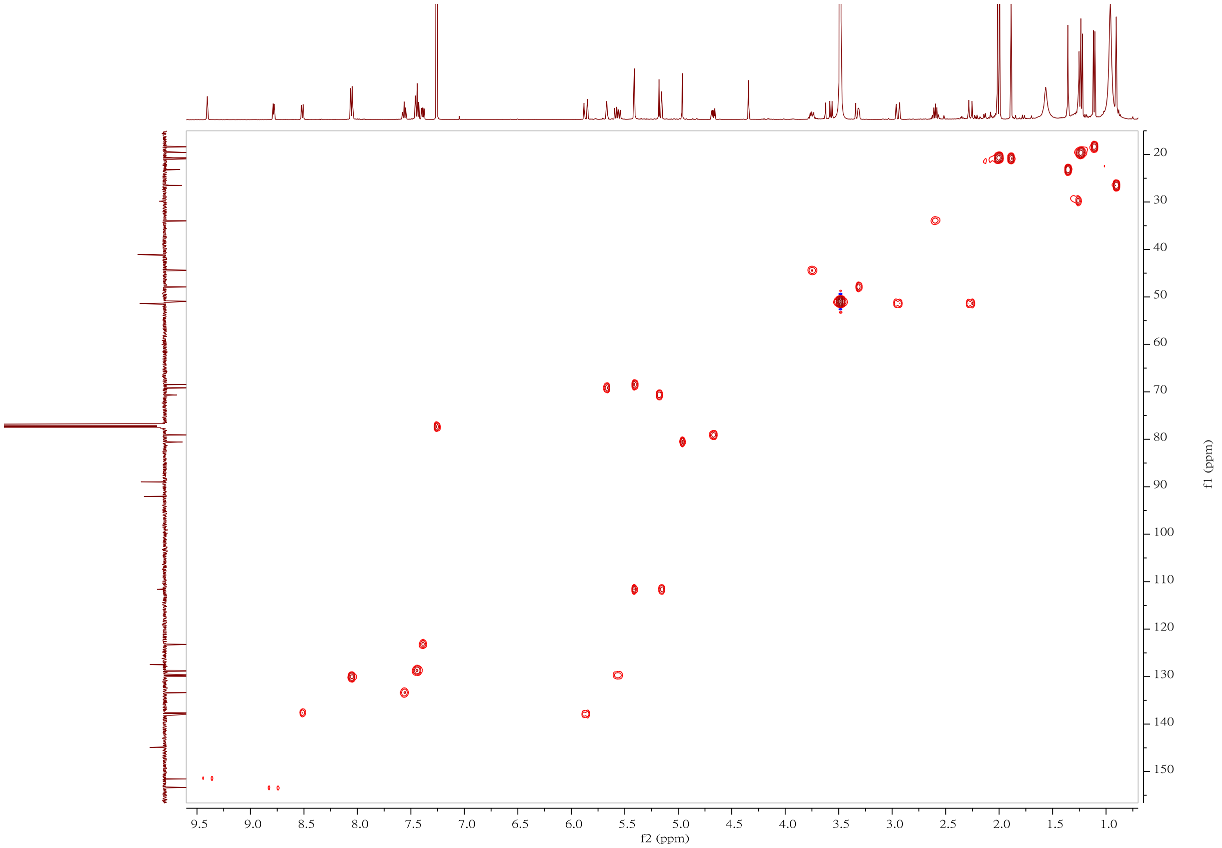

Figure S52. The HSQC spectrum of usambariphane G (**8**).

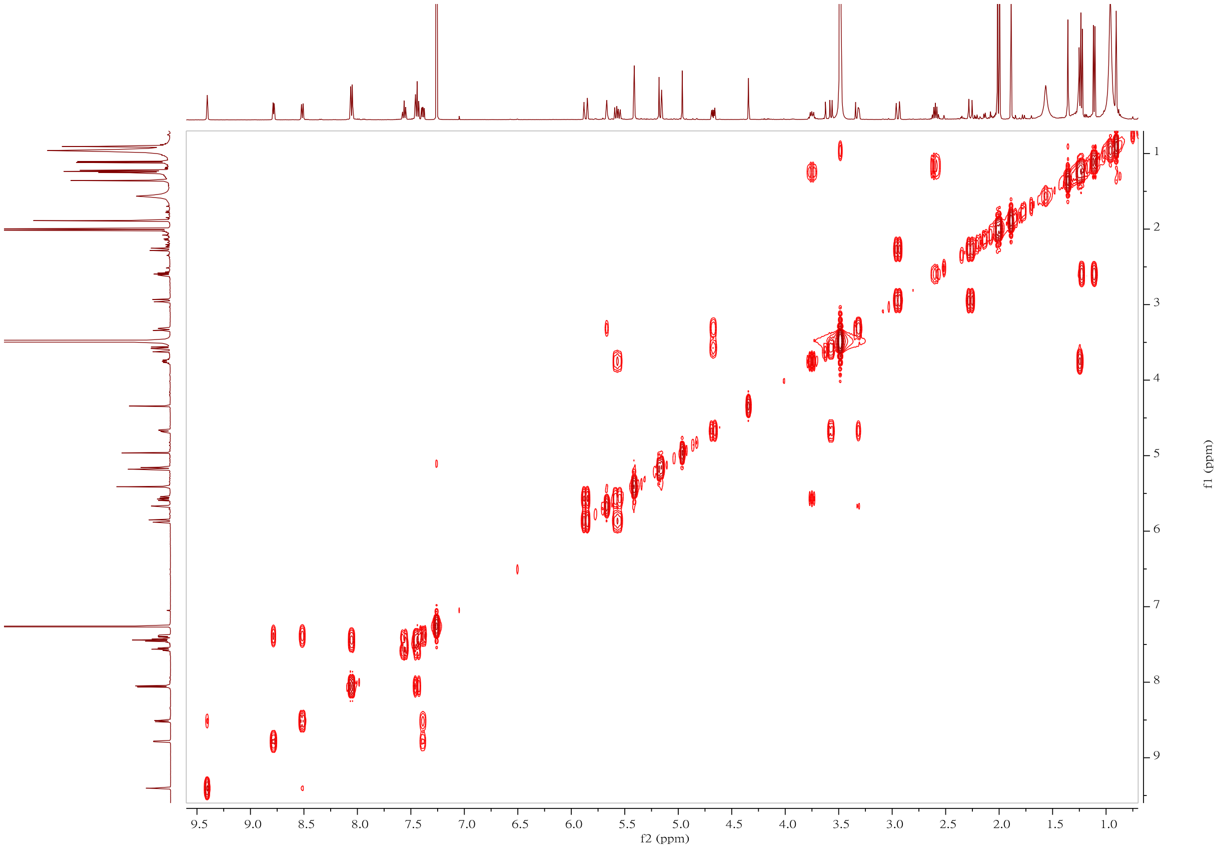

Figure S53. The ^{1}H-^{1}H COSY spectrum of usambariphane G (**8**).

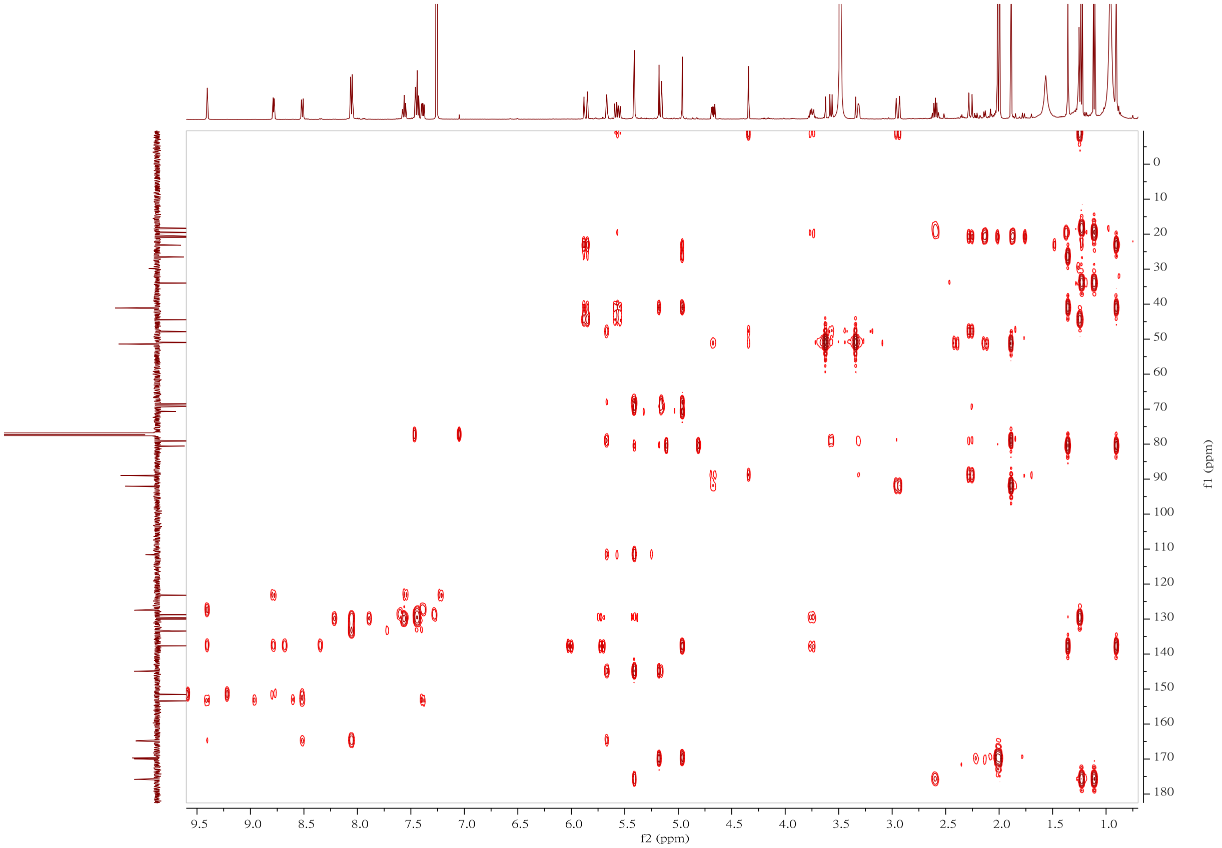

Figure S54. The HMBC spectrum of usambariphane G (**8**).

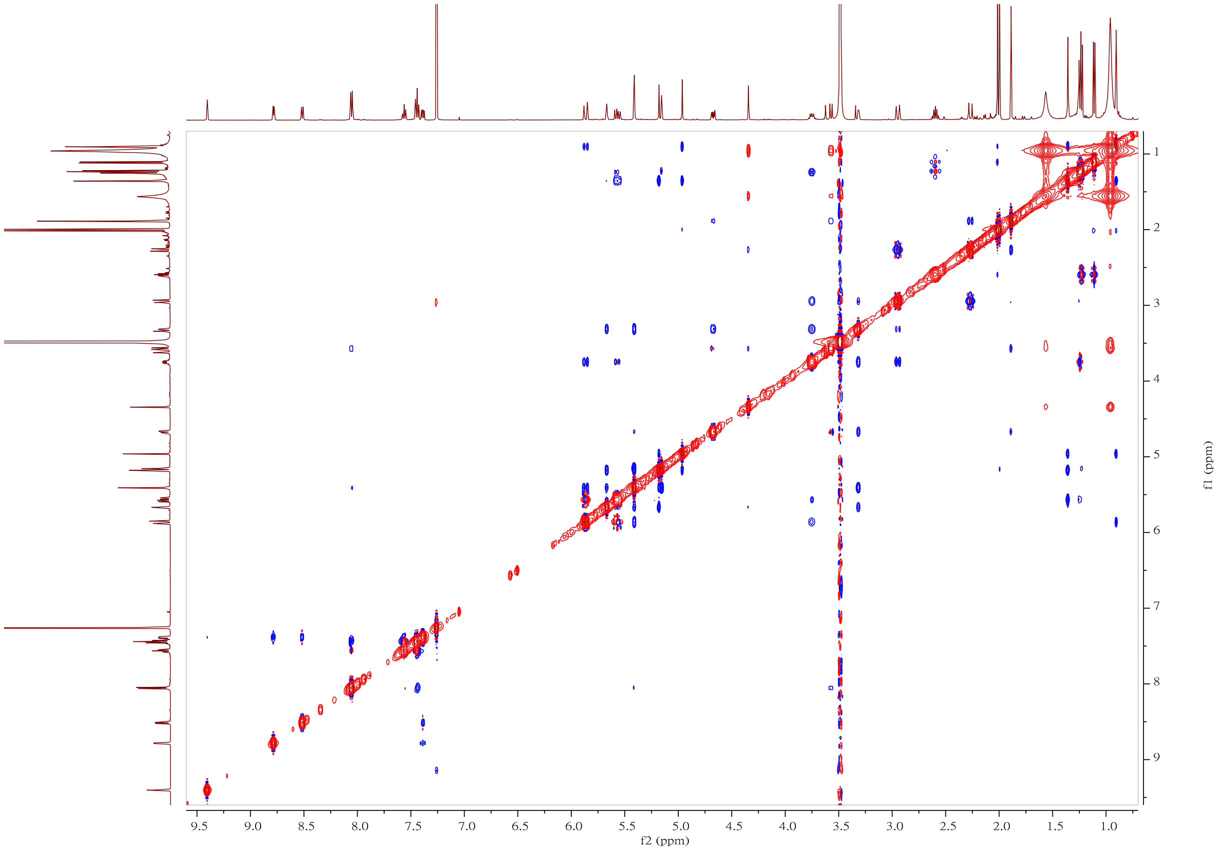

Figure S55. The NOESY spectrum of usambariphane G (**8**).

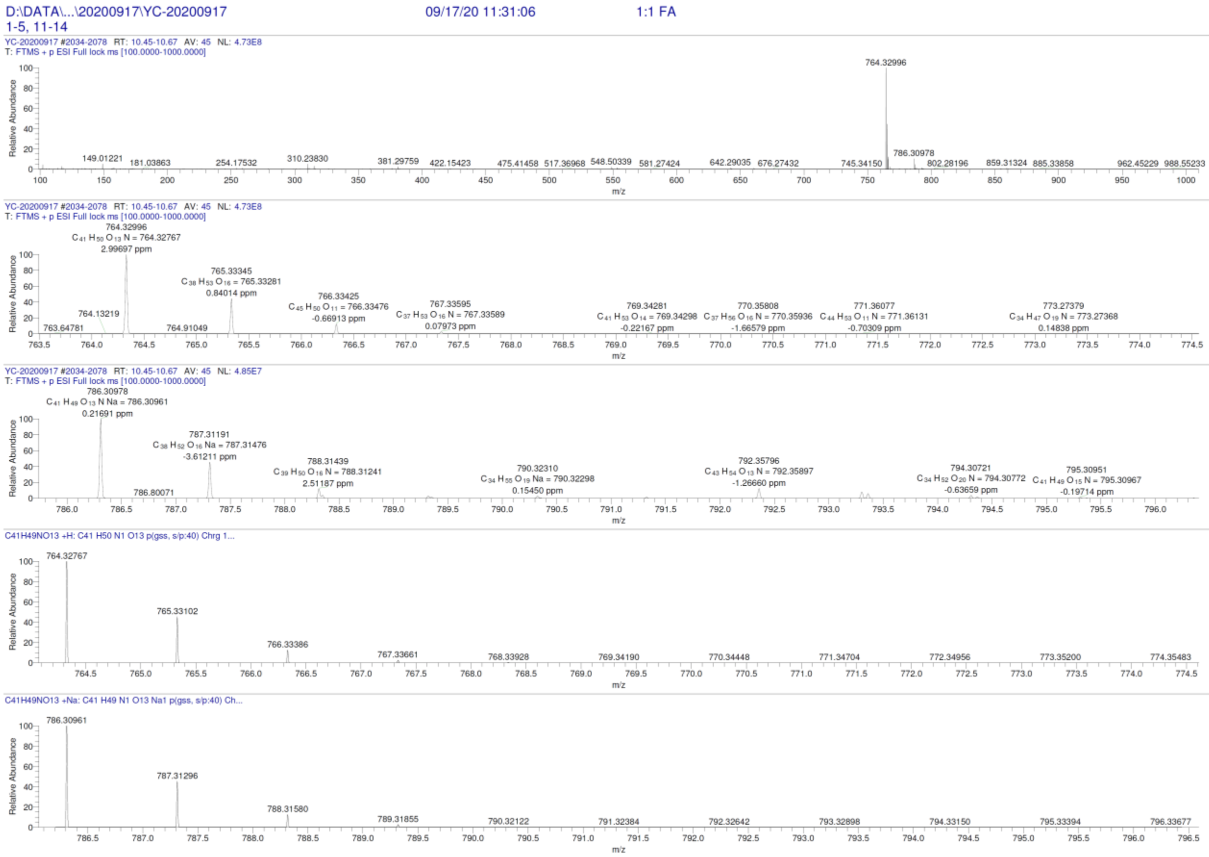

Figure S56. The HR-ESI-MS spectra of usambariphane G (**8**).

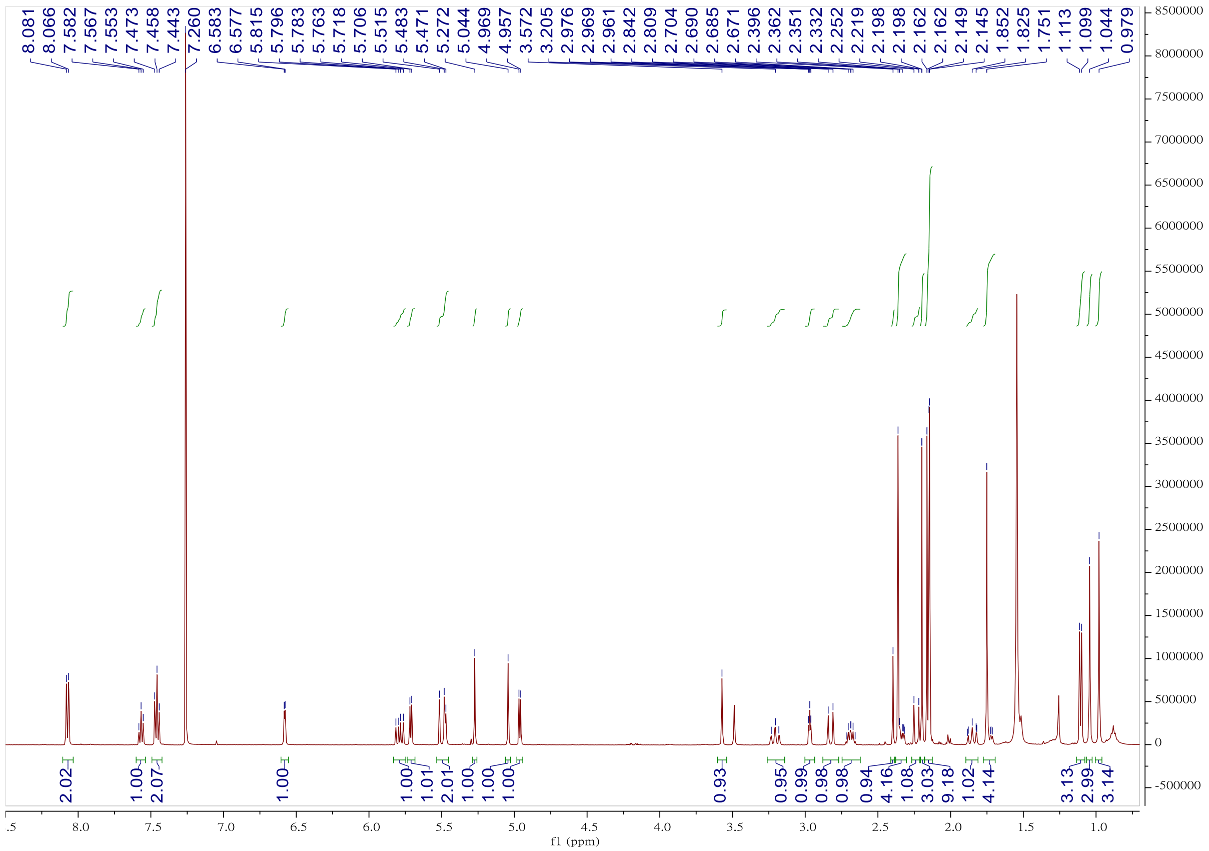

Figure S57. The ^{1}H-NMR spectrum of isoterracinolide C (**9**) (500 MHz, $CDCl_3$).

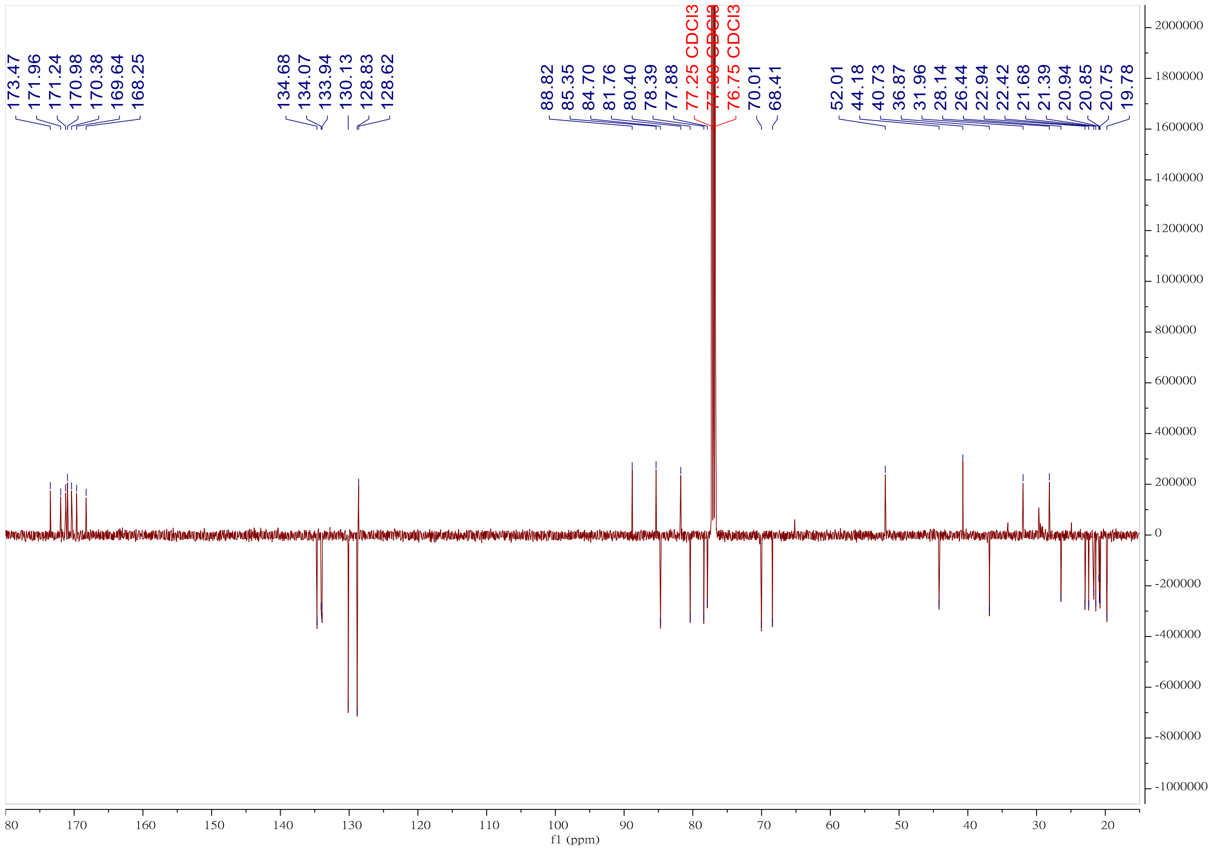

Figure S58. The ^{13}C-JMOD spectrum of isoterracinolide C (**9**) (500 MHz, $CDCl_3$).

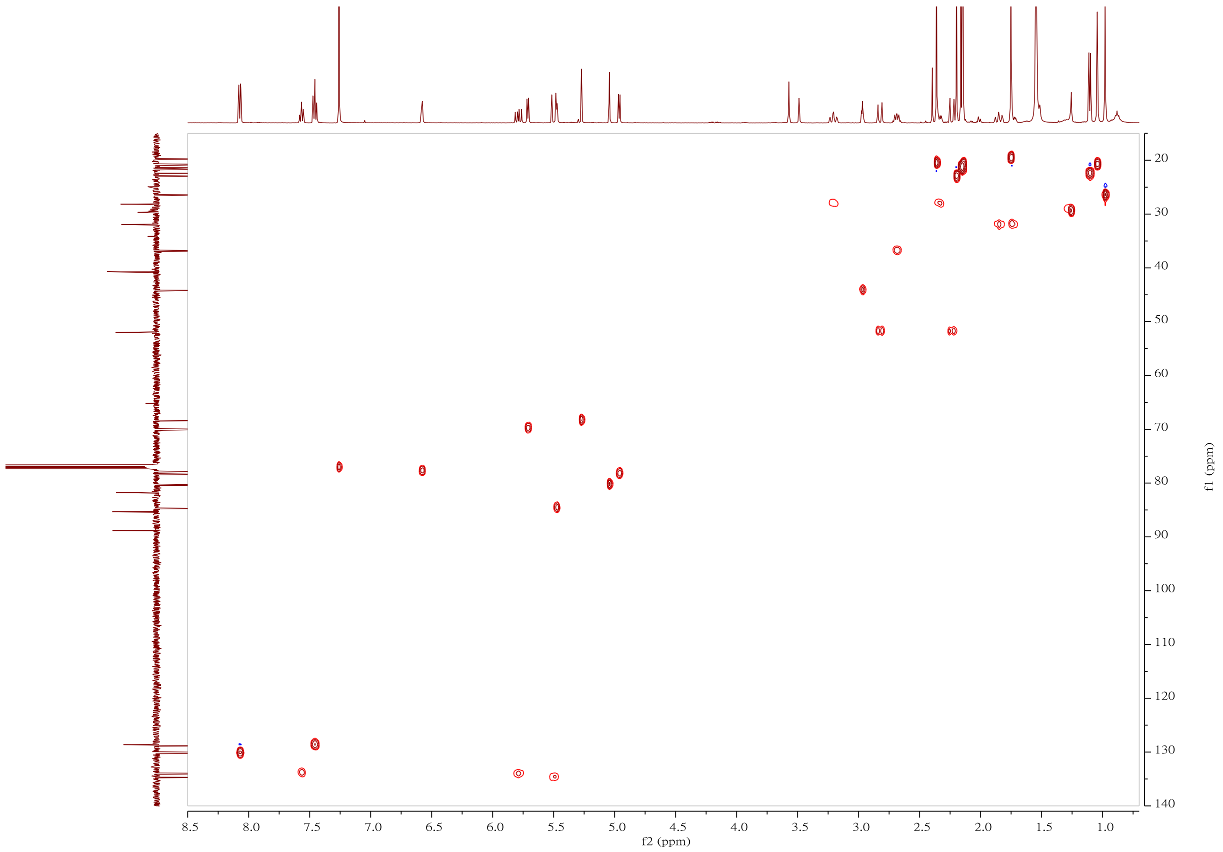

Figure S59. The HSQC spectrum of isoterracinolide C (**9**).

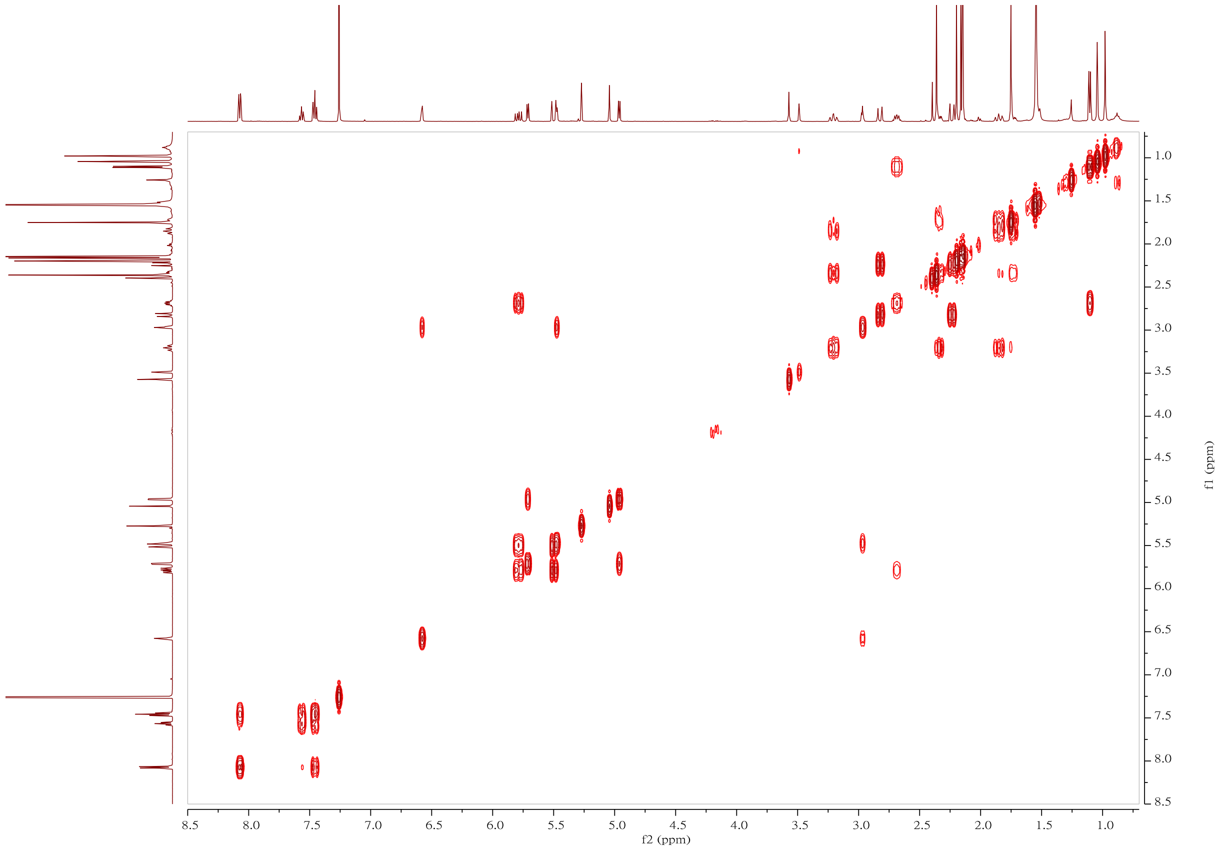

Figure S60. The ^{1}H-^{1}H COSY spectrum of isoterracinolide C (**9**).

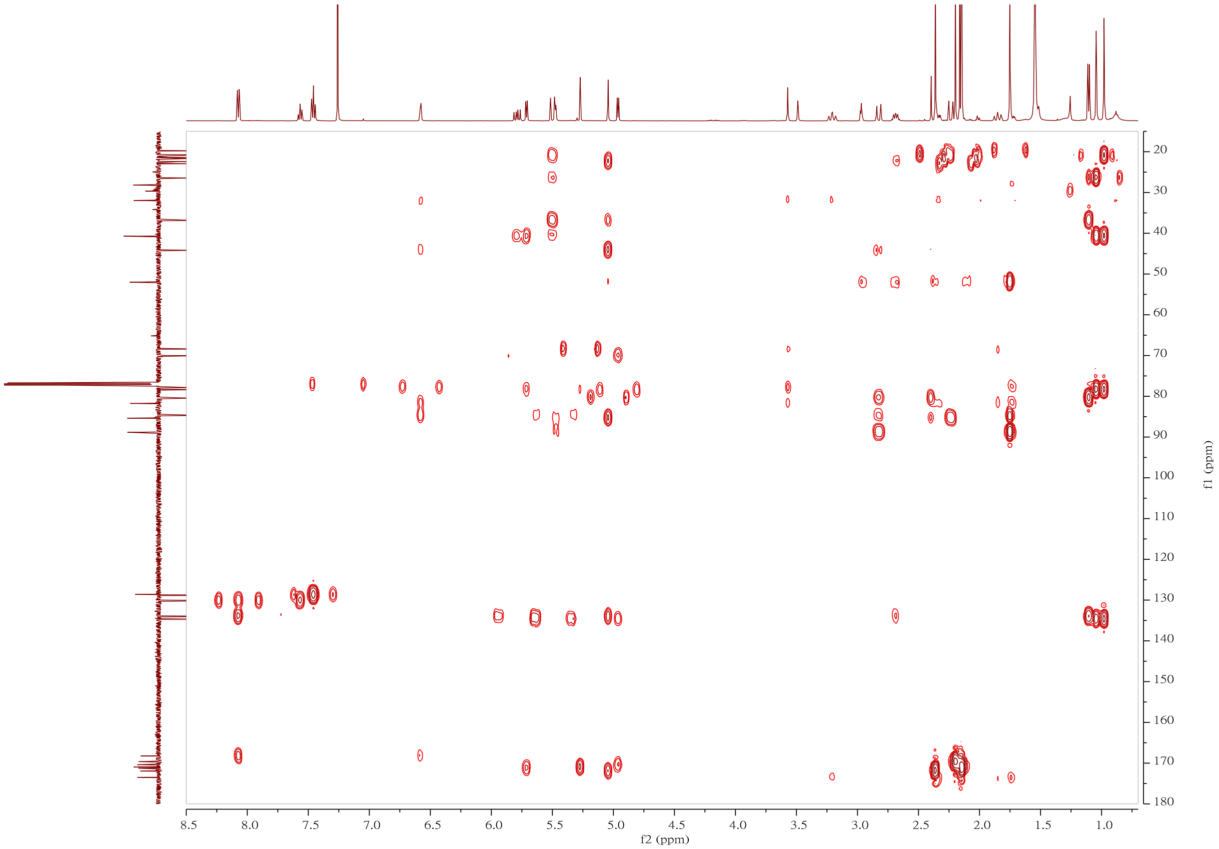

Figure S61. The HMBC spectrum of isoterracinolide C (**9**).

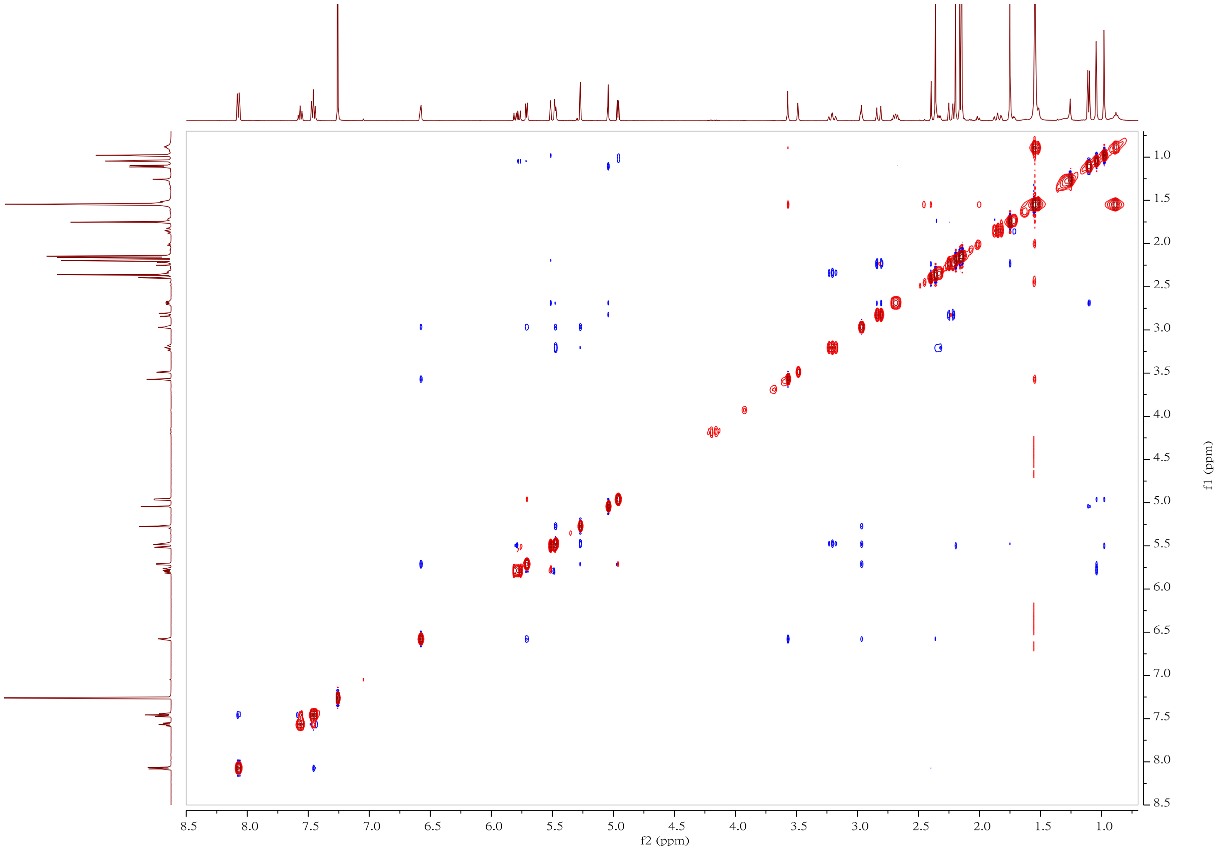

Figure S62. The NOESY spectrum of isoterracinolide C (**9**).

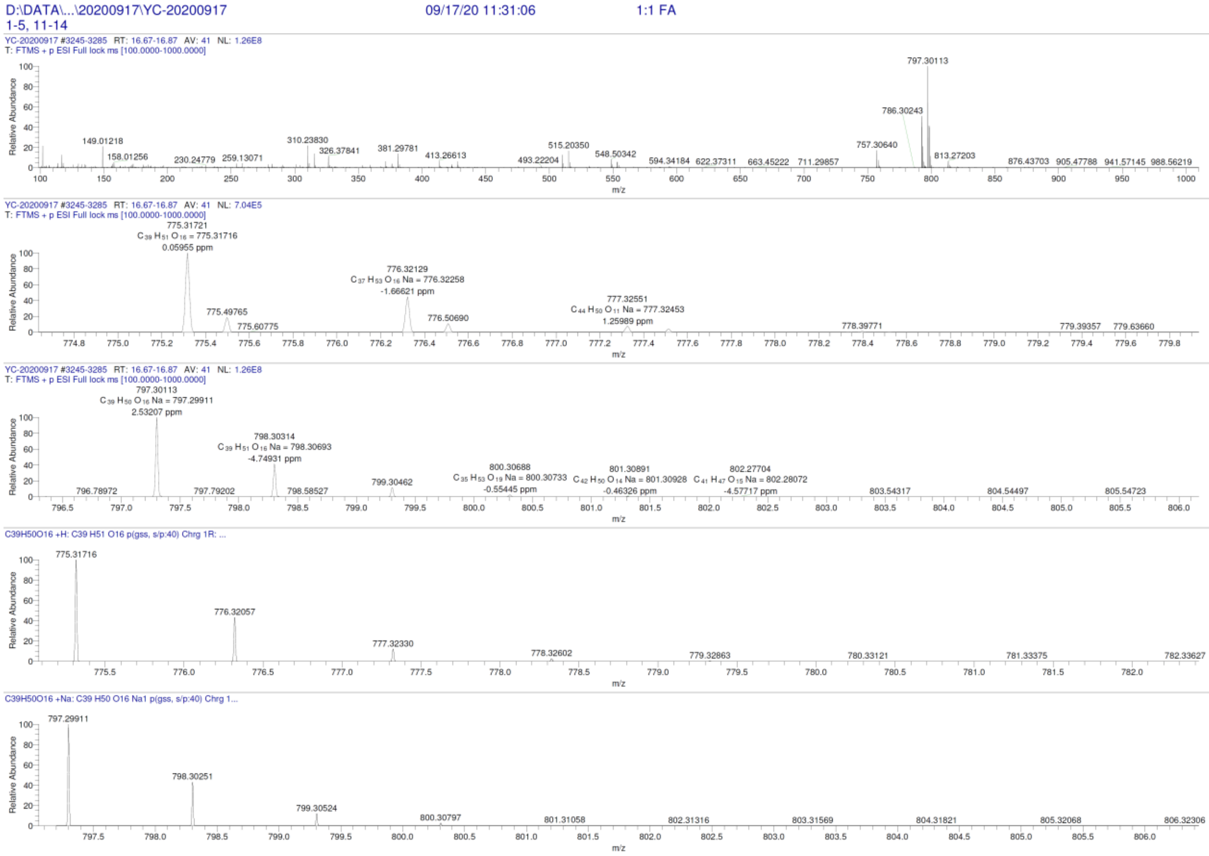

Figure S63. The HR-ESI-MS spectra of isoterracinolide C (**9**).

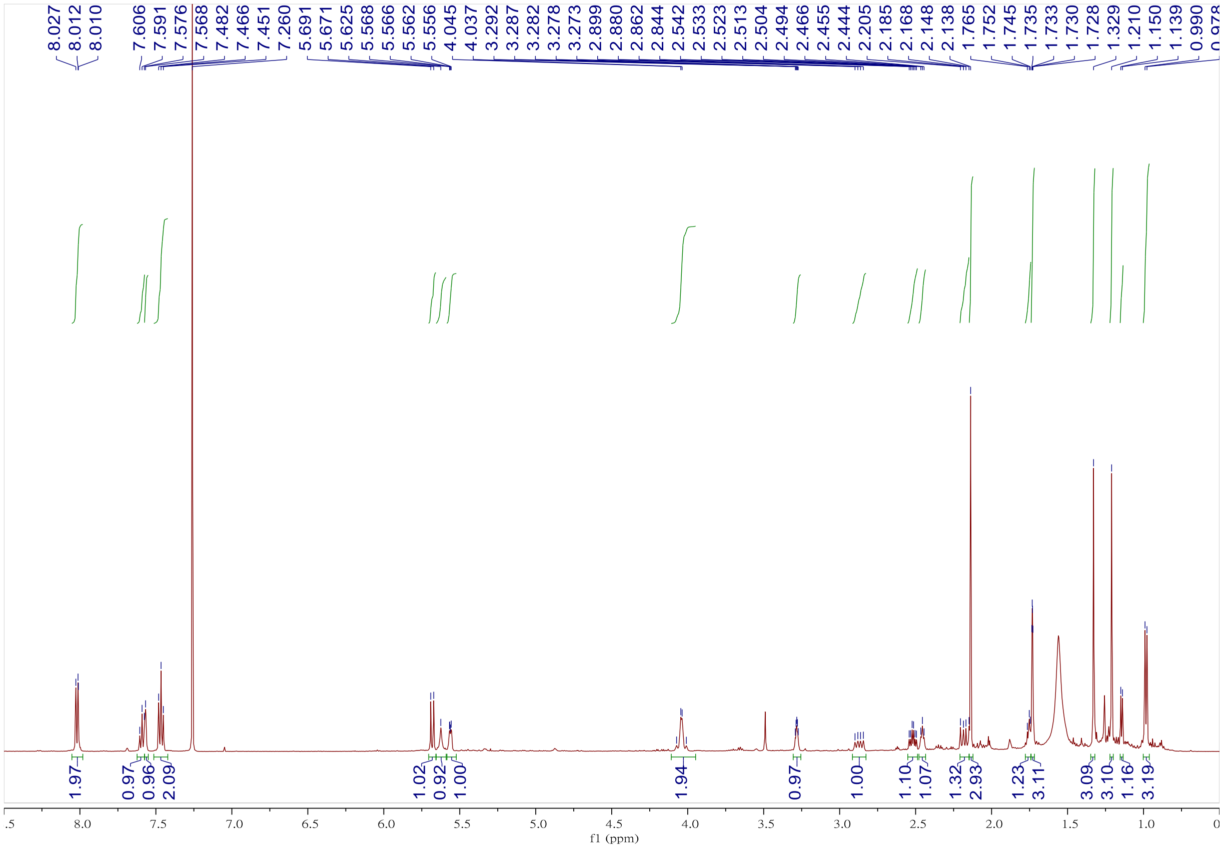

Figure S64. The ^{1}H-NMR spectrum of 4β-crotignoid K (**14**) (500 MHz, $CDCl_3$).

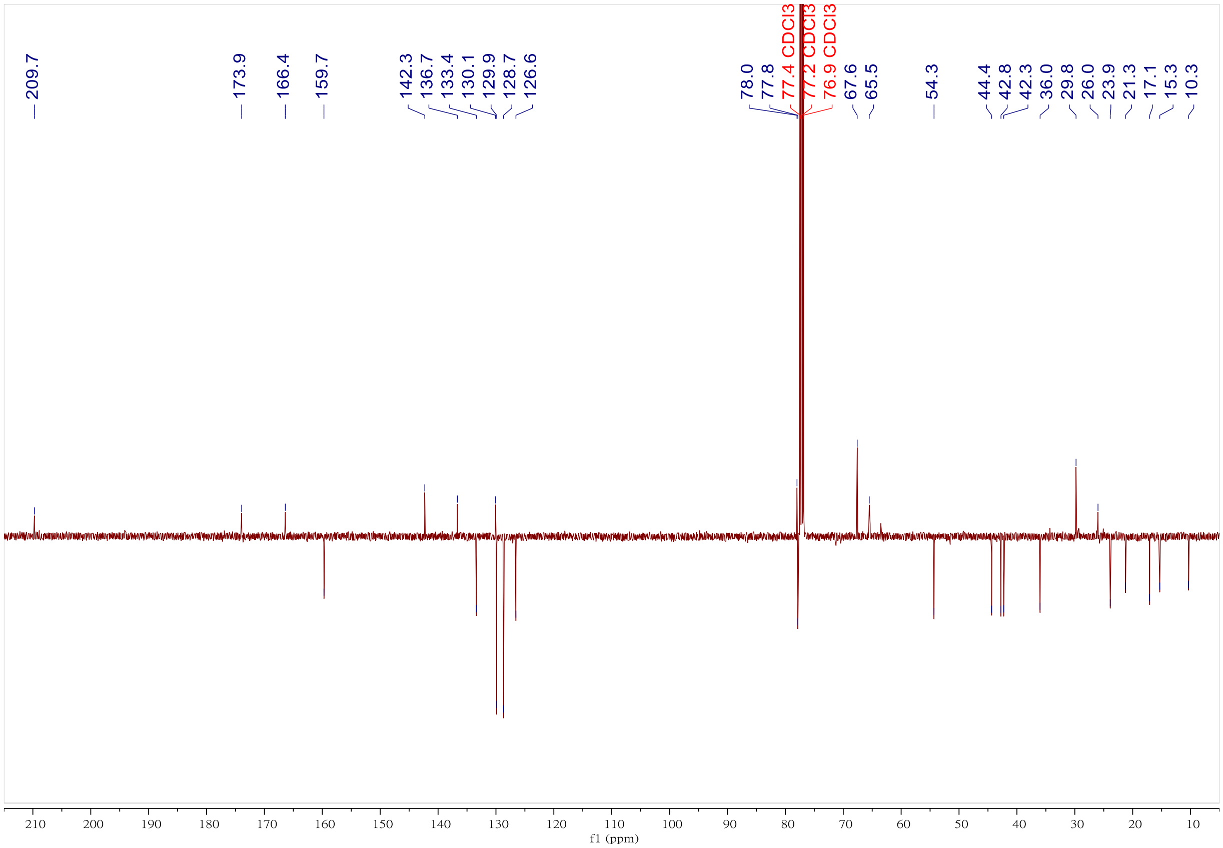

Figure S65. The ^{13}C-JMOD spectrum of 4β-crotignoid K (**14**) (125 MHz, $CDCl_3$).

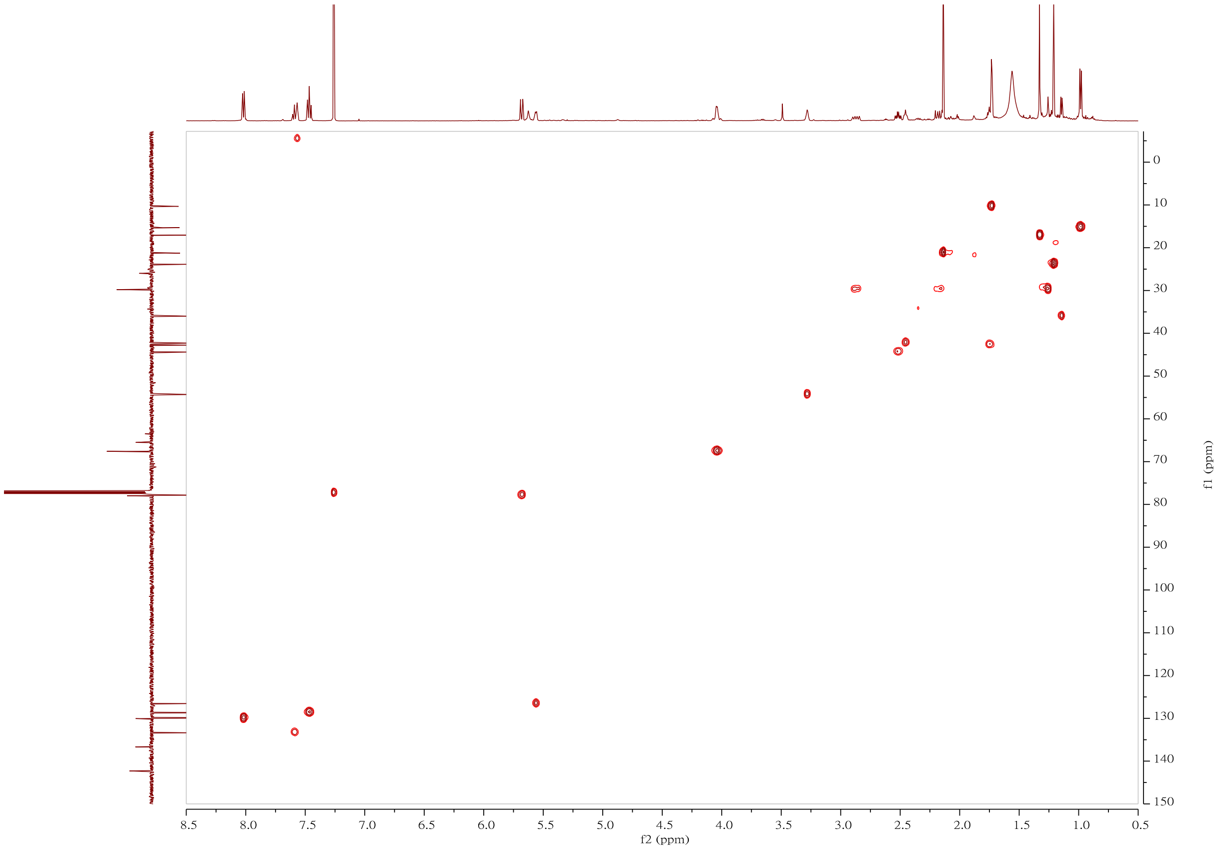

Figure S66. The HSQC spectrum of 4β-crotignoid K (**14**).

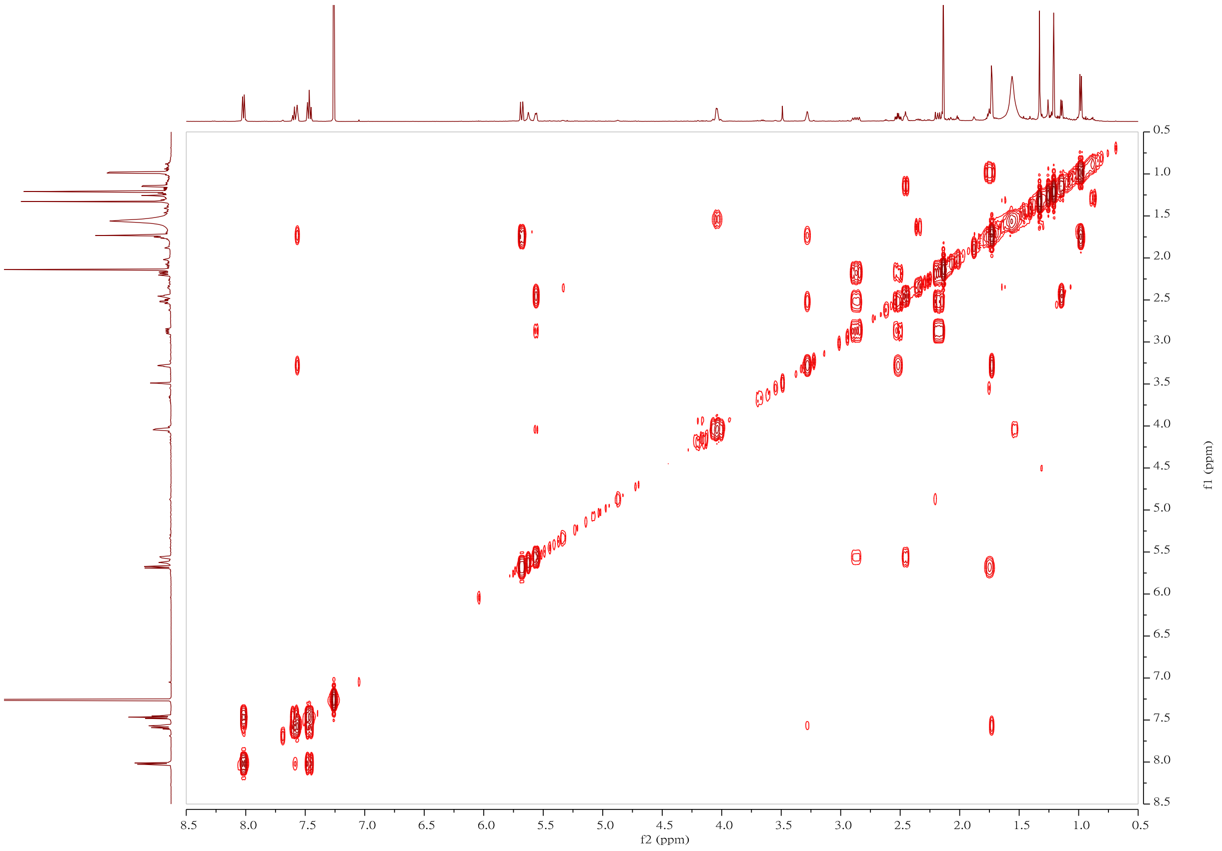

Figure S67. The ^{1}H-^{1}H COSY spectrum of 4β-crotignoid K (**14**).

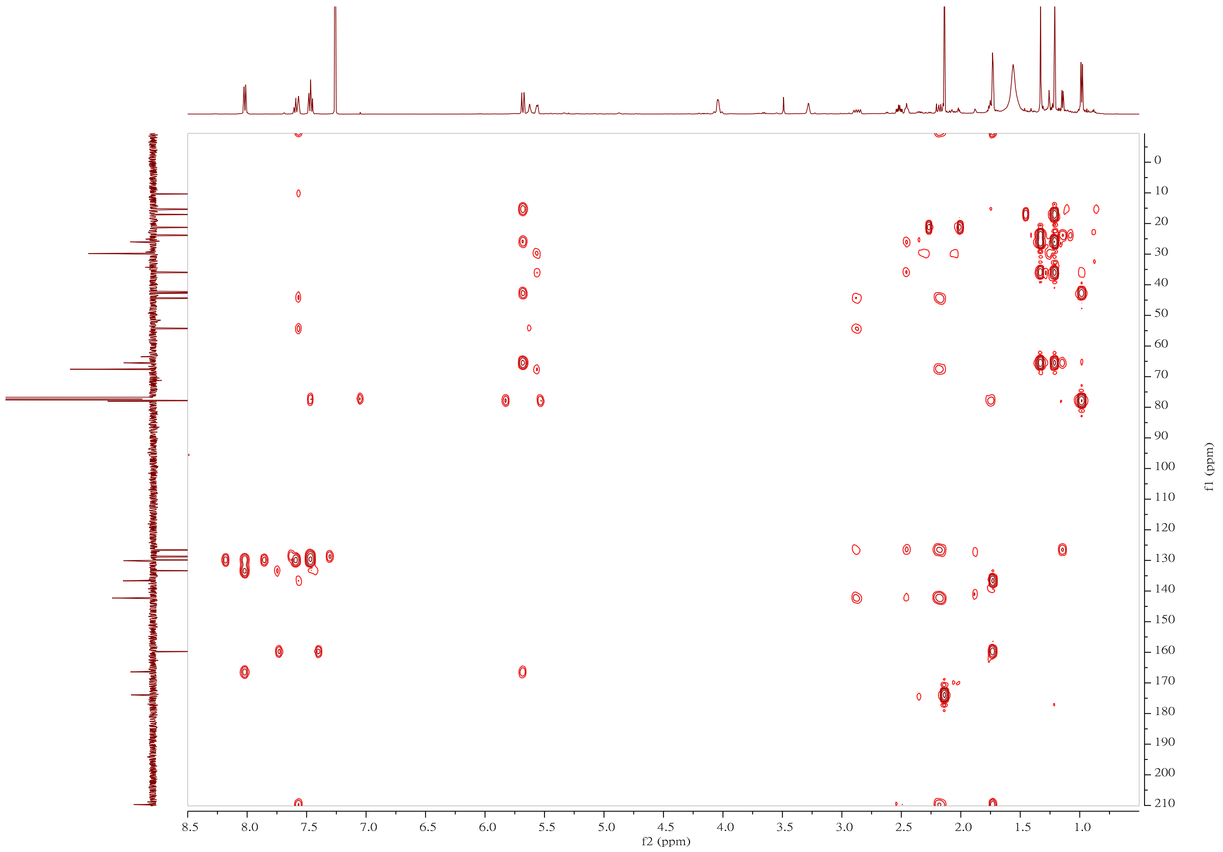

Figure S68. The HMBC spectrum of 4*β*-crotignoid K (**14**).

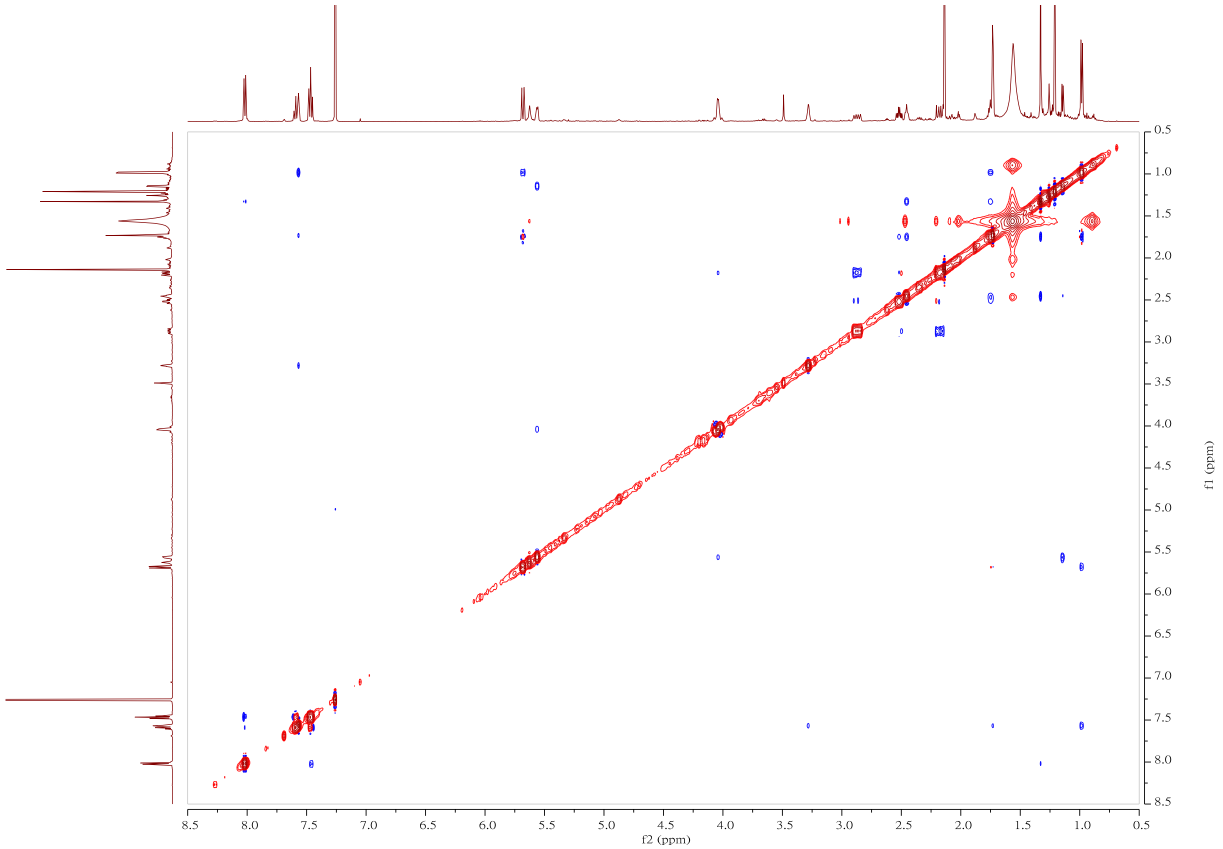

Figure S69. The NOESY spectrum of 4*β*-crotignoid K (**14**).

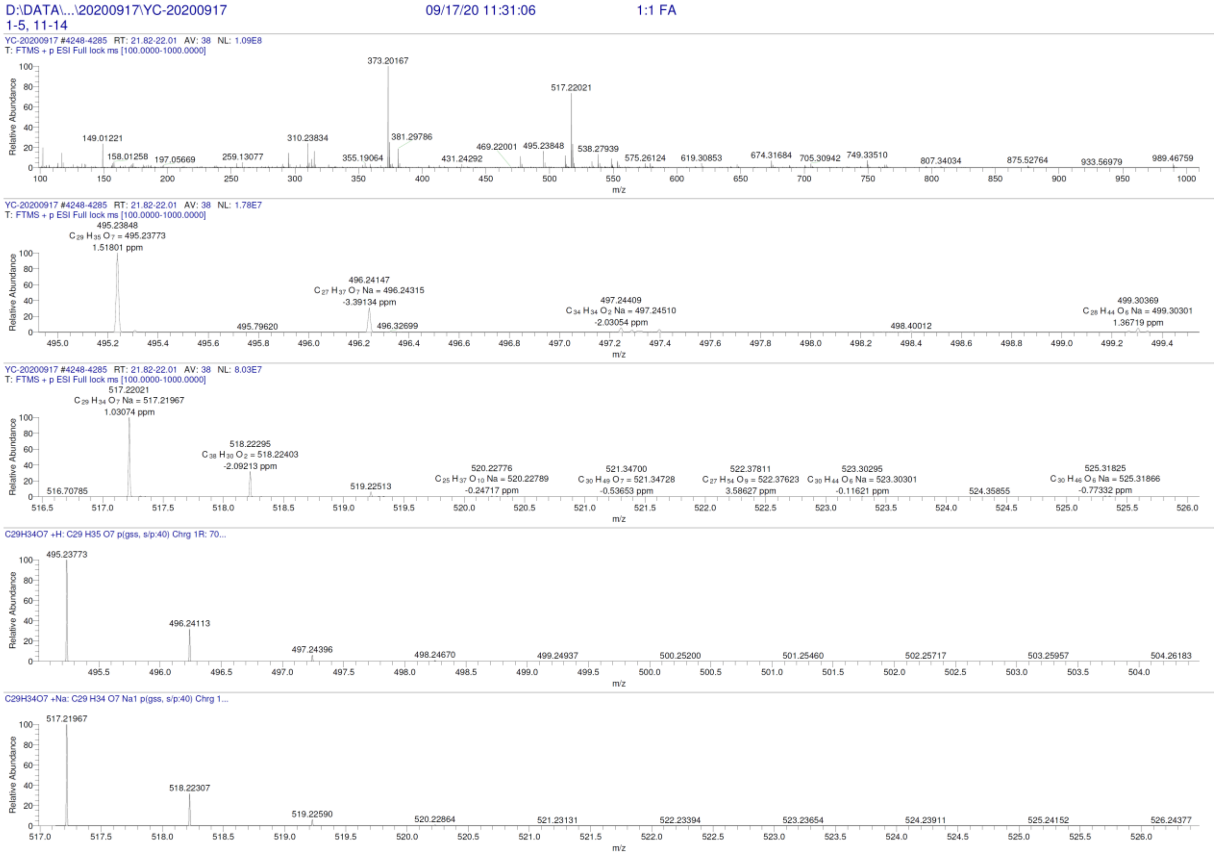

Figure S70. The HR-ESI-MS spectra of 4*β*-crotignoid K (**14**).

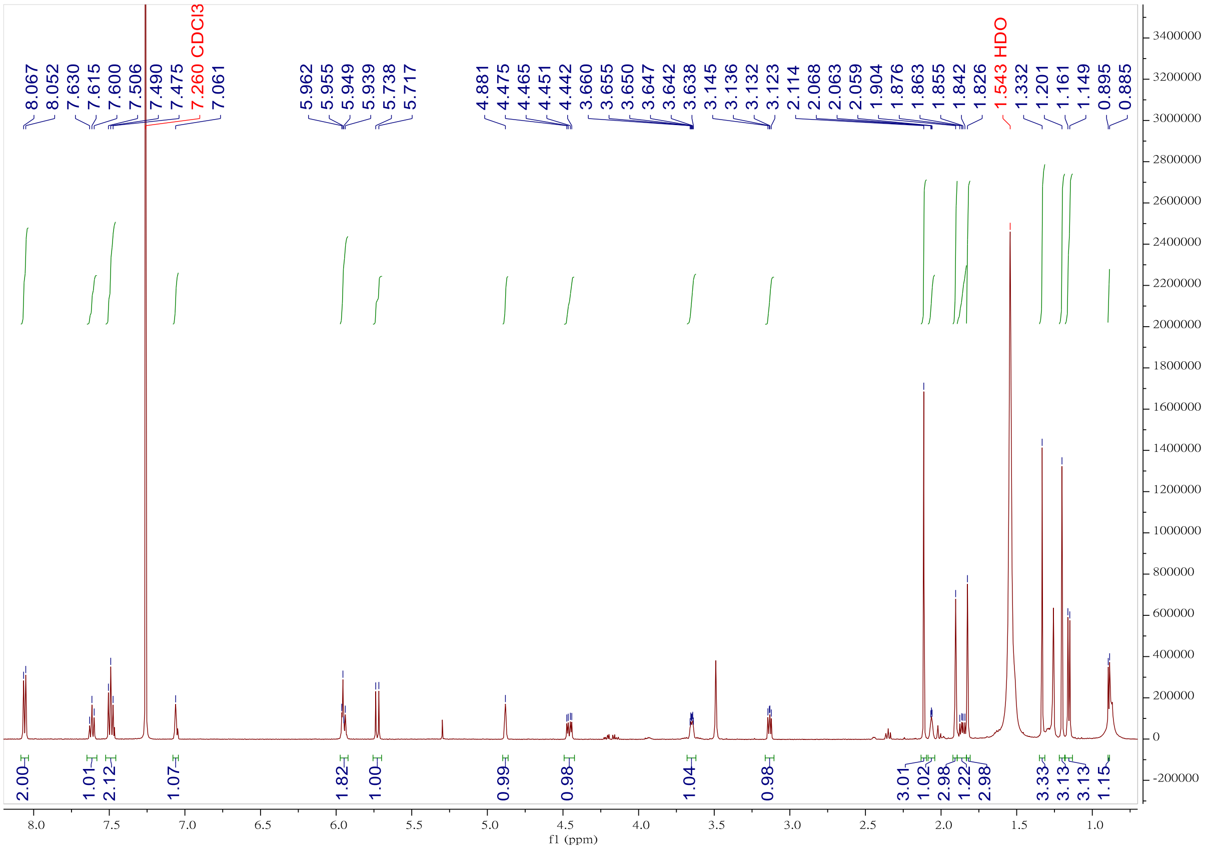

Figure S71. The ^{1}H-NMR spectrum of euphodendriane B (**15**) (500 MHz, $CDCl_3$).

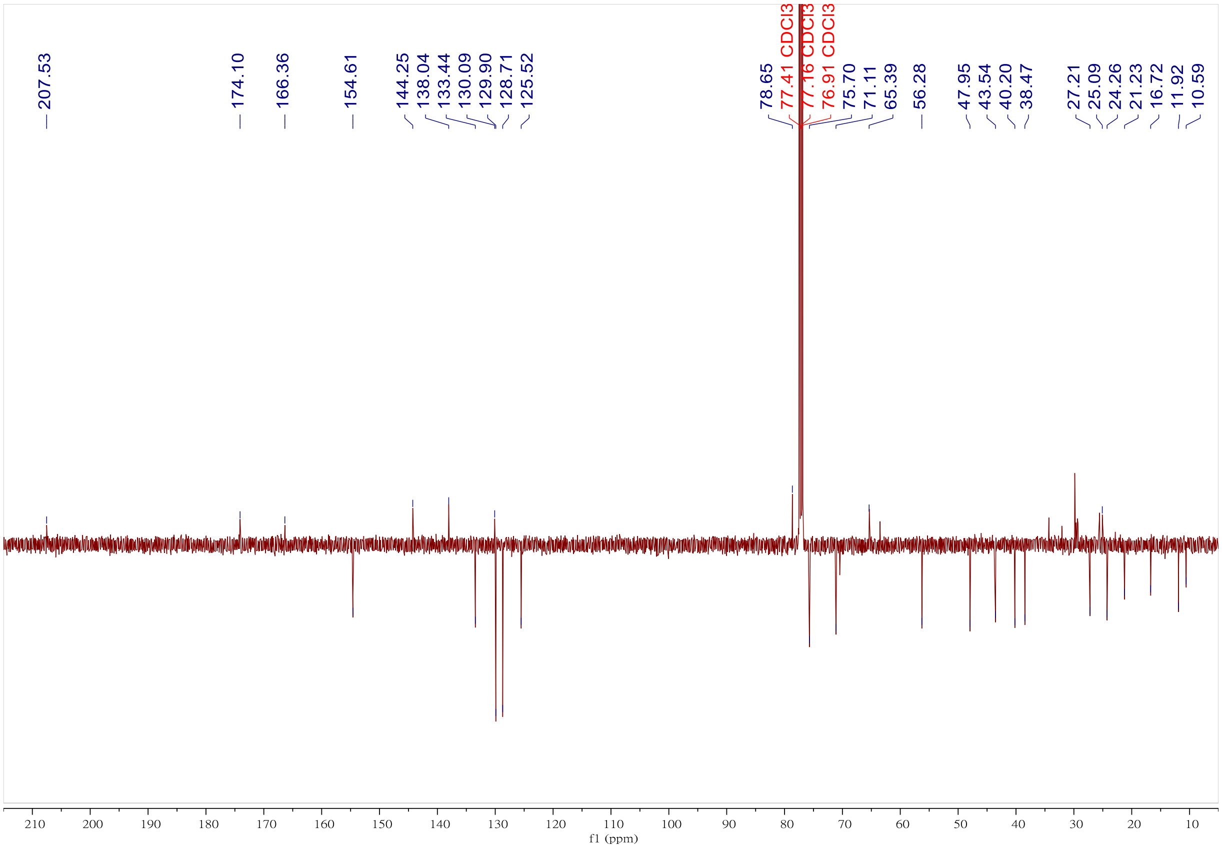

Figure S72. The ^{13}C-JMOD spectrum of euphodendriane B (**15**) (125 MHz, $CDCl_3$).

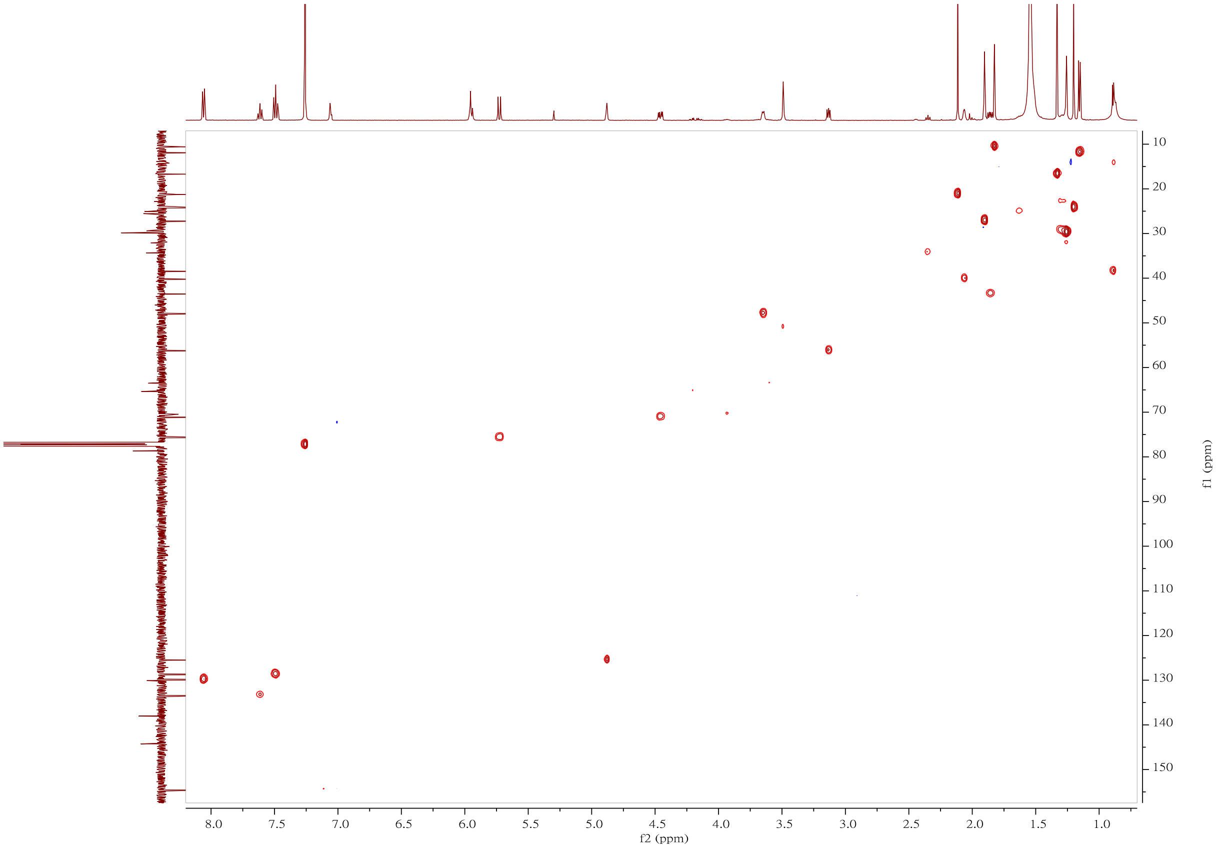

Figure S73. The HSQC spectrum of euphodendriane B (**15**).

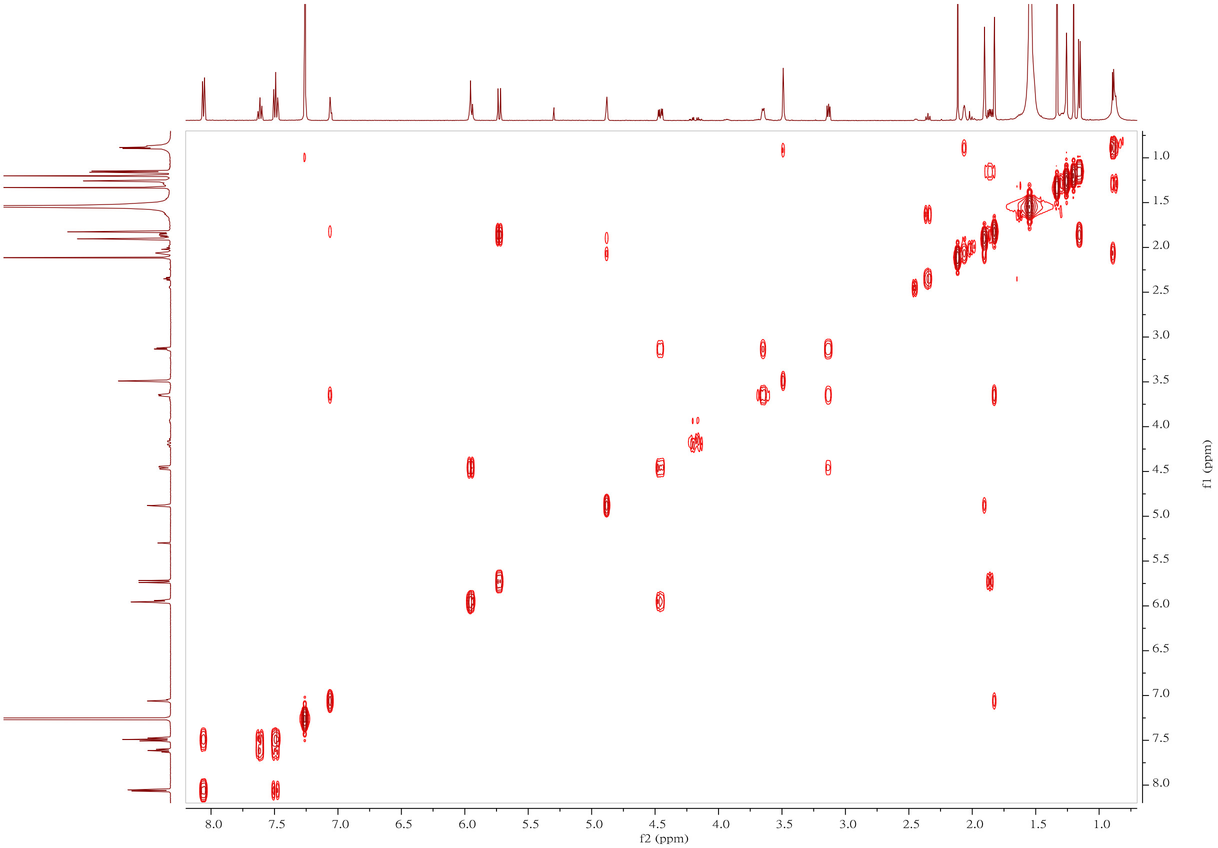

Figure S74. The ^{1}H-^{1}H COSY spectrum of euphodendriane B (**15**).

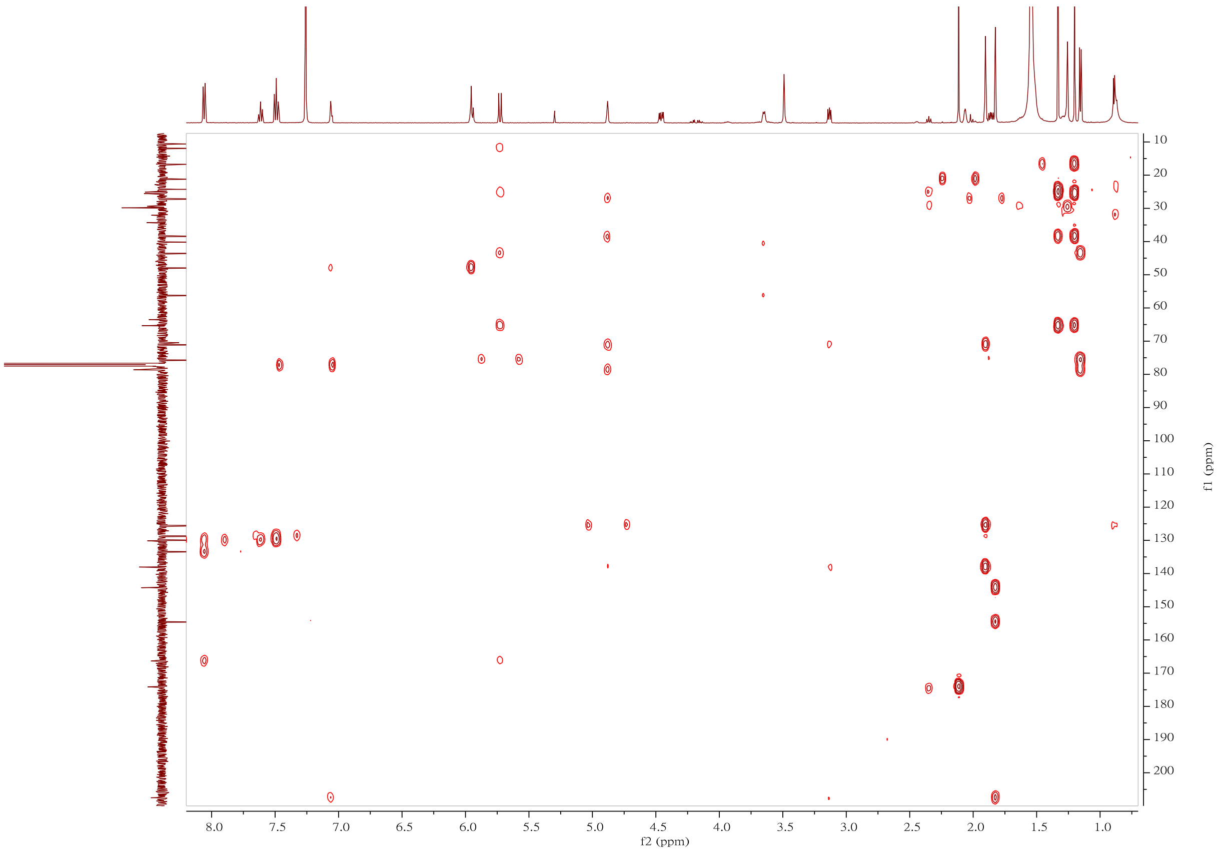

Figure S75. The HMBC spectrum of euphodendriane B (**15**).

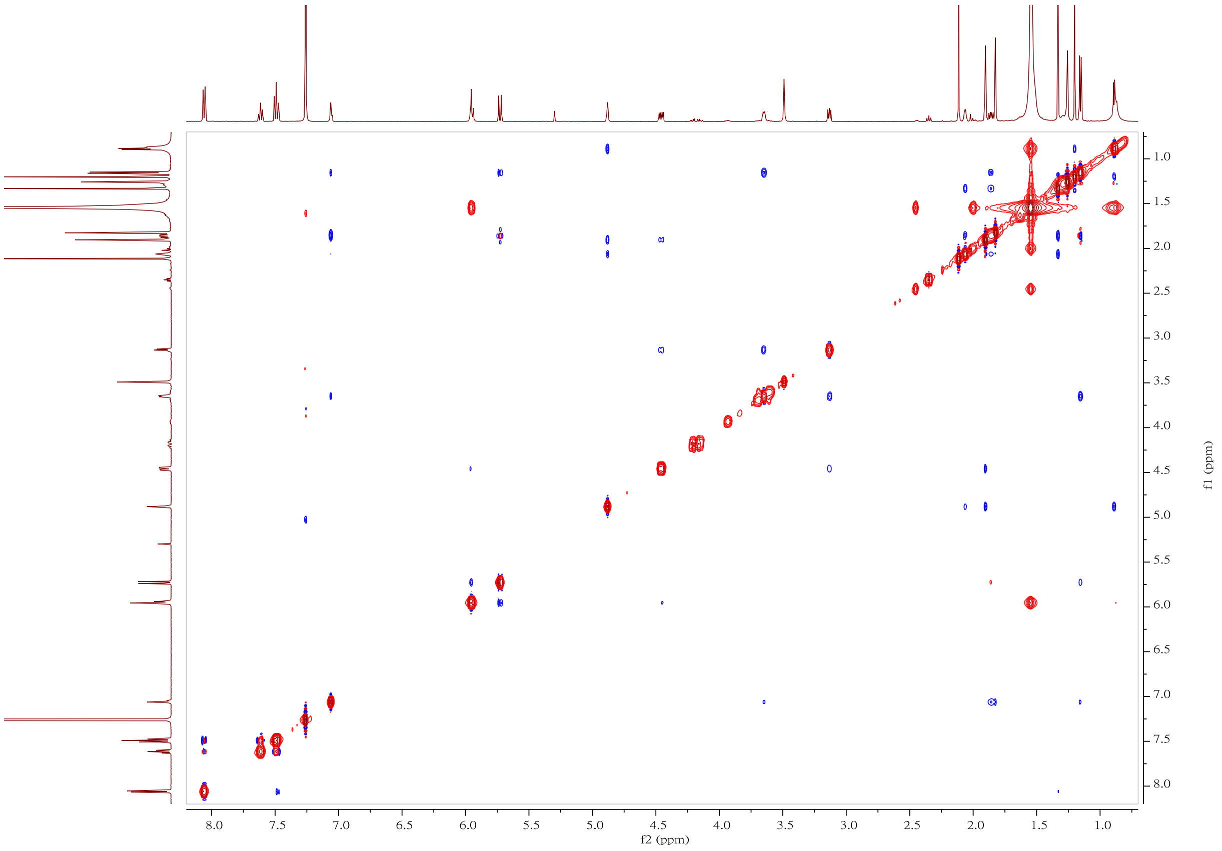

Figure S76. The NOESY spectrum of euphodendriane B (**15**).

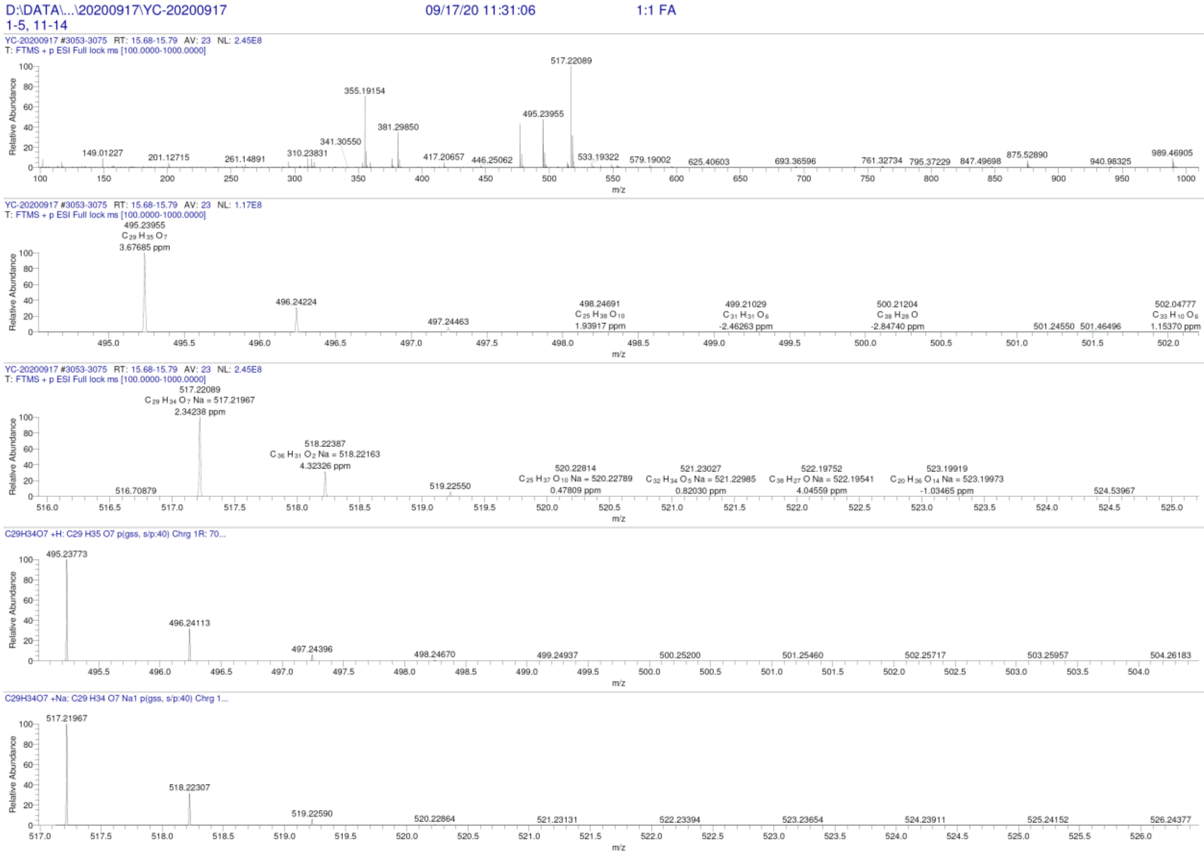

Figure S77. The HR-ESI-MS spectra of euphodendriane B (**15**).

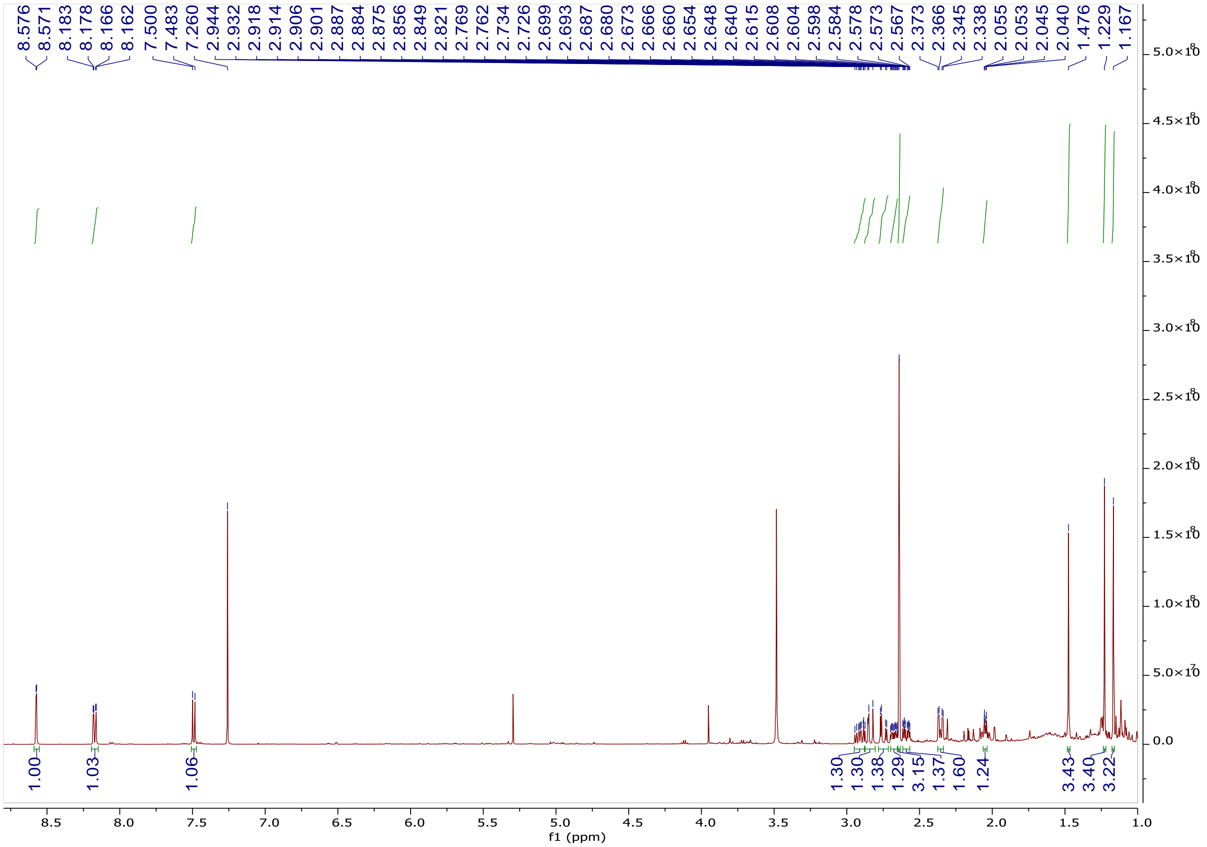

Figure S78. The ^{1}H-NMR spectrum of 16-nor-abieta-8,11,13-trien-3,7,15-trione (**16**) (500 MHz, $CDCl_3$).

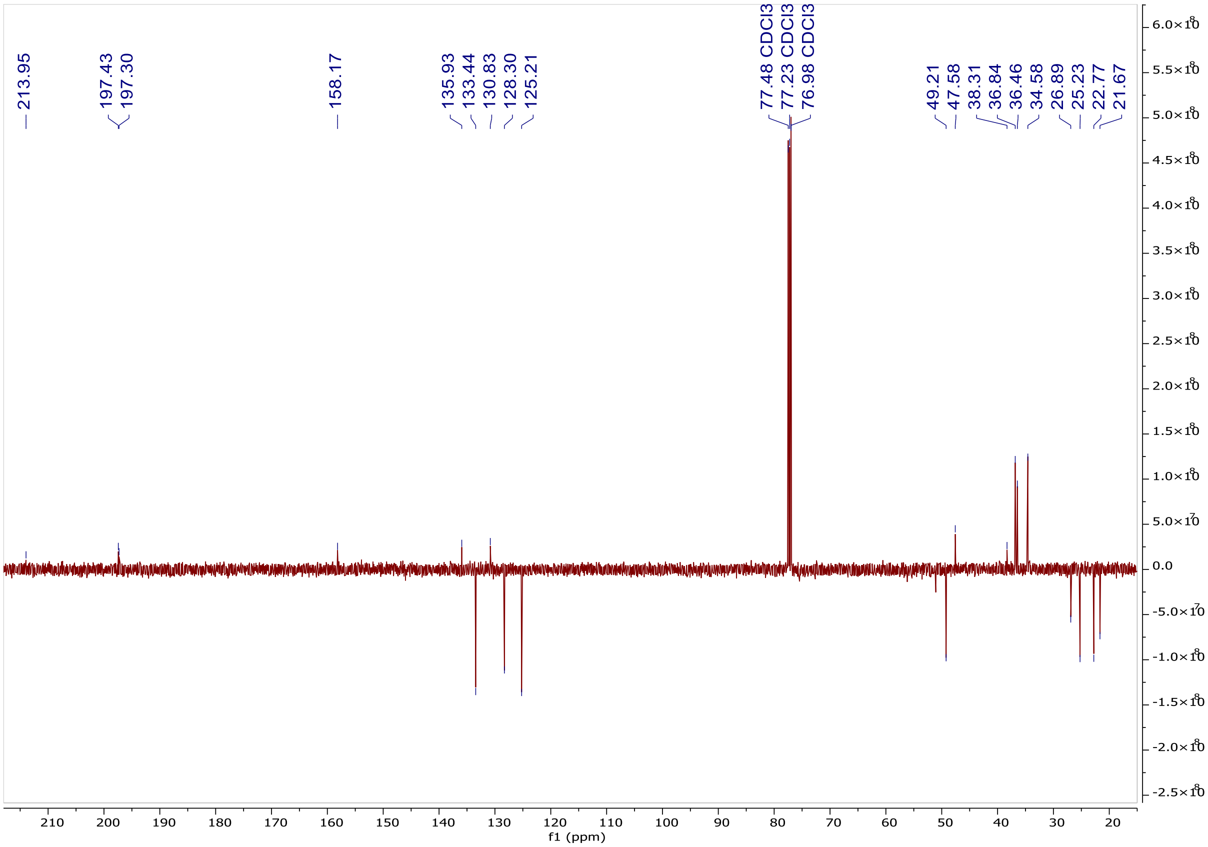

Figure S79. The ^{13}C-JMOD spectrum of 16-nor-abieta-8,11,13-trien-3,7,15-trione (**16**) (125 MHz, $CDCl_3$).

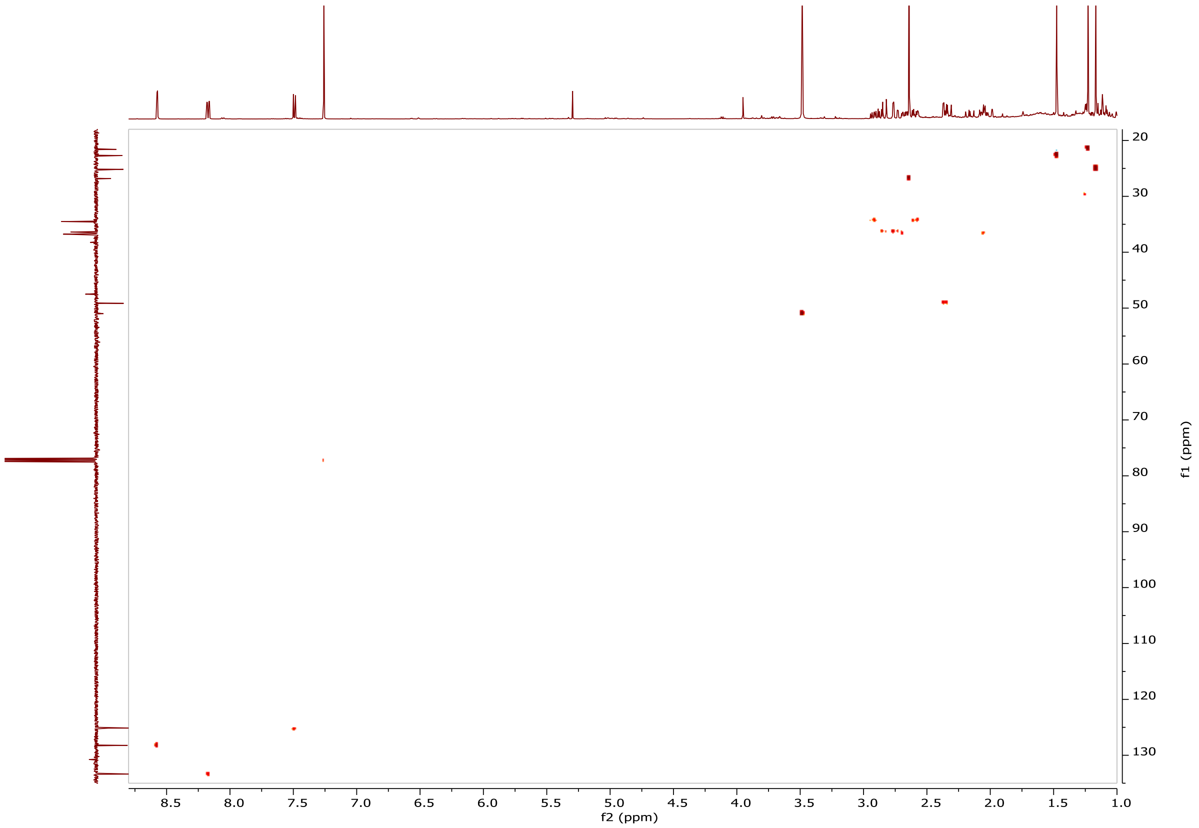

Figure S80. The HSQC spectrum of 16-nor-abieta-8,11,13-trien-3,7,15-trione (**16**).

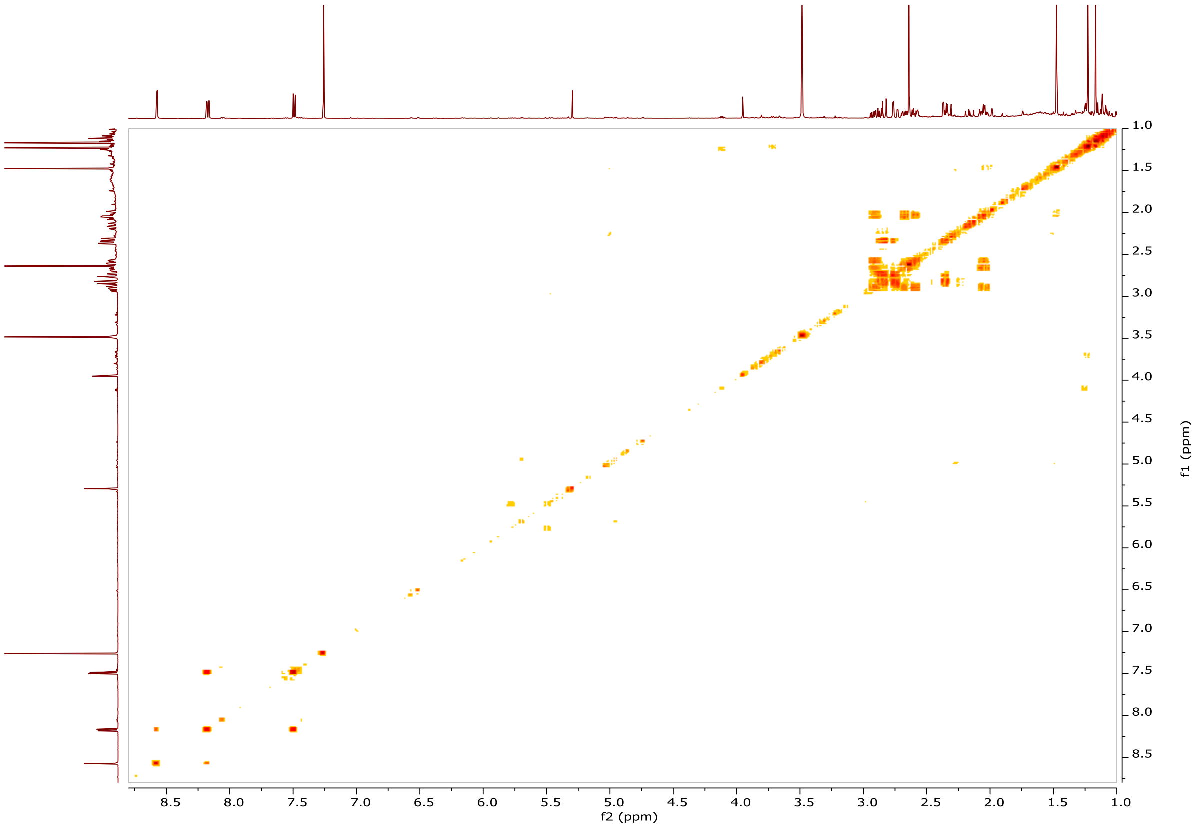

Figure S81. The ^1H-^1H COSY spectrum of 16-nor-abieta-8,11,13-trien-3,7,15-trione (**16**).

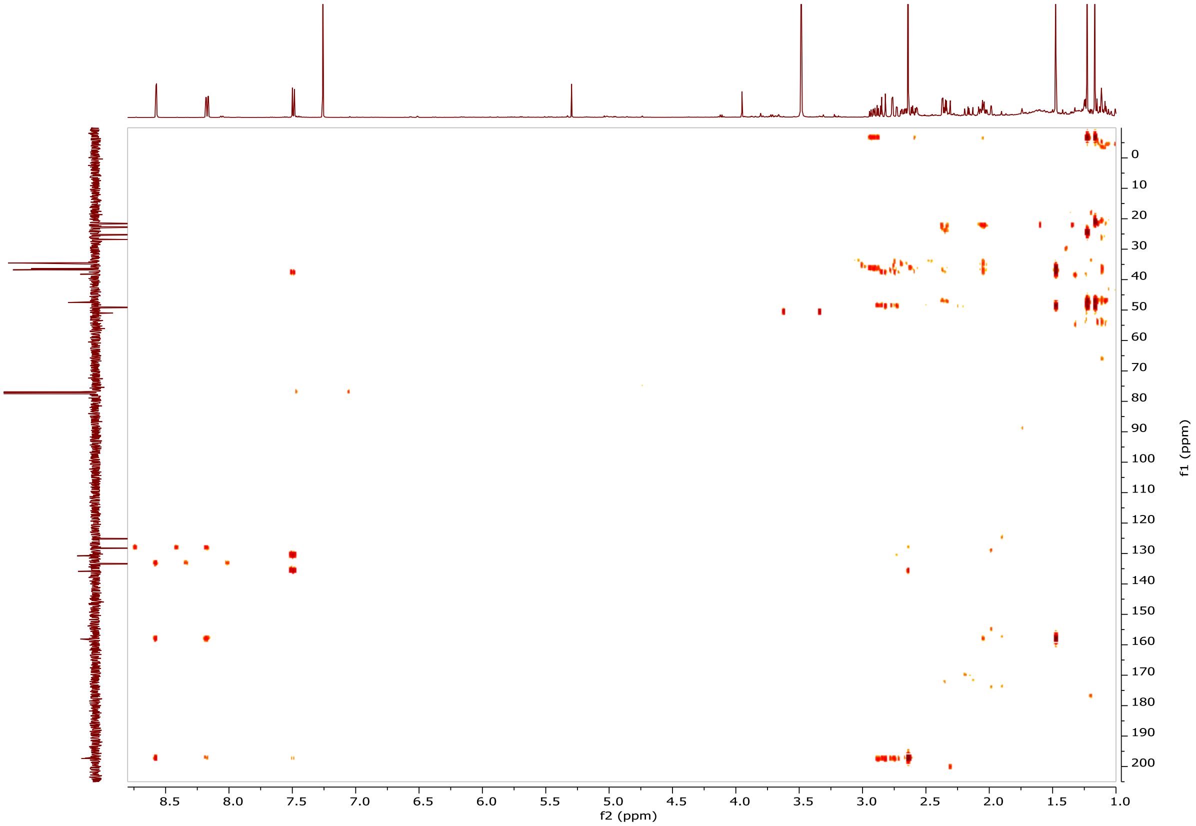

Figure S82. The HMBC spectrum of 16-nor-abieta-8,11,13-trien-3,7,15-trione (**16**).

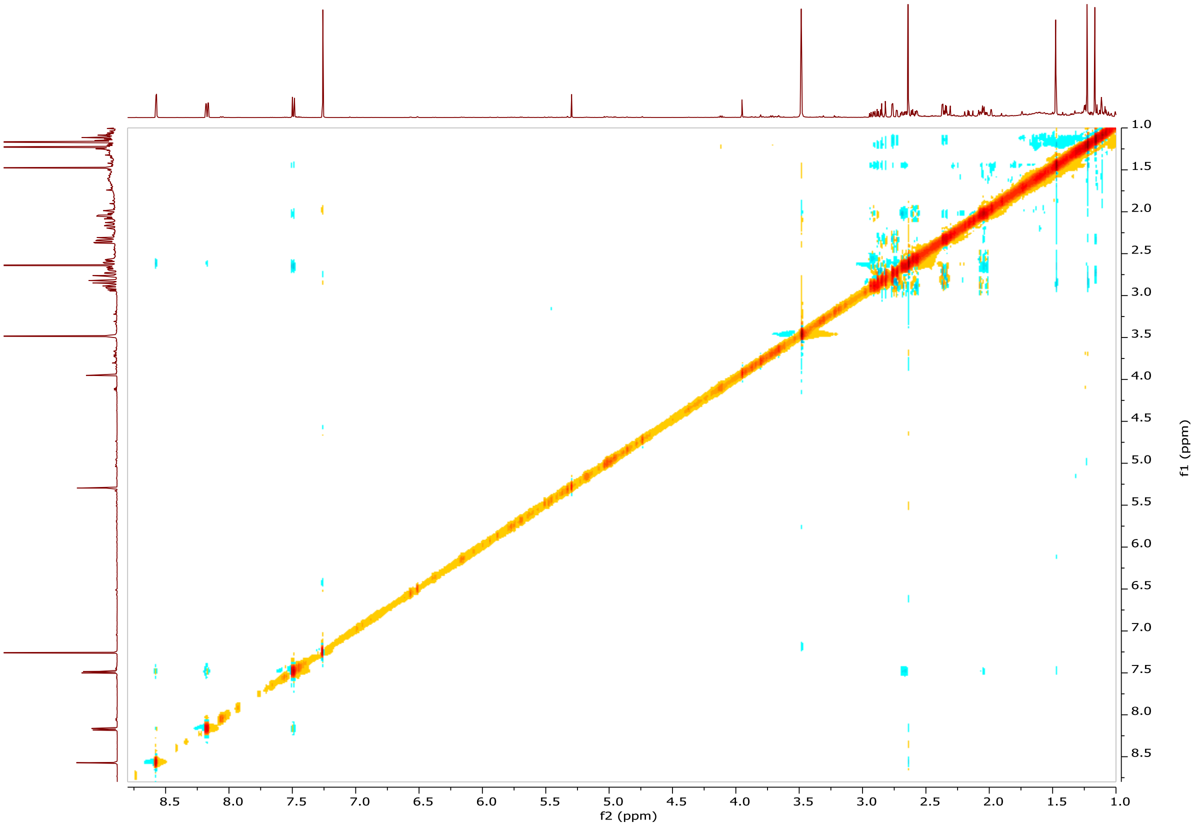

Figure S83. The NOESY spectrum of 16-nor-abieta-8,11,13-trien-3,7,15-trione (**16**).

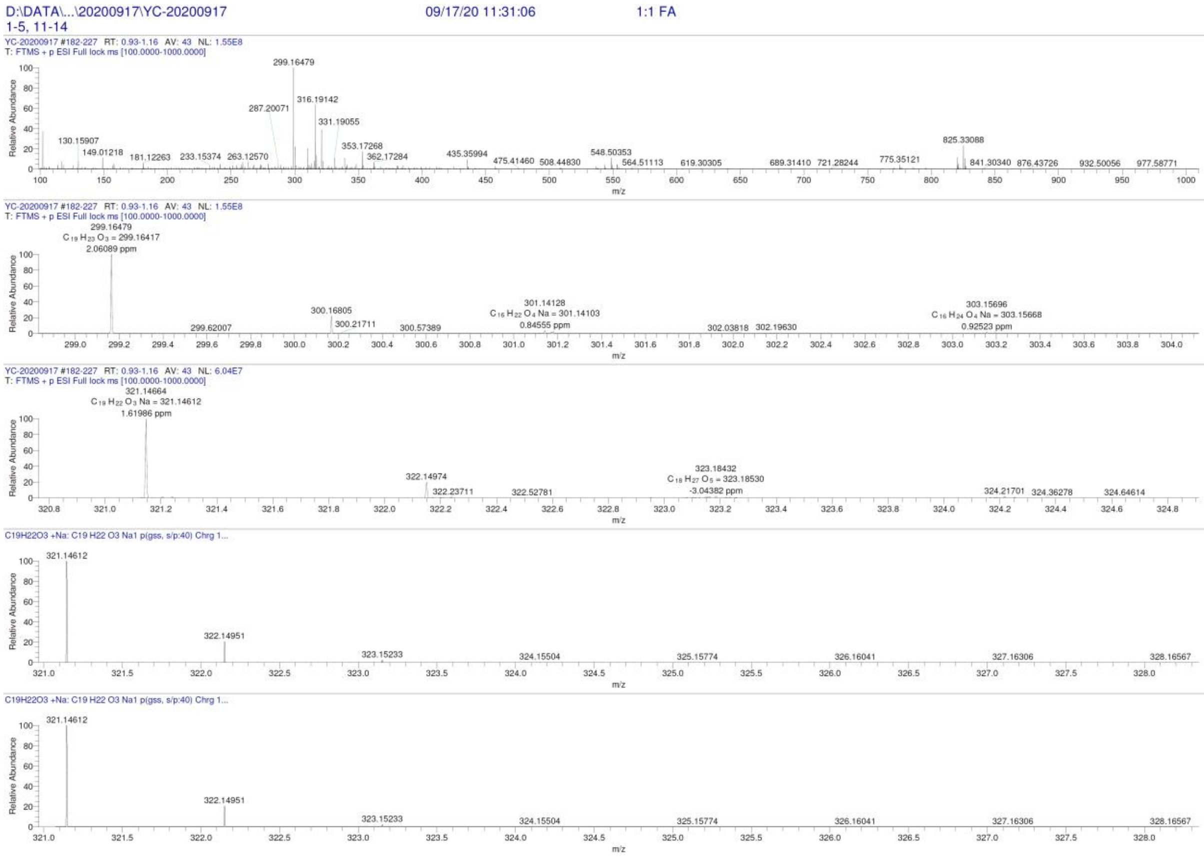

Figure S84. The HR-ESI-MS spectra of 16-nor-abieta-8,11,13-trien-3,7,15-trione (**16**).

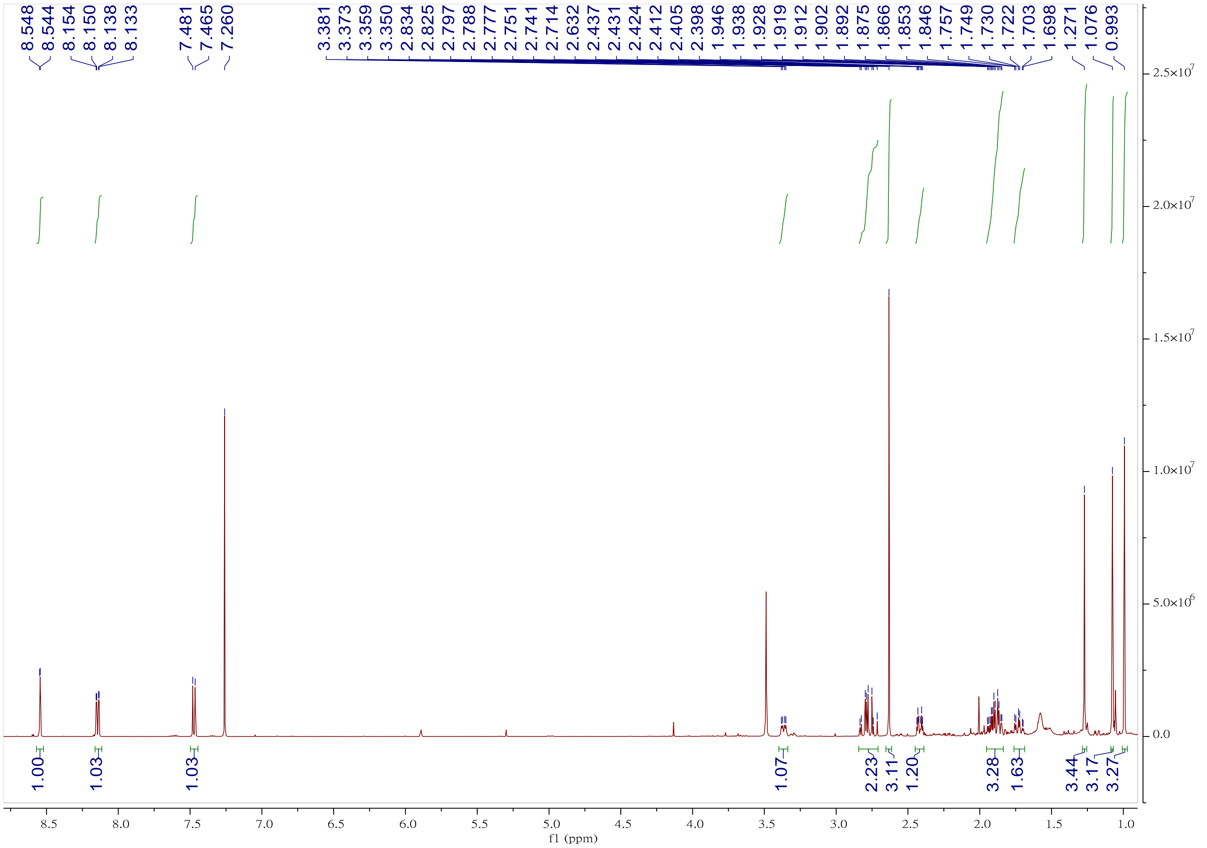

Figure S85. The ^{1}H-NMR spectrum of 16-nor-3*β*-hydroxy-abieta-8,11,13-trien-7,15-dione (**17**) (500 MHz, $CDCl_3$).

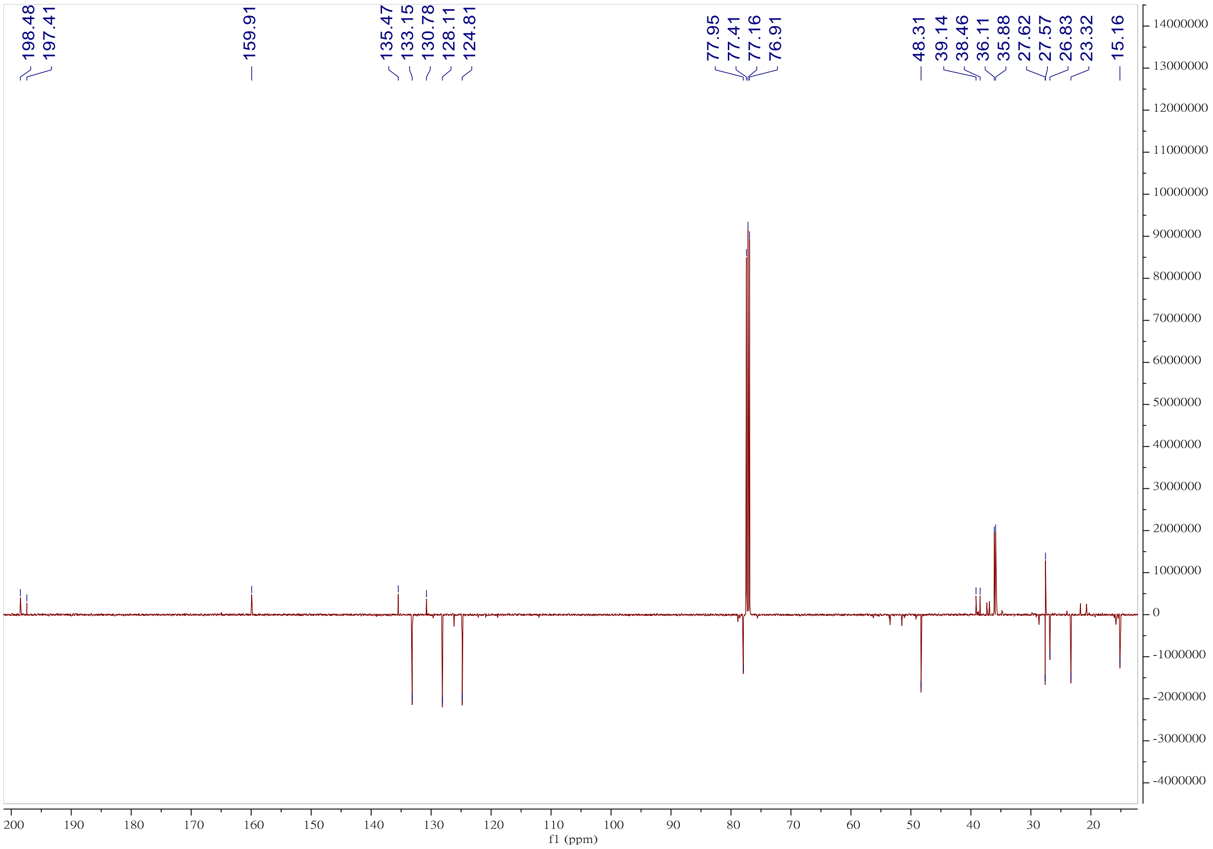

Figure S86. The ^{13}C-JMOD spectrum of 16-nor-3*β*-hydroxy-abieta-8,11,13-trien-7,15-dione (**17**) (125 MHz, $CDCl_3$).

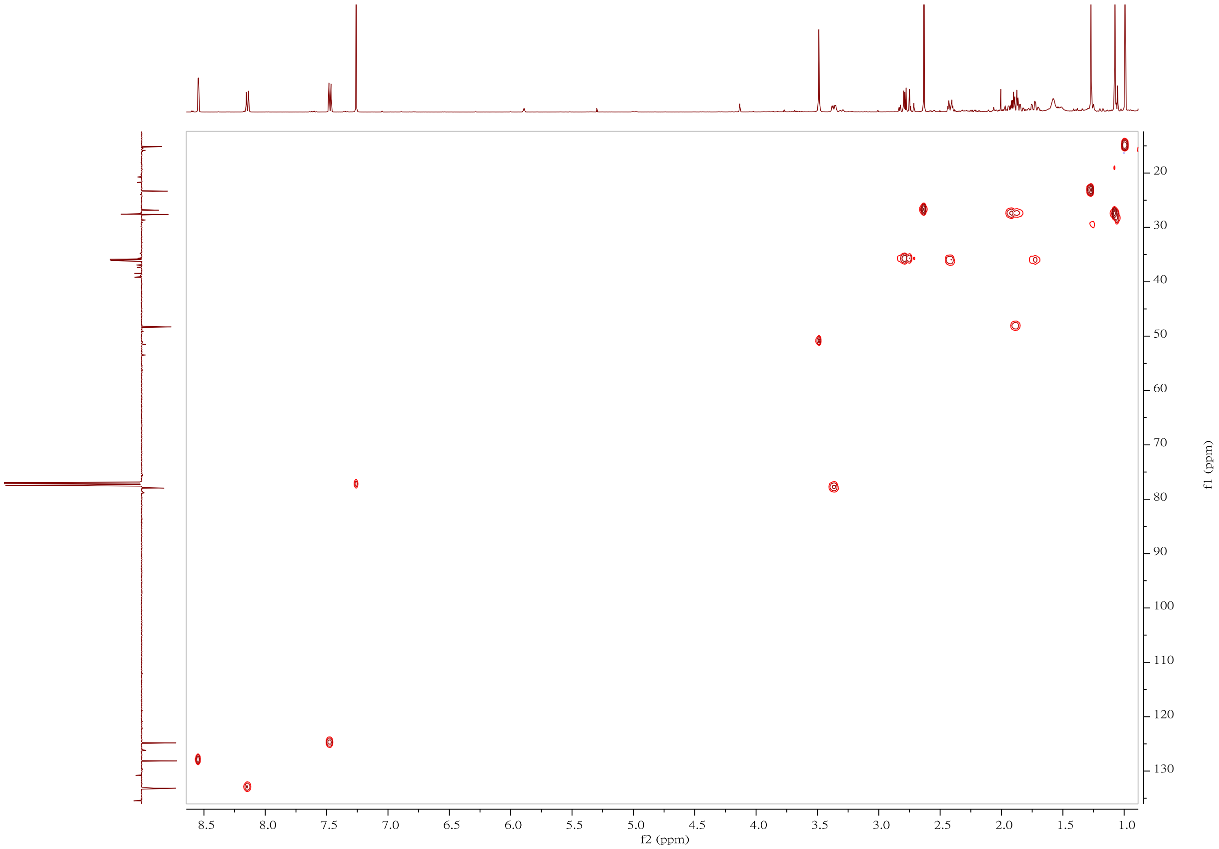

Figure S87. The HSQC spectrum of 16-nor-3*β*-hydroxy-abieta-8,11,13-trien-7,15-dione (**17**).

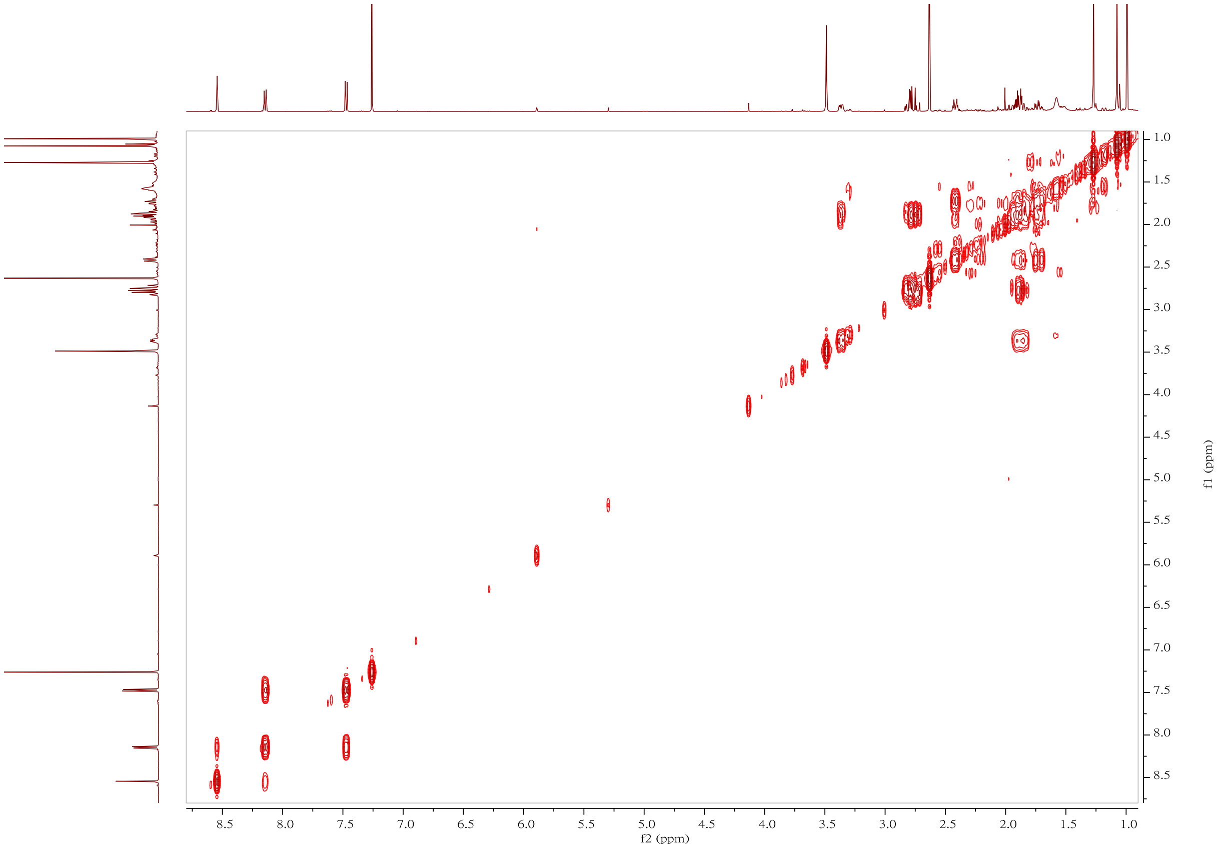

Figure S88. The ^1H-^1H COSY spectrum of 16-nor-3*β*-hydroxy-abieta-8,11,13-trien-7,15-dione (**17**).

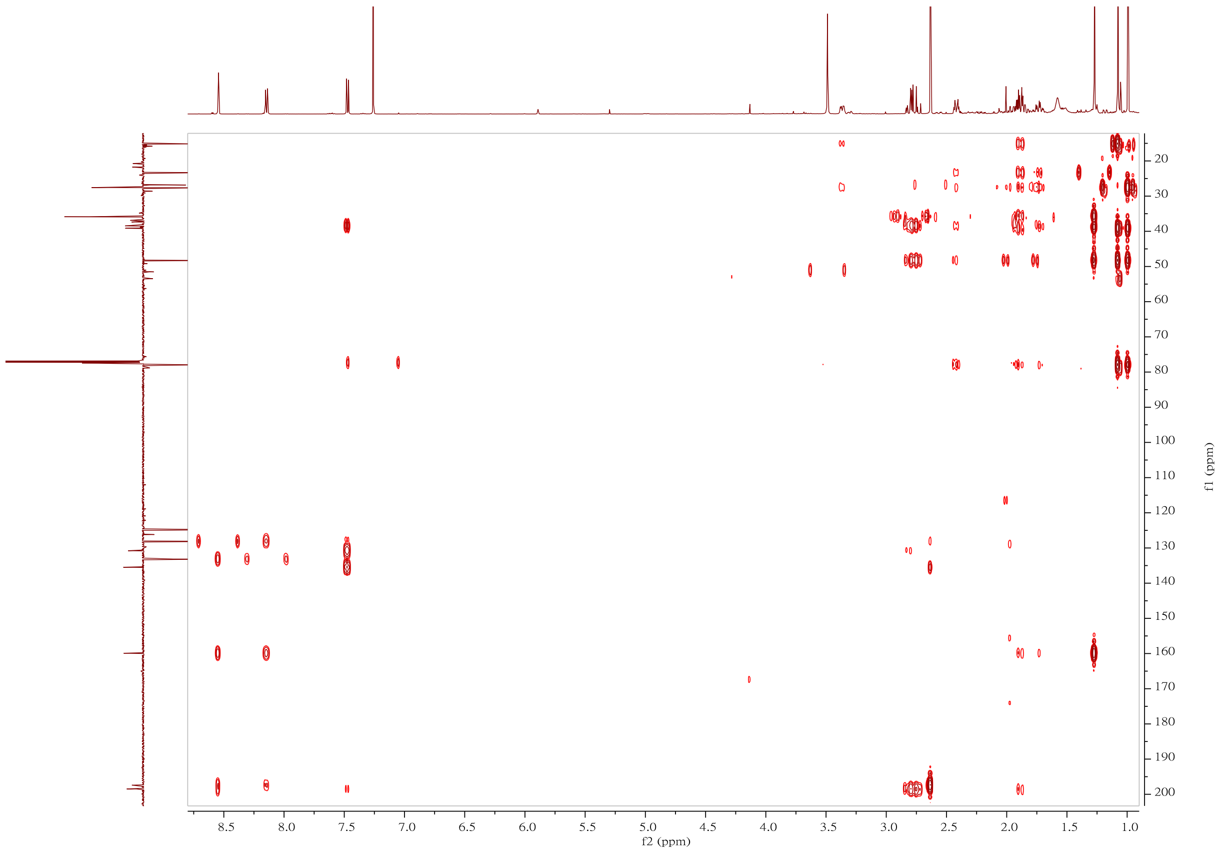

Figure S89. The HMBC spectrum of 16-nor-3*β*-hydroxy-abieta-8,11,13-trien-7,15-dione (**17**).

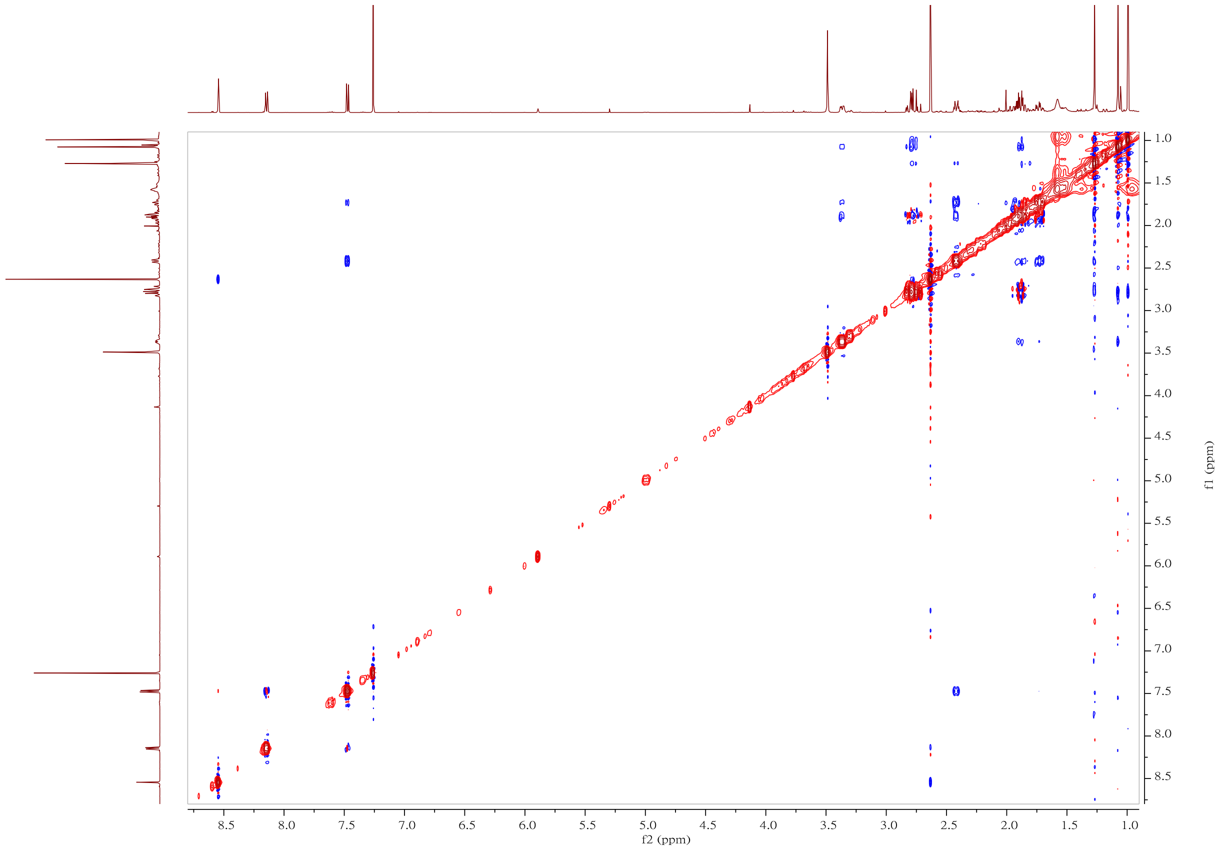

Figure S90. The NOESY spectrum of 16-nor-3*β*-hydroxy-abieta-8,11,13-trien-7,15-dione (**17**).

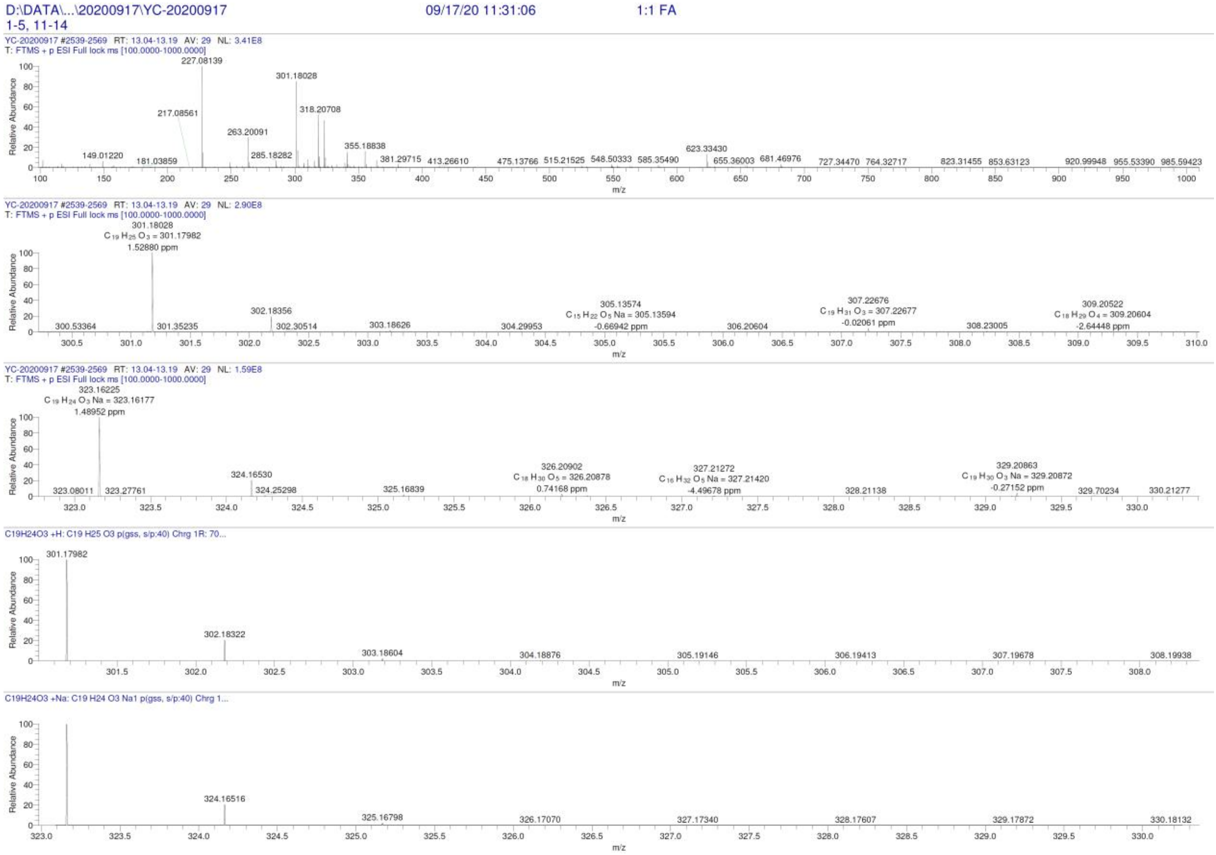

Figure S91. The HR-ESI-MS spectra of 16-nor-3*β*-hydroxy-abieta-8,11,13-trien-7,15-dione (**17**).

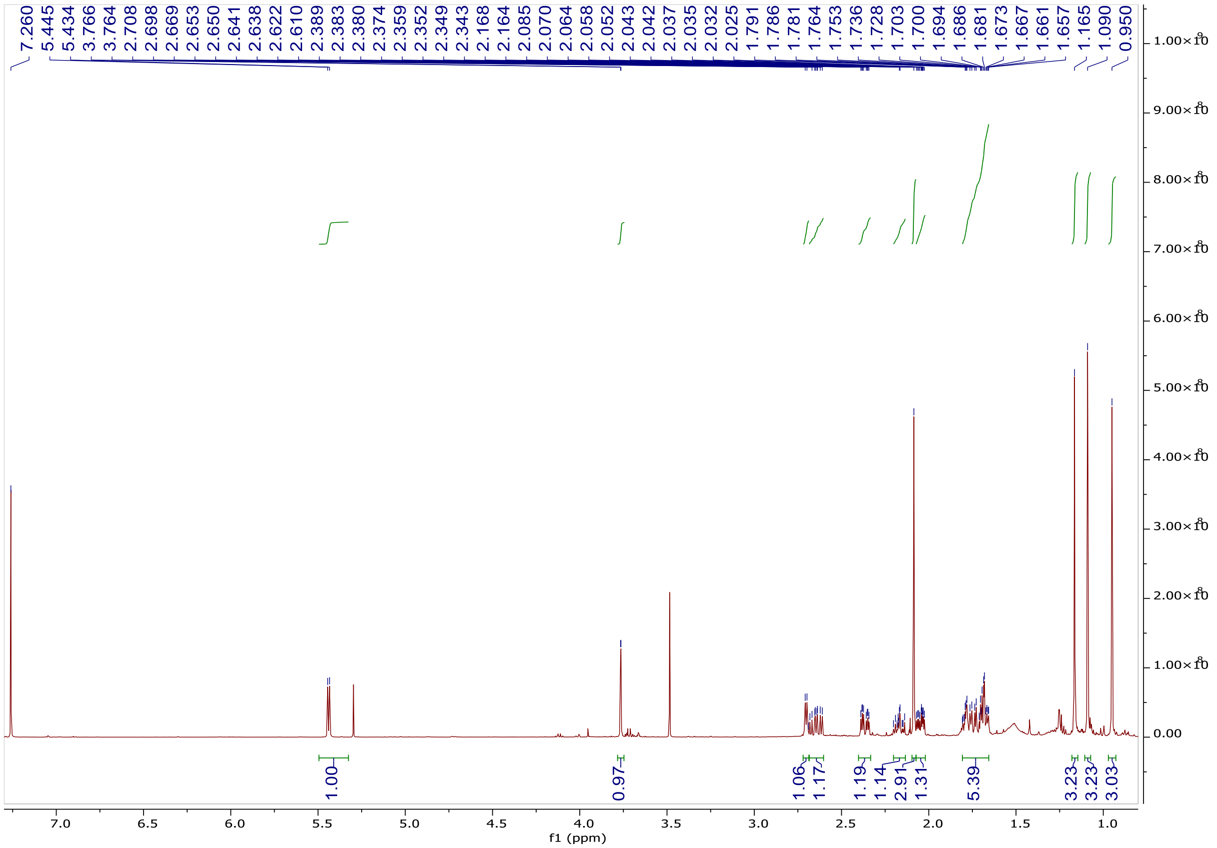

Figure S92. The ^{1}H-NMR spectrum of *ent*-8*β*,14*β*-epoxyabieta-3-one-11,13(15)-dien-16,12-olide (**19**) (500 MHz, $CDCl_3$).

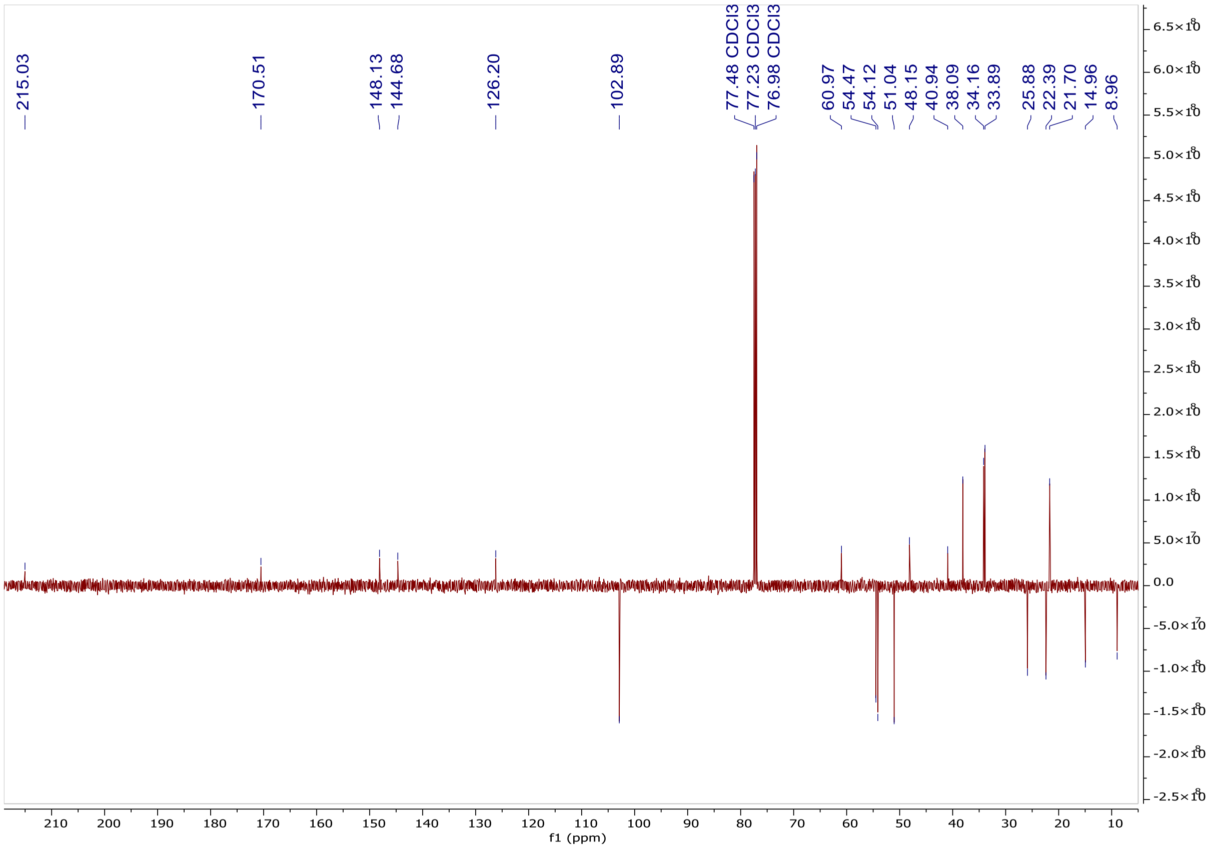

Figure S93. The ^{13}C-JMOD spectrum of *ent*-8*β*,14*β*-epoxyabieta-3-one-11,13(15)-dien-16,12-olide (**19**) (125 MHz, $CDCl_3$).

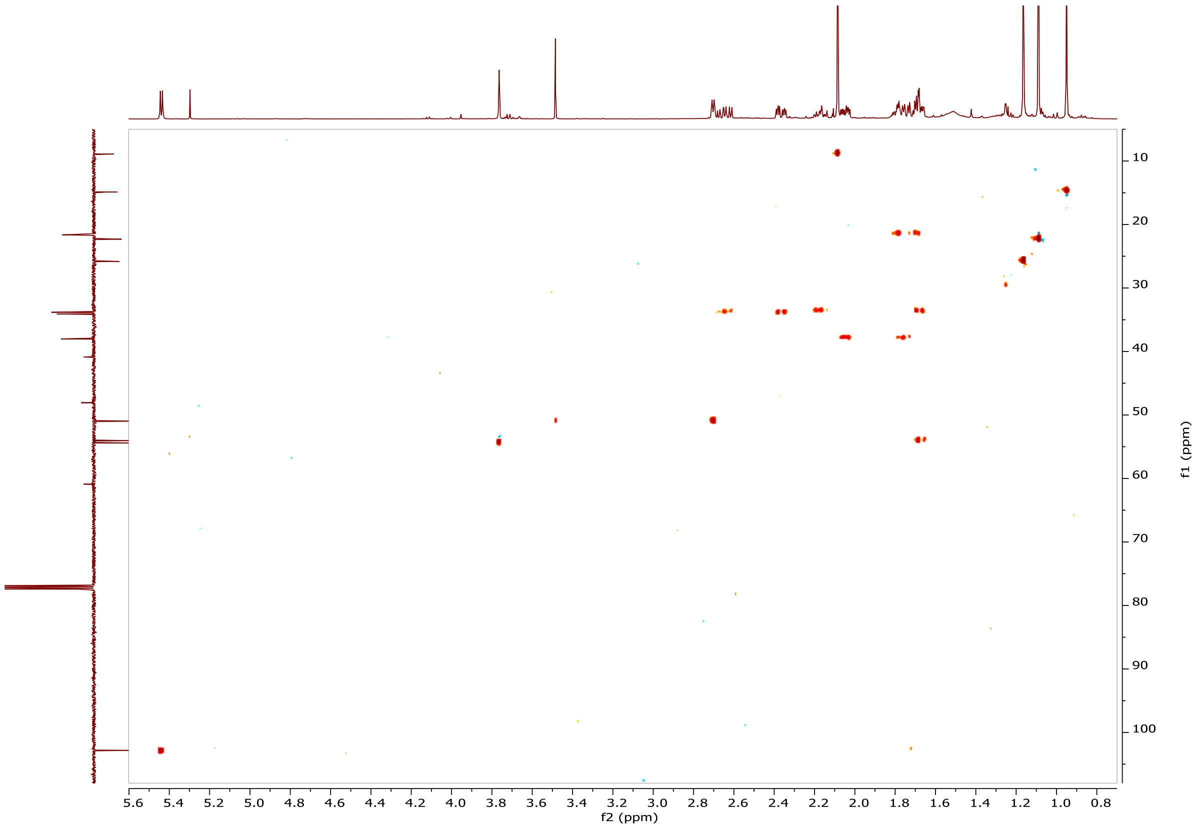

Figure S94. The HSQC spectrum of *ent*-8*β*,14*β*-epoxyabieta-3-one-11,13(15)-dien-16,12-olide (**19**).

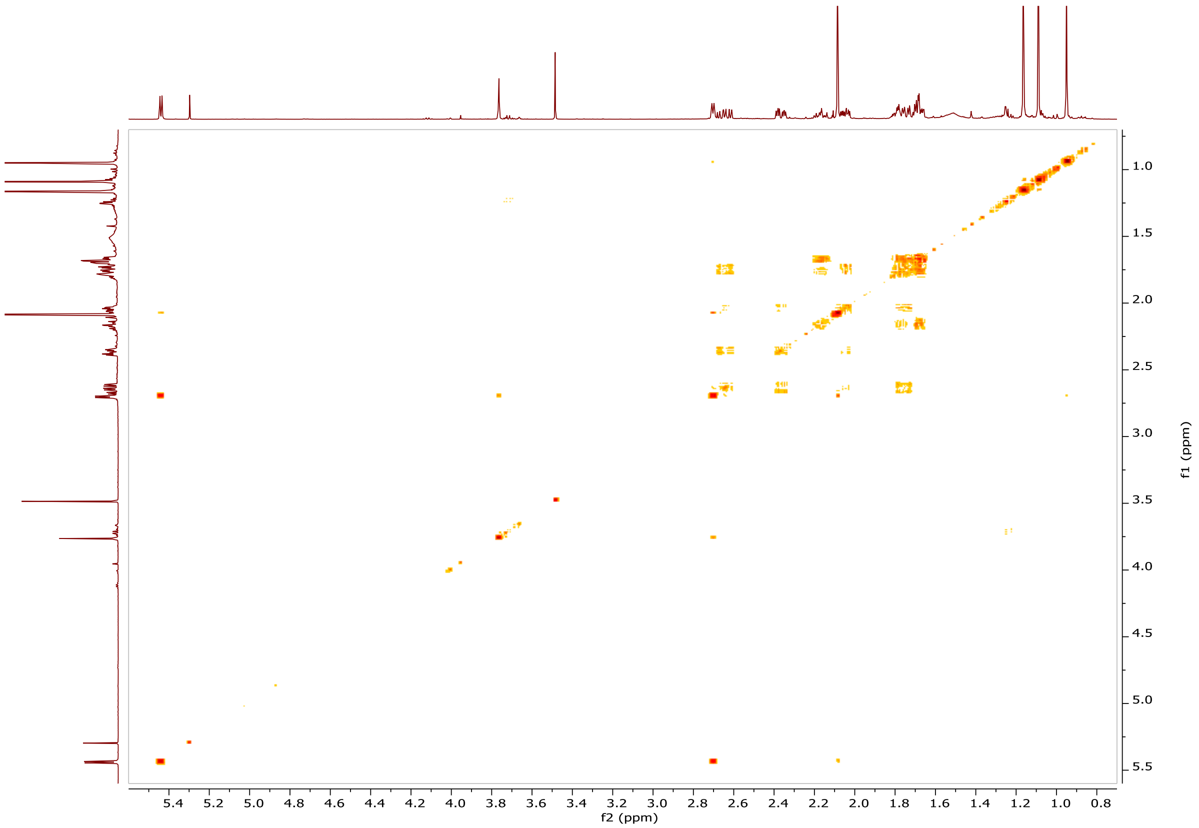

Figure S95. The ^1H-^1H COSY spectrum of *ent*-8*β*,14*β*-epoxyabieta-3-one-11,13(15)-dien-16,12-olide (**19**).

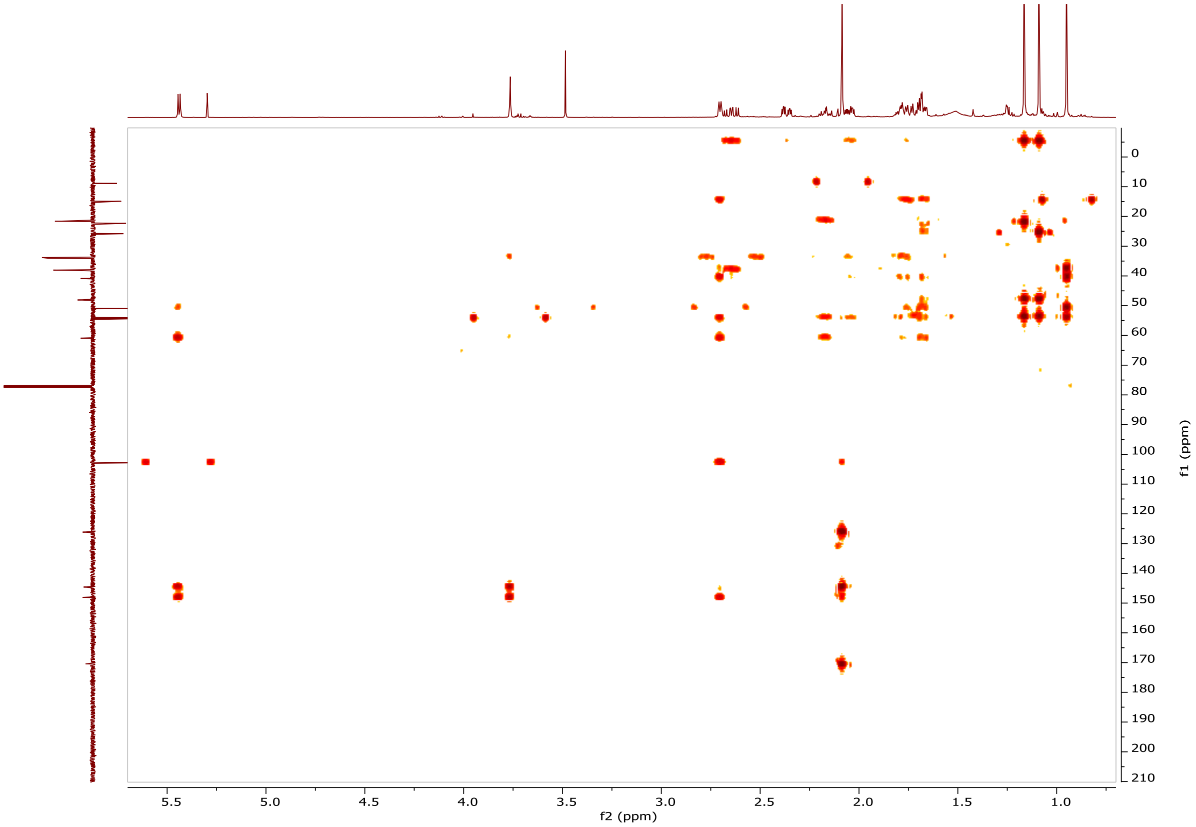

Figure S96. The HMBC spectrum of *ent*-8*β*,14*β*-epoxyabieta-3-one-11,13(15)-dien-16,12-olide (**19**).

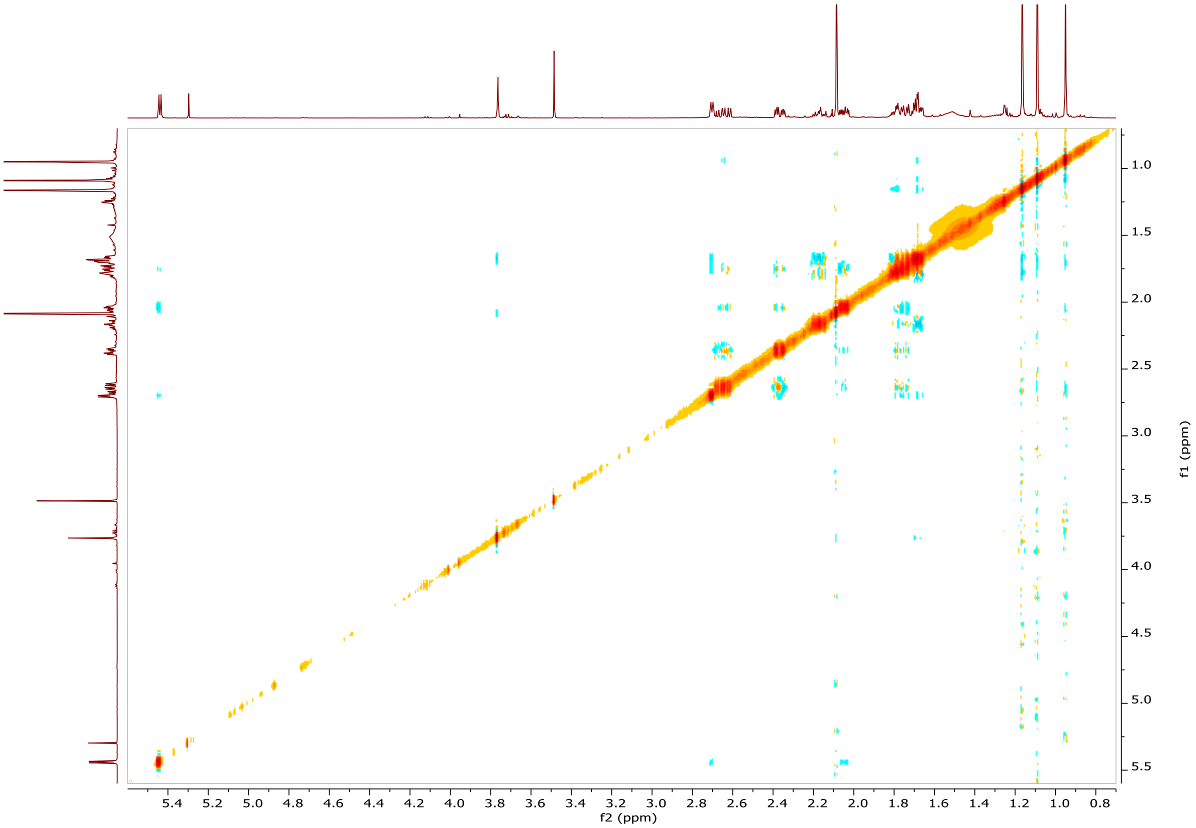

Figure S97. The NOESY spectrum of *ent*-8*β*,14*β*-epoxyabieta-3-one-11,13(15)-dien-16,12-olide (**19**).

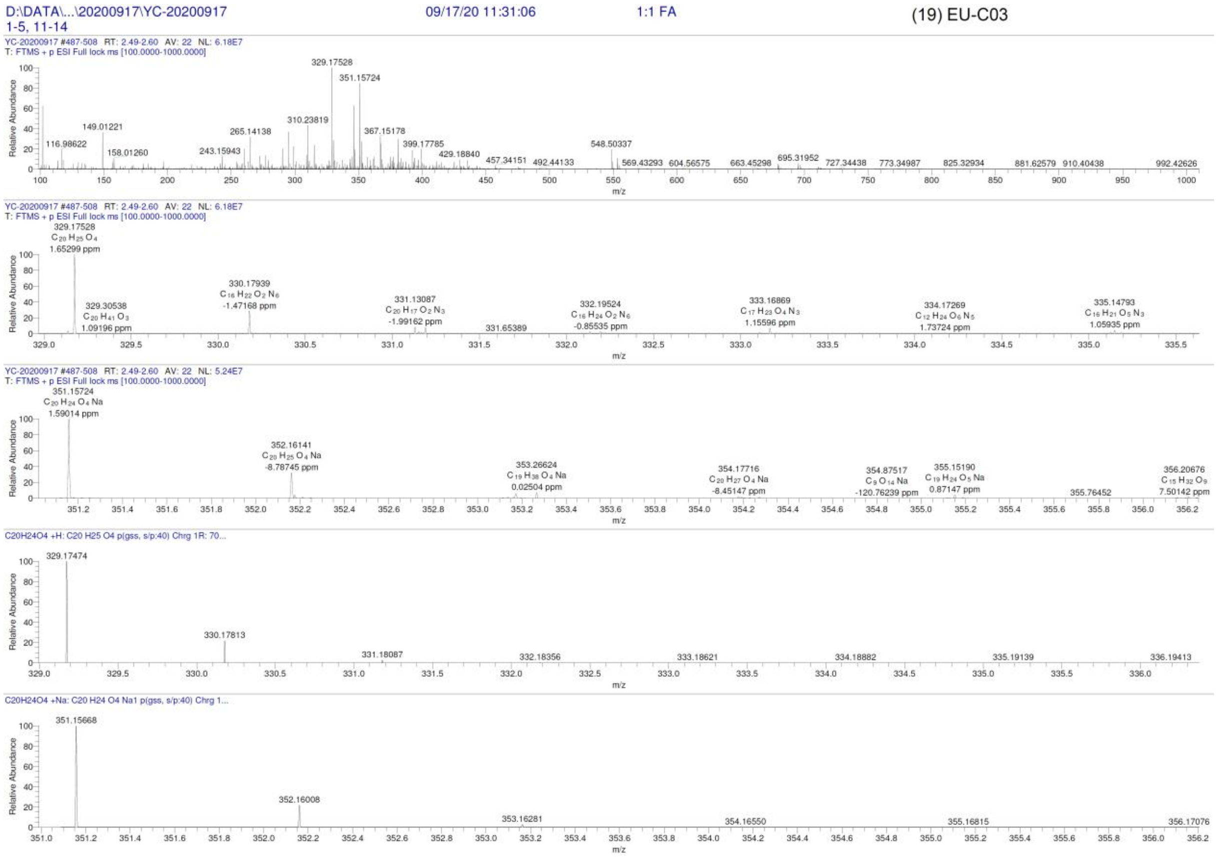

Figure S98. The HR-ESI-MS spectra of *ent*-8*β*,14*β*-epoxyabieta-3-one-11,13(15)-dien-16,12-olide (**19**).

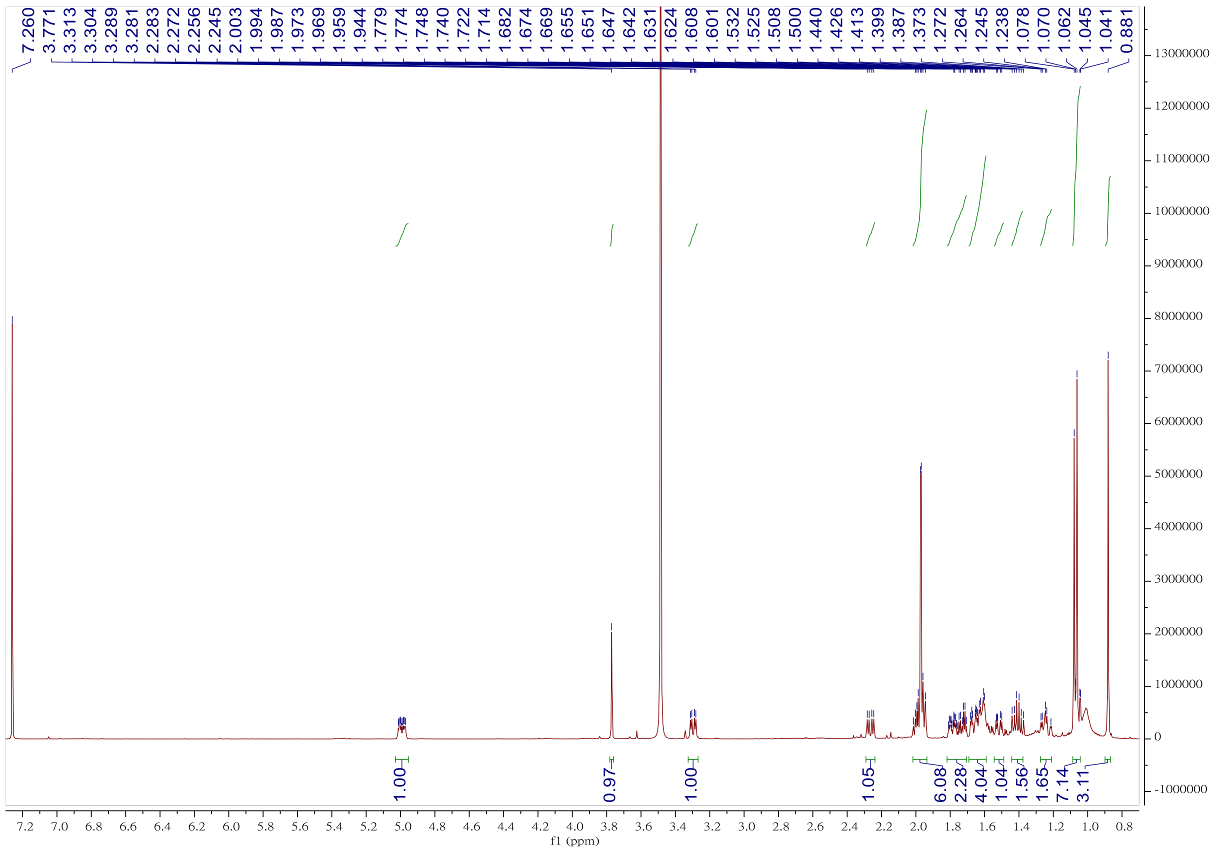

Figure S99. The ¹H-NMR spectrum of *ent*-8*β*,14*β*-epoxyabieta-3*α*-hydroxy-13(15)-en-16,12-olide (**20**) (500 MHz, $CDCl_3$).

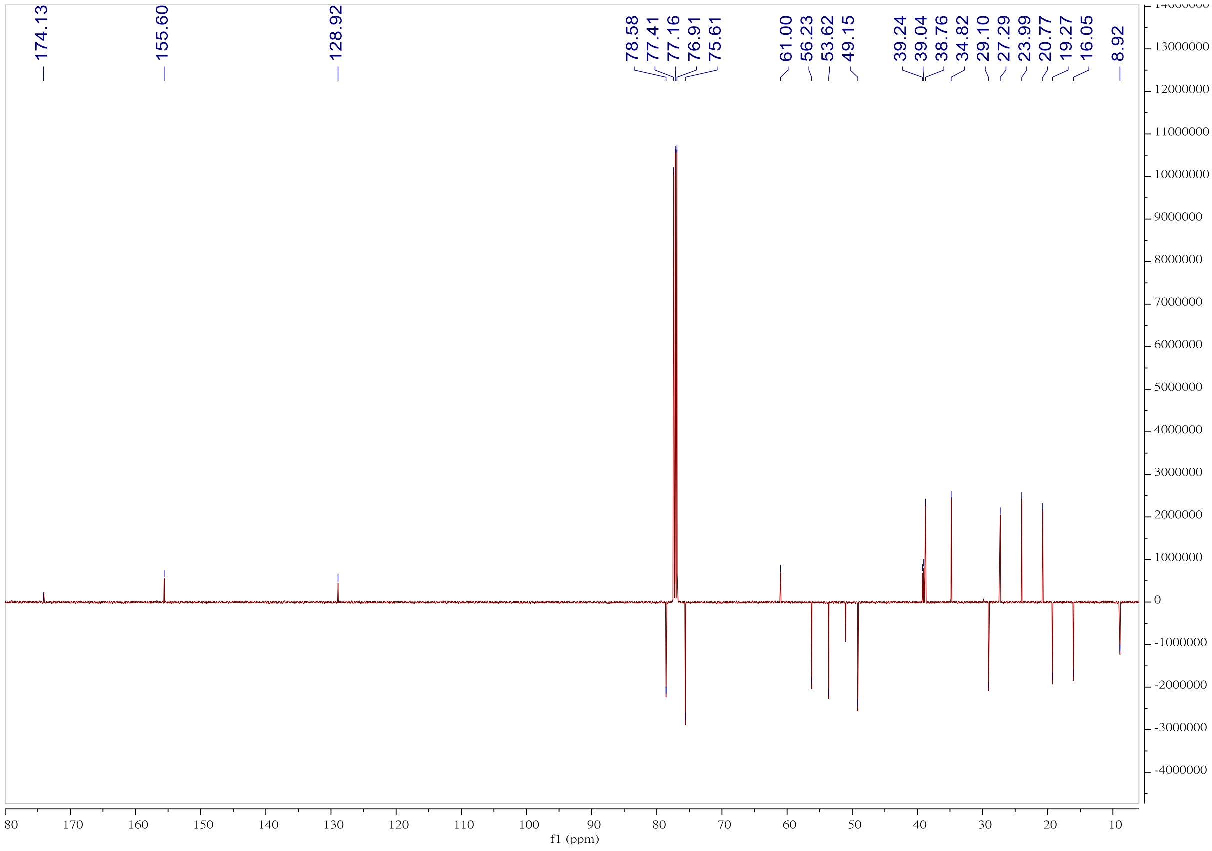

Figure S100. The ¹³C-JMOD spectrum of *ent*-8*β*,14*β*-epoxyabieta-3*α*-hydroxy-13(15)-en-16,12-olide (**20**) (125 MHz, $CDCl_3$).

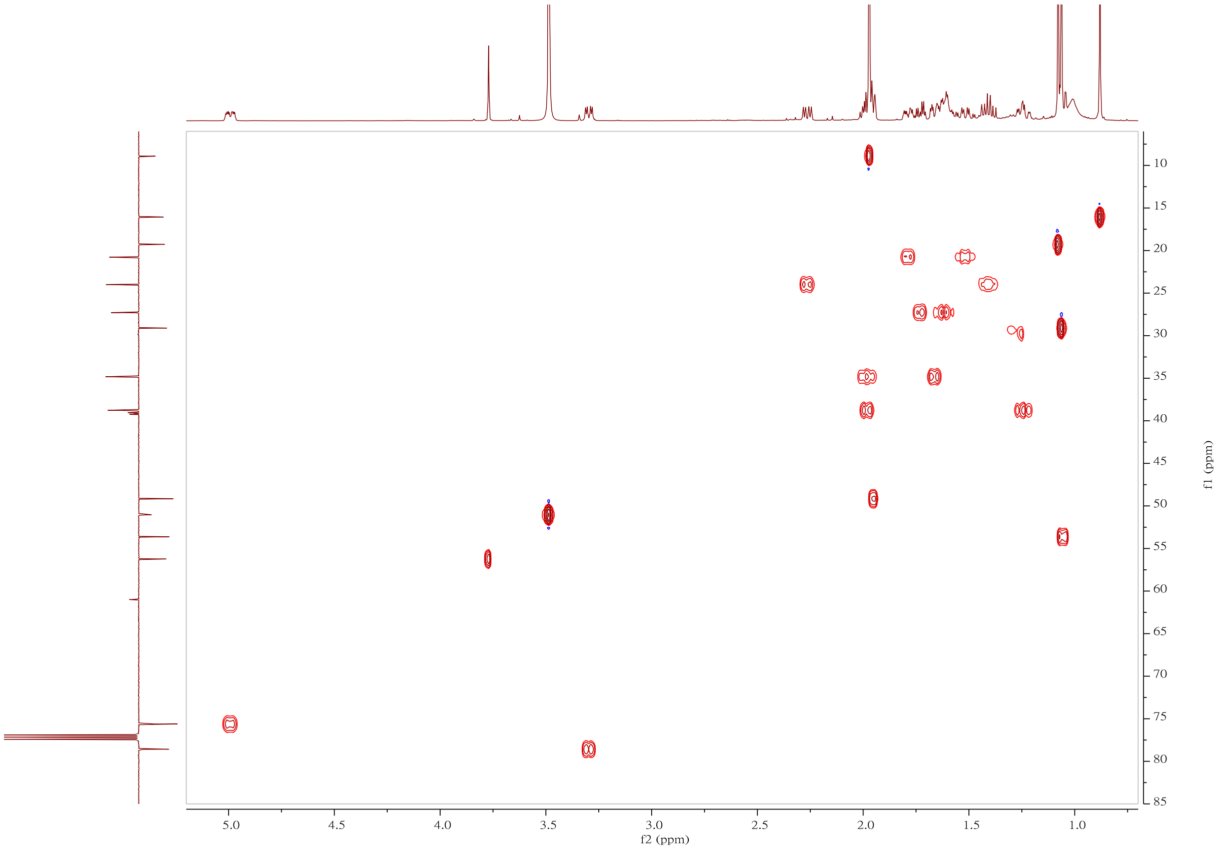

Figure S101. The HSQC spectrum of *ent*-8*β*,14*β*-epoxyabieta-3*α*-hydroxy-13(15)-en-16,12-olide (**20**).

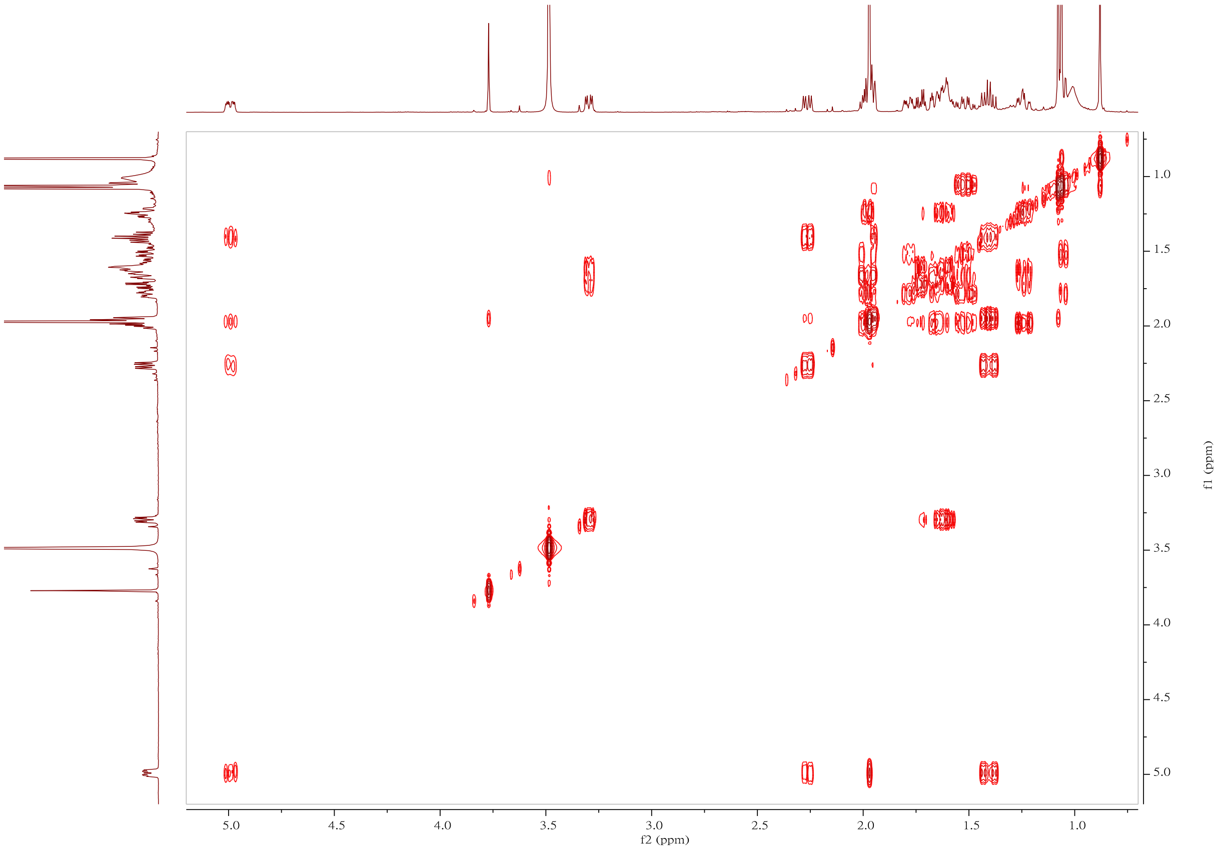

Figure S102. The ^{1}H-^{1}H COSY spectrum of *ent*-8*β*,14*β*-epoxyabieta-3*α*-hydroxy-13(15)-en-16,12-olide (**20**).

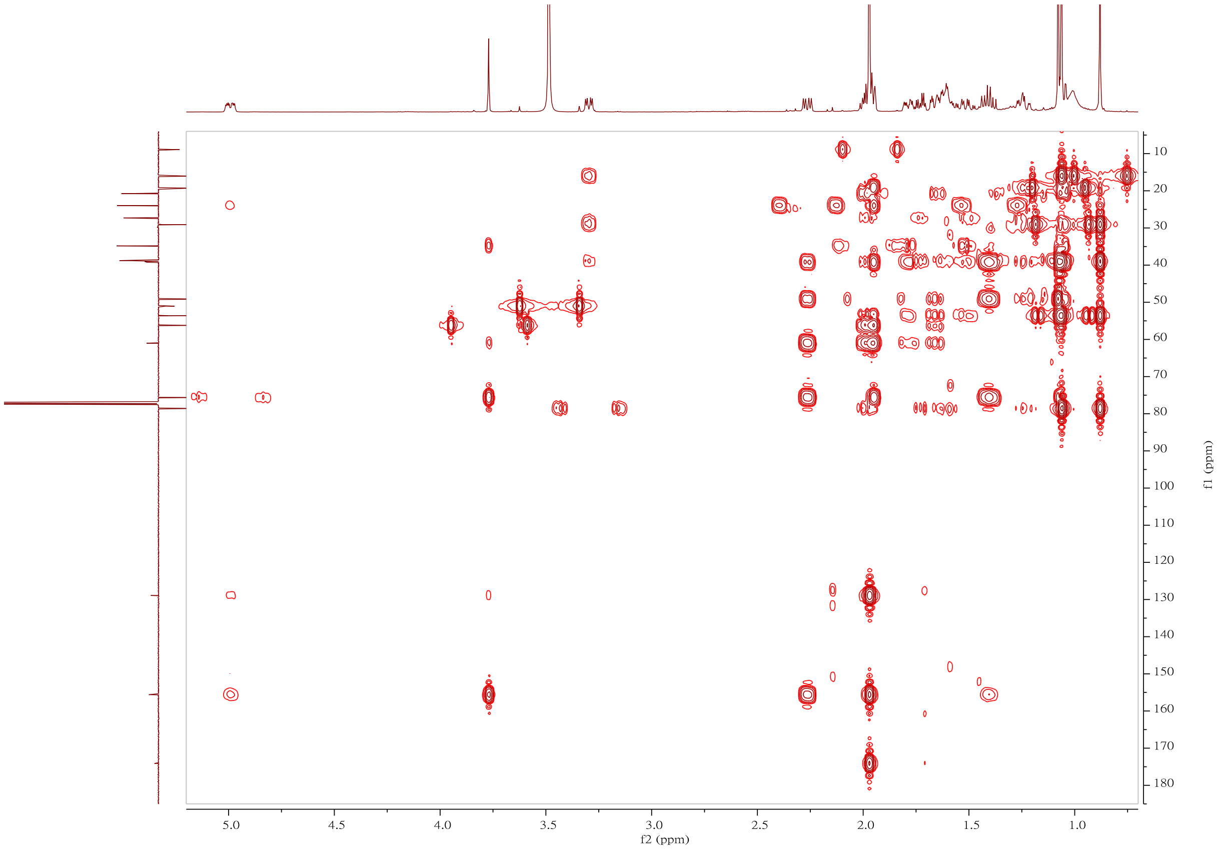

Figure S103. The HMBC spectrum of *ent*-8*β*,14*β*-epoxyabieta-3*α*-hydroxy-13(15)-en-16,12-olide (**20**).

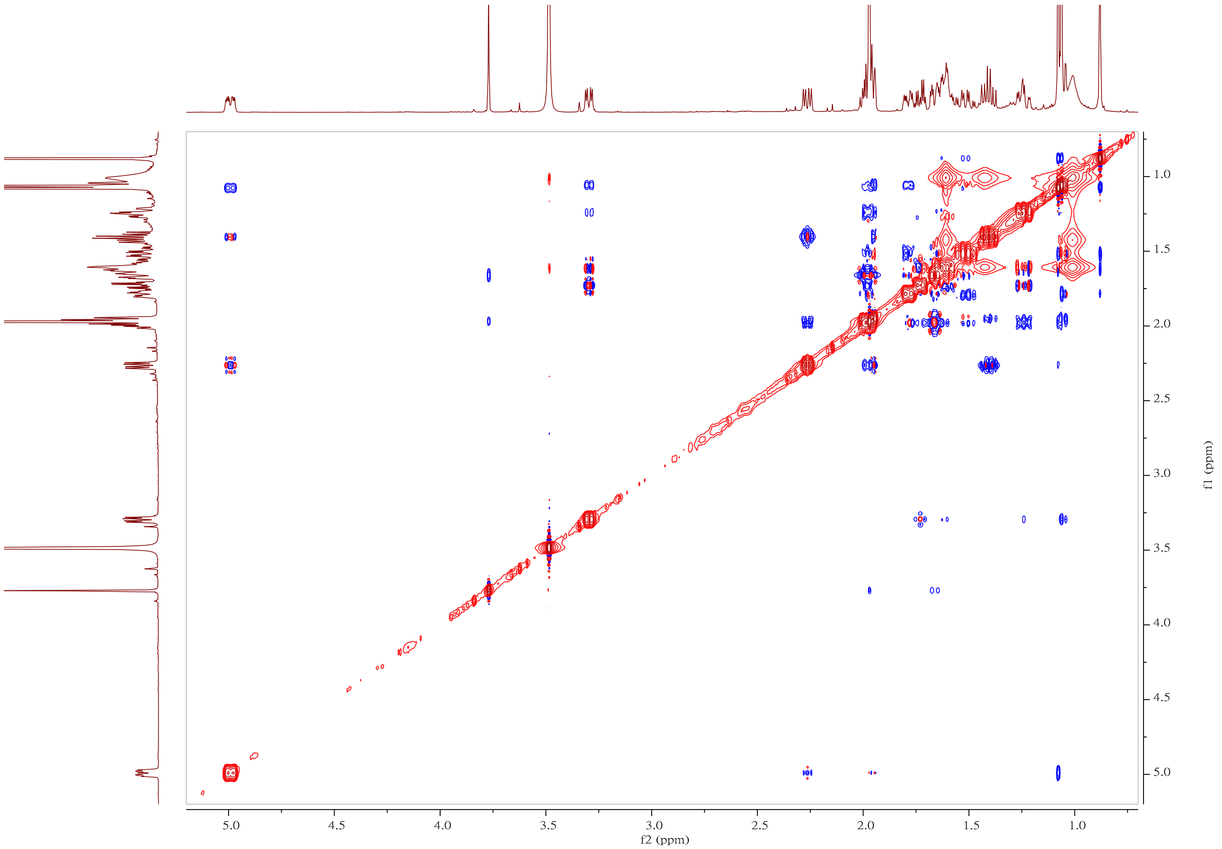

Figure S104. The NOESY spectrum of *ent*-8*β*,14*β*-epoxyabieta-3*α*-hydroxy-13(15)-en-16,12-olide (**20**).

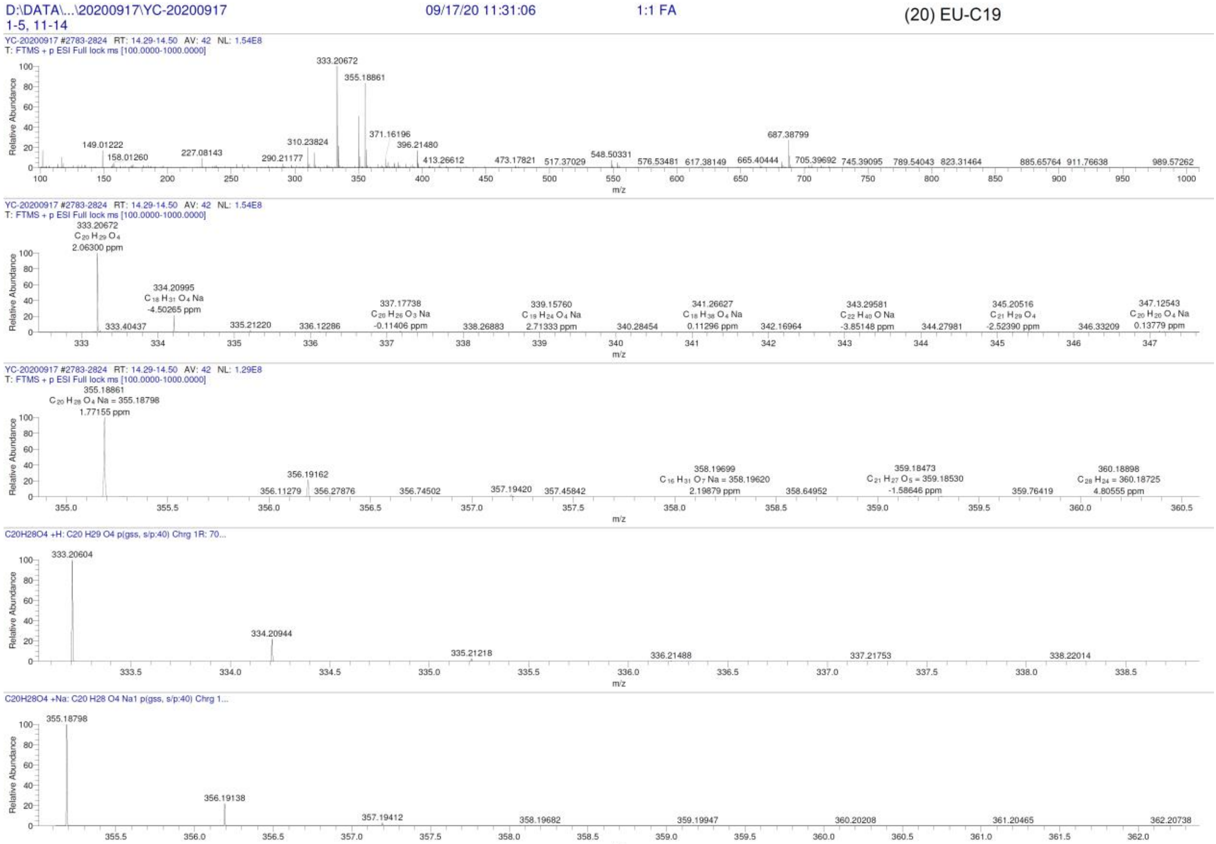

Figure S105. The HR-ESI-MS spectra of *ent*-8β,14β-epoxyabieta-3α-hydroxy-13(15)-en-16,12-olide (**20**).

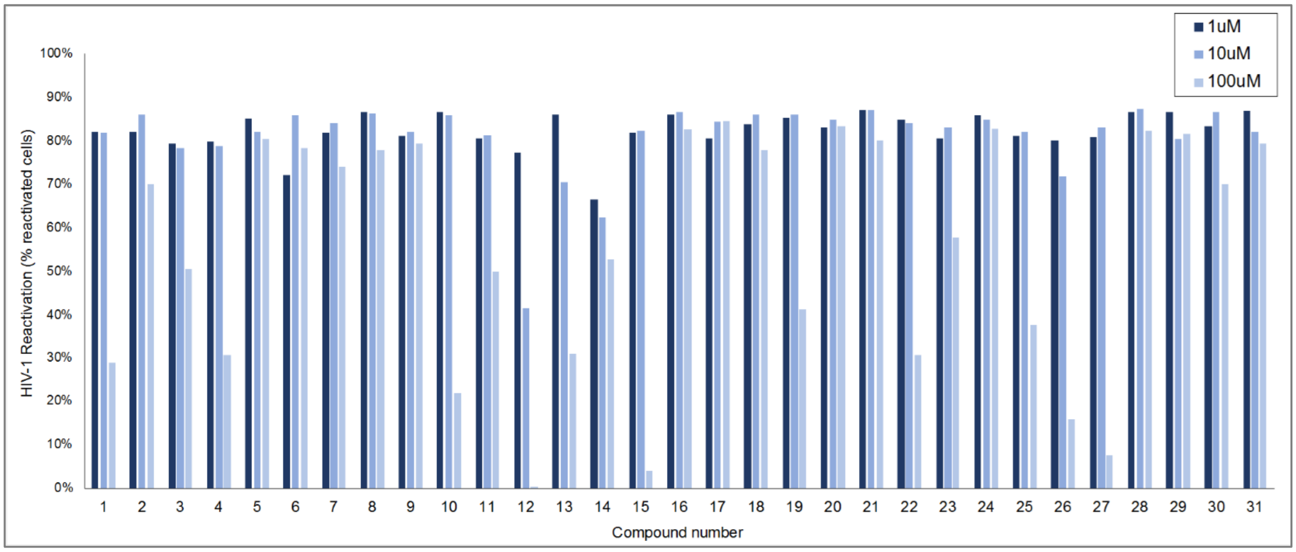

Figure S106. 24 hours cell viability after exposure to the compounds **1–31**.

All compounds were resuspended in DMSO at a concentration of 10 mM, and diluted with PBS and tested with J-lat 10.6 cells at concentrations of 100 µM, 10 µM, and 1 µM. 1% DMSO was used as a control to account for any effect of DMSO in the highest dilution of compounds.
